# π‑Extended Ru–COUBPY Photosensitizers for *In Vivo* Anticancer Phototherapy Using One-Photon 780 nm Near-Infrared Light

**DOI:** 10.1021/jacs.5c15343

**Published:** 2025-12-08

**Authors:** Diego Abad-Montero, Eduardo Izquierdo-García, Pierre Mesdom, Albert Gandioso, Elena de la Torre-Rubio, Manel Bosch, Juan Sanz-Villafruela, Alba Deyà, Marta Redrado, Valentin V. Novikov, José Luis Hernández, Jorge Galino, Marta E. Alberto, Antonio Francés-Monerris, Gilles Gasser, Vicente Marchán

**Affiliations:** † Departament de Química Inorgànica i Orgànica, Secció de Química Orgànica, Universitat de Barcelona (UB), 16724 Institut de Biomedicina de la Universitat de Barcelona (IBUB), Martí i Franquès 1-11, E-08028 Barcelona, Spain; ‡ Chimie ParisTech, PSL University, CNRS, Institute of Chemistry for Life and Health Sciences, Laboratory for Inorganic Chemical Biology, F-75005 Paris, France; § Unitat de Microscòpia Òptica Avançada, Centres Científics i Tecnològics, Universitat de Barcelona, Av. Diagonal 643, E-08028 Barcelona, Spain; ∥ Departamento de Química, Facultad de Ciencias, 16725 Universidad de Burgos, Plaza Misael Bañuelos s/n, E-09001 Burgos, Spain; ⊥ Health and Biomedicine Department, Leitat Technological Center, Carrer de la Innovació 2, E-08225 Terrassa, Spain; # Departament de Química Inorgànica i Orgànica, Secció de Química Inorgànica, Universitat de Barcelona (UB), and Institute of Nanoscience and Nanotechnology of the University of Barcelona (IN2UB), Martí i Franquès 1-11, E-08028 Barcelona, Spain; ¶ Dipartimento di Chimica e Tecnologie Chimiche, Università Della Calabria, Arcavacata di Rende I-87036, Italy; ∇ Institut de Ciència Molecular, 16781 Universitat de València, P.O. Box 22085, València 46071, Spain; ○ Serra-Húnter Professor at the Universitat de Barcelona, E-08028 Barcelona, Spain

## Abstract

Photodynamic therapy (PDT) is a promising cancer treatment modality, offering precise spatial and temporal control of drug activation using light. However, clinical translation of current photosensitizers (PSs) is limited by inefficient activation at wavelengths within the phototherapeutic window, especially in the deep-red and near-infrared (NIR) region. NIR light provides advantages such as reduced absorption by endogenous chromophores, minimized tissue photodamage, and improved tissue penetration, highlighting the need for PSs to be activatable in this range. Herein, we report a novel series of ruthenium­(II) polypyridyl complexes (**Ru4–7**) featuring π-extended COUBPY ligands, designed *via* a vinylogation strategy and synthesized through an innovative postcoordination ligand assembly approach. This structural modification enhances molar absorptivity and red-shifts the absorption bands well into the NIR region without substantially compromising photostability. Complexes **Ru4–7** efficiently generate both Type I and Type II reactive oxygen species, and their photodynamic activity, combined with preferential mitochondrial accumulation, leads to potent nanomolar phototoxicity against CT-26 colorectal cancer cells under deep-red and NIR irradiation, even under hypoxia. Notably, the lead complex **Ru6** demonstrated strong *in vivo* phototoxicity in mice bearing subcutaneous CT-26 tumors, achieving significant tumor growth inhibition upon irradiation with 660 and 780 nm light. **Ru6** thus represents one of the first Ru­(II) polypyridyl complexes to exhibit robust *in vivo* PDT antitumor activity under one-photon NIR activation. Its broad wavelength activation profile further underscores its potential versatility for treating tumors of varying size and anatomical location depending on specific light penetration requirements. These findings mark a promising step toward next-generation PSs for treating deep-seated and hypoxic tumors.

## Graphical abstract

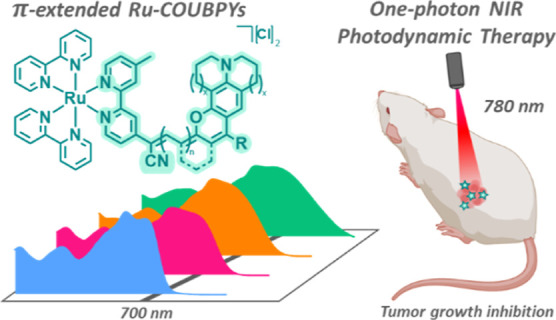

## Introduction

Photodynamic therapy (PDT) is a clinically approved cancer treatment modality that combines a photosensitizer (PS) with light of a specific wavelength, which is selected on the basis of the intended cancer indication and the required tissue penetration depth. The procedure involves local or systemic administration of a nontoxic dose of the PS drug, followed by localized illumination at the tumor site.^1,2^ Upon light activation, various highly cytotoxic reactive oxygen species (ROS) are generated through photochemical reactions involving either electron transfer (Type I) or energy transfer (Type II) mechanisms, leading to cellular damage and ultimately causing the death of cancer cells and the destruction of the associated tumor vasculature.^3–5^ The Type I mechanism produces species, such as superoxide (^•^O_2_^–^) and hydroxyl (^•^OH) radicals. Conversely, the Type II mechanism involves energy transfer from the triplet excited state of the PS to molecular oxygen, resulting in the production of singlet oxygen (^1^O_2_).^6^ Moreover, PDT can elicit immunomodulatory effects and induce additional vascular disruption, further enhancing its overall therapeutic efficacy.^7^

Light is a critical component of PDT, enabling precise spatial and temporal activation of the PS within tumor tissue, thereby minimizing off-target toxicity compared with conventional chemotherapy. Light within the phototherapeutic window (deep-red to near-infrared (NIR), 650–900 nm) offers several advantages over shorter-wavelength visible light, including minimal absorption by endogenous chromophores such as hemoglobin and melanin, reduced tissue photodamage, and greater tissue penetration depth (*e.g.*, NIR light can reach 1–2 cm).^8–11^ Consequently, achieving efficient absorption in this spectral region is a key consideration in the design and optimization of PSs, particularly for the PDT treatment of large solid tumors that require deeper penetration to reach the hypoxic tumor core.^12–14^

Ruthenium­(II) polypyridyl complexes have garnered significant interest in PDT, particularly with the advancement of TLD-1433 PS into phase II clinical trials for the intravesical green light treatment of nonmuscle-invasive bladder cancer (NMIBC) (NCT03945162).^15–18^ These compounds can access a diverse range of excited states and efficiently generate ROS upon light irradiation.^19^ Additionally, their octahedral geometry allows for the coordination of multiple ligands, as well as bioactive cargo molecules for photoactivated chemotherapy (PACT), providing structural versatility and enabling the tuning of their photophysical, photochemical, physicochemical and photobiological properties.^20–25^ While Ru­(II) polypyridyl complexes meet many criteria for an ideal PS, their low absorption within the phototherapeutic window remains a significant limitation for clinical translation. Most ruthenium complexes exhibit metal-to-ligand charge transfer (MLCT) absorption maxima below 500 nm, limiting their use to the treatment of superficial or highly localized small tumors.^26^ To address this limitation, various structural modification strategies have been developed to broaden the applicability of Ru-based PSs in PDT. These strategies include the expansion of the ligands’ π-conjugation system accessing excited states with longer lifetimes and different CT natures^27–29^ or conjugating them to far-red/NIR-absorbing organic fluorophores such as BODIPYs,^30^ cyanines,^31^ or coumarin dyes, including COUPY derivatives.^32,33^ Despite these advances, the number of Ru­(II) polypyridyl complexes demonstrating effective performance in the NIR spectral region using one-photon activation remains limited.^31,34–36^ Recently, various Ru­(II) complexes compatible with two-photon PDT have been reported as an alternative to achieve NIR activation.^37–39^ However, this technique presents several intrinsic challenges that limit its clinical applicability, including the need for highly specialized and expensive femtosecond lasers capable of delivering the required photon density, the relatively low efficiency of two-photon absorption (TPA) processes, and the potential phototoxicity associated with the high energy densities used.

Recently, we reported a new family of PSs^40^ based on Ru­(II) polypyridyl complexes incorporating coumarin-based COUBPY ligands derived from COUPY dyes^41–45^ that exhibit potent *in vitro* cytotoxicity against cancer cells upon irradiation with visible light (*i.e.*, green to deep-red), while remaining nontoxic in the dark. Among them, **SCV42** and **SCV49**, here referred to as **Ru1** and **Ru3**, respectively (Figure 1A), demonstrated nanomolar photoactivity under both normoxic (21% O_2_) and hypoxic (2% O_2_) conditions. Notably, **Ru3** exhibited a favorable pharmacokinetic profile, excellent *in vivo* toxicological tolerability, and potent tumor growth inhibition in mice bearing subcutaneous colorectal tumors (CT-26) following deep-red light irradiation at 660 nm.

**Figure 1:**
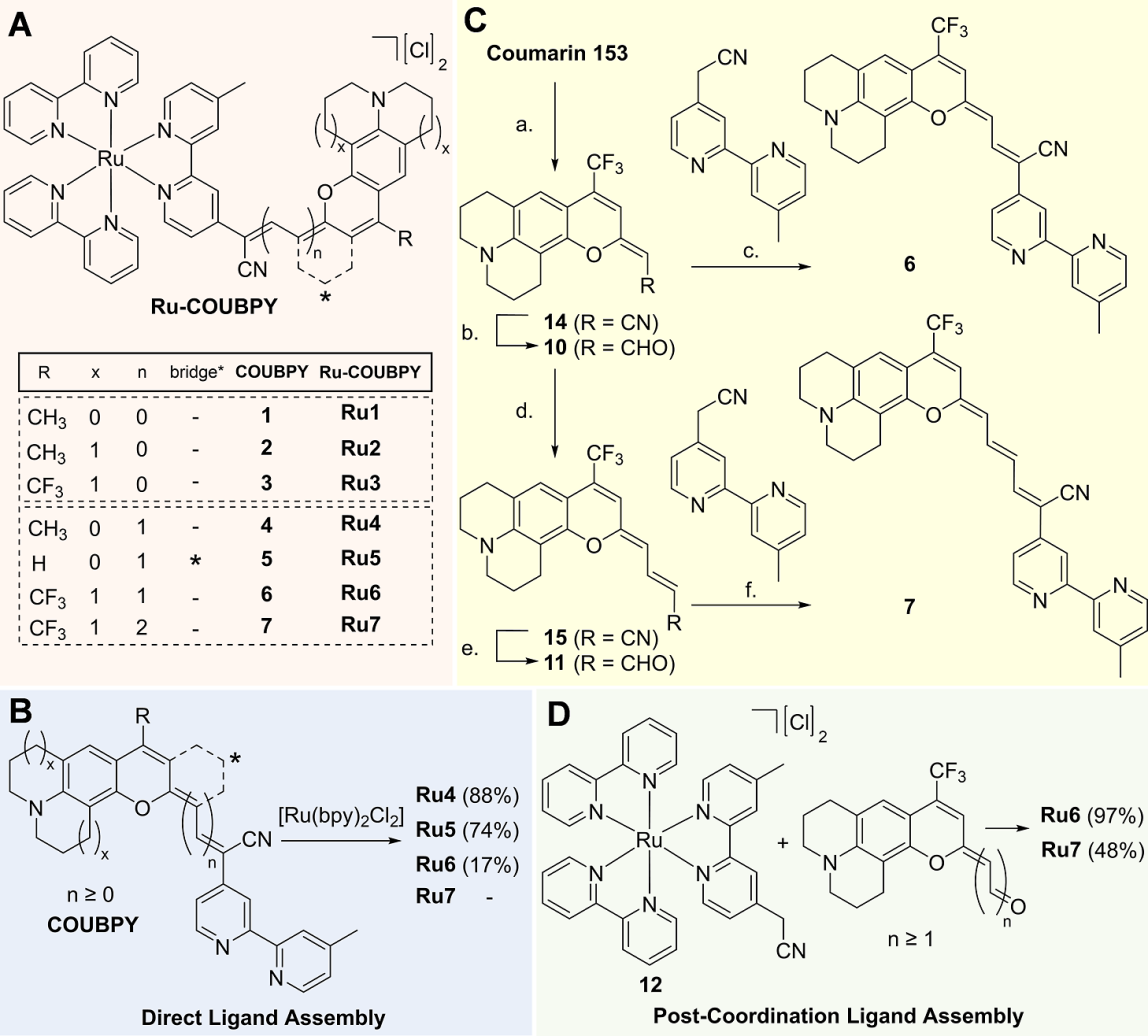
Chemical structures of Ru–COUBPY complexes and schematic representation of the two synthetic strategies used in this work to synthesize π-extended Ru–COUBPY PSs. (A) Chemical structures of the parent Ru–COUBPY complexes **Ru1–3** and their π-extended derivatives **Ru4–7**. (B) Coordination of the preassembled COUBPY ligands **4–7** for the synthesis of **Ru4–7**. (C) Synthetic route for the preparation of COUBPY ligands **6**–**7**. Reagents and conditions: (a) (1) CH_3_CN, *n*-BuLi, THF, Ar atm, −78 °C, 30 min and (2) HCl aq, rt, 3 h, and 70%; (b) (1) DIBAL-H, toluene, rt, and 30 min and (2) potassium sodium tartrate, rt, 30 min, and 50%; (c) piperidine, EtOH, 80 °C, 16 h, and 83%; (d) Ph_3_PCHCN, toluene, 60 °C, 7 days, and 84%; (e) (1) DIBAL-H, toluene, rt, and 30 min and (2) potassium sodium tartrate, rt, 30 min, and 69%; and (f) piperidine, EtOH, 80 °C, 16 h, and 89%. (D) Postcoordination ligand assembly strategy for synthesizing **Ru6** and **Ru7**.

Building on these antecedents, herein, we present a series of π-extended Ru–COUBPY complexes (**Ru4–7**, Figure 1) as one-photon NIR-activated PSs. As shown in Figure 1A, the exocyclic double bond of the original coumarin scaffold was strategically vinylogated to enable absorption in the NIR region. Specifically, vinylogation of parent compound **Ru1** produced derivatives **Ru4** and **Ru5**, while vinylogation of **Ru3** yielded derivatives **Ru6** and **Ru7**. In derivative **Ru5**, a more rigid 2,3-dihydro-1H-xanthene scaffold was introduced to investigate the effect of conformational constraint on photostability, whereas **Ru7** was synthesized to evaluate the impact of incorporating an additional vinyl unit into the polymethine chain with respect to **Ru6**. These π-extended Ru–COUBPY PSs demonstrated potent *in vitro* phototoxicity against CT-26 cells upon NIR irradiation at 740 and 770 nm, both under normoxic and hypoxic conditions, representing a clear improvement over the parent Ru–COUBPY complexes **Ru1** and **Ru3**. Remarkably, **Ru6**, the lead compound in this series, exhibited strong *in vivo* tumor growth inhibition in mice bearing subcutaneous colorectal tumors upon irradiation with highly penetrating one-photon NIR light at 780 nm. This positions **Ru6** among the first Ru­(II) polypyridyl complexes to demonstrate *in vivo* PDT antitumor efficacy under one-photon 780 nm NIR activation, underscoring its strong potential as a next-generation PS for the effective treatment of deep-seated hypoxic solid tumors.

## Results and Discussion

### Design, Synthesis, and Chemical Characterization of π-Extended Ru–COUBPY Complexes

π-Extended Ru–COUBPY complexes **Ru4–7** were successfully synthesized using two alternative synthetic approaches, as outlined in Figure 1. Initially, we attempted the coordination of π-extended COUBPY ligands **4–7** to [Ru­(bpy)_2_Cl_2_] following our previously reported methodology for parental Ru–COUBPY complexes **Ru1–3** ^40^ (Figure 1B). Ligands **4–7** were directly assembled *via* a Knoevenagel condensation of the corresponding coumarin aldehydes (**8**–**11**) with 2-(4′-methyl-[2,2′-bipyridin]-4-yl)­acetonitrile, as exemplified for COUBPY ligands **6** and **7** in Figure 1C and further detailed in the Supporting Information (Schemes S1 and S3). Using this synthetic method, **Ru4** and **Ru5** were obtained in high yields (88% and 74%, respectively). In contrast, **Ru6** was obtained with a much lower yield (17%), and no product was detected for **Ru7**.

These results prompted us to explore an alternative synthetic approach based on a novel postcoordination ligand assembly strategy involving the Ru­(II) complex intermediate **12**, which was synthesized by coordinating 2,2′-bipyridyl acetonitrile to [Ru­(bpy)_2_Cl_2_] (Figure 1D). To our delight, the piperidine-mediated Knoevenagel condensation of this key heteroleptic Ru­(II) complex with coumarin aldehyde derivatives **10** and **11** successfully afforded the desired complexes **Ru6** and **Ru7** in moderate to excellent yields (97% and 48%, respectively). This strategy expands the synthetic versatility of Ru­(II) polypyridyl complexes and opens new avenues for the design and synthesis of metal complexes with tunable photophysical, photochemical, and photobiological properties. All Ru–COUBPY complexes, along with their corresponding COUBPY ligands, were fully characterized by 1D ^1^H and ^13^C­{^1^H}-NMR, 2D ^1^H,^1^H NOESY NMR, and HRMS. The purity of the products was confirmed by reversed-phase HPLC-MS analysis (Figures S9 and S10). As expected, according to their log *P*_O/W_ values (Table S1 and Figure S11), **Ru4–7** complexes were more lipophilic than the parent Ru–COUBPY complexes^40^ which indicates that vinylogation results in an increase of lipophilicity.

The (*E/Z*) stereochemistry of the olefinic bonds within the polymethine chains of COUBPY ligands **4–7** and Ru–COUBPY complexes **Ru4–7** was unequivocally assigned through 2D-NOESY experiments (Figures S1–S8). COUBPY ligands **4–6** (*n* = 1, Figure 1) were assigned a *Z,Z* stereochemistry, while COUBPY ligand **7** (with *n* = 2, Figure 1) displayed a *Z,E,Z* configuration. Similarly, a single stereoisomer was identified for each Ru–COUBPY complex, which retained the configuration of the corresponding free COUBPY ligand. Nonetheless, closer examination of the ^1^H NMR spectra of **Ru4** and **Ru6** revealed the presence of a minor set of proton signals. In the case of **Ru4**, the presence of characteristic exchange cross-peaks in the 2D NOESY spectrum (Figure S5) indicated that the additional set of signals could be attributed to a rotameric equilibrium. In contrast, the 2D NOESY spectrum of **Ru6** showed no exchange cross-peaks. However, the presence of two distinct peaks in the HPLC chromatogram after purification, both exhibiting identical UV–Vis and ESI-MS spectra, suggested once more the existence of two interconverting isomers. Indeed, this hypothesis was validated by individually collecting each peak and reinjecting them in HPLC, revealing that the proportion of the two species returned to equilibrium (Figure S10).

### Photophysical Characterization of π-Extended Ru–COUBPY Complexes: Experimental and Computational Studies

The photophysical properties of the new π-extended Ru–COUBPY complexes were experimentally measured in acetonitrile (ACN) at room temperature. As shown in Figure 2A, the absorption spectra of **Ru4–7** differ significantly from those of their parent Ru–COUBPY complexes **Ru1** and **Ru3**,^40^ exhibiting stronger red-shifted absorption within the deep-red and the NIR region. Among the three Ru–COUBPY complexes containing a single extra vinyl group, **Ru5** and **Ru6** displayed broad absorption bands centered at around 630 nm (Table 1), extending beyond 750 nm. Notably, Ru–COUBPY complex **Ru7** with the longer polymethine chain also showed absorption beyond 850 nm. Furthermore, the vinylogation strategy greatly enhanced absorptivity, yielding significantly higher molar extinction coefficients than those of the parent compounds. (*e.g.*, 35.2 mM cm^–1^ at λ_max_ = 629 nm for **Ru6** *vs* 20 mM cm^–1^ at λ_max_ = 571 nm for **Ru3** ^40^).

**Figure 2:**
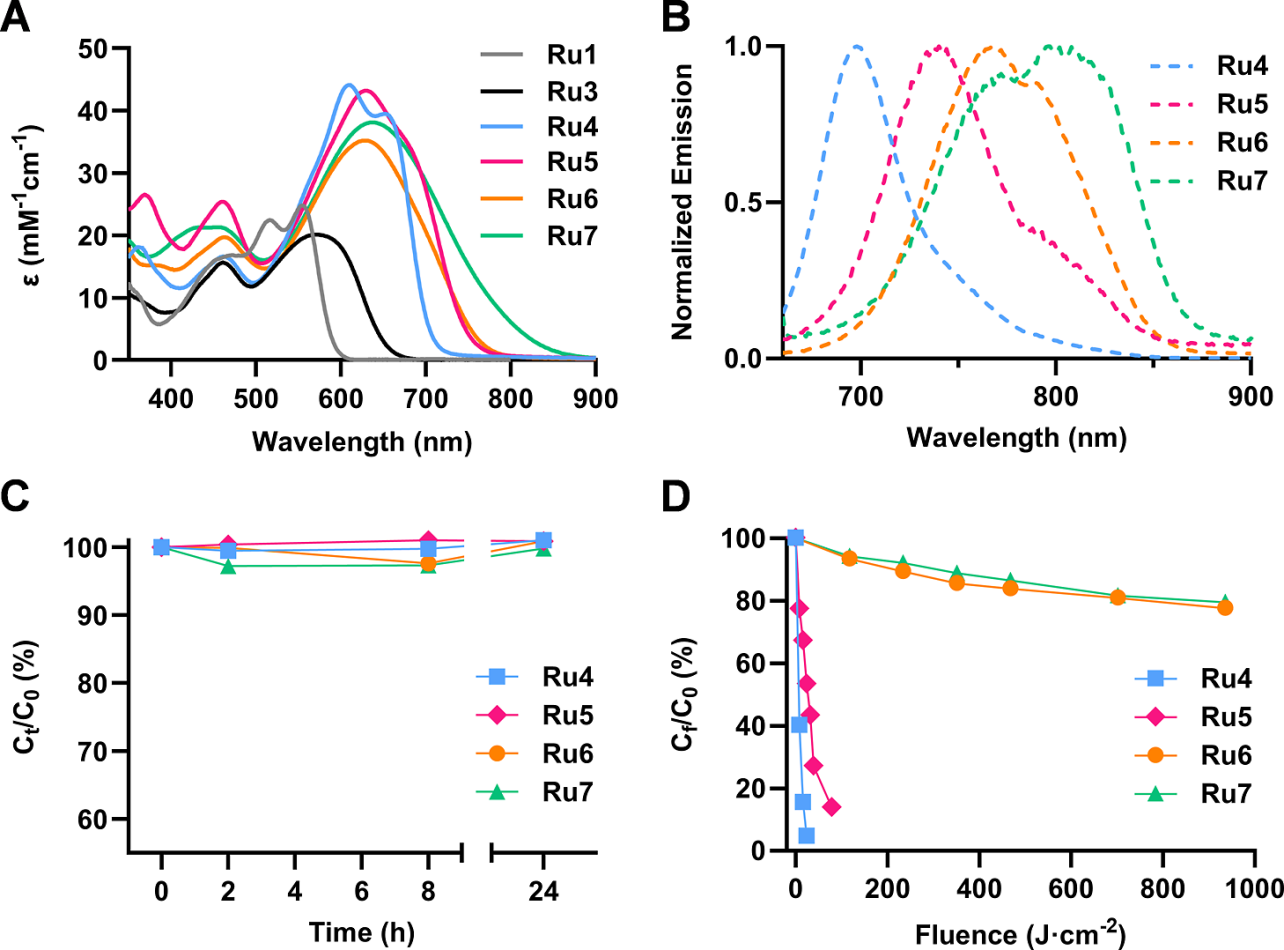
Photophysical characterization and dark/light stability of Ru–COUBPY complexes. (A) Absorption spectra of the parent Ru–COUBPY complexes and their π-extended derivatives in ACN. (B) Emission spectra (λ_ex_ = 650 nm) of π-extended Ru–COUBPY complexes in ACN. C) Dark stability of **Ru4–7** in cell culture medium (DMEM, 10% FBS) at 37 °C. (D) Photostability of π-extended Ru–COUBPY complexes in DMEM, 10% FBS at 37 °C after red-light irradiation (620 ± 15 nm; 130 mW cm^–2^). *C*_0_ represents the concentration of the compound at the beginning of the experiments (*t* = 0); *C*_t_ and *C*_f_ represent the concentration at various time points and fluence doses throughout the experiments, respectively.

**Table 1:** Photophysical Properties and Singlet Oxygen Quantum Yields of Ru–COUBPY Complexes in ACN at Room Temperature^a^

	spectroscopic properties					singlet oxygen quantum yield
	λ_abs_/nm (ε/mM^–1^cm^–1^)	λ_em_/nm (650 nm)	λ_em_/nm (720 nm)	τ/ns (405 nm)	τ/ns (635 nm)	Φ_Δ_ (620 nm)
**Ru4**	290 (29.7), 363 (18.1), 462 (16.6), 609 (44.1), 653 (39.4)	698	-	4.2	0.4	0.30
				0.4		
**Ru5**	291 (20.8), 369 (26.6), 462 (25.4), 630 (43.2)	740	739	4.5	0.3	0.06
				0.3		
**Ru6**	290 (28.6), 462 (19.9), 629 (35.2)	768	767	6.4	0.3	0.10
				0.3		
**Ru7**	291 (27.0), 455 (21.3), 637 (38.1)	797	815	5.9	0.3	0.04
				0.3		

a Absorption maxima wavelengths (λ_abs_), molar absorption coefficients (ε) at λ_abs_, emission maxima wavelengths (λ_em_) at the indicated λ_exc_, emission lifetimes (τ) in deoxygenated ACN upon excitation at the indicated λ_exc_, and singlet oxygen quantum yield (Φ_Δ_) determined upon excitation at the indicated wavelength.

The emission spectra of the π-extended Ru–COUBPY complexes were registered in air-saturated ACN using three different excitation wavelengths (Figures 2B and S14). Upon excitation within the COUBPY ligand absorption band (λ_exc_ = 650 or 720 nm), all Ru–COUBPY complexes exhibited strong emission within the NIR region, with **Ru5–7** exhibiting emission between 739 to 815 nm when excited at 720 nm. In all cases, the fluorescence quantum yield was very low (Table S2) and short emission lifetimes were obtained (0.3–0.4 ns) upon excitation at 635 nm in deoxygenated ACN solutions under an inert atmosphere (Table 1 and Figure S15). These measurements correlate very well with efficient intersystem crossing to the triplet state, which is the PDT active state. A second slightly longer emission lifetime was observed upon excitation at 405 nm (4.2–6.4 ns), which can also be assigned to the fluorescence of the coumarin moiety (Table 1 and Figures S16 and S17). Despite being part of a π-conjugated ligand system, the coumarin unit retains its emissive character, as supported by previous studies.^40^

Overall, these results confirmed our hypothesis that extending the π-conjugation within the COUBPY ligands through vinylogation of the exocyclic double bond of the coumarin scaffold produces Ru–COUBPY complexes with enhanced red-shifted absorption and emission relative to the parent compounds, offering a promising strategy for the design of one-photon NIR-activated PSs.

The absorption properties of π-extended Ru–COUBPY complexes were also investigated from a computational perspective.^46–48^ The main electronic transitions are summarized in Table S3. The data confirm a bathochromic shift in the **Ru4–7** series compared to the previously studied parent compounds **Ru1–3** ^40^ (see Figure S18). Although the computed wavelengths are slightly blue-shifted relative to the experimental wavelengths, the calculations reveal a notable change in the nature of the lowest energy absorption bands. Specifically, the natural transition orbitals (NTOs)^49^ involved in the bright *S*_1_ states of the π-extended Ru–COUBPY complexes (Figure S19) clearly indicate a strong intraligand charge transfer (ILCT) character. This contrasts with the predominantly MLCT nature observed in the lowest-energy absorption bands of the parent Ru–COUBPY complexes **Ru1** and **Ru3**,^40^ as also shown in Figure S20. The vinylogous extension involving the exocyclic double bond of the coumarin core leads to the localization of both the HOMO and the LUMO on the COUBPY ligand, as illustrated in Figure S21 and detailed in Table S4. In the case of **Ru4**, an additional absorption band was identified at wavelengths above 600 nm alongside the ILCT band, the nature of which was ascribed to a Ru → bpy transition.

The metal-centered HOMO-1 orbital predominantly contributed to the transitions computed at 460 nm, consistent with the experimental broad shoulders observed for all Ru–COUBPY complexes (**Ru1** to **Ru7**) in this spectral region. The computations confirmed that these transitions are primarily of the Ru → ligand character. For instance, two singlet–singlet transitions at 489 and 457 nm with similar intensity, both of the Ru → bpy character, contribute to the experimental band located at 462 nm for **Ru4,** (see Figure S22). Similarly, the experimental band at 462 nm for **Ru5** is attributable to two transitions at 459 and 435 nm, also of Ru → bpy character (Figure S23). For **Ru6** and **Ru7** (Figures S24 and S25, respectively), the experimental bands peaking at 462 and 455 nm, respectively, can be explained by a combination of Ru → COUBPY and Ru → bpy excitations. The Ru→COUBPY transitions are calculated at 455 nm for **Ru6** and 469 nm for **Ru7**, while the Ru → bpy transitions are computed at 452 and 456 nm, respectively. At shorter wavelengths, the transition computed at 370 nm in **Ru4**, of a Ru → bpy nature, can be attributed to the experimental band at 363 nm. For **Ru5**, the band observed at 372 nm corresponds to a mixed ILCT/MLCT state calculated at 341 nm (see Table S3).

### Dark and Light Stability of π-Extended Ru–COUBPY Complexes Ru4**–**7 in Cell Culture Medium

The stability of Ru–COUBPY complexes was investigated in complete cell culture medium (DMEM supplemented with 10% FBS), both in the dark and under visible light irradiation (Figures S26–S33). According to HPLC-MS analysis, all compounds remained completely stable after 24 h of incubation at 37 °C in the dark (Figure 2C). After irradiation with red light (620 ± 15 nm; 130 mW cm^–2^), **Ru4** and **Ru5** proved to be much less photostable than their parent compound **Ru1** (Figure 2D).^40^ Specifically, **Ru4** was completely degraded after receiving an accumulated light dose of 25 J·cm^–2^, whereas **Ru5** required a higher light dose (125 J·cm^–2^) to undergo complete degradation. These findings suggest that the conformational restriction around the exocyclic double bond can compensate for the reduction in photostability associated with vinylogation compared to that of the parent **Ru1** complex. Conversely, **Ru6** and **Ru7** demonstrated exceptional high photostability, with **Ru7** being particularly photostable despite its longer polymethine chain. This contrasts the typically reduced photostability observed in polymethine cyanine dyes with comparable number of conjugated double bonds.^50–52^ Specifically, both **Ru6** and **Ru7** retained about 80% of their integrity even after exposure to a light dose of 900 J·cm^–2^, a value far exceeding typical fluences used in photobiological studies, including *in vivo* testing (*vide infra*). This is consistent with the results obtained for the parent compound **Ru3** and confirms that the incorporation of a strong electron-withdrawing CF_3_ group at the 4-position of the coumarin scaffold, either in COUPY dyes or COUBPY ligands, increases the photostability of the resulting compounds.^53^

### Photochemical Characterization: Experimental and Computational Studies

The ability of π-extended Ru–COUBPY complexes **Ru4–7** to photogenerate Type I and Type II ROS was investigated by using a combination of spectroscopic techniques. As shown in Figure 3A, ^1^O_2_ production was initially confirmed with the fluorogenic probe Singlet Oxygen Sensor Green (SOSG). A significant increase in probe emission was observed during red light irradiation in the presence of **Ru5** and **Ru6**. In contrast, the emission increase mediated by **Ru4** was rapidly quenched, likely due to its limited photostability. Meanwhile, **Ru7** demonstrated a reduced capacity to sensitize ^1^O_2_ compared to that of the other compounds in the series. As expected, the increase in SOSG fluorescence resulting from ^1^O_2_ photogeneration was completely suppressed in the presence of sodium azide, a specific ^1^O_2_ scavenger (Figure S35). Furthermore, singlet oxygen quantum yields (Φ_Δ_) were determined by using 1,3-diphenylisobenzofuran (DPBF) as a ^1^O_2_ scavenger and methylene blue as the standard (Figures S36 and S37), confirming the results obtained with SOSG (Table 1).

**Figure 3:**
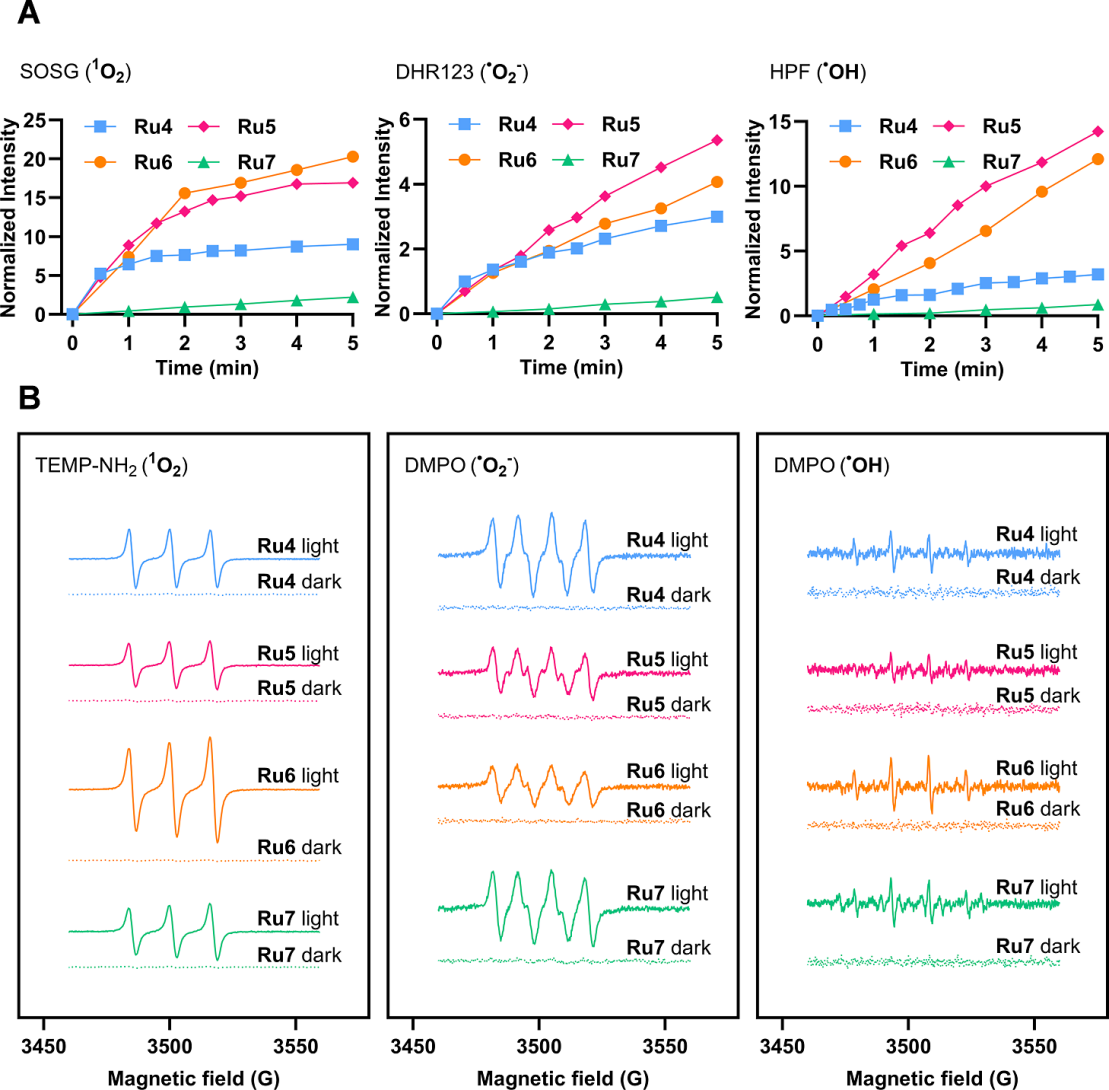
Photogeneration of ROS by π-extended Ru–COUBPY complexes studied using specific fluorogenic probes and EPR spectroscopy. (A) Increase in fluorescence emission of SOSG (5 μM), HPF (5 μM), and DHR123 (10 μM) upon irradiation of Ru–COUBPY complexes (10 μM) in PBS with red light (620 ± 15 nm, 130 mW/cm^2^). (B) EPR spectra of Ru–COUBPY complexes trapped by 4-amino-TEMP in MeOH (left), DMPO in MeOH (center), and DMPO in PBS (right), measured in the dark and after white-light irradiation (50 s for ^1^O_2_, 120 s for ^•^O_2_^–^, and 50–120 s for ^•^OH; 430–1000 nm).

Computational analysis of the lowest triplet state T_1_ reveals an energy decrease trend from **Ru4** (1.66 eV) to **Ru7** (1.22 eV). In all cases, however, the energy remains above the threshold required to generate singlet oxygen (>0.98 eV).^54^ Triplet NTOs reported in Figure S38 clearly show that both donor and acceptor orbitals are localized over the COUBPY ligands allowing to characterize them as ^3^ILCT, analogously to the previous investigated **Ru3** compound.^40^

Similarly, the π-extended Ru–COUBPY complexes were confirmed to photogenerate Type I ROS, under red light irradiation, using dihydrorhodamine 123 (DHR123) and hydroxyphenyl fluorescein (HPF) as specific fluorogenic probes for ^•^O_2_^–^ and ^•^OH radicals, respectively (Figure 3A). Once again, the use of specific scavengers (*e.g.*, tiron for ^•^O_2_^–^ and terephthalic acid for ^•^OH) further confirmed the generation of Type I ROS (Figures S40 and S42). Notably, the generation capability of Type I ROS by **Ru4–7** followed the same trend observed for ^1^O_2_.

Electron paramagnetic resonance (EPR) provided direct evidence for the light-induced generation of Type I and Type II ROS by π-extended Ru–COUBPY complexes. In these studies, 4-amino-2,2,6,6-tetramethylpiperidine (TEMP-NH_2_) was employed as a spin trap to detect ^1^O_2_ under both red-light and white-light irradiation. 5,5-Dimethyl-1-pyrroline-*N*-oxide (DMPO) was also used to trap photogenerated ^•^O_2_^–^ and ^•^OH radicals in methanol and aqueous solutions, respectively. As illustrated in Figure 3B, the photogeneration of ^1^O_2_ by **Ru4–7** complexes upon white-light irradiation was confirmed by the detection of the characteristic EPR triplet signal (peak integral ratio of 1:1:1), corresponding to the TEMPO adduct. Interestingly, a clear correlation was found between the EPR signal intensities and the singlet oxygen photogeneration efficiency determined with SOSG (*i.e.*, compare Figures 3A and S43) under similar red-light irradiation conditions (5 min, 620 ± 15 nm, 130 mW/cm). Similarly, as shown in Figure 3B, the appearance of the diagnostic signals for the DMPO-^•^O_2_^–^ (peak integral ratio of 1:1:1:1) and DMPO-^•^OH (peak integral ratio of 1:2:2:1) adducts further verified the production of ^•^O_2_^–^ and ^•^OH. Importantly, no paramagnetic signals were observed in the absence of light, indicating that Type I and Type II ROS production is exclusively a light-driven process.

The feasibility of Type I reactions was also ascertained computationally through the analysis of the vertical electron affinity and ionization potentials of Ru–COUBPY complexes and molecular dioxygen. The thermodynamic data for the characteristic electron transfer reactions involved in Type I PDT are summarized in Table 2, where Ru–COUBPY complexes are denoted as PS despite the +2 total molecular charge to facilitate the reading of the photoreactions. Results indicate that the autoionization process could take place in all cases *via* reaction 2, which involves the reduction of the complexes in their triplet states. This net electron transfer is strongly exothermic, with Δ*E* < −1 eV for complex **Ru4**, and becomes progressively less exothermic along the series, reaching its minimum value for **Ru7**.

**Table 2:** Thermodynamics [Δ*E* = *E*(products) – *E*(reactants), in eV] of the Type I PDT Reactions in Water for π-Extended Ru–COUBPY Complexes Based on the VEA and VIP Values Shown in Table S5

	type I photoreactions	**Ru4**	**Ru5**	**Ru6**	**Ru7**
	**Autoionization Reactions**				
(1)	^3^PS + ^1^PS → ^•^PS^+^ + ^•^PS^–^	0.47	0.50	0.49	0.43
(2)	^3^PS + ^3^PS → ^•^PS^+^ + ^•^PS^–^	–1.16	–0.97	–0.90	–0.79
	**Indirect Reaction**				
(3)	^•^PS^–^ + ^3^O_2_ → ^1^PS + ^•^O_2_^–^	–0.35	–0.34	–0.10	–0.02
	**Direct Electron Transfer**				
(4)	^1^PS + ^3^O_2_ → ^•^PS^+^ + ^•^O_2_^–^	1.75	2.13	2.28	2.12
(5)	^3^PS + ^3^O_2_ → ^•^PS^+^ + ^•^O_2_^–^	0.12	0.16	0.39	0.41

Following autoionization (reaction 2), the reduced Ru–COUBPY complexes (^•^PS^–^) can transfer an electron to molecular oxygen *via* reaction 3. This process shows a decreasing exothermic trend from **Ru4** to **Ru7**, indicating that the ability of the reduced forms of the Ru complexes to promote superoxide formation varies, with **Ru7** being the least efficient in promoting this process—consistent with experimental observations. In contrast, direct electron transfer through reactions 4 and 5 is predicted to be thermodynamically unfeasible in both the ground and triplet states due to their high endothermicity. This conclusion is supported by cyclic voltammetry (CV) measurements performed on **Ru6** and **Ru7** (Figure S44), which were selected for this comparative analysis. These experiments provided the oxidation and reduction potentials of +0.29/–1.21 V for **Ru6** and +0.23/–1.21 V for **Ru7** (*vs* Fc^+^/Fc; Fc = ferrocene) (Table S7), offering the necessary experimental data to complement the theoretical analysis.

Since the lowest-lying triplet states of the complexes, responsible for ROS generation, are nonemissive due to their strong intraligand (^3^IL) character (Figure S38), experimental phosphorescence data are not available. Therefore, the triplet excited-state energy (Δ*E*_T1–S0_) must be estimated computationally (Table S7). Combining these theoretical values with the CV-derived redox potentials is a well-established method described in the literature for determining excited-state redox properties.^55,56^ The resulting excited-state redox potentials, calculated from the combination of experimental and theoretical data, are summarized in Figures S45 and S46. They confirm that the direct Type I photoreduction pathway is thermodynamically unfavorable for both complexes **Ru6** and **Ru7**, as the processes are endothermic. This finding aligns with theoretical predictions (Table 2) and is further supported by comparison with the O_2_/^•^O_2_^–^ redox couple (−1.235 V *vs* Fc^+^/Fc in acetonitrile).^57^ Given that the excited-state oxidation potentials of **Ru6** (−0.95 V) and **Ru7** (−0.85 V) are less negative than −1.235 V, the direct reduction of molecular oxygen to the superoxide radical is energetically unfeasible. To reinforce this conclusion, the Type I photoreactions in acetonitrile were recalculated for the **Ru6** complex (Table S6), confirming that reaction 5 is thermodynamically unfavorable. Overall, these results demonstrate that direct electron transfer from the complexes to molecular oxygen is not viable in either the singlet or triplet states, in both water and acetonitrile.

### Cellular Uptake Studies by Confocal Microscopy

The cellular uptake of π-extended Ru–COUBPY complexes **Ru4–7** was evaluated in HeLa cells by confocal microscopy, leveraging their intrinsic luminescent properties. Following a 30 min incubation at 10 μM, confocal images confirmed that all compounds were readily internalized (Figure 4). A diffuse staining pattern was observed for all complexes, with filamentous structures suggesting preferential accumulation in the mitochondria, along with some localization in intracellular vesicles. As shown in Figures 4 and S47 and in supplementary videos, the rapid onset of membrane blebbing and mitochondrial disintegration within 2 min of exposure to the confocal microscope’s laser lines underscored the high phototoxicity of these complexes (*vide infra*).

**Figure 4:**
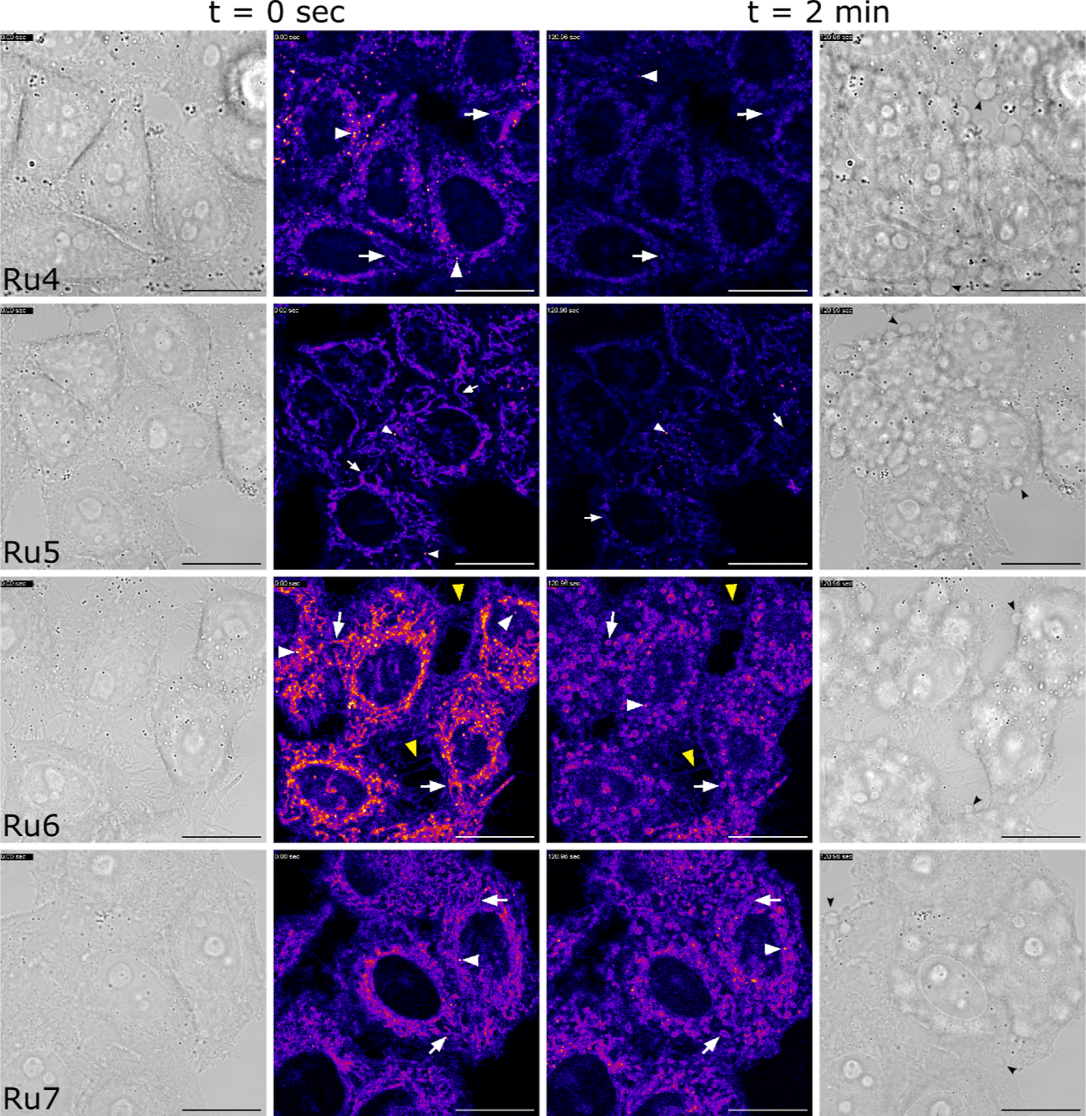
Cellular uptake of π-extended Ru–COUBPY complexes **Ru4–7**. Single confocal planes of HeLa cells incubated with the compounds (10 μM) for 30 min at 37 °C, imaged at *t* = 0 (left) and after 2 min (right) of first observation. Excitation was performed with the 561 nm laser line. White arrows point out mitochondria, white arrowheads point out vesicle staining, and black arrowheads point out cell blebbings. Yellow arrowheads for **Ru6** point out filopodia of the extracellular membrane of the cells. Scale bar: 20 μm. Fluorescence intensities are color coded using the “fire” look-up table.

The subcellular localization of **Ru4–7** was studied through colocalization experiments using mitochondria-specific (MitoTracker Green, MTG) and lysosome-specific (LysoTracker Green, LTG) fluorescent markers. As depicted in Figures S48–S49 and Table S8, **Ru6** exhibited a strong correlation with MTG (Pearson’s correlation coefficient, PCC = 0.76) and a much weaker correlation with LTG (PCC = 0.47), indicating predominant accumulation in the mitochondria. **Ru7** displayed a similar pattern, with a high PCC of 0.74 with MTG and a lower PCC of 0.44 with LTG. **Ru5** showed the strongest mitochondrial localization among the series, with the highest PCC with MTG (PCC = 0.78) and a markedly lower correlation with LTG (PCC = 0.28), confirming its greater specificity for mitochondria with minimal lysosomal accumulation. In contrast, **Ru4** displayed a more even distribution with moderate correlations with both MTG (PCC = 0.61) and LTG (PCC = 0.57), suggesting a mixed localization between mitochondria and lysosomes.

### *In Vitro* (Photo)­cytotoxicity Evaluation of π-Extended Ru–COUBPY Complexes toward CT-26 Colorectal Cancer Cells

The *in vitro* (photo)­cytotoxicity of **Ru4–7** was evaluated in CT-26 colorectal cancer cells under both normoxic (21% O_2_) and hypoxic conditions (2% O_2_) (Tables 3 and 4). Cells were incubated for 4 h with increasing concentrations of the compounds, followed by medium refreshment and exposure to deep-red (670 nm) or NIR (740 or 770 nm) light for 1 h or kept in the dark. After 48 h, cell viability was assessed using the resazurin assay, and IC_50_ values were determined from the corresponding dose–response curves (Figure S50). Phototherapeutic indices (PI), defined as the ratio [IC_50_]­dark/[IC_50_]­light, were also calculated as a measure of phototherapeutic efficacy.

**Table 3:** (Photo)­cytotoxicity of π-Extended Ru–COUBPY Complexes **Ru4–7**, PpIX, and Redaporfin toward CT-26 Cancer Cells Expressed as IC_50_ Values (μM) under Normoxia (21% O_2_)­^a^

	dark	670 nm		740 nm		770 nm	
	IC_50_	IC_50_	PI^b^	IC_50_	PI^b^	IC_50_	PI^b^
**Ru1**	>250	1.460 ± 0.450	>171	31.3 ± 6.1	>8	>100	-
**Ru3**	>250	0.036 ± 0.003	>6944	0.76 ± 0.06	>329	24.86 ± 1.96	>10
**Ru4**	>100	0.121 ± 0.019	>828	0.441 ± 0.054	>227	2.558 ± 0.613	>39
**Ru5**	>100	0.793 ± 0.103	>126	1.508 ± 0.443	>66	2.578 ± 0.504	>39
**Ru6**	>100	0.095 ± 0.004	>1048	0.177 ± 0.007	>564	0.798 ± 0.031	>125
**Ru7**	>100	1.083 ± 0.012	>92	7.949 ± 1.773	>13	13.2 ± 1.97	>8
**PpIX**	>100	1.062 ± 0.101	>94	-	-	-	-
**Redaporfin**	>100	-	-	0.421 ± 0.09	>238	0.874 ± 0.033	>114

a Experimental conditions: Cells were treated for 4 h at 37 °C, followed by either 1 h in the dark or irradiation under the specified light conditions. Cell viability was determined after 44 h using the resazurin assay. Irradiation parameters: 670 nm (3.75 mW cm^–2^, 13.5 J cm^–2^), 740 nm (3.50 mW cm^–2^, 12.6 J cm^–2^), and 770 nm (6.75 mW cm^–2^, 24.3 J cm^–2^).

b PI = phototherapeutic index defined as [IC_50_]­dark/[IC_50_]­light.

**Table 4:** (Photo)­cytotoxicity of π-Extended Ru–COUBPY Complexes **Ru4–7** toward CT-26 Cancer Cells Expressed as IC_50_ Values (μM) under Hypoxia (2% O_2_)­^a^

	dark	670 nm			740 nm		
	IC_50_	IC_50_	PI^b^	HI^c^	IC_50_	PI^b^	HI^c^
**Ru4**	>100	0.208 ± 0.006	>481	1.72	3.72 ± 0.958	>27	1.19
**Ru5**	>100	1.043 ± 0.062	>96	1.32	3.215 ± 0.873	>31	1.25
**Ru6**	>100	0.377 ± 0.061	>265	3.97	0.597 ± 0.216	>168	3.37
**Ru7**	>100	8.917 ± 0.474	>11	8.23	25.140 ± 1.451	>4	3.16

a Experimental conditions and irradiation parameters: see legend to Table 3 and Supporting Information.

b Phototherapeutic index (PI) = [IC_50_]­dark/[IC_50_]­light.

c Hypoxia index (HI) = [IC_50_] light­(hypoxia)/[IC_50_]­light­(normoxia).

Table 3 summarizes the IC_50_ values for the new π-extended Ru–COUBPY complexes **Ru4–7**, alongside those for the two parent compounds **Ru1** and **Ru3**, enabling the analysis of structure–activity relationships. Protoporphyrin IX (PpIX) and Redaporfin, both clinically relevant PSs, were included as benchmark compounds in this study for comparison under red and NIR light activation, respectively.^55^ Notably, none of the newly synthesized Ru–COUBPY complexes exhibited dark toxicity at concentrations up to 100 μM, mirroring the behavior of the parent complexes **Ru1** and **Ru3**.^40^ More importantly, complexes **Ru4–6** showed potent phototoxicity under deep-red irradiation, as well as upon light irradiation within the NIR region (Figure 5A and S51).

**Figure 5:**
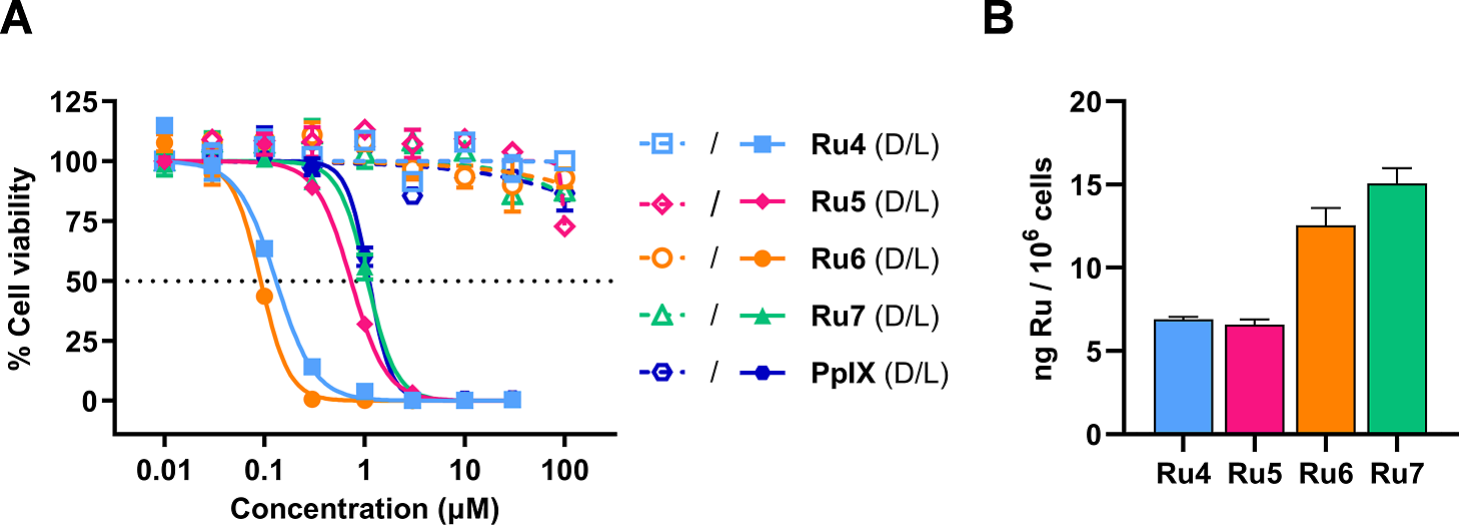
*In vitro* evaluation of π-extended Ru–COUBPY complexes in CT-26 cells. (A) Dose–response curves for complexes **Ru4–7** and PpIX following 4 h incubation under normoxic conditions, either in the dark (open symbols) or upon deep-red light irradiation (670 nm, 13.5 J cm^–2^; filled symbols). (B) Intracellular accumulation of complexes **Ru4–7** in CT-26 cells after 4 h treatment at 5 μM, quantified by ICP-MS.

A detailed comparison of the photobiological data for parent Ru–COUBPY complex **Ru1** and its π-extended derivatives **Ru4** and **Ru5** (Table 3) reveals that the vinylogation strategy significantly enhances photoactivity under both deep-red and NIR illumination. While **Ru1** had previously demonstrated outstanding phototoxicity upon irradiation at 540 and 645 nm,^40^ it was found almost nonphototoxic under NIR light (*e.g.*, IC_50_ = 31.3 μM at 740 nm). In contrast, **Ru4** demonstrated superior performance across all tested wavelengths, achieving nanomolar phototoxicity at 670 nm (IC_50_ = 121 nM, PI > 828) and at 740 nm (IC_50_ = 441 nM, PI > 227). Similarly, **Ru5** exhibited submicromolar activity at 670 nm (IC_50_ = 793 nM, PI > 126) and maintained good activity under NIR light irradiation, with IC_50_ values of 1.51 μM (PI > 66) and 2.58 μM (PI > 39) upon irradiation at 740 and 770 nm, respectively. These results, however, indicate that the improved photostability of **Ru5** did not translate into superior phototoxicity relative to that of **Ru4**.

A comparison of the parent **Ru3** complex with its vinylogous derivative **Ru6** further confirmed that the extension of the π-conjugated system leads to improved photoactivity under long-wavelength irradiation. In this sense, **Ru6** exhibited comparable nanomolar phototoxicity to **Ru3** at 670 nm (IC_50_ = 95 nM for **Ru6** *vs* IC_50_ = 36 nM for **Ru3**), while showing markedly superior potency under NIR irradiation, specially at 770 nm (IC_50_ = 0.798 μM for **Ru6** *vs* 24.86 μM for **Ru3**). Unfortunately, the Ru–COUBPY complex with the longer polymethine chain, **Ru7**, was the least phototoxic compound in the series, with IC_50_ values of 7.95 μM (PI > 13) at 740 nm and 13.19 μM (PI > 8) at 770 nm. To exclude the possibility that **Ru7**’s lower photoactivity resulted from reduced cellular uptake, intracellular Ru levels were measured by inductively coupled plasma mass spectrometry (ICP-MS) following a 4 h incubation of CT-26 cancer cells with **Ru4–7** at 5 μM. As shown in Figure 5B, the intracellular Ru content of the 4-CF_3_-substituted derivatives **Ru6** and **Ru7** was slightly higher than that observed for **Ru4** and **Ru5**, indicating that the lower photoactivity of **Ru7** may be primarily attributed to its lower ROS photogeneration capability.

Gratifyingly, complexes **Ru4–6** outperformed PpIX, the active metabolite of 5-ALA, under deep-red light irradiation. Among them, **Ru6** emerged as the most promising candidate for NIR-activated PDT, exhibiting slightly higher activity than Redaporfin (*e.g.*, IC_50_ = 117 nM for **Ru6** *vs* IC_50_ = 421 nM for Redaporfin at 740 nm), also known as LUZ11, which has received approval in the European Union for the treatment of biliary tract cancer and is currently in clinical development for the treatment of cisplatin-resistant head and neck squamous cell carcinoma.^58^

Furthermore, π-extended Ru–COUBPY complexes **Ru4–6** retained high photoactivity under deep-red light irradiation under hypoxic conditions (2% O_2_, Table 4). Notably, **Ru4** and **Ru6** exhibited nanomolar IC_50_ values of 208 nM (PI > 481) and 377 nM (PI > 265), respectively, under deep-red light irradiation. Once again, **Ru6** was the most active compound in the series under NIR light irradiation at 740 nm, with an IC_50_ of 597 nM and a PI > 168. The hypoxia index (HI),^32^ defined as the ratio [IC_50_]­normoxia/[IC_50_]­hypoxia under light conditions, was calculated to illustrate the oxygen-dependence of the phototoxic effects of the Ru–COUBPY complexes. As shown in Table 4, **Ru4** and **Ru5** tolerated low-oxygenation conditions much better than **Ru6**, exhibiting an HI of around 1. Nevertheless, only a 3-fold decrease in phototoxicity was observed for **Ru6** upon changing from normoxic to hypoxic conditions upon NIR irradiation at 740 nm. Overall, the π-extended Ru–COUBPY complexes demonstrated effectiveness under hypoxic conditions similar to that of the parent compounds **Ru1** and **Ru3**, which can be attributed to their ability to generate both Type I and Type II ROS in sensitive organelles such as mitochondria.

To better understand the cell death mechanisms triggered by lead π-extended Ru–COUBPY complex **Ru6** upon deep-red and NIR light irradiation, we performed dual staining with Annexin V-FITC and propidium iodide (AV/PI), using cisplatin as a positive control for apoptotic cell death. As shown in Figure S52, a significant proportion of CT-26 cells displayed phosphatidylserine translocation (AV+/PI– and AV+/PI+ populations) after irradiation with either deep-red (660 nm) or NIR (760 nm) light in the presence of **Ru6** at a concentration equivalent to 2xIC_50_. The apoptotic ratios were ∼6% and ∼22%, respectively, which are comparable to the apoptotic ratio induced by cisplatin (∼8%). This suggests that apoptosis is the primary cell death mechanism involved. Furthermore, considering the **Ru6**’s ability to photogenerate ^•^OH and ^•^O_2_^–^, which can promote lipid peroxidation and thereby induce ferroptosis,^59,60^ we conducted a series of cell viability assays using the ferroptosis inhibitors ferrostatin-1 (Fer-1) and deferoxamine (DFO). As shown in Figure S53 and presented in Table S9, both Fer-1 and DFO slightly rescued the viability of CT-26 cells treated with **Ru6** and irradiated with deep-red or NIR light, suggesting that ferroptosis may also contribute to the observed cell death.

### *In Vivo* Evaluation of the Antitumoral PDT Efficacy of **Ru6** in a Subcutaneous CT-26 Syngeneic Murine Colorectal Tumor Model Using Deep-Red and NIR Light Activation

Based on the overall *in vitro* photobiological data, **Ru6** was selected as the lead PS of the new π-extended Ru–COUBPY series for *in vivo* evaluation due to its potent phototoxicity under both normoxic and hypoxic conditions upon irradiation with deep-red and NIR light. The *in vivo* antitumoral PDT efficacy of **Ru6** was investigated in female BALB/c mice bearing subcutaneous CT-26 syngeneic colon tumors using both deep-red (660 nm) and NIR (780 nm) LED light activation. To this end, three independent *in vivo* studies were designed and sequentially conducted, as detailed in Table S10 and illustrated in Figure 6A. Initially, the efficacy of **Ru6** was assessed under deep-red (study I) and NIR (study II) illumination. Subsequently, study III was designed to enable direct comparison of the PS’s efficacy upon activation with the two light sources in a parallel *in vivo* experiment.

**Figure 6:**
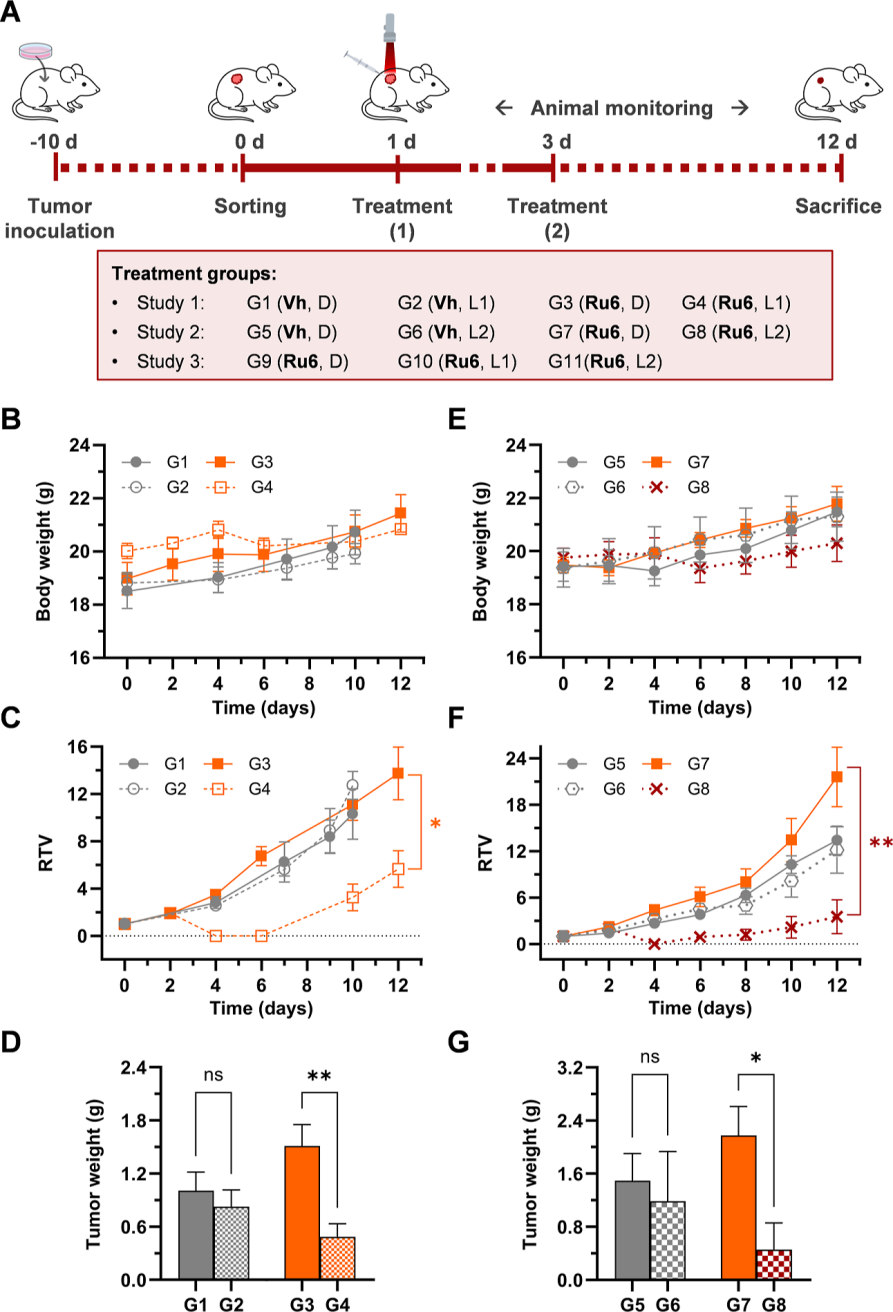
*In vivo* evaluation of **Ru6**’s PDT antitumor efficacy in a subcutaneous CT-26 tumor model in BALB/c mice using deep-red and NIR light. (A) Experimental design: eight week old female BALB/c mice were subcutaneously inoculated with *ca* 1.15 × 10^6^ CT-26 cells on day 10. By day 0, when tumors reached *ca* 50–100 mm^3^, mice were randomly divided into different groups (*n* = 4 or 5/group, see Table S10). On days 1 and 3, each group received the assigned treatment and was either exposed to light irradiation or not (660 or 780 nm, Table S10, 100 mW/cm^2^). On day 12, animals were sacrificed, and tumor samples were collected. (B,E) Body weight (g), (C,F) RTV curves of mice over the 12 day study period, and (D,G) average tumor weights of mice on the day of sacrifice for the studies I (B,C,D) and II (E,F,G). Data are presented as mean ± SEM (*n* = 4 or 5 females in studies II and I, respectively). RTV values on day 12 and average tumor weight data were analyzed using a one-way ANOVA followed by Bonferroni’s multiple comparison test (asterisks: *p < 0.05, **p < 0.01).

As illustrated in Figure 6A, mice were subcutaneously inoculated with *ca* 1.15 × 10^6^ CT-26 cells and once tumors reached the desired size (*ca* 50–100 mm^3^), animals were randomly assigned to various groups, each receiving a specific treatment as summarized in Table S10: study I, groups G1–G4 (*n* = 5); study II, groups G5–G8 (*n* = 4); and study III, groups G9–G11 (*n* = 5). The treatment regime involved administering either vehicle (**Vh**) or **Ru6** (6 mg/kg) on days 1 and 3 *via* intratumoral (IT) injection (40 μL) over 2 min to ensure even distribution within the tumor. For light-treated groups, immediately after injection, tumors were irradiated for 20 min with either deep-red light (660 ± 20 nm, 100 mW/cm^2^) or NIR light (780 ± 15 nm, 100 mW/cm^2^), using two separate LED devices while maintaining a consistent light dose of 120 J/cm^2^. Nonirradiated groups, receiving either the vehicle or **Ru6**, served as controls to evaluate tumor growth in the absence of light exposure. Following the specified treatments, all animals were monitored for clinical signs, changes in body weight, and tumor volumes. Throughout the 12 day study period, no mortality occurred in any group, and all animals showed normal behavior with no signs of stress or discomfort, including those treated with **Ru6**. These results are consistent with the previously reported excellent *in vivo* tolerability of the parent Ru–COUBPY complex **Ru3**.^40^ As shown in Figure 6B,E and 7A, no significant differences in body weight were observed between **Ru6**-treated groups, whether exposed to light or not, and the vehicle groups. However, some animals treated with **Ru6** and light showed a decrease in body weight change (BWC) parameter compared to other groups of the study, which was most evident on day 4. This effect is likely associated with the potent antitumor response induced by the treatment (*vide infra*). Following this temporary weight loss, which never fell below 5% of the initial baseline, the animals rapidly regained weight, ultimately reaching values comparable to those of the other groups.

**Figure 7:**
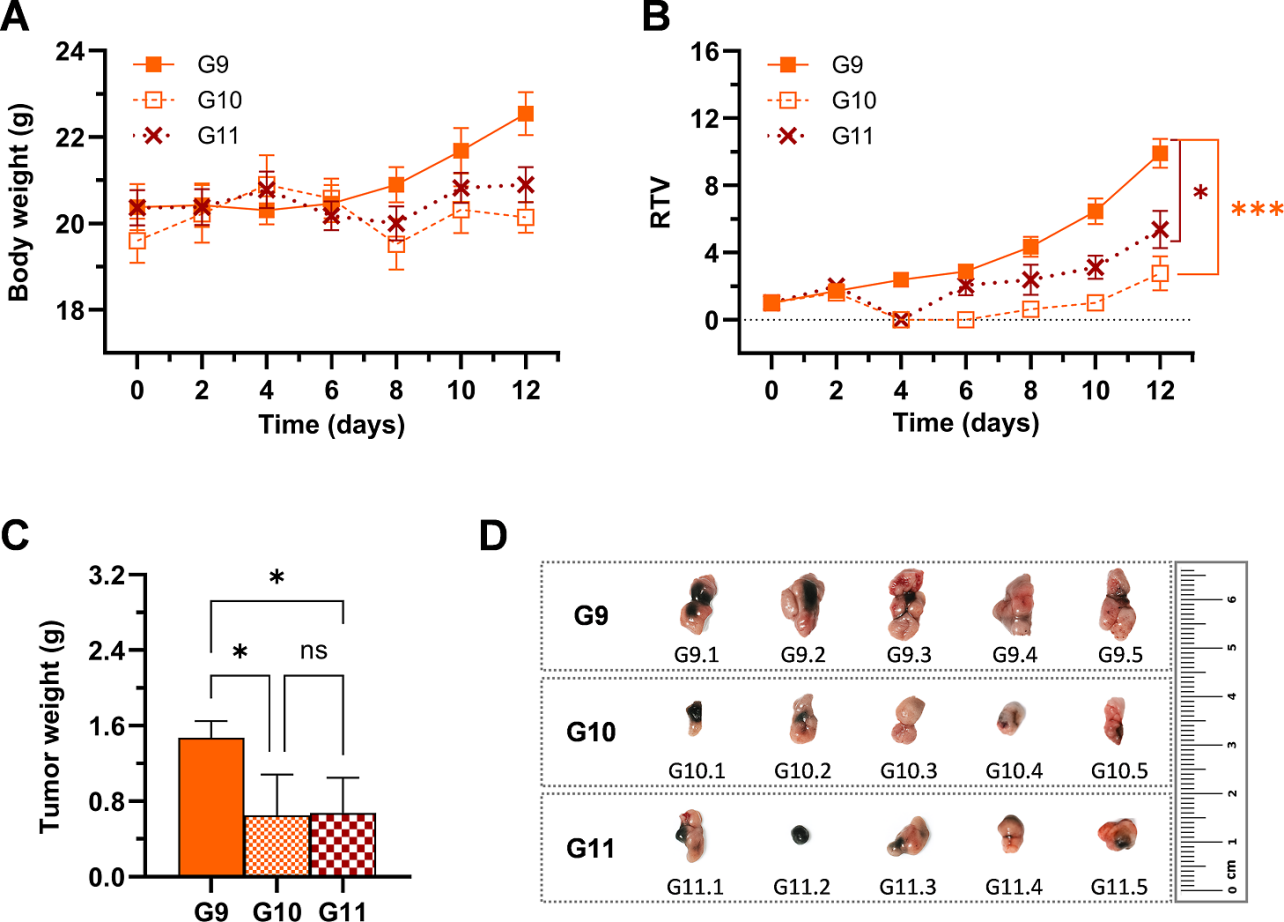
*In vivo* evaluation of **Ru6**’s PDT antitumor efficacy in a subcutaneous CT-26 tumor model in BALB/c mice using deep-red and NIR light. (A) Body weight (g), (B) RTV curves of mice throughout the 12 day study period, and (C) average tumor weights of mice on the day of sacrifice for the study III. Data are presented as mean ± SEM (*n* = 5 females). (D) Representative images of tumors from mice in groups G9 (**Ru6**, dark), G10 (**Ru6**, 660 nm), and G11 (**Ru6**, 780 nm) at the study end point. RTV values on day 12 and average tumor weight data were analyzed using a one-way ANOVA followed by Bonferroni’s multiple comparison test (asterisks: *p < 0.05, **p < 0.01, ***p < 0.001).

Two parameters were used to evaluate the *in vivo* antitumor efficacy of the PDT treatment with **Ru6** across the three different studies (I–III) and experimental groups (G1–G11): (1) measurement of tumor volumes (mm^3^) with a caliper, expressed as relative tumor volumes (RTV) and (2) comparison of tumor weight at the study end point. To our delight, as shown in Figure 6C, mice treated with **Ru6** and irradiated with deep-red light (G4) showed significantly lower RTV values compared to those of the **Ru6**-treated nonirradiated group (G3), which displayed values comparable to those of the vehicle-treated controls, either irradiated (G2) or not (G1). Remarkably, tumors in all mice in G4 became unmeasurable on day 4, mirroring the behavior of its parent Ru–COUBPY complex, **Ru3**, under the same experimental conditions (6 mg/kg, IT administration, 660 nm irradiation, 120 J/cm^2^).^40^ Although animals in G4 exhibited a slight recurrence in RTV after day 6 (Figure 6C), a statistically significant reduction in tumor weight was still observed at the study end point (Figure 6D). The results of study I further validated the potent tumor-destructive capacity of Ru–COUBPY complexes under deep-red light irradiation and, more importantly, confirmed that the *in vitro* photoactivity of **Ru6** was replicated in an animal model.

Given the excellent *in vitro* phototoxicity of **Ru6** toward CT-26 cancer cells under NIR light irradiation (see Tables 3 and 4), we next performed study II to assess its *in vivo* PDT efficacy study using one-photon NIR light at 780 nm. Gratifyingly, **Ru6** achieved comparable tumor growth inhibition with NIR light as with deep-red light, as evidenced by both RTV and tumor weight values (Figure 6F,G, respectively). For both parameters, statistical significance was observed between the G7 (**Ru6**, dark) and G8 (**Ru6**, light, 780 nm) groups. Furthermore, consistent with the results of study I, tumors in group G8 became unmeasurable by day 4, reproducing the tumor destructive capacity of **Ru6** even under 780 nm NIR irradiation.

Finally, study III was conducted to compare the *in vivo* antitumor efficacy of **Ru6** under 660 and 780 nm light irradiation in a parallel experiment, while also confirming the reproducibility of the results obtained in studies I and II. As anticipated, **Ru6** demonstrated comparable efficacy in inhibiting tumor growth under both deep-red and NIR light irradiation, with no statistically significant differences observed between groups G10 (**Ru6**, 660 nm) and G11 (**Ru6**, 780 nm) in terms of RTV or tumor weight (Figure 7B,C), further validating the potent *in vivo* PDT efficacy of **Ru6** in a third independent study. Images of the tumors from groups G9 to G11 are depicted in Figure 7D to illustrate the differences in tumor volume at the study end point. Overall, these results position the Ru–COUBPY complex **Ru6** as a potent PS that can be effectively activated with either deep-red (660 nm) or one-photon NIR (780 nm) light, offering a versatile platform for addressing a range of tumor indications with varying light penetration requirements.

## Conclusions

In this study, a series of π-extended Ru–COUBPY complexes (**Ru4–7**) were synthesized *via* the vinylogous extension of the COUBPY ligands. This structural modification enhanced their photophysical properties, including increased molar absorptivity and red-shifted absorption bands extending beyond 750 nm, compared with the parent compounds **Ru1** and **Ru3**. A key innovation in the synthetic strategy was the use of postcoordination ligand assembly through the Knoevenagel condensation, offering a versatile and modular approach for late-stage ligand functionalization of transition metal complexes.

The π-extended Ru–COUBPY complexes exhibited negligible dark toxicity in colorectal CT-26 cancer cells, while displaying strong phototoxicity upon irradiation with deep-red and NIR light under both normoxic and hypoxic conditions (*e.g.*, **Ru6** at 740 nm: [IC_50_]­normoxia = 177 nM, [IC_50_]­hypoxia = 597 nM). These potent photodynamic effects are attributed to the simultaneous generation of Type I (^•^O_2_^–^ and ^•^OH) and Type II (^1^O_2_) ROS within the mitochondria, ultimately inducing apoptosis.

Among the series, **Ru6** emerged as a lead candidate, combining favorable photophysical properties, efficient ROS photogeneration, and robust *in vitro* photobiological performance. *In vivo* PDT efficacy studies in mice bearing subcutaneous CT-26 tumors revealed that **Ru6** was well tolerated and achieved significant tumor growth inhibition following intratumoral administration (6 mg/kg) under both deep-red (660 nm) and NIR (780 nm) light irradiation.

Overall, **Ru6** stands out as a first-in-class Ru­(II) polypyridyl complex capable of eliciting potent *in vivo* antitumor responses under one-photon NIR activation at 780 nm—a clinically relevant wavelength with superior tissue penetration. This positions **Ru6** as a pioneering PS with a strong translational potential for PDT, particularly in the treatment of aggressive and treatment-resistant hypoxic tumors. Moreover, its efficient photoactivation across a broad wavelength range highlights its versatility for treating diverse tumor types with varying light penetration requirements. These findings establish **Ru6** not only as a promising therapeutic agent but also as a valuable platform for the future development of multifunctional metal-based PSs.

## Supplementary Material











## Notes

Full experimental procedures regarding the synthesis and chemical, photophysical, and photochemical characterization of the compounds; computational studies; cellular uptake and *in vitro* photobiological studies and *in vivo* PDT efficacy studies in mice; copies of HPLC traces; and NMR and HRMS spectra (PDF)
Fluorescence imaging video of **Ru4** time lapse 2 min (AVI)
Fluorescence imaging video of **Ru5** time lapse 2 min (AVI)
Fluorescence imaging video of **Ru6** time lapse 2 min (AVI)
Fluorescence imaging video of **Ru7** time lapse 2 min (AVI)
