# Supplementary material for: π‑Extended Ru–COUBPY Photosensitizers for In Vivo Anticancer Phototherapy Using One-Photon 780 nm Near-Infrared Light
Source: J Am Chem Soc. 2025 Dec 8;147(50):46291–304. doi: 10.1021/jacs.5c15343 (PMC12715793; doi:10.1021/jacs.5c15343)
Supplement: Supplementary file 1 [file ja5c15343_si_001.pdf]

SUPPORTING INFORMATION

π-Extended Ru-COUBPY photosensitizers for *in vivo* anticancer phototherapy using one-photon 780 nm near-infrared light

Diego Abad-Montero,[1] Eduardo Izquierdo-García,[1] Pierre Mesdom,[2] Albert Gandioso,[1] Elena de la Torre-Rubio,[1] Manel Bosch,[3] Juan Sanz-Villafruela,[4] Alba Deyà,[5] Marta Redrado,[2] Valentin V. Novikov,[6] José Luis Hernández,[5] Jorge Galino,[5] Marta E. Alberto,[7] Antonio Francés-Monerris,[8] Gilles Gasser[2,*] and Vicente Marchán[1,9,*]

[1] Departament de Química Inorgànica i Orgànica, Secció de Química Orgànica, Universitat de Barcelona (UB), and Institut de Biomedicina de la Universitat de Barcelona (IBUB), Martí i Franquès 1-11, E-08028 Barcelona, Spain. Email: vmarchan@ub.edu

[2] Chimie ParisTech, PSL University, CNRS, Institute of Chemistry for Life and Health Sciences, Laboratory for Inorganic Chemical Biology, F-75005 Paris, France. Email: gilles.gasser@chimieparistech.psl.eu

[3] Unitat de Microscòpia Òptica Avançada, Centres Científics i Tecnològics, Universitat de Barcelona, Av. Diagonal 643, E- 08028 Barcelona, Spain.

[4] Departamento de Química, Facultad de Ciencias, Universidad de Burgos, Plaza Misael Bañuelos s/n, E-09001 Burgos, Spain.

[5] Health and Biomedicine Department, Leitat Technological Center, Carrer de la Innovació 2, E-08225 Terrassa, Spain.

[6] Departament de Química Inorgànica i Orgànica, Secció de Química Inorgànica, Universitat de Barcelona (UB), and Institute of Nanoscience and Nanotechnology of the University of Barcelona (IN2UB), Martí i Franquès 1-11, E-08028 Barcelona, Spain.

[7] Dipartimento di Chimica e Tecnologie Chimiche, Università della Calabria, Arcavacata di Rende I-87036, Italy

[8] Institut de Ciència Molecular, Universitat de València, P.O. Box 22085, València 46071, Spain

[9] Serra-Húnter Professor at the Universitat de Barcelona, E-08028 Barcelona, Spain

Table of contents

1. Synthesis and characterization of the compounds

1.1. Materials and methods S3

1.2. Synthesis of COUBPY ligands **4-7** S4

1.3. NOESY spectra of COUBPY ligands **4-7** S16

1.4. Synthesis of Ru complex **12** and of Ru-COUBPY complexes S20

1.5. NOESY spectra of Ru-COUBPY complexes S25

1.6. HPLC analysis of Ru-COUBPY complexes S29

1.7. Lipophilicity determination S31

2.- Photophysical characterization: experimental and computational studies

2.1. Spectroscopic studies S33

2.2. Computational studies S38

3.- Dark and light stability studies in cell culture medium

3.1. Dark stability S44

3.3. Photostability S49

4.- Photochemical characterization: experimental and computational studies

4.1. Evaluation of singlet oxygen generation using SOSG S54

4.2. Quantification of singlet oxygen generation S56

4.3. Evaluation of superoxide anion radical generation using DHR123 S59

4.4. Evaluation of hydroxyl radical generation using HPF S61

4.5. Electron paramagnetic resonance (EPR) studies S63

4.6. Computational studies S64

4.7. Cyclic voltammetry S65

5. Cellular uptake by confocal microscopy S68

6. *In vitro* (photo)cytotoxicity determination of Ru-COUBPY complexes

6.1. Cell culture S73

6.2. (Photo)cytotoxicity evaluation in cancer cells S73

6.3. Cellular accumulation by ICP-MS S77

6.4. Cell apoptosis assay S77

6.5. Ferroptosis assay S79

7. *In vivo* PDT efficacy study of **Ru6** in BALB/c mice bearing subcutaneous CT-26 syngeneic colon tumors

7.1. Ethical animal procedures and animal housing conditions S80

7.2. Tumor cell line S80

7.3. Formulation of the compound S80

7.4. Irradiation systems for *in vivo* PDT studies S81

7.5. *In vivo* PDT efficacy study S82

8. ^{1}H and ^{13}C NMR spectra and HR ESI-MS of the compounds S84

9. References S116

1. Synthesis and characterization of the compounds

1.1. Materials and methods

Unless otherwise stated, common chemicals and solvents (HPLC grade or reagent grade quality) were purchased from commercial sources and used without further purification. A hot plate magnetic stirrer, together with an aluminum reaction block of the appropriate size, was used as the heating source in all reactions requiring heat. Aluminum plates coated with a 0.2 mm thick layer of silica gel 60 F_{254} were used for thin-layer chromatography analyses (TLC), whereas flash column chromatography purification was carried out using silica gel 60 (230-400 mesh). NMR spectra were recorded at 25 °C in 400 or 500 MHz spectrometers using the deuterated solvent as an internal deuterium lock. The residual protic signal of $CHCl_3$, CH_3OH and DMSO was used as a reference in ^1H and ^{13}C NMR spectra recorded in $CDCl_3$, CD_3OD and DMSO-d_6, respectively. Chemical shifts are reported in part per million (ppm) in the δ scale, coupling constants in Hz and multiplicity as follows: s (singlet), d (doublet), t (triplet), q (quartet), p (pentuplet), m (multiplet), dd (doublet of doublets), dt (doublet of triplets), td (triplet of doublets), br (broad signal), etc. The proton signals of the *E* and *Z* rotamers were identified by simple inspection of the ^1H spectrum and the rotamer ratio was calculated by peak integration. 2D-NOESY spectra were acquired in $CDCl_3$ or in CD_3OD with mixing times of 500 ms. Electrospray ionization mass spectra (ESI-MS) were recorded on an instrument equipped with single quadrupole detector coupled to an HPLC and high-resolution (HR) ESI-MS on an LC/MS-TOF instrument. Reversed-phase high-performance liquid chromatography (HPLC) analyses were carried out on a Jupiter Proteo C12 column (150 × 4.6 mm, 90 Å, 4 µm, flow rate: 1 mL/min) using linear gradients of 0.1% formic acid in Milli-Q H_2O (A) and 0.1% formic acid in ACN (B). The HPLC column was maintained at 25 °C. All final compounds were >95% pure by this method.

1.2. Synthesis of COUBPY ligands 4-7

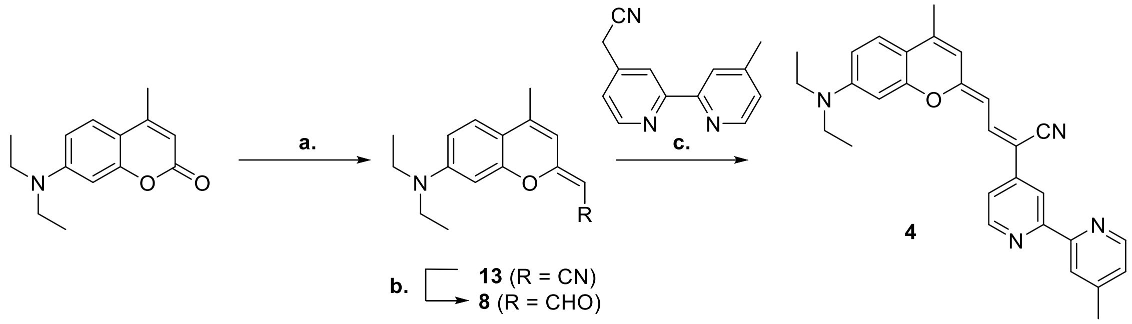

Scheme S1. Synthetic route for the preparation of COUBPY ligand **4**. Reagents and conditions: a) (1) CH_3CN, n-BuLi, THF, Ar atm, − 78 ºC, 30 min, (2) HCl aq, rt, 16 h, 92 %; b) (1) DIBAL-H, toluene, rt, 30 min, (2) Potassium sodium tartrate, rt, 30 min, 15%; c) piperidine, EtOH, 80 ºC, 16 h, 96%.

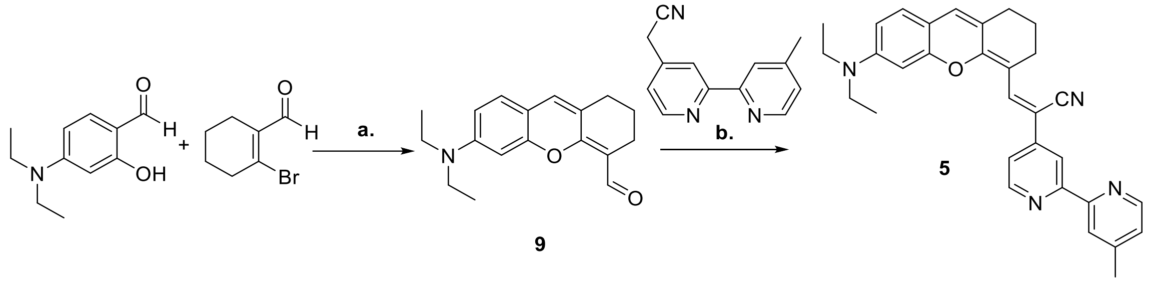

Scheme S2. Synthetic route for the preparation of COUBPY ligand **5**. Reagents and conditions: a) (1) Cs_2CO_3, DMF, rt, 36 h, 16%; b) piperidine, EtOH, 80 ºC, 16 h, 47%.

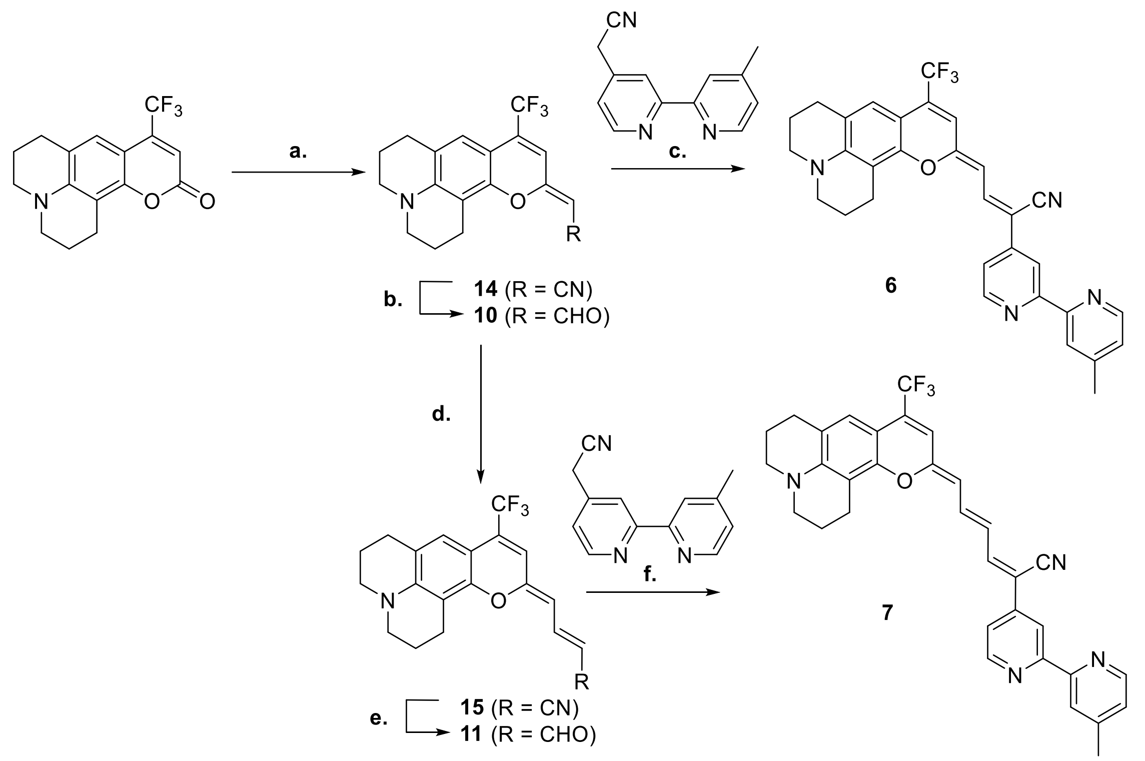

Scheme S3. Synthetic route for the preparation of COUBPY ligands **6-7**. Reagents and conditions: a) (1) CH_3CN, n-BuLi, THF, Ar atm, − 78 ºC, 30 min, (2) HCl aq, rt, 3 h, 70 %; b) (1) DIBAL-H, toluene, rt, 30 min, (2) Potassium sodium tartrate, rt, 30 min, 50%; c) piperidine, EtOH, 80 ºC, 16 h, 83 %; d) Ph_3PCHCN, Toluene, 60 ºC, 7 days, 84 %; e) (1) DIBAL-H, toluene, rt, 30 min, (2) Potassium sodium tartrate, rt, 30 min, 69 %; f) piperidine, EtOH, 80 ºC, 16h, 89 %.

Compound 13

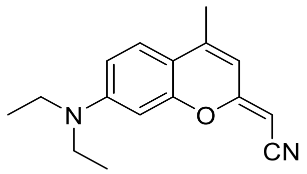

To a cold solution (-78 ºC) of CH_3CN (2.42 mL, 34.6 mmol) in anhydrous THF (40 mL), n-BuLi (13.8 mL, 2.5 M in hexanes, 34.6 mmol) was added under an Ar atmosphere. The resulting transparent and colorless solution was stirred for 15 min at -78 ºC, time after which the appearance of a white suspension was observed. Then, a solution of 7-(diethylamino)-4-methyl-2H-chromen-2-one coumarin (2.008 g, 8.65 mmol) in anhydrous THF (30 mL) was slowly added and the reaction mixture was stirred for 30 min at -78 ºC under Ar. The resulting white mixture was quenched by addition of saturated aqueous NH_4Cl (35 mL) while still at -78 ºC, and a white solid precipitated. The mixture was warmed to room temperature and extracted with AcOEt (3x50 mL). The combined organic phases were evaporated to dryness. To the resulting orange crude oil, 0.5 M aqueous HCl (200 mL) was added and the mixture stirred vigorously for 16 h. The solution turned into brown color and a yellow solid precipitated. The precipitate was dissolved by adding 50 mL of DCM and the mixture was extracted with DCM (3x100 mL), dried over anhydrous $MgSO_4$, filtered and evaporated to dryness under reduced pressure to obtain a brown solid. The crude mixture was purified by flash chromatography silica gel, 0-22% AcOEt in hexanes. 2.01 g of the title compound, as bright orange solid, were obtained (yield: 92%).

TLC: Rf (50% AcOEt in hexanes) 0.61.

^{1}H NMR (500 MHz, $CDCl_3$) δ (ppm): 7.20 (d, J = 8.8 Hz, 1H), 6.45 (dd, J = 8.8, 2.6 Hz, 1H), 6.41 (d, J = 1.3 Hz, 1H), 6.30 (d, J = 2.6 Hz, 1H), 4.42 (s, 1H), 3.38 (q, J = 7.1 Hz, 4H), 2.22 (dd, J = 1.3, Hz, 3H), 1.19 (t, J = 7.1 Hz, 6H).

^{13}C NMR (126 MHz, $CDCl_3$) δ (ppm): 166.9, 165.5, 154.6, 154.5, 150.3, 150.2, 141.5, 140.8, 125.2, 125.0, 119.9, 119.0, 111.9, 110.9, 109.8, 109.8, 107.8, 107.7, 97.8, 97.3, 66.1, 65.0, 44.8, 18.1, 18.1, 12.7, 12.7.

LR-ESI MS (ESI): m/z 255.2 calc. for $[C_{16}H_{18}N_2O+H]^+$: 255.15.

Compound 8

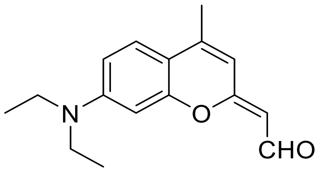

Nitrile coumarin of formula **13** (500 mg, 1.97 mmol) was dissolved in anhydrous toluene (25 mL) under an Ar atmosphere and the mixture was let stir for 10 min (orange solution). Then, 1 M toluene solution of DIBALH was added (3.0 mL, 2.95 mmol) and the reaction mixture stirred for 30 min at room temperature under an Ar atmosphere. The crude was cooled in an ice-bath and acetone (10 mL) was added for decomposition of the excess reagent. After addition of saturated potassium sodium tartrate (40 mL), the crude was extracted with DCM (3 x 50 mL) and the combined organic phases washed with brine (50 mL), dried over anhydrous $MgSO_4$, filtered and evaporated to dryness under reduced pressure. The compound was purified by silica column chromatography with hexanes increasing the polarity with DCM first (0-100%) and with MeOH then (0-0.5%). 78 mg of the title compound, as a yellow solid, were obtained (yield: 15%).

TLC: Rf (5% MeOH in DCM) 0.52.

^{1}H NMR (400 MHz, Chloroform-*d*) δ (ppm) ***Z*-isomer**: 10.09 (d, *J* = 8.7 Hz, 1H), 7.22 (d, *J* = 7.4 Hz, 1H), 6.53 – 6.44 (m, 1H), 6.38 (d, *J* = 2.6 Hz, 1H), 5.96 (q, *J* = 1.2 Hz, 1H), 5.11 (d, *J* = 8.7 Hz, 1H), 3.43 – 3.34 (m, 4H), 2.19 (d, *J* = 1.1 Hz, 3H), 1.22 – 1.15 (m, 6H). ***E*-isomer**: 9.71 (d, *J* = 7.1 Hz, 1H), 7.24 (d, *J* = 7.5 Hz, 2H), 7.07 (s, 1H), 6.53 – 6.44 (m, 1H), 6.35 (d, *J* = 2.6 Hz, 1H), 5.49 (d, *J* = 7.1 Hz, 1H), 3.43 – 3.34 (m, 4H), 2.26 (d, *J* = 1.1 Hz, 3H), 1.22 – 1.15 (m, 6H).

^{13}C-NMR ($CDCl_3$, 126 MHz) δ (ppm) ***Z*** and ***E*-isomer**: 187.6, 186.9, 165.4, 154.6, 154.5, 150.5, 150.4, 143.1, 125.2, 125.2, 113.4, 110.7, 110.5, 108.4, 108.2, 102.5, 97.3, 97.3, 44.7, 44.7, 18.5, 18.2, 12.6, 12.6.

LR-ESI MS (ESI): *m/z* 258.2 calc. for $[C_{16}H_{19}NO_2+H]^+$: 258.15.

COUBPY ligand 4

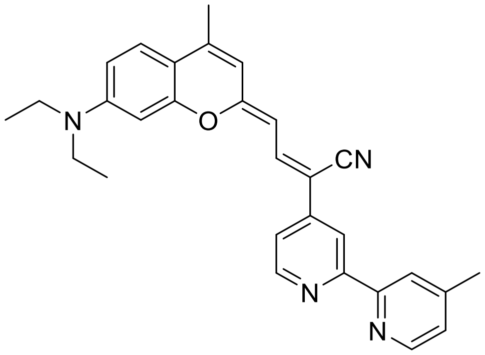

To a solution of compound **8** (47 mg, 0.18 mmol) in absolute ethanol (10 mL), piperidine (84 mg, 1.0 mmol) and 2-(4'-methyl-[2,2'-bipyridin]-4-yl)acetonitrile (44 mg, 0.20 mmol) were added. The reaction mixture was stirred at 80 ^{o}C overnight. After evaporation to dryness under reduced pressure, the compound was purified by silica column chromatography with hexanes increasing the polarity with DCM first (0-100%) and with MeOH then (0-2%). 79 mg of a violet solid, identified as the COUBPY ligand **4**, were obtained (yield: 96%).

TLC: Rf (5% MeOH in DCM) 0.38.

^{1}H NMR (400 MHz, $CDCl_3$) δ (ppm): 8.61 (d, *J* = 5.3 Hz, 1H), 8.57 (d, *J* = 5.2 Hz, 1H), 8.55 (d, *J* = 2.0 Hz, 1H), 8.25 (d, *J* = 13.7 Hz, 1H), 8.24 (s, 1H), 7.46 (dd, *J* = 5.2, 1.9 Hz, 1H), 7.20 (d, *J* = 8.6 Hz, 1H), 7.15 (dd, *J* = 4.9, 1.8, 0.8 Hz, 1H), 6.61 – 6.40 (m, 2H), 6.07 (d, *J* = 1.3 Hz, 1H), 5.82 (d, *J* = 12.3 Hz, 1H), 3.43 (q, *J* = 7.1 Hz, 3H), 2.45 (d, *J* = 0.9 Hz, 3H), 2.21 (d, *J* = 1.1 Hz, 3H), 1.24 (t, *J* = 7.0 Hz, 6H).

^{13}C NMR (101 MHz, $CDCl_3$) δ (ppm): 159.3, 156.6, 156.1, 155.2, 150.4, 149.7, 149.0, 148.3, 144.1, 141.1, 139.2, 125.2, 124.9, 122.4, 119.5, 118.5, 115.8, 115.2, 111.2, 108.1, 100.7, 99.5, 97.6, 44.8, 21.3, 18.3, 12.8.

HR-ESI MS (ESI): *m/z* 449.2336 calc. for $[C_{29}H_{28}N_4O+H]^+$: 449.2336.

Compound 9

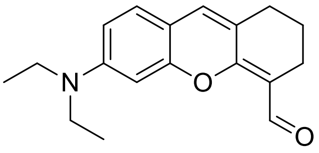

The coumarin aldehyde **9** was synthesized according to a previously described procedure.[1]

COUBPY ligand 5

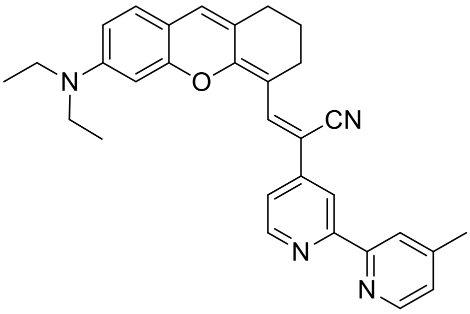

To a solution of compound **9** (92 mg, 0.32 mmol) in absolute ethanol (10 mL), piperidine (166 mg, 2.0 mmol) and 2-(4'-methyl-[2,2'-bipyridin]-4-yl)acetonitrile (68 mg, 0.32 mmol) were added. The reaction mixture was stirred at 80 ºC overnight. After evaporation to dryness under reduced pressure, the compound was purified by silica column chromatography with hexanes increasing the polarity with DCM first (0-100%) and with MeOH then (0-1%). 73 mg of a violet solid, identified as the COUBPY ligand **5**, were obtained (yield: 47%).

TLC: Rf (5% MeOH in DCM) 0.59.

^{1}H NMR (400 MHz, $CDCl_3$) δ (ppm): 8.66 (dd, *J* = 2.1, 0.8 Hz, 1H), 8.65 (dd, *J* = 5.3, 0.7 Hz, 1H), 8.53 (dd, *J* = 5.0, 0.8 Hz, 1H), 8.35 (d, *J* = 0.9 Hz, 1H), 8.28 – 8.19 (m, 1H), 7.53 (dd, *J* = 5.3, 2.1 Hz, 1H), 7.14 (ddd, *J* = 4.9, 1.7, 0.8 Hz, 1H), 6.99 (d, *J* = 8.4 Hz, 1H), 6.56 (s, 1H), 6.42 (s, 1H), 6.41 (dd, *J* = 8.4, 2.5 Hz, 1H), 3.41 (q, *J* = 7.1 Hz, 4H), 3.04 (t, *J* = 6.1 Hz, 2H), 2.55 (t, *J* = 7.6 Hz, 2H), 2.46 (s, 1H), 1.83 (p, *J* = 6.2 Hz, 2H), 1.22 (d, *J* = 7.1 Hz, 6H).

^{13}C NMR (101 MHz, $CDCl_3$) δ (ppm): 156.7, 156.3, 156.1, 155.0, 149.7, 149.1, 148.2, 145.8, 139.2, 127.5, 127.3, 124.9, 124.1, 122.2, 120.0, 119.8, 116.7, 110.9, 109.0, 108.1, 98.8, 97.4, 44.7, 29.6, 26.3, 21.4, 21.4, 12.8.

HR-MS (ESI): *m/z* 475.2495 calc. for $[C_{31}H_{30}N_4O+H]^+$: 475.2492.

Compound 14

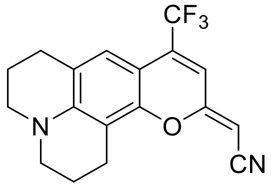

To a cold solution (-78 ºC) of CH_3CN (0.7 mL, 13.4 mmol) in anhydrous THF (40 mL), n-BuLi (5.2 mL, 2.5 M in hexanes, 13 mmol) was added under an Ar atmosphere. The resulting transparent and colorless solution was stirred for 15 min at -78 ºC, time after which the appearance of a white suspension was observed. Then, a solution of 2,3,6,7-tetrahydro-9-trifluoromethyl-1H,5H-quinolizino[9,1-gh] coumarin (1.023 g, 3.31 mmol) in anhydrous THF (35 mL) was slowly added and the reaction mixture was stirred for 30 min at -78 ºC under Ar. The resulting orange mixture was quenched by addition of saturated aqueous NH_4Cl (35 mL) while still at -78 ºC, and a white solid precipitated. The mixture was warmed to room temperature and extracted with AcOEt (3x50 mL). The combined organic phases were evaporated to dryness. To the resulting crude oil, 0.5 M aqueous HCl (200 mL) was added and the mixture stirred vigorously for 3 h. The solution color turned from orange to brown. This solution was extracted with AcOEt (3x80 mL), dried over anhydrous $MgSO_4$, filtered and evaporated to dryness under reduced pressure. The crude mixture was purified by flash chromatography silica gel, 0-15% AcOEt in hexanes. 760 mg of the title compound, as bright orange solid, were obtained (yield: 70%).

TLC: Rf (DCM) 0.7 (25% AcOEt in hexanes) 0.38.

^{1}H-NMR (500 MHz, $CDCl_3$) δ (ppm) ***Z*-isomer**: 6.89 (s, 1H), 6.35 (s, 1H), 4.44 (s, 1H), 3.29-3.23 (m, 4H), 2.74 (t, *J* = 6.0 Hz, 4H), 2.02-1.96 (m, 4H); ***E*-isomer**: 6.92 (s, 1H), 6.83 (s, 1H), 4.76 (s, 1H), 3.29-3.23 (m, 4H), 2.89 (t, *J* = 6.5 Hz, 2H), 2.74 (t, *J* = 6.0 Hz, 2H), 2.02-1.96 (m, 4H).

^{13}C-NMR (126 MHz, $CDCl_3$) δ (ppm) ***Z*-isomer**: 162.9, 149.6, 145.4, 131.3 (q, *J* = 31 Hz), 122.4, 122.3 (q, *J* = 273 Hz), 122.2, 118.5 (q, *J* = 6 Hz), 117.2, 111.7, 108.5, 103.5, 71.4, 50.3, 22.6, 21.4, 20.5, 20.3.

^{19}F-NMR (470 MHz, $CDCl_3$): δ (ppm) ***Z*-isomer**: -64.2 (s, 3F); ***E*-isomer**: -64.1 (s, 3F).

HR-MS (ESI): *m/z* 332.1209 calc. for $[C_{18}H_{15}F_3N_2O+H]^+$: 333.1215.

Compound 10

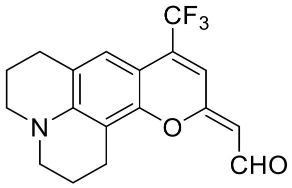

Compound **14** (300 mg, 0.90 mmol) was dissolved in anhydrous toluene (35 mL) under an Ar atmosphere and the mixture was let stir for 10 min (orange solution). Then, 1 M toluene solution of DIBALH was added (1.35 mL, 1.35 mmol) and the reaction mixture stirred for 30 min at room temperature under an Ar atmosphere (the color changed to maroon). The crude was cooled in an ice-bath and acetone (4 mL) was added for decomposition of the excess reagent (the color changed from maroon to dark red). After addition of saturated potassium sodium tartrate (25 mL), the crude was extracted with AcOEt (3 x 25 mL) and the organic phases washed with water (25 mL) and brine (25 mL), dried over anhydrous $MgSO_4$, filtered and evaporated to dryness under reduced pressure. The compound was purified by silica column chromatography with hexanes increasing the polarity with DCM first (0-100%) and with MeOH then (0-0.5%). 150 mg of the title compound, as a red solid, were obtained (yield: 50%).

TLC: Rf (5% MeOH in DCM) 0.74.

^{1}H-NMR ($CDCl_3$, 400 MHz) δ (ppm) ***Z*-isomer**: 10.18 (d, *J* = 8.4 Hz, 1H), 6.92 (s, 1H), 6.38 (s, 1H), 5.30 (d, *J* = 8.4 Hz, 1H), 3.32-3.24 (m, 4H), 2.82 (t, *J* = 6.4 Hz, 2H), 2.73 (t, *J* = 6.4 Hz, 2H), 2.00-1.94 (m, 4H); ***E*-isomer**; 9.80 (d, *J* = 6.8 Hz, 1H), 7.46 (s, 1H), 6.94 (s, 1H), 5.73 (d, *J* = 6.8 Hz, 1H), 3.32-3.24 (m, 4H), 2.82 (t, *J* = 6.4 Hz, 2H), 2.73 (t, *J* = 6.4 Hz, 2H), 2.00-1.94 (m, 4H).

^{13}C-NMR (101 MHz, $CDCl_3$) δ (ppm) ***Z*-isomer**:187.7, 162.6, 150.0, 146.3, 132.4 (q, *J* = 31 Hz), 122.5 (q, *J* = 273 Hz), 122.3, 118.3, 112.7 (q, *J* = 6 Hz), 107.0, 106.6, 103.6, 50.1, 49.6, 27.7, 21.5, 20.6, 20.4.

^{19}F NMR (470 MHz, $CDCl_3$) δ (ppm) ***E*-isomer:** -63.91 (s, 3F); ***Z*-isomer:** -64.05 (s, 3F).

HR-MS (ESI): *m/z* 336.1206 calc. For $[C_{18}H_{16}F_3NO_2+H]^+$: 336.1211.

COUBPY ligand 6

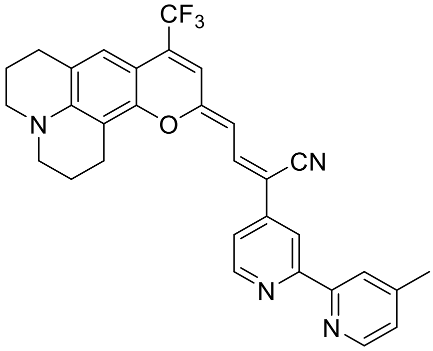

To a solution of the compound **12** (50 mg, 0.15 mmol) in absolute ethanol (7 mL), piperidine (70 mg, 0.83 mmol) and 2-(4'-methyl-[2,2'-bipyridin]-4-yl)acetonitrile (46 mg, 0.22 mmol) were added. The reaction mixture (dark maroon) was stirred at 80 ºC overnight. After evaporation to dryness under reduced pressure, the compound was purified by silica column chromatography with hexanes increasing the polarity with DCM first (0-100%) and with MeOH then (0-2%). 66 mg of a violet solid, identified as the COUBPY ligand **6**, were obtained (yield: 83%).

TLC: Rf (5% MeOH in DCM) 0.4.

^{1}H NMR (500 MHz, $CDCl_3$) δ (ppm): 8.65 (d, *J* = 6.1 Hz, 1H), 8.64 (s, 1H), 8.54 (d, *J* = 4.9 Hz, 1H), 8.23 (s, 1H), 8.21 (d, *J* = 12.2 Hz, 1H), 7.44 (dd, *J* = 5.2, 2.0 Hz, 1H), 7.15 (d, *J* = 4.9 Hz, 1H), 6.89 (s, 1H), 6.47 (s, 1H), 6.03 (d, *J* = 12.1 Hz, 1H), 3.29 (q, *J* = 6.4 Hz, 4H), 2.96 (t, *J* = 6.5 Hz, 2H), 2.74 (t, *J* = 6.3 Hz, 1H), 2.45 (s, 3H), 2.10 – 2.03 (m, 2H), 2.01 – 1.94 (m, 2H).

^{19}F NMR (471 MHz, $CDCl_3$) δ (ppm): -63.62.

^{13}C NMR (126 MHz, $CDCl_3$) δ(ppm): 156.9, 156.3, 155.7, 150.3, 149.9, 149.2, 148.3, 146.2, 143.1, 137.7, 130.3 (q, *J* = 31 Hz), 125.1, 122.6 (q, *J* = 273 Hz), 122.3, 122.2, 119.3, 118.1, 117.4, 116.3, 114.1 (q, *J* = 6 Hz), 107.0, 104.8, 104.2, 103.6, 50.1, 49.6, 27.7, 21.6, 21.4, 20.9, 20.6.

HR-ESI MS (ESI): *m/z* 527.2077 calc. for $[C_{31}H_{25}F_3N_4O+H]^+$: 527.2053.

Compound 15

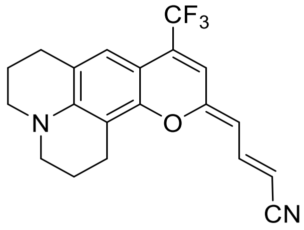

To a solution of compound **10** (207 mg, 0.62 mmol) in toluene (15 mL), (triphenylphosphoranylidene)acetonitrile (850 mg, 2.82 mmol) was added, and the resulting the solution was degassed with Ar and then the vial was sealed. The mixture was let stir at 60 ºC for 7 days. Once the starting aldehyde was totally consumed, deionized water was added to the crude and then extracted with AcOEt (3 x 30 mL). The combined organic phases were dried over anhydrous $MgSO_4$, filtered, evaporated to dryness under reduced pressure, and the crude was purified by silica colum chromatography with AcOEt in hexanes (0-12%). 184 mg of the title compound, as a red solid, were obtained (yield: 84%).

TLC: Rf (50% AcOEt in Hexanes) 0.90.

^{1}H NMR (500 MHz, $CDCl_3$) δ(ppm) ***Z,E*-isomer**: 7.50 (dd, *J* = 15.9, 11.7 Hz, 1H), 6.82 (s, 1H), 6.26 (s, 1H), 5.41 (d, *J* = 11.7 Hz, 1H), 5.16 (d, *J* = 15.9 Hz, 1H), 3.26 – 3.21 (m, 4H), 2.80 – 2.74 (m, 2H), 2.70 (t, *J* = 6.4 Hz, 2H), 2.03 – 1.92 (m, 4H); ***Z,Z*-isomer**: 7.36 (dd, *J* = 12.0, 10.7 Hz, 1H), 6.82 (s, 1H), 6.36 (s, 1H), 5.80 (d, *J* = 12.0 Hz, 1H), 4.94 (dd, *J* = 10.7, 1.0 Hz, 1H), 3.26 – 3.21 (m, 4H), 2.80 – 2.74 (m, 2H), 2.70 (t, *J* = 6.4 Hz, 2H), 2.03 – 1.92 (m, 4H).

^{13}C NMR (126 MHz, $CDCl_3$) δ (ppm) ***Z*-rotamer + *E*-rotamer:** 153.9, 153.5, 149.99, 149.96, 145.7, 145.6, 143.4, 141.9, 129.1 (q, *J* = 32 Hz), 122.9 (q, *J* = 273 Hz), 122.1, 122.0, 120.5, 118.2, 117.3, 117.2, 114.7 (q, *J* = 6 Hz), 114.2 (q, *J* = 6.1 Hz), 107.2, 107.1, 104.5, 104.0, 103.7, 103.6, 92.0, 90.42, 50.1, 49.5, 49.5, 29.8, 27.6, 21.7, 20.9, 20.8, 20.7, 20.6.

^{19}F NMR (470 MHz, $CDCl_3$) δ (ppm) ***E*-isomer: -**64.17 (s, 3F); ***Z*-isomer:** -64.20 (s, 3F).

HR-MS (ESI): *m/z* 359.1366 calc. for $[C_{20}H_{17}F_3N_2O+H]^+$: 359.1357.

Compound 11

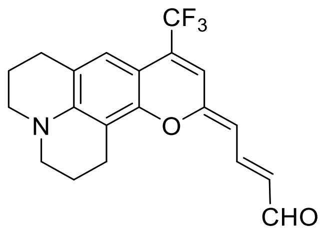

Compound **15** (103 mg, 0.29 mmol) was dissolved in anhydrous THF (30 mL) under an Ar atmosphere and the mixture was cooled in an ice bath. Then, 1 M toluene solution of DIBALH was added (3.0 mL, 2.95 mmol) and the reaction mixture stirred for 30 min at room temperature under an Ar atmosphere. The crude was cooled in an ice-bath and acetone (10 mL) was added for decomposition of the excess reagent. After addition of saturated potassium sodium tartrate (25 mL) and let stir for 30 min, the crude was extracted with DCM (3 x 50 mL) and the combined organic phases washed with brine (50 mL), dried over anhydrous $MgSO_4$, filtered and evaporated to dryness under reduced pressure. The compound was purified by silica column chromatography with hexanes increasing the polarity with DCM first (0-100%) and with MeOH then (0-0.5%). 78 mg of the title compound, as a yellow solid, were obtained (yield: 15%).

TLC: Rf (5% MeOH in DCM) 0.52.

^{1}H NMR ($CDCl_3$, 500 MHz) δ (ppm) ***Z*-isomer**: 9.56 (d, *J* = 8.1 Hz, 1H), 7.62 (dd, *J* = 15.1, 11.8 Hz, 1H), 6.85 (s, 1H), 6.35 (s, 1H), 6.11 (dd, *J* = 15.1, 8.1 Hz, 1H), 5.59 (d, *J* = 11.8 Hz, 1H), 3.27 – 3.24 (m, 4H), 2.84 (t, *J* = 6.5 Hz, 2H), 2.72 (t, *J* = 6.2 Hz, 2H), 2.02 (qt, *J* = 6.5 Hz, 2H), 1.96 (qt, *J* = 6.2 Hz, 2H); ***E*-isomer**: 9.52 (d, *J* = 8.1 Hz, 1H), 7.42 (dd, *J* = 14.8, 12.6 Hz, 1H), 6.85 (s, 1H), 6.35 (s, 1H), 6.08 (dd, *J* = 14.8, 8.1 Hz, 1H), 6.01 (d, *J* = 12.6 Hz, 1H), 3.27 – 3.24 (m, 4H), 2.84 (t, *J* = 6.5 Hz, 2H), 2.72 (t, *J* = 6.2 Hz, 2H), 2.02 (qt, *J* = 6.5 Hz, 2H), 1.96 (qt, *J* = 6.2 Hz, 2H).

^{13}C NMR (126 MHz, $CDCl_3$) δ (ppm) ***Z*-isomer**:193.4, 154.7, 150.0, 145.7, 145.3, 129.3 (q, *J* = 31.6 Hz), 127.2, 122.3 (q, *J* = 273.7 Hz), 122.0, 117.4, 114.4 (q, *J* = 6.3 Hz), 107.0, 105.5, 103.7, 50.0, 49.4, 27.6, 21.5, 20.7, 20.5.

^{19}F NMR (470 MHz, $CDCl_3$) δ (ppm) ***Z*-isomer:** -64.12 (s, 3F); ***E*-isomer:** -64.00 (s, 3F).

HR-MS (ESI): *m/z* 362.1362 calc. for $[C_{20}H_{17}F_3N_2O+H]^+$: 362.1359.

COUBPY ligand 7

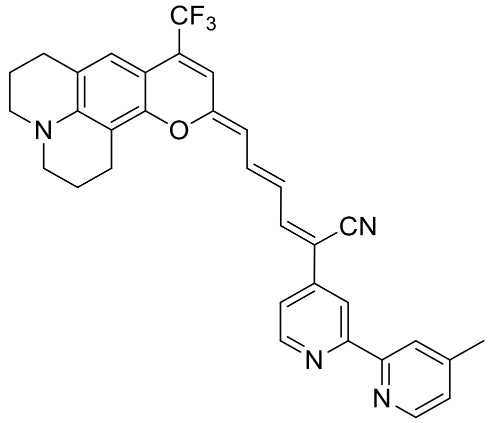

To a solution of compound **11** (32 mg, 0.088 mmol) in absolute ethanol (10 mL), piperidine (45 mg, 0.531 mmol) and 2-(4'-methyl-[2,2'-bipyridin]-4-yl)acetonitrile (23 mg, 0.11 mmol) were added. The reaction mixture was stirred at 80 ºC overnight. After evaporation to dryness under reduced pressure, the compound was purified by silica column chromatography with hexanes increasing the polarity with DCM first (0-100%) and with MeOH then (0-1%). 44 mg of a violet solid, identified as the ligand **COUBPY7**, were obtained (yield: 89%).

TLC: Rf (5% MeOH in DCM) 0.59.

^{1}H NMR (500 MHz, $CDCl_3$) δ (ppm): 8.66 (dd, *J* = 5.2, 0.8 Hz, 1H), 8.57 (d, *J* = 5.0 Hz, 1H), 8.55 (d, *J* = 2.0 Hz, 1H), 8.29 – 8.27 (m, 1H), 7.77 (d, *J* = 11.8 Hz, 1H), 7.48 (dd, *J* = 5.2, 1.9 Hz, 1H), 7.32 (dd, *J* = 14.4, 12.0 Hz, 1H), 7.17 (dd, *J* = 5.1, 1.7 Hz, 1H), 6.83 (t, *J* = 12.0 Hz, 1H), 6.83 (s, 1H), 6.35 (s, 1H), 5.59 (d, *J* = 11.8 Hz, 1H), 3.29 – 3.22 (m, 3H), 2.90 (t, *J* = 6.5 Hz, 2H), 2.71 (t, *J* = 6.3 Hz, 2H), 2.46 (s, 3H), 2.07 (q, *J* = 6.4 Hz, 2H), 1.96 (d, *J* = 5.8 Hz, 2H).

^{13}C NMR (101 MHz, $CDCl_3$) δ (ppm): 156.6, 155.5, 153.4, 150.2, 150.0, 148.9, 148.4, 145.5, 142.6, 137.9, 127.8 (q, *J* = 31.6 Hz), 125.1, 125.0, 122,7 (q, *J* = 273.7 Hz), 122.3, 121.8, 120.1, 117.2, 117.0, 115.3, 115.2 (q, *J* = 6.0 Hz), 107.9, 107.2, 106.1, 104.2, 50.0, 49.5, 27.5, 21.6, 21.2, 20.8, 20.7.

^{19}F NMR (471 MHz, $CDCl_3$) δ (ppm): -63.99.

HR-MS (ESI): *m/z* 553.2205 calc. for $[C_{33}H_{27}F_3N_4O+H]^+$: 553.2210.

1.3. NOESY spectra of COUBPY ligands 4-7

COUBPY ligand 4

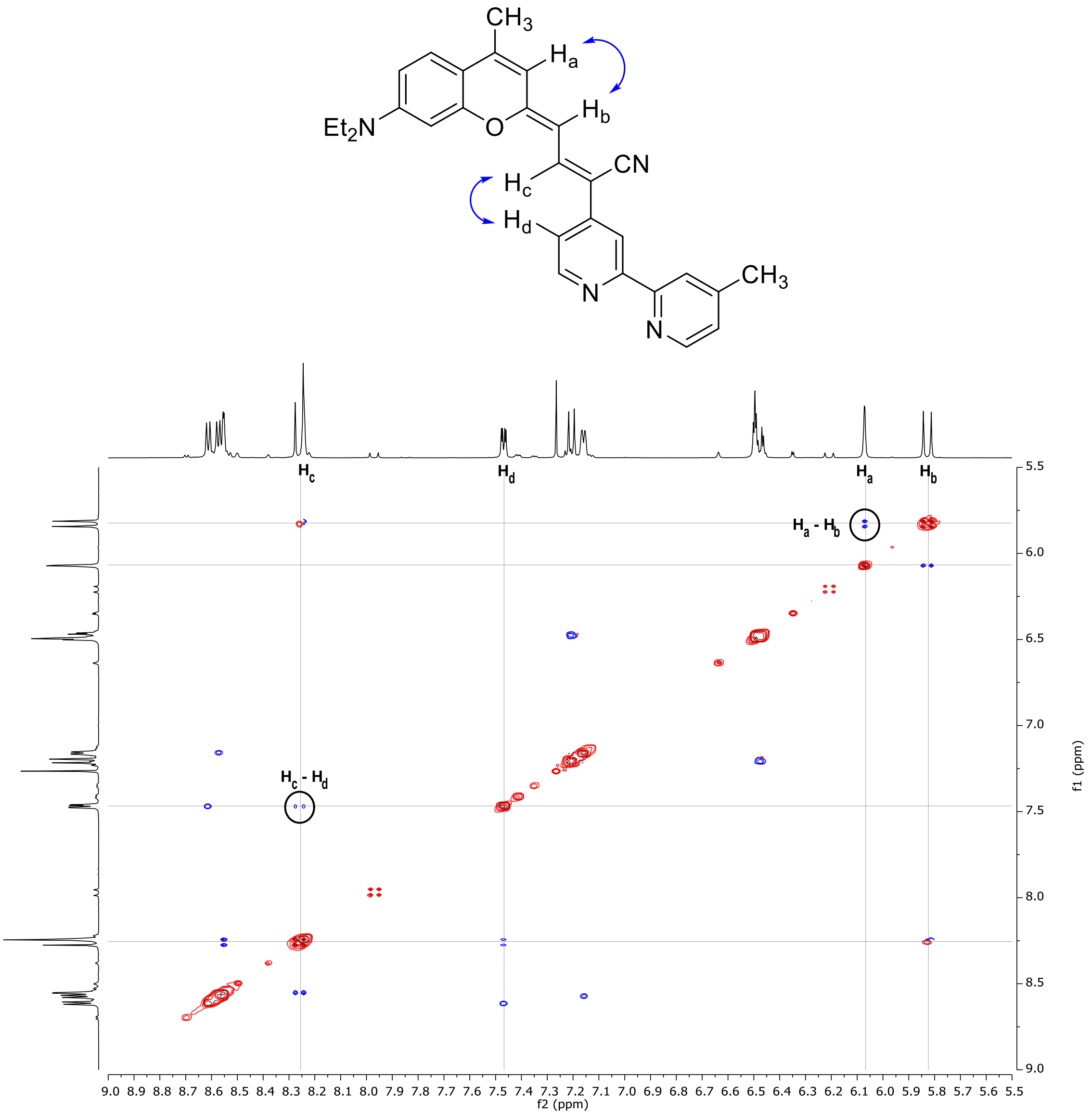

Figure S1. Expansion of the 2D NOESY spectrum of COUBPY ligand **4** in $CDCl_3$ showing diagnostic NOE cross-peaks.

COUBPY ligand 5

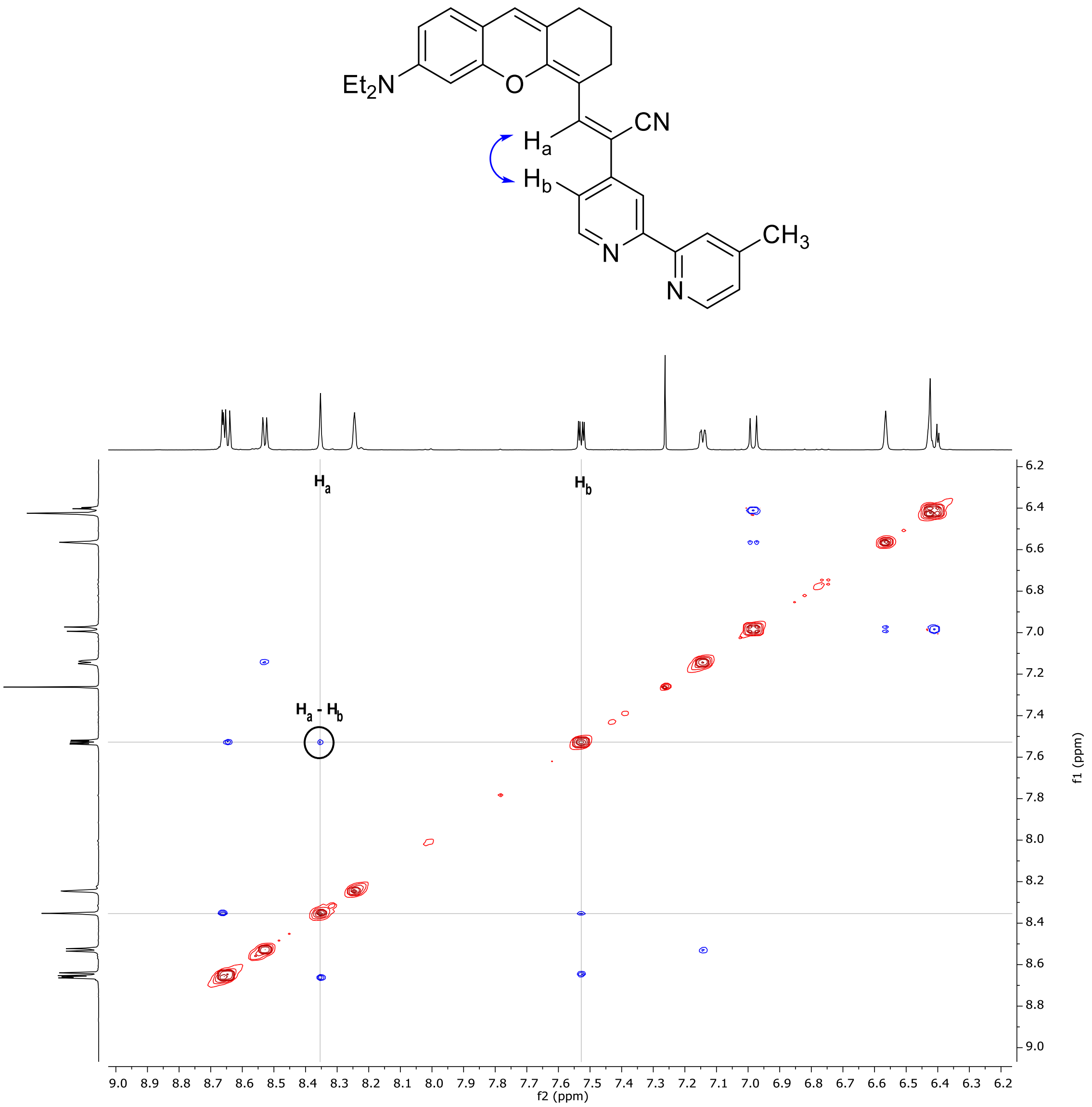

Figure S2. Expansion of the 2D NOESY spectrum of COUBPY ligand **5** in $CDCl_3$ showing diagnostic NOE cross-peaks.

COUBPY ligand 6

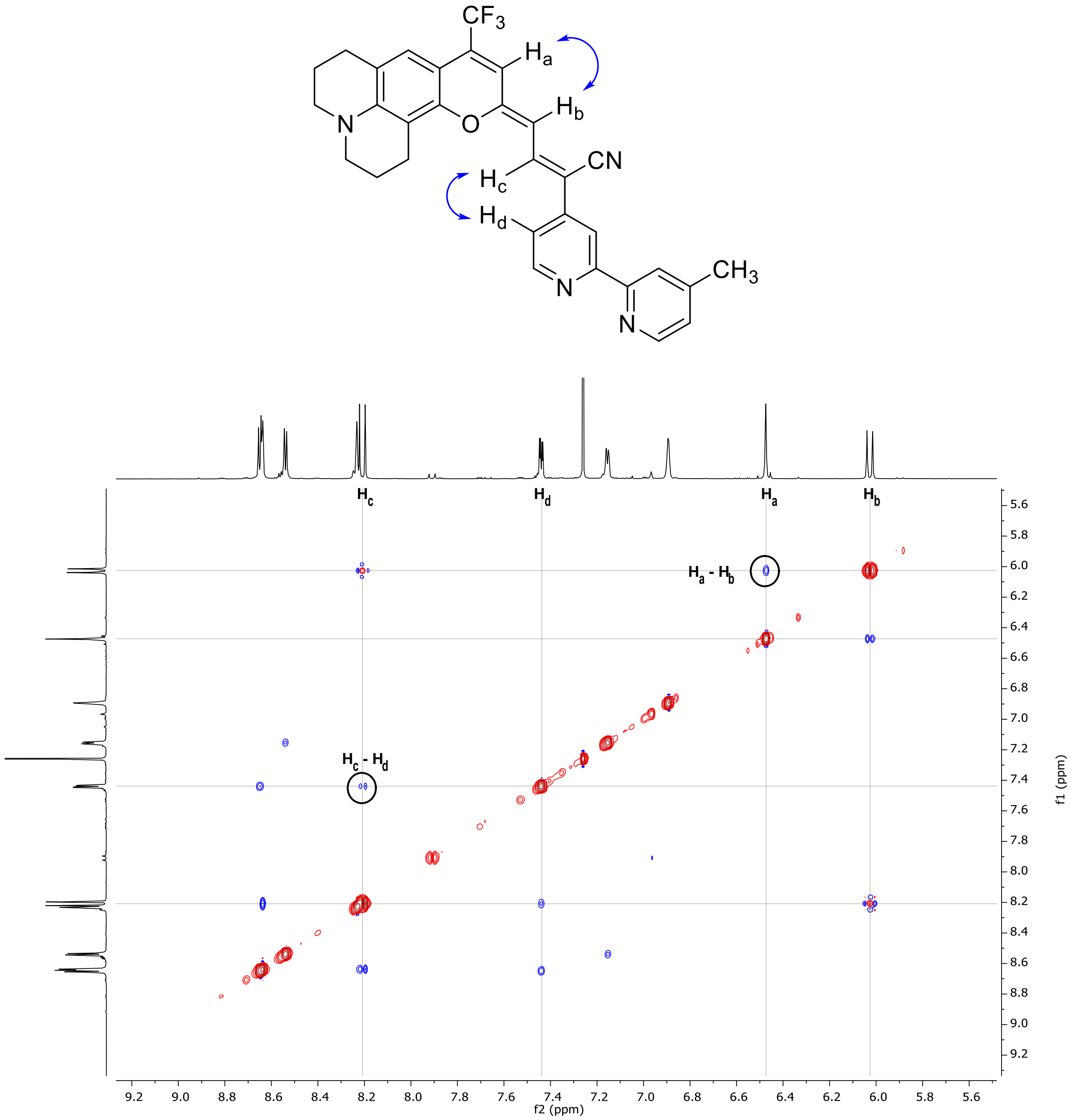

Figure S3. Expansion of the 2D NOESY spectrum of COUBPY ligand **6** in $CDCl_3$ showing diagnostic NOE cross-peaks.

COUBPY ligand 7

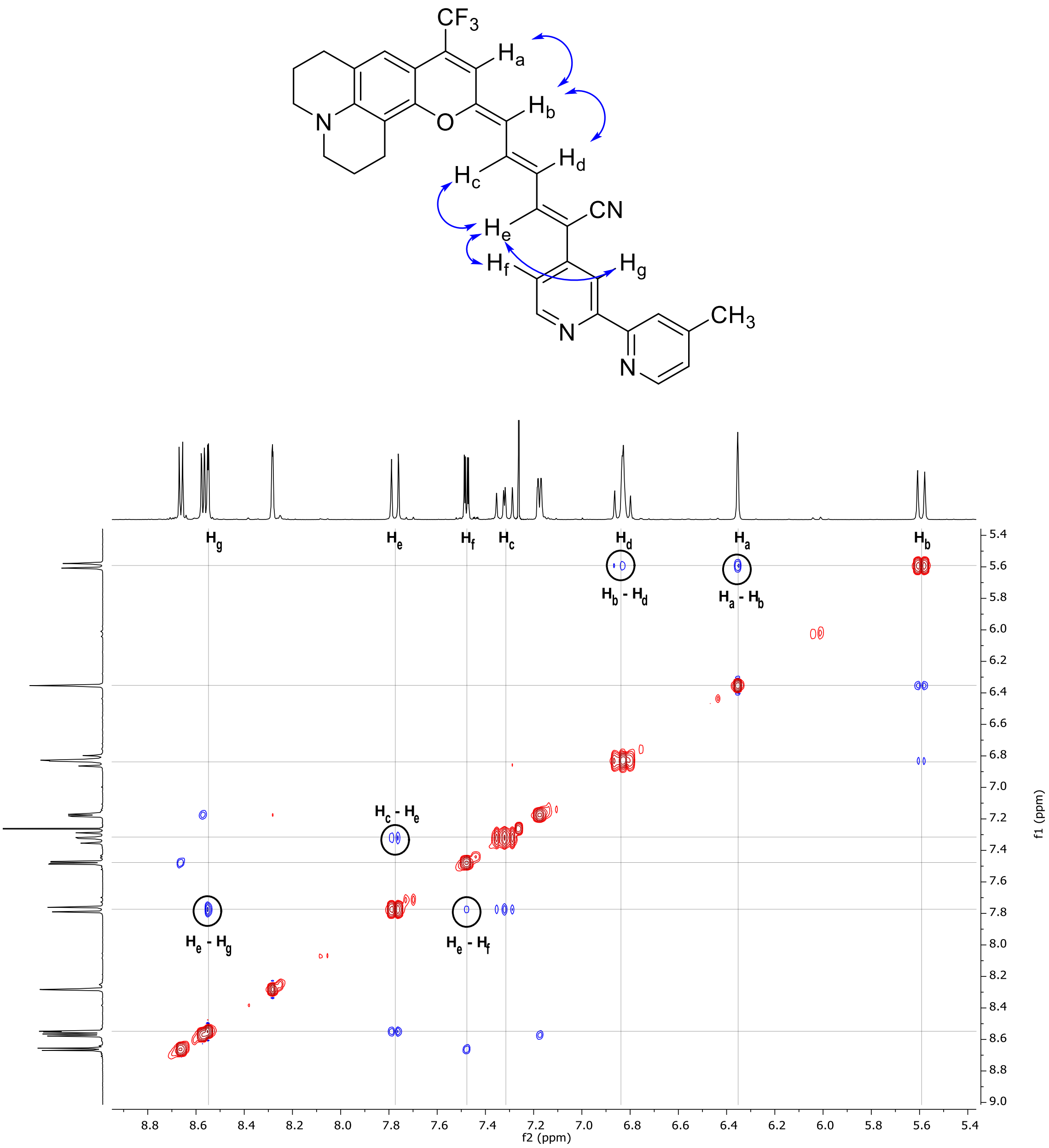

Figure S4. Expansion of the 2D NOESY spectrum of COUBPY ligand **7** in $CDCl_3$ showing diagnostic NOE cross-peaks.

1.4. Synthesis of Ru complex 12 and of Ru-COUBPY complexes Ru4-7

Complex 12:

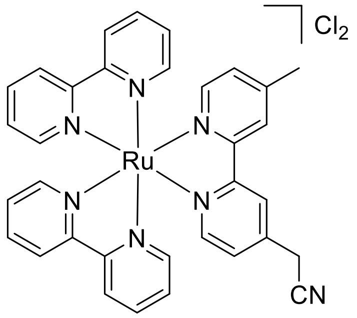

[Ru(bpy)$_2$Cl$_2$] (72 mg, 0.14 mmol) and 2-(4'-methyl-[2,2'-bipyridin]-4-yl) acetonitrile (31 mg, 0.14 mmol) were dissolved in EtOH (8 mL), and the resulting reaction mixture was stirred at 80 ºC overnight. After evaporation to dryness under reduced pressure, the compound was purified by silica column chromatography with ACN and increasing the eluent polarity with 0.1 M KNO$_3$ first (0-8%). Then, compound was re-dissolved in MeOH (5 mL) and treated with Amberlite IRA-410 chloride form in MeOH for 16 h. 95 mg of an orange solid were obtained after filtration (yield: 92 %).

^{1}H NMR (500 MHz, CD$_3$OD) δ (ppm): 8.69 – 8.66 (m, 5H), 8.58 – 8.57 (m, 1H), 8.09 (tt, *J* = 8.0, 1.3 Hz, 4H), 7.83 (ddd, *J* = 5.6, 1.6, 0.7 Hz, 1H), 7.80 – 7.76 (m, 4H), 7.60 (d, *J* = 5.8 Hz, 1H), 7.50 – 7.41 (m, 5H), 7.33 (ddd, *J* = 5.8, 1.8, 0.8 Hz, 1H), 3.31 (s, 2H), 2.56 (s, 3H).

^{13}C NMR (101 MHz, CD$_3$OD) δ (ppm): 157.6, 157.2, 157.2, 157.2, 157.1, 156.1, 151.5, 151.3, 151.2, 150.9, 150.4, 143.1, 137.8, 128.7, 127.5, 127.5, 126.7, 125.4, 124.3, 124.2, 124.2, 123.7, 116.2, 29.4, 19.8.

HR-ESI MS (ESI): *m/z* 311.5696 calc. for [$C_{33}H_{27}N_7Ru$]$^{2+}$: 311.5680

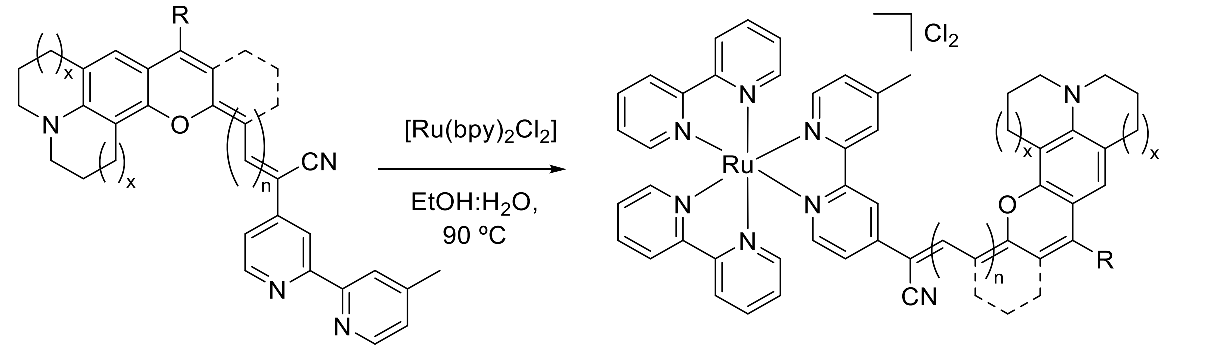

Scheme S4. General scheme for the synthesis of Ru-COUBPY complexes by direct ligand assembly.

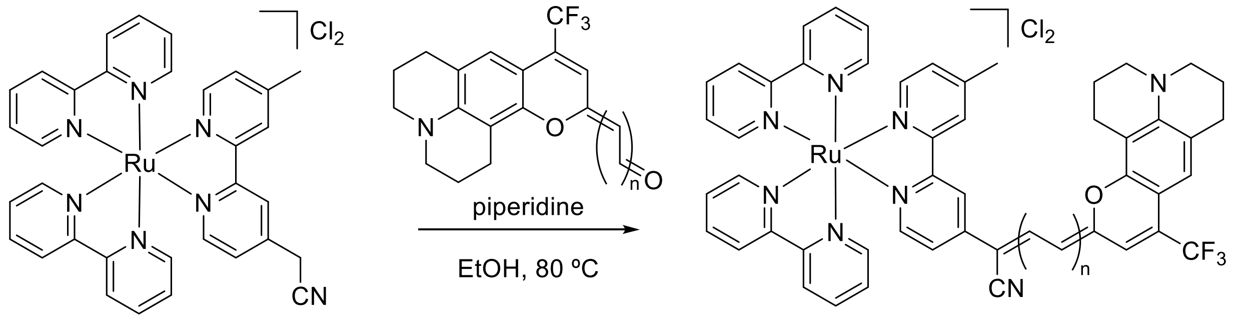

Scheme S5. General scheme for the synthesis of Ru-COUBPY complexes by post-coordination ligand assembly.

Ru4

COUBPY ligand **4** (22 mg, 0.049 mmol) and [Ru(bpy)$_2$Cl$_2$] (25 mg, 0.051 mmol) were dissolved in a 1:1 (v/v) solution of EtOH/H_2O (4 mL) and the reaction mixture was stirred for 48 h at 90 ºC under an Ar atmosphere. The solvent was evaporated to dryness and the product was purified by neutral aluminum oxide column starting with DCM and increasing the eluent polarity with MeOH (0-4%). 37 mg of a dark blue solid were obtained (yield: 88%), identified as Ru-COUBPY complex **Ru4**.

^{1}H NMR (400 MHz, CD_3OD) δ (ppm) (major rotamer): 8.75 – 8.70 (m, 4H), 8.65 (s, 1H), 8.59 (s, 1H), 8.53 (d, *J* = 12.5 Hz, 1H), 8.13 (t, *J* = 7.8 Hz, 4H), 7.96 (d, *J* = 5.4 Hz, 1H), 7.87 (d, *J* = 5.5 Hz, 1H), 7.86 – 7.80 (m, 2H), 7.62 (d, *J* = 5.9 Hz, 1H), 7.59 – 7.46 (m, 6H), 7.43 (d, *J* = 8.8 Hz, 1H), 7.34 (d, *J* = 6.7 Hz, 1H), 6.74 (d, *J* = 2.5 Hz, 1H), 6.70 (dd, *J* = 8.9, 2.3 Hz, 1H), 6.33 (s, 1H), 5.88 (d, *J* = 12.4 Hz, 1H), 3.54 – 3.44 (m, 4H), 2.59 (s, 3H), 2.31 (s, 3H), 1.23 (t, *J* = 7.0 Hz, 6H).

^{13}C NMR (101 MHz, CD_3OD) δ (ppm): 169.3, 166.6, 163.9, 158.7, 158.6, 158.2, 158.1, 156.8, 156.5, 152.6, 152.6, 152.1, 151.6, 151.4, 147.3, 147.0, 146.6, 144.3, 142.7, 139.0, 133.6, 132.4, 129.9, 129.8, 128.9, 126.9, 126.4, 125.6, 122.5, 122.1, 119.2, 118.8, 118.4, 115.2, 112.6, 112.3, 110.7, 103.0, 101.9, 98.0, 97.9, 95.2, 94.9, 45.7, 21.3, 18.3, 12.9.

HR-ESI MS (ESI): *m/z* 431.1344 calc. for $[C_{49}H_{44}N_8ORu]^{2+}$: 431.1336

Ru5

COUBPY ligand **5** (15 mg, 0.032 mmol) and [Ru(bpy)$_2$Cl$_2$] (17 mg, 0.035 mmol) were dissolved in a 1:1 (v/v) solution of EtOH/H_2O (5 mL) and the reaction mixture was stirred overnight at 90 ºC. The solvent was evaporated to dryness and the product was purified by neutral aluminum oxide column starting with DCM and increasing the eluent polarity with MeOH (0-6%). 21 mg of a dark green solid were obtained (yield: 74%), identified as Ru-COUBPY complex **Ru5**.

^{1}H NMR (400 MHz, CD_3OD) δ (ppm): 8.66 – 8.60 (m, 4H), 8.52 (t, *J* = 1.4 Hz, 1H), 8.47 (s, 1H), 8.33 (s, 1H), 8.07 – 8.00 (m, 4H), 7.85 (d, *J* = 5.6 Hz, 1H), 7.78 (d, *J* = 5.3 Hz, 1H), 7.73 (d, *J* = 5.5 Hz, 2H), 7.54 – 7.52 (m, 3H), 7.45 – 7.37 (m, 4H), 7.25 (d, *J* = 5.8 Hz, 2H), 7.04 (d, *J* = 8.7 Hz, 1H), 6.81 (s, 1H), 6.54 (d, *J* = 2.4 Hz, 1H), 6.51 (dd, *J* = 8.7, 2.5 Hz, 1H), 3.36 (q, *J* = 7.0 Hz, 4H), 2.87 (d, *J* = 6.1 Hz, 2H), 2.49 (t, *J* = 7.0 Hz, 2H), 2.47 (s, 3H), 1.71 (p, *J* = 6.3 Hz, 2H), 1.10 (t, *J* = 7.1 Hz, 6H).

^{13}C NMR (101 MHz, CD_3OD) δ (ppm): 159.2, 157.3, 157.2, 156.8, 156.7, 155.2, 151.2, 151.2, 150.6, 150.4, 150.3, 150.2, 146.9, 139.7, 137.7, 131.0, 128.4, 128.0, 127.5, 127.5, 124.9, 124.2, 122.9, 121.3, 119.3, 118.4, 111.1, 109.2, 109.1, 96.4, 93.4, 44.2, 28.7, 26.0, 21.0, 20.0, 11.6.

HR-ESI MS (ESI): *m/z* 444.1420 calc. for $[C_{51}H_{44}N_8ORu]^{2+}$: 444.1414

Ru6

Procedure 1 (direct ligand assembly):

COUBPY ligand **6** (31.6 mg, 0.060 mmol) and [Ru(bpy)$_2$Cl$_2$] (37.4 mg, 0.077 mmol) were dissolved in a 3:1 (v/v) solution of EtOH/H_2O (2 mL) and the reaction mixture was stirred overnight at 90 ºC. The solvent was evaporated to dryness and the product was purified by silica column chromatography starting with hexanes and increasing the eluent polarity with DCM first (0-100%) and then with MeOH (0-20%). 10 mg of a blue solid were obtained (yield: 17%), identified as Ru-COUBPY complex **Ru6**.

Procedure 2 (post-coordination ligand assembly):

To a solution of the coumarin aldehyde **10** (10.7 mg, 0.032 mmol) in absolute ethanol (8 mL), piperidine (16 mg, 0.192 mmol) and complex **12** (22.3 mg, 0.032 mmol) were added. The reaction mixture was stirred at 80 ºC overnight. After evaporation to dryness under reduced pressure, the compound was purified by silica column chromatography starting with hexanes and increasing the eluent polarity with DCM first (0-100%) and then with MeOH (0-20%). 32.0 mg of a blue solid were obtained (yield: 97%), identified as Ru-COUBPY complex **Ru6**.

TLC: Rf (30% MeOH in DCM) 0.3.

^{1}H NMR (400 MHz, CD_3OD) δ (ppm) *Z,Z*-isomer: 8.76 – 8.69 (m, 4H), 8.64 (d, *J* = 2.1 Hz, 1H), 8.56 (d, *J* = 1.7 Hz, 1H), 8.37 (d, *J* = 12.3 Hz, 1H), 8.17 – 8.10 (m, 4H), 7.97 – 7.91 (m, 1H), 7.88 – 7.85 (m, 1H), 7.84 – 7.80 (m, 2H), 7.70 (d, *J* = 6.2 Hz, 1H), 7.64 (d, *J* = 5.8 Hz, 1H), 7.57 – 7.47 (m, 5H), 7.39 – 7.35 (m, 1H), 6.92 (s, 1H), 6.75 (s, 1H), 6.14 (d, *J* = 12.3 Hz, 1H), 3.36 – 3.33 (m, 4H), 3.01 – 2.78 (m, 2H), 2.75 – 2.70 (m, 2H), 2.61 (s, 3H), 2.08 – 1.88 (m, 4H).

^{13}C NMR (101 MHz, CD_3OD) δ (ppm): 157.8, 156.6, 156.6, 156.5, 155.8, 151.2, 151.1, 150.3, 149.8, 149.7, 146.2, 143.0, 140.1, 137.8, 128.8, 127.9, 125.4, 124.5, 124.2 (q, *J* = 256 Hz), 122.2, 121.1, 118.7, 118.5, 116.7, 113.7 (q, *J* = 6 Hz), 106.7, 104.9, 103.1, 99.8, 54.9, 49.3, 48.8, 27.0, 20.7, 20.7, 20.1, 19.6.

Z,Z-isomer: -65.05 (s, 3F); Minor-isomer: -64.60 (s, 3F).

HR-ESI MS (ESI): *m/z* 470.1193 calc. for $[C_{51}H_{41}F_3N_8ORu]^{2+}$: 470.1199

Ru7

2-(4'-Methyl-[2,2'-bipyridin]-4-yl)acetonitrile (8 mg, 0.038 mmol) and [Ru(bpy)$_2$Cl$_2$] (19 mg, 0.038 mmol) were dissolved in EtOH (5 mL) and the reaction mixture was stirred overnight at 80 ºC. Then the coumarin aldehyde **11** (14 mg, 0.038 mmol) and piperidine (20 mg, 0.229 mmol) were added to the reaction mixture. The reaction mixture was stirred at 80 ºC overnight. The solvent was evaporated to dryness and the product was purified by neutral aluminum oxide column starting with DCM and increasing the eluent polarity with MeOH (0-5%). 19 mg of a dark green solid were obtained (yield: 48%), identified as Ru-COUBPY complex **Ru7**.

^{1}H NMR (400 MHz, CD$_3$OD) δ (ppm): 8.81 (d, J = 2.1 Hz, 1H), 8.78 (s, 1H), 8.74 – 8.70 (m, 5H), 8.30 (d, J = 11.7 Hz, 1H), 8.18 – 8.10 (m, 5H), 7.92 (d, J = 5.7 Hz, 1H), 7.85 (d, J = 5.7 Hz, 1H), 7.84 – 7.80 (m, 3H), 7.70 (d, J = 6.2 Hz, 1H), 7.63 (d, J = 5.8 Hz, 1H), 7.55 – 7.46 (m, 6H), 7.36 (d, J = 5.7 Hz, 1H), 6.85 (dd, J = 14.2, 11.8 Hz, 1H), 6.78 (s, 1H), 6.60 (s, 1H), 5.86 (d, J = 11.9 Hz, 1H), 3.30 – 3.27 (m, 4H), 2.91 (t, J = 6.5 Hz, 2H), 2.68 (t, J = 6.3 Hz, 2H), 2.63 (s, 3H), 2.04 (p, J = 6.0 Hz, 2H), 1.94 (p, J = 6.0 Hz, 2H).

^{13}C NMR (101 MHz, CD$_3$OD) δ (ppm): 157.2, 157.2, 157.1, 156.5, 154.9, 151.3, 151.2, 151.2, 151.0, 150.8, 150.3, 150.2, 148.1, 145.9, 143.8, 140.7, 137.8, 137.8, 128.6, 128.3 (q, J = 31.6 Hz), 127.5, 125.3, 124.9, 124.2, 123.2 (q, J = 273.7 Hz) 122.3, 121.4, 118.2, 118.0, 115.9, 114.4 (q, J = 6 Hz), 108.3, 106.8, 103.7, 102.6, 49.5, 49.0, 27.2, 21.2, 20.5, 20.4, 19.9.

^{19}F NMR (471 MHz, CDCl$_3$) δ (ppm): -65.15 (s, 3F).

HR-ESI MS (ESI): m/z 483.1282 calc. for $[C_{53}H_{43}F_3N_8ORu]^{2+}$: 483.1272

1.5. NOESY spectra of Ru-COUBPY complexes

Ru4

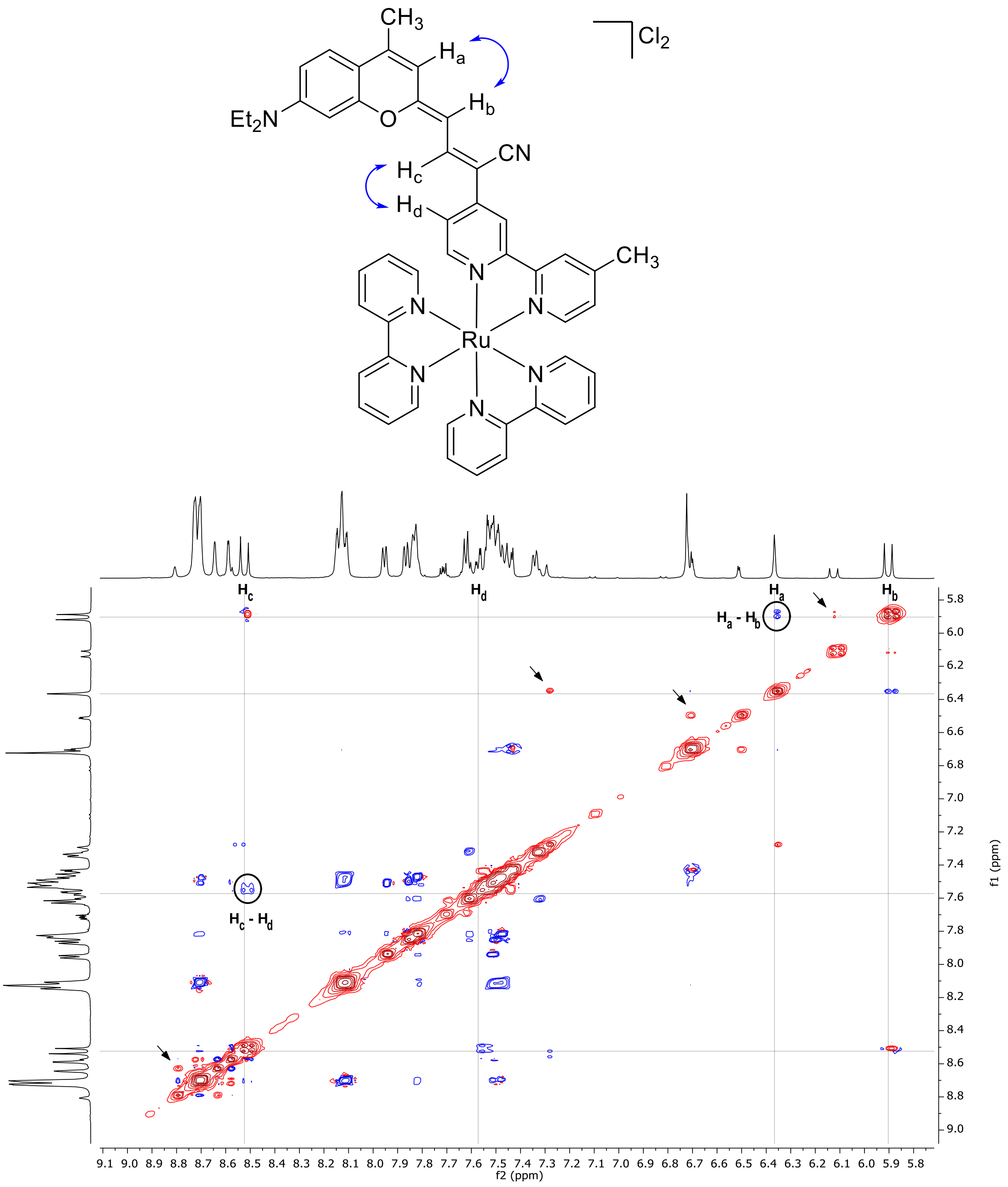

Figure S5. Expansion of the 2D NOESY spectrum of **Ru4** in CD_3OD showing diagnostic NOE cross-peaks and exchange cross-peaks between rotamer resonances of the same sign as the diagonal.

Ru5

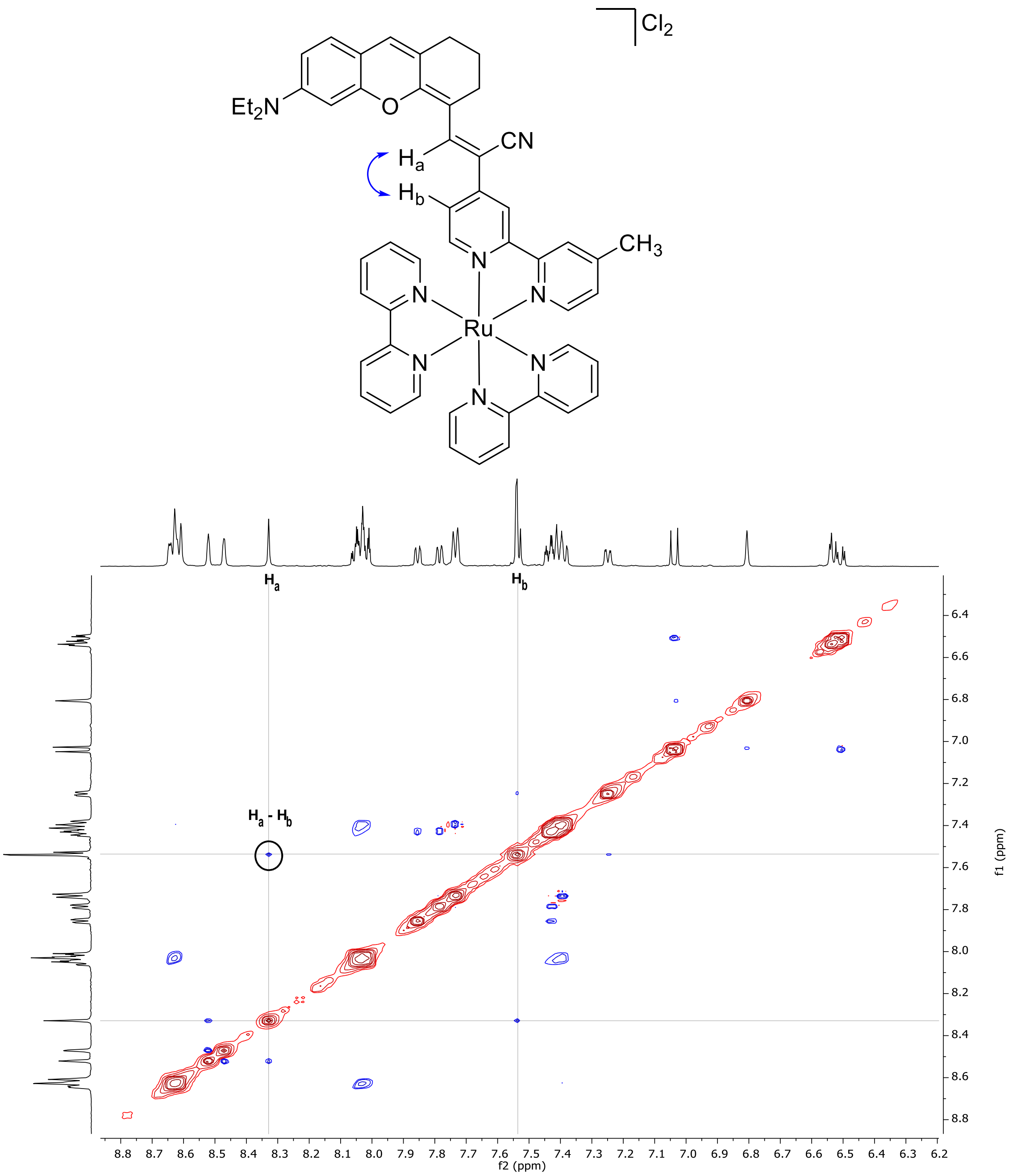

Figure S6. Expansion of the 2D NOESY spectrum of **Ru5** in CD_3OD showing diagnostic NOE cross-peaks.

Ru6

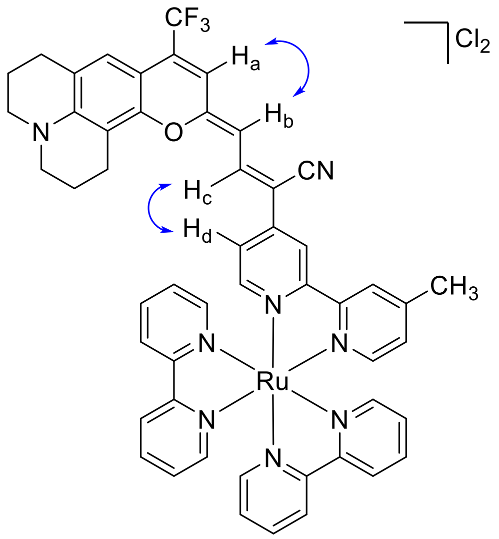

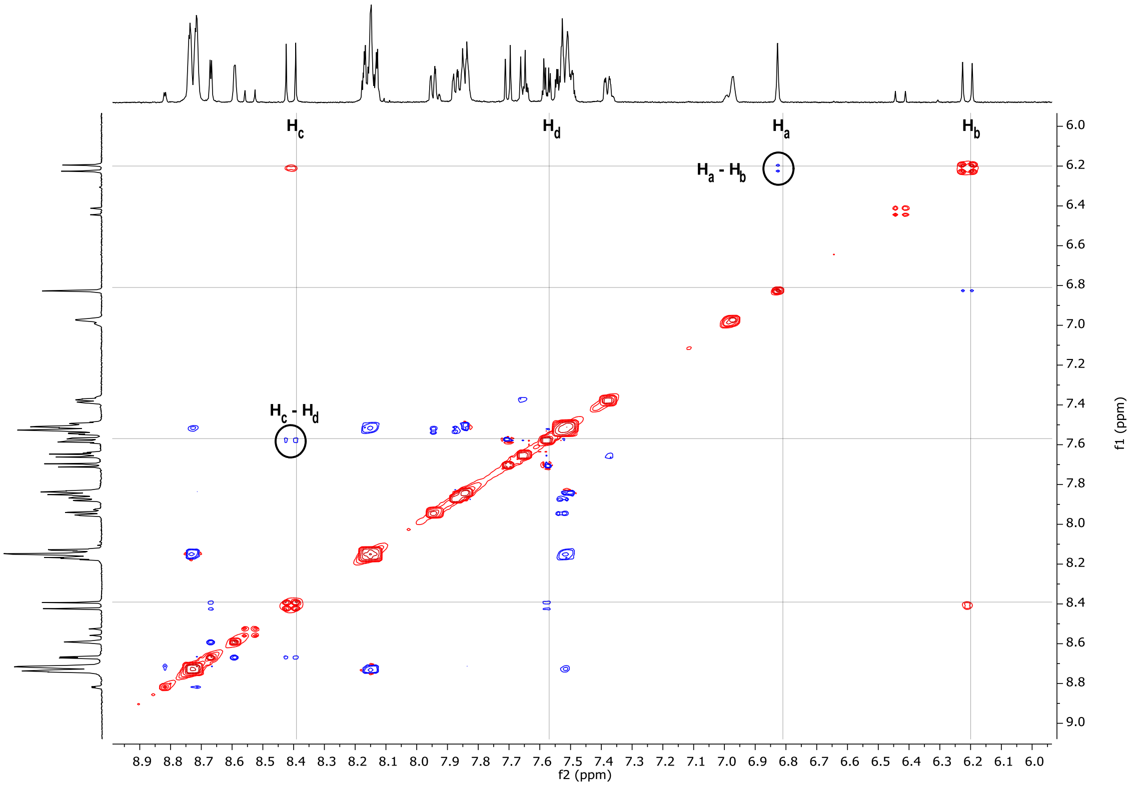

Figure S7. Expansion of the 2D NOESY spectrum of **Ru6** in CD_3OD showing diagnostic NOE cross-peaks.

Ru7

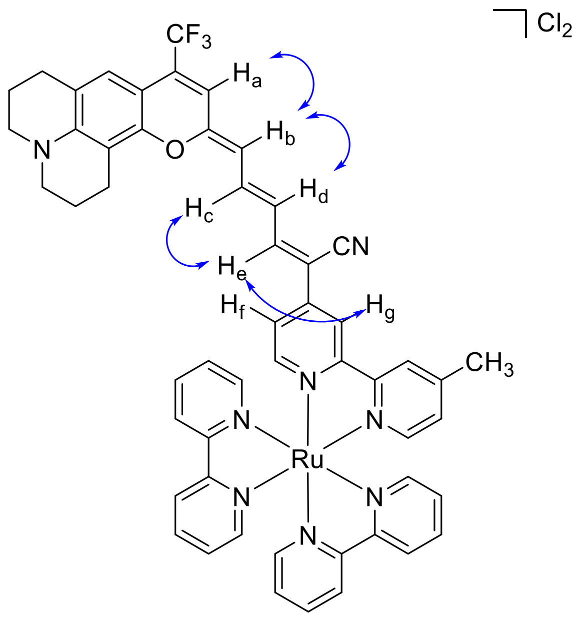

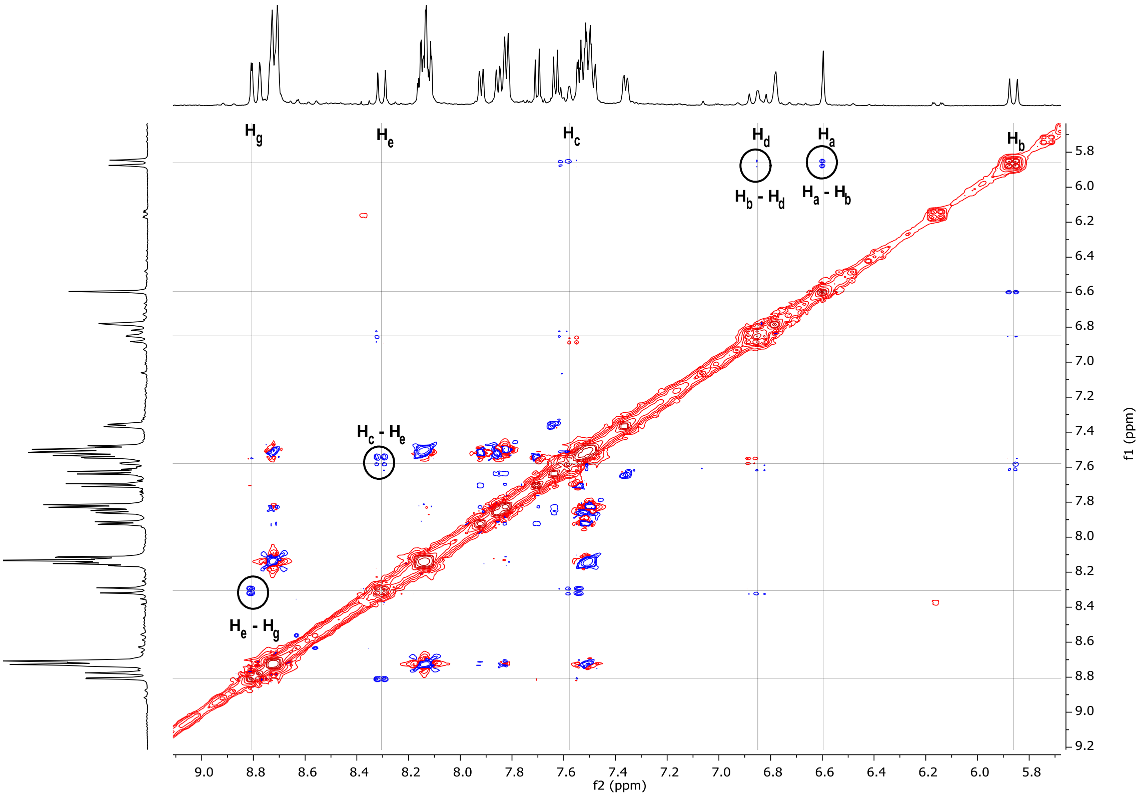

Figure S8. Expansion of the 2D NOESY spectrum of **Ru7** in CD_3OD showing diagnostic NOE cross-peaks.

1.6. HPLC analysis of Ru-COUBPY complexes

The HPLC analysis was performed in a Waters alliance 2695 Separations Module, comprised of a quaternary pump solvent delivery module, online degasser, auto sampler and a Waters 2996 photodiode array detector. HPLC separation was carried out using a Jupiter Proteo C12 column (150 x 4.6 mm, 90 Å, 4 µm) from Phenomenex. The mobile phase was a linear gradient beginning with 70:30 (v/v) A/B and ending with 0:100 (v/v) A/B over 15 min at a flow rate of 1 mL/min (A: 0.05% TFA in H_2O; B: 0.05% TFA in ACN). Control of the HPLC instrument, as well as processing of the chromatogram output (annotation of retention times, integration of peaks, calculation of peak areas) was carried out with MassLynx V4.1 software.

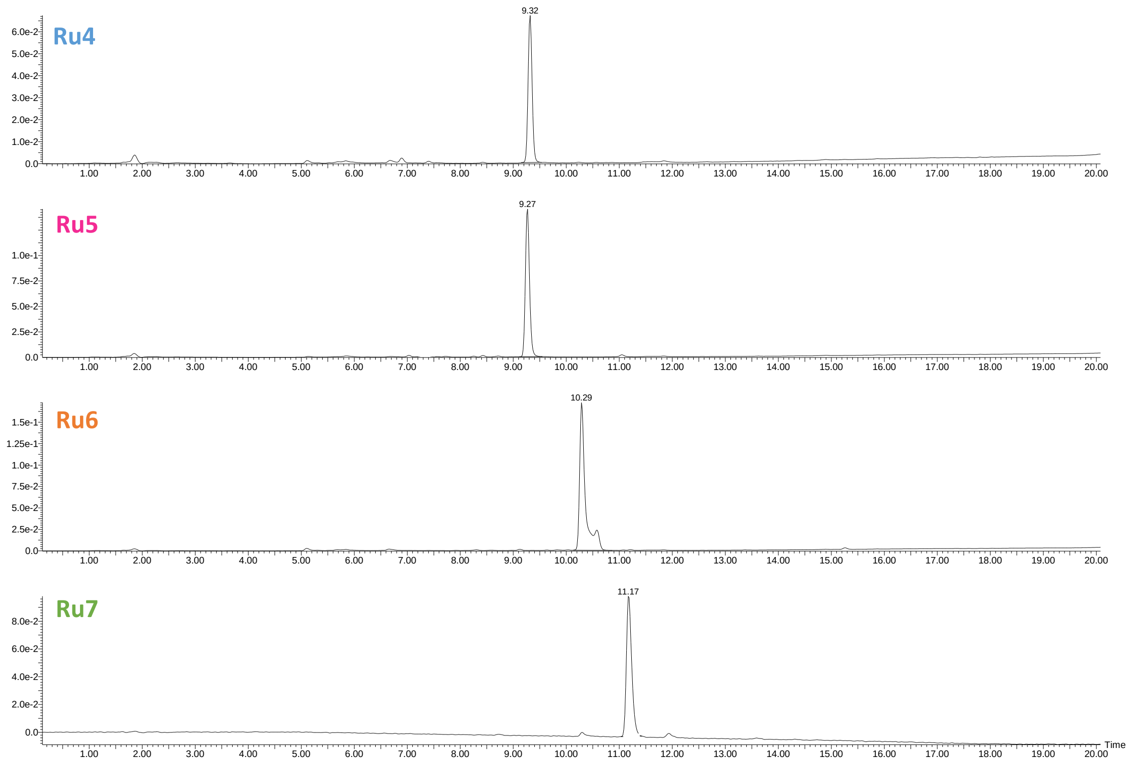

Figure S9. Reversed-phase HPLC chromatograms of Ru-COUBPY complexes **Ru4-7**.

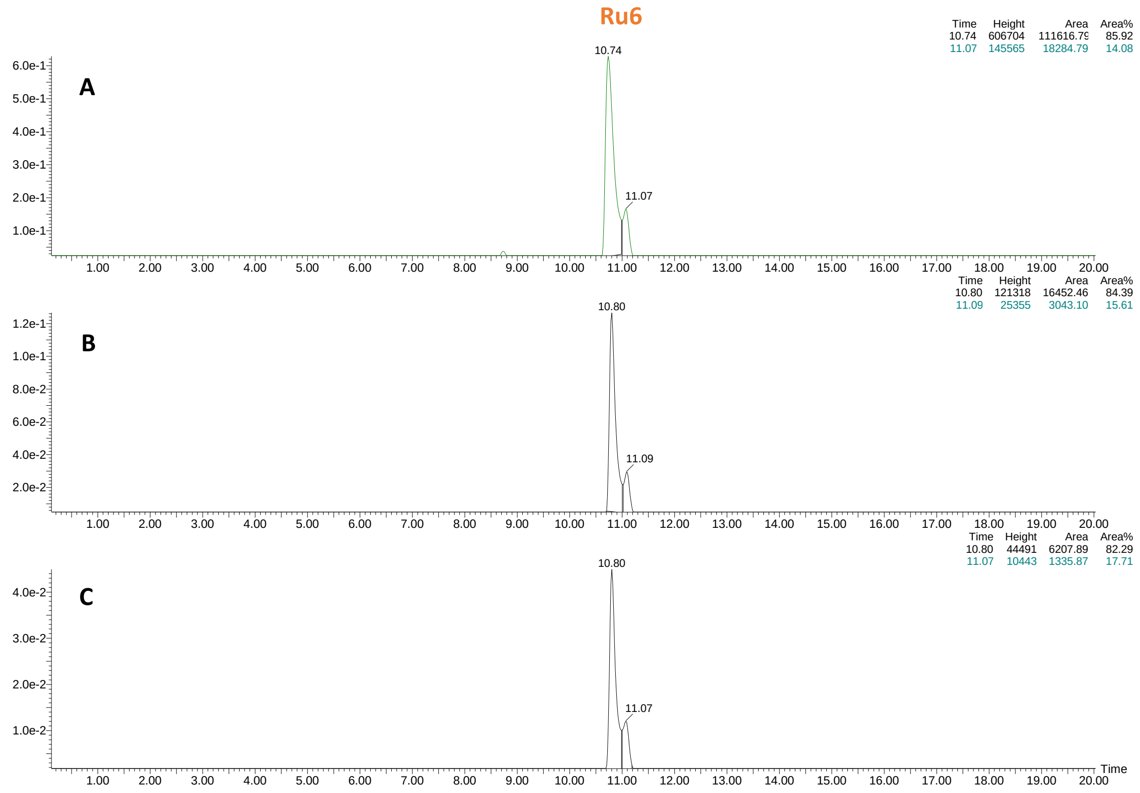

Figure S10. Reversed-phase HPLC analysis of the complex **Ru6**. (**A**) Chromatogram of the compound after purification using silica column chromatography. (**B**, **C**) Chromatograms of the two peaks collected independently from (A), with retention times of 10.74 minutes and 11.07 minutes, respectively. Each peak was re-injected separately, resulting in chromatograms identical to the original profile in (A), indicating an interconverting equilibrium between the two species.

1.7- Lipophilicity determination

Distribution coefficients between n-octanol and water ($K_{O/W}$) and the corresponding log P values for compounds **Ru4-7** were determined using the classical "shake-flask" method.[2] Working solutions (30 µM) were prepared by diluting a 10 mM DMSO stock into n-octanol-saturated Milli-Q water (7 mL total volume). For each compound, three 2 mL aliquots of the aqueous solution were transferred into centrifuge tubes. The remaining 1 mL of the aqueous solution was kept separately in a clean centrifuge tube and served as the reference sample. Each 2 mL aqueous aliquot was then mixed with an equal volume (2 mL) of Milli-Q water-saturated n-octanol. The mixtures were vigorously vortexed for 45 min to ensure partition equilibrium. Following equilibration, the biphasic systems were centrifuged at 6000 rpm for 10 min to achieve clear phase separation. The UV–Vis absorption spectra of the aqueous phases were recorded using a Jasco V-550 UV–Vis spectrophotometer and compared to that of the reference solution.

Log P values were calculated using the following equation:

$$\log P = \log(K_{O/W}) = \log\left(\frac{A0-A}{A}\right)$$

where A_0 is the absorbance of the aqueous reference solution (before extraction) at their maximum absorption wavelengths and A is the absorbance of the aqueous phase after partitioning is the absorbance of the aqueous phase of the corresponding octanol/water mixtures at the same wavelengths.

Table S1. Log P values of **Ru4-7** in octanol/water.

	**Ru4**	**Ru5**	**Ru6**	**Ru7**
Log P	**-0.49 ± 0.07**	**-0.55 ± 0.06**	**-0.65 ± 0.06**	**-0.34 ± 0.10**

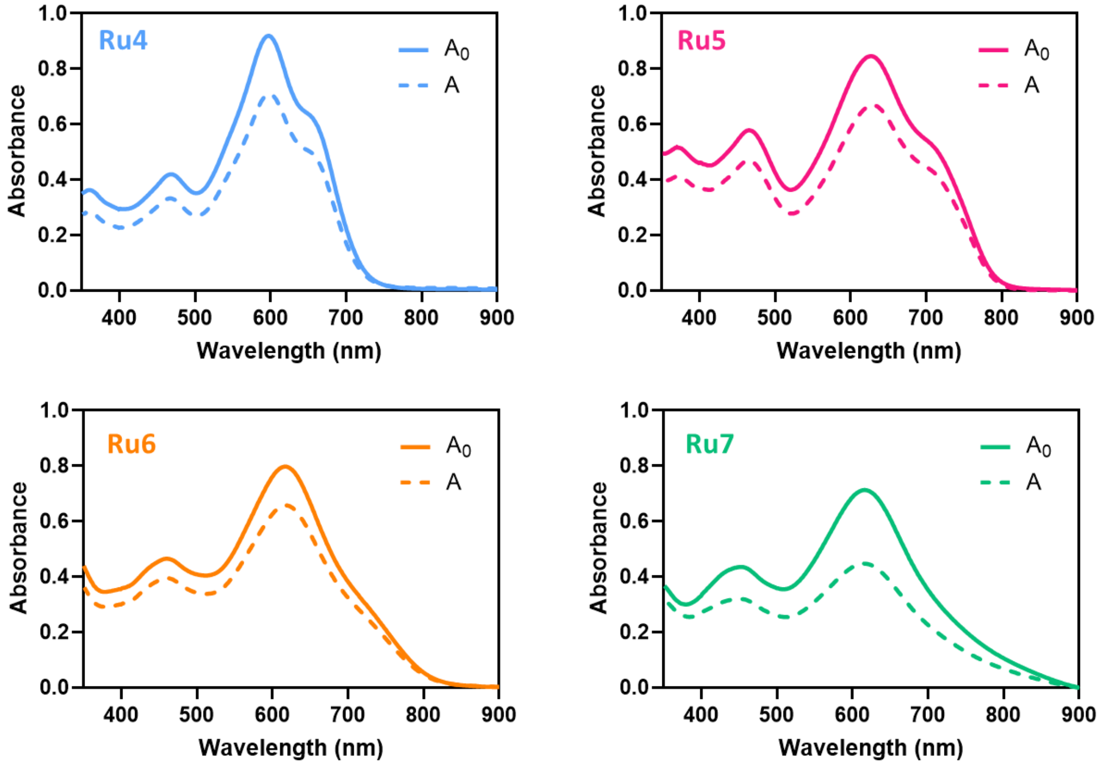

Figure S11. UV-Vis spectra of **Ru4-7** (30 μM) used for log *P* determination. The spectra of the reserved aliquots (A_0) are shown in solid lines, whereas the spectra of the aqueous phases of the O/W mixtures (A) are shown in dashed lines.

2.- Photophysical characterization: experimental and computational studies

2.1. Spectroscopic studies

Stock solutions of **Ru4-Ru7** complexes were prepared at a concentration of 1 mM in DMSO. These solutions were then diluted in ACN to achieve final concentrations ranging from 1 µM to 25 µM. UV-Vis absorption spectra were recorded at room temperature in the 550-750 nm range using 1 mL quartz cuvette with a 1 cm path length. For each compound, the absorbance at the maximum absorption wavelength (λ_{max}) was plotted as a function of concentration. The data were fitted using linear regression, and the slope of the resulting line corresponds to the molar absorption coefficient (ε), according to the Lambert–Beer Law ($A = \varepsilon \cdot c \cdot l$ (where A is the absorbance, c is the concentration ($mol \cdot L^{-1}$), and l is the path length (cm)).

Emission spectra were registered from solutions of the compounds (50 µM) in ACN using a Photon Technology International (PTI) fluorimeter. The excitation wavelengths were set to 460 nm, 650 nm and 720 nm. Emission spectra were recorded in the ranges of 470-900 nm, 660-900 nm and 730-900 nm respectively. The entrance and exit slits of the excitation and emission monochromators were set at 0.5 mm, providing a spectral bandwidth of 2 nm. The data interval was 1 nm and the integration time was 0.5 sec.

The fluorescence quantum yields (Φ_F) were determined using a comparative method. For this, the Ru-COUBPY complexes and reference standards, either Rho800 or ICG, were dissolved in ACN, EtOH, and DMSO, respectively. The solutions were prepared so that the absorbance at the excitation wavelength (λ_{ex}) ranged between 0.05 and 0.08 at the tabulated wavelength of 623 nm for Rho800 and 678 nm for ICG. Both the absorption and emission spectra for each solution were recorded in a 1 cm path length quartz cuvette. The test compounds and standards were analyzed under identical experimental conditions to ensure consistency. The integrated fluorescence intensity (i.e. the area under the curve of the emission spectrum) was calculated using GraphPad Prism version 10.00 for Windows (GraphPad Software Inc., La Jolla, CA, USA). Then, Φ_F was calculated using the following equation:

$$\phi F\ sample = \frac{A\ sample}{A\ ref} \cdot \frac{Abs\ ref}{Abs\ sample} \left(\frac{\eta\ sample}{\eta\ ref}\right)^2 \cdot \phi F\ ref$$

where Abs_{sample} and Abs_{ref} are the measured absorption of the sample at the excitation wavelength, A_{sample} and A_{ref} are the integrated fluorescence for the sample and the reference, and η_{sample} and η_{ref} are the refractive index of sample and reference solutions, respectively.

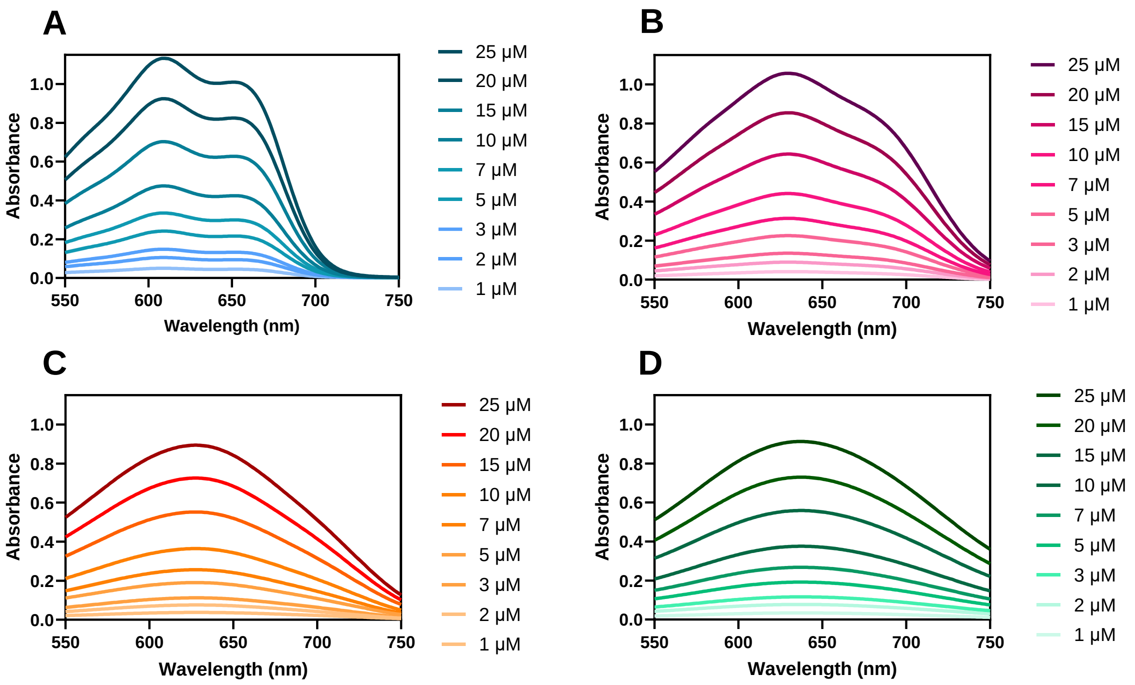

Figure S12. Absorption spectra of **Ru4-7** (A-D respectively) in ACN at concentrations ranging from 1 to 25 μM.

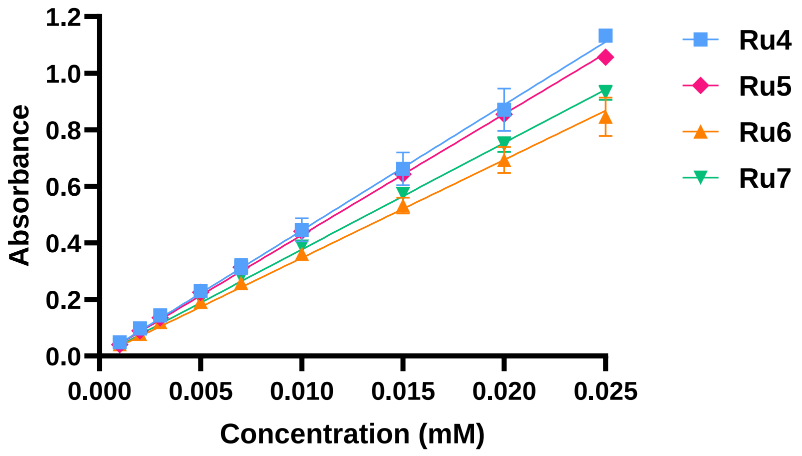

Figure S13. Absorbance at λ_{max} of π-extended Ru-COUBPY complexes **Ru4-7** in ACN plotted against concentration. The slope of the linear fit corresponds to the molar extinction coefficient (ε), according to Lambert-Beer Law.

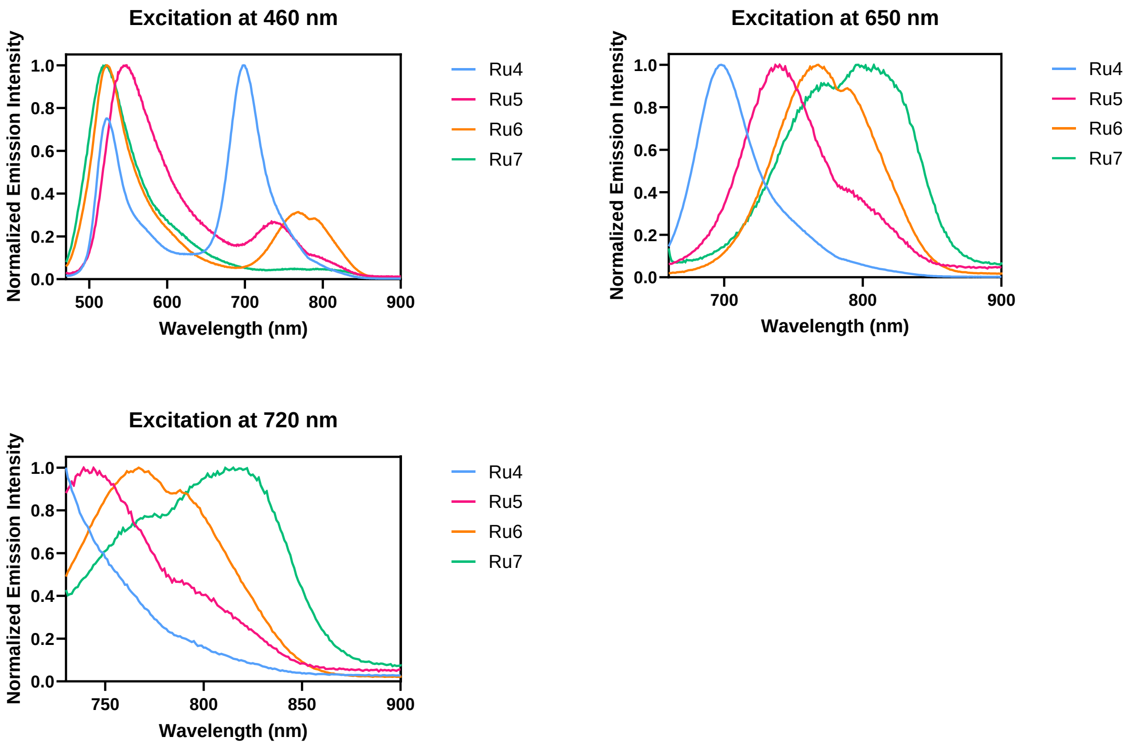

Figure S14. Normalized emission spectra of Ru-COUBPY complexes upon excitation at 460, 650 or 720 nm.

Table S2. Fluorescence quantum yield of Ru-COUBPY complexes.

	Compound			
Standard	**Ru4**	**Ru5**	**Ru6**	**Ru7**
**Rho800**	0.028	0.003	0.005	0.001
**ICG**	-	0.009	0.021	0.004

Time-resolved fluorescence measurements.

Solutions of the metal complexes (10 μM in ACN) were prepared and deoxygenated in a Schlenk flask using the freeze–pump–thaw technique. The deoxygenated solutions were then transferred to 1 cm quartz cuvettes equipped with Teflon septum screw caps and maintained under an inert atmosphere for all luminescence measurements. Time-resolved fluorescence measurements were performed using the time-correlated single-photon counting (TCSPC) technique on an FLS980 spectrometer (Edinburgh Instruments) equipped with a single-photon counting photomultiplier tube (PMT). Excitation was provided by picosecond pulsed diode lasers (EPL-405 nm and EPL-635 nm) operating at a repetition rate of 20 MHz (pulse period: 50 ns). Fluorescence decays were analyzed using reconvolution fitting, in which the measured decay curve was fitted using the experimentally determined IRF (instrument response function) from Ludox® scatter. Fit quality was assessed by the reduced chi-squared (χ^2) value and the autocorrelation of residuals using the FAST (Advanced Fluorescence Lifetime Analysis Software) program from Edinburgh Instruments. All measurements were conducted at 25.0 °C

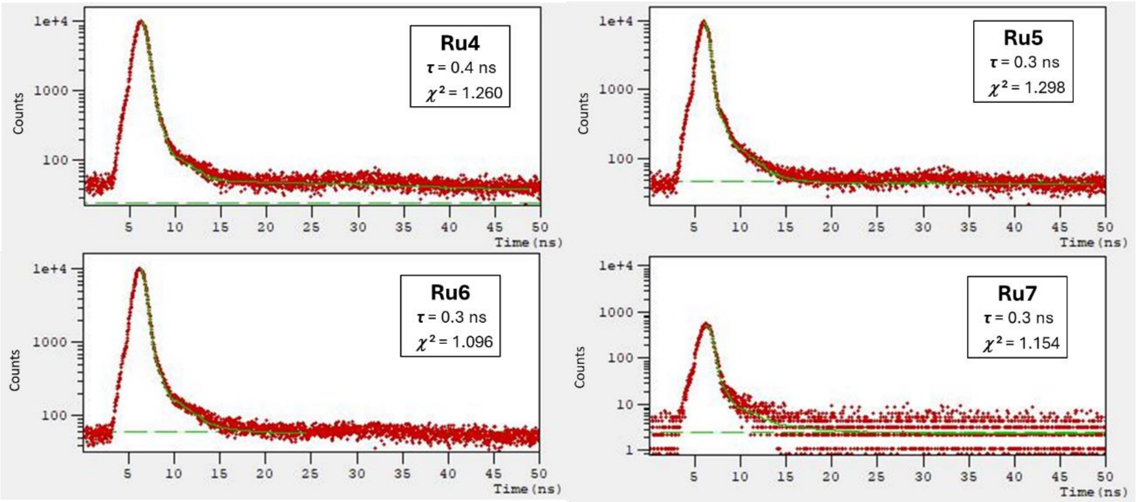

Figure S15. Time-resolved emission decays of **Ru4-7** under 635 nm laser excitation recorded at their emission maxima (698, 740, 768, and 797 nm, respectively).

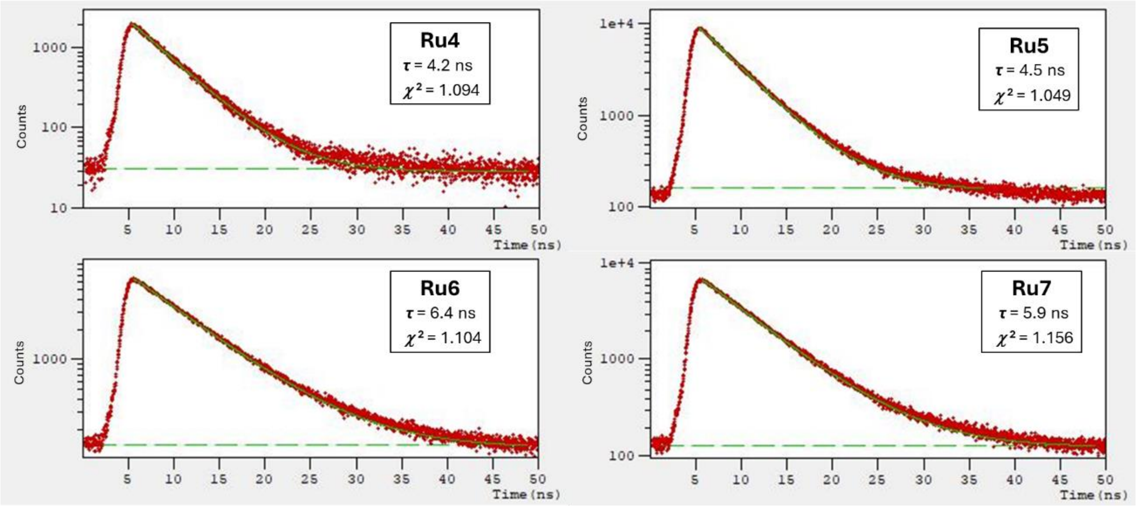

Figure S16. Time-resolved emission decays of **Ru4-7** under 405 nm laser excitation recorded at their emission maxima located around 520 nm.

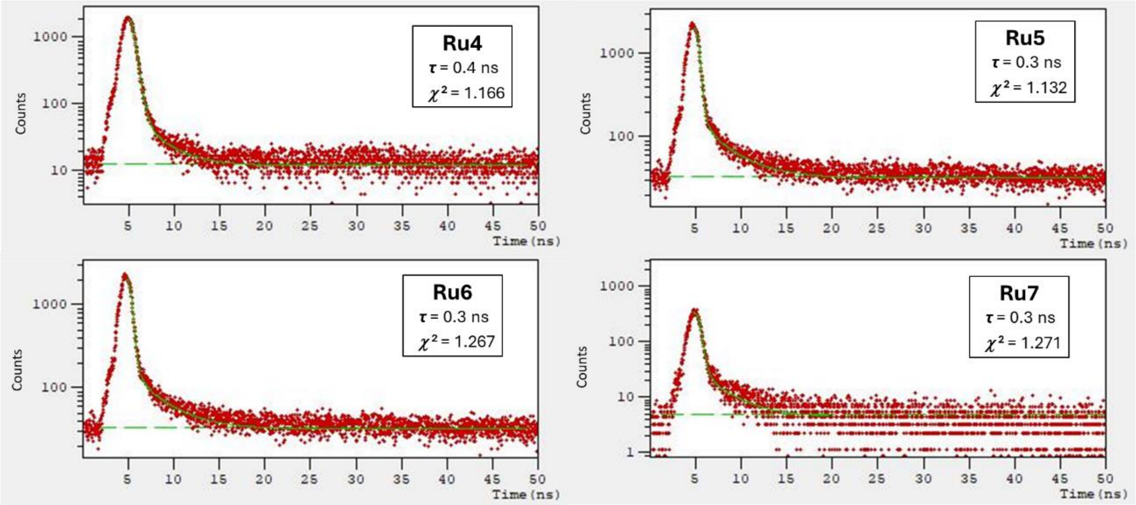

Figure S17. Time-resolved emission decays of **Ru4-7** under 405 nm laser excitation recorded at their emission maxima (698, 740, 768, and 797 nm, respectively).

2.2. Computational studies

All geometry optimizations were performed with the density functional theory (DFT) method as implemented in Gaussian 16[3] and without symmetry restrictions. The PBE0 functional[4] was used in combination with the 6-31+G(d,p) for all atoms except Ru, which was treated with the Stuttgart/Dresden pseudopotential (hereafter PBE0/6-31+G(d,p)/SDD). Frequency analysis on top of each minimized structure was performed to ensure the absence of any negative normal mode. Dispersion was corrected through the Grimme D3 method[5] and solvent effects (acetonitrile) were described with the conductor polarizable continuum model (CPCM) using the default settings.[6] Excited singlet and triplet states were computed on top of the PBE0/6-31+G(d,p)/SDD optimized geometries using the time-dependent DFT (TD-DFT) method, the Minnesota M06 functional,[7] and the same basis set, in coherence with previous computational studies of a variety of related Ru complexes.[8,9,10,11] The Tamm-Dancoff approximation[12] was used throughout the work. Natural transition orbitals (NTOs)[13] were computed with the Chemissian software[14] by post-processing the Gaussian 16 output.

Table S3. Energies (nm), oscillator strength (*f*) and Nature of the most relevant singlet-singlet transitions computed for the **Ru4-7** family at the TDDFT/M06/6-31+(d,p)/SDD level of theory.

	**High energy band**				**Mid-energy band**				**Low-energy band**			
**Complex**	**State**	**λ (nm)**	***f***	**Nature**	**State**	**λ (nm)**	***f***	**Nature**	**State**	**λ (nm)**	***f***	**Nature**
**Ru4**	S_{19}	370	0.103	Ru→bpy	S_5	489	0.136	Ru→bpy	S_3	524	0.162	Ru→bpy
					S_8	457	0.122	Ru→bpy	S_1	545	1.137	ILCT (COUBPY)
**Ru5**	S_{28}	341	0.261	ILCT (COUBPY)	S_8	459	0.079	Ru→bpy	S_1	567	1.083	ILCT (COUBPY)
					S_{11}	435	0.140	Ru→bpy				
**Ru6**					S_9	455	0.181	Ru→COUBPY	S_1	579	1.289	ILCT (COUBPY)
					S_{10}	452	0.118	Ru→bpy				
**Ru7**					S_9	469	0.265	Ru→COUBPY	S_1	618	1.990	ILCT (COUBPY)
					S_{10}	456	0.075	Ru→bpy				

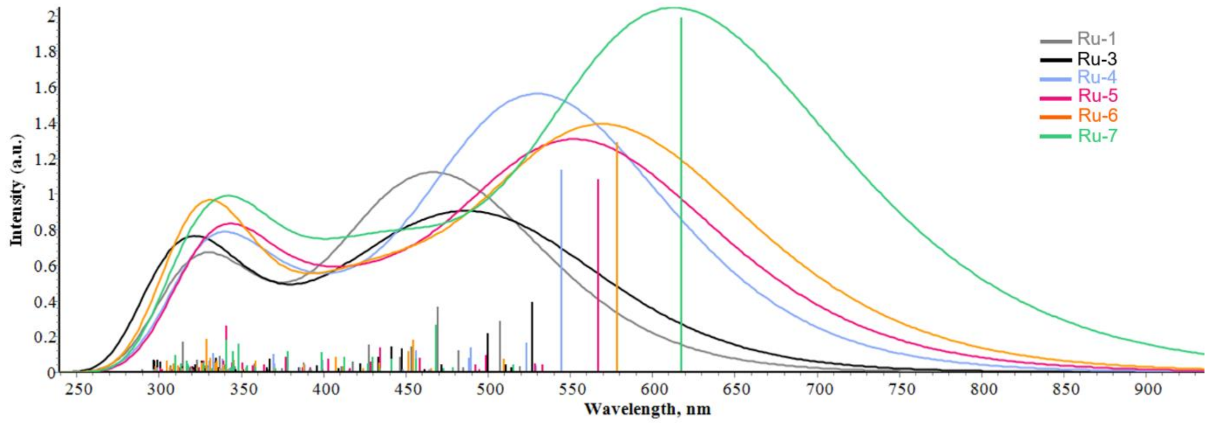

Figure S18. Absorption spectra for each π-extended Ru-COUBPY complex computed with the TDDFT/M06/6-31+G(d,p)/SDD method on top of the S_0 min geometry. Electronic transitions are shown as vertical lines, while spectral shapes are convoluted through Gaussian functions centered at each electronic excitation.

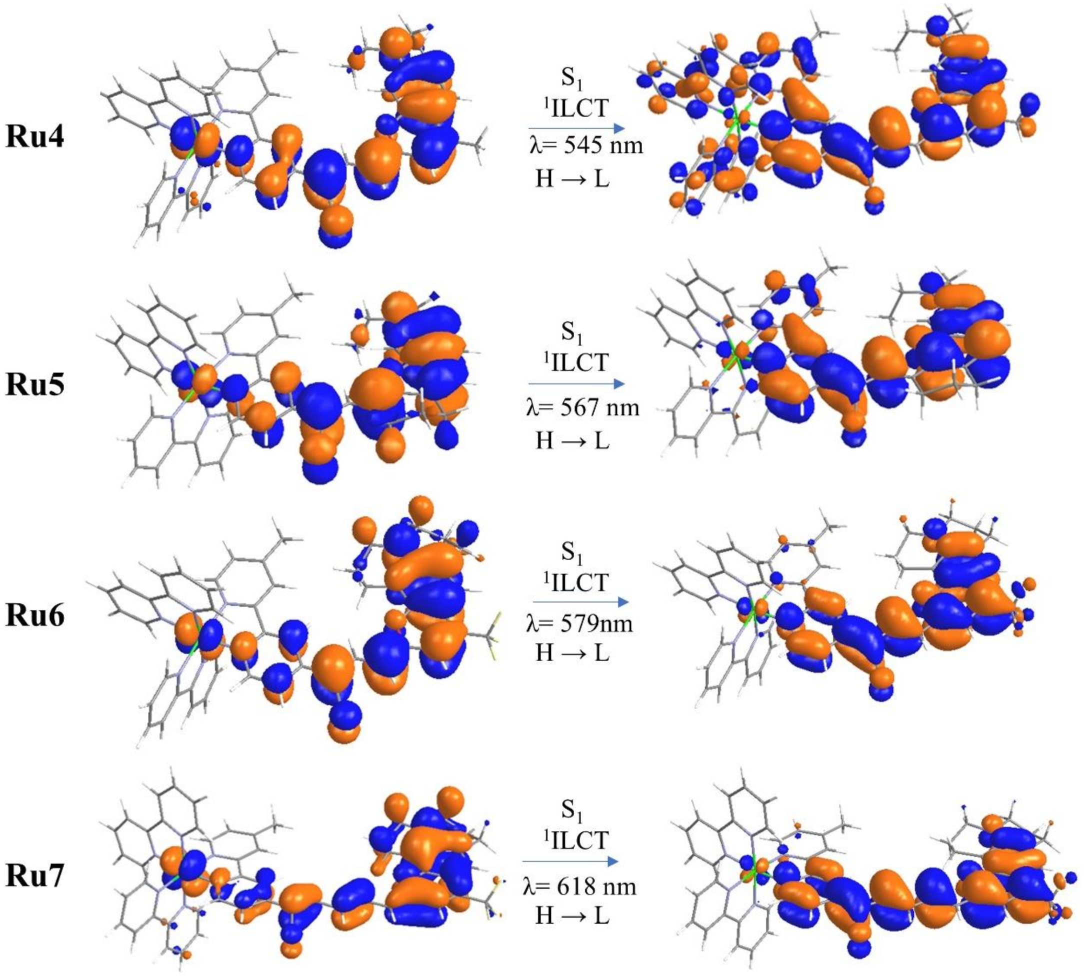

Figure S19. Natural transition orbitals (NTOs) that describe the first singlet excited state (S_1) for each π-extended Ru-COUBPY complex studied in this work (**Ru4-Ru7**). In all cases, the excitation corresponds to the HOMO→LUMO excitation of intra-ligand charge transfer (ILCT) character.

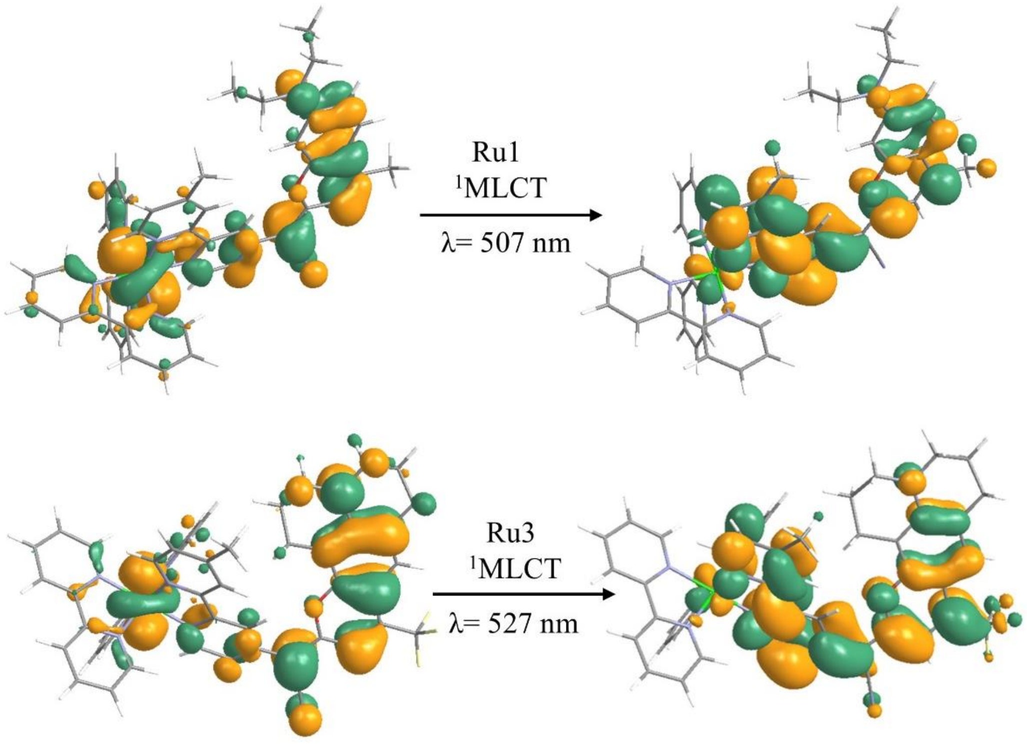

Figure S20. Natural transition orbitals (NTOs) for the lowest energy transitions computed for **Ru1** and **Ru3** parent compounds. Data retrieved with permission from Abad-Montero *et al*, *J. Am. Chem. Soc.* **2025**, *147*, 7360-7376 under a CC-BY 4.0 license.

Table S4. Calculated fragment contribution (Ru, COUBPY and BPY, in %) to frontier orbitals, for **Ru4-Ru7** series.

MO	Fragment	Ru4	Ru5	Ru6	Ru7
HOMO-1	Ru	43	46	43	42
	COUBPY	21	20	20	33
	BPY	36	35	37	25
HOMO	Ru	9	5	7	5
	COUBPY	69	71	73	63
	BPY	22	25	21	32
LUMO	Ru	4	4	5	2
	COUBPY	60	57	70	53
	BPY	36	39	25	45
LUMO+1	Ru	7	5	6	5
	COUBPY	25	42	40	34
	BPY	68	53	54	61

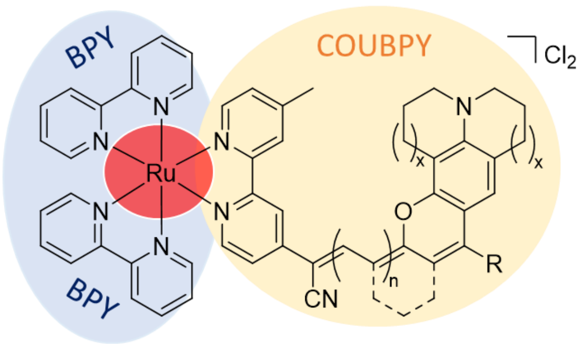

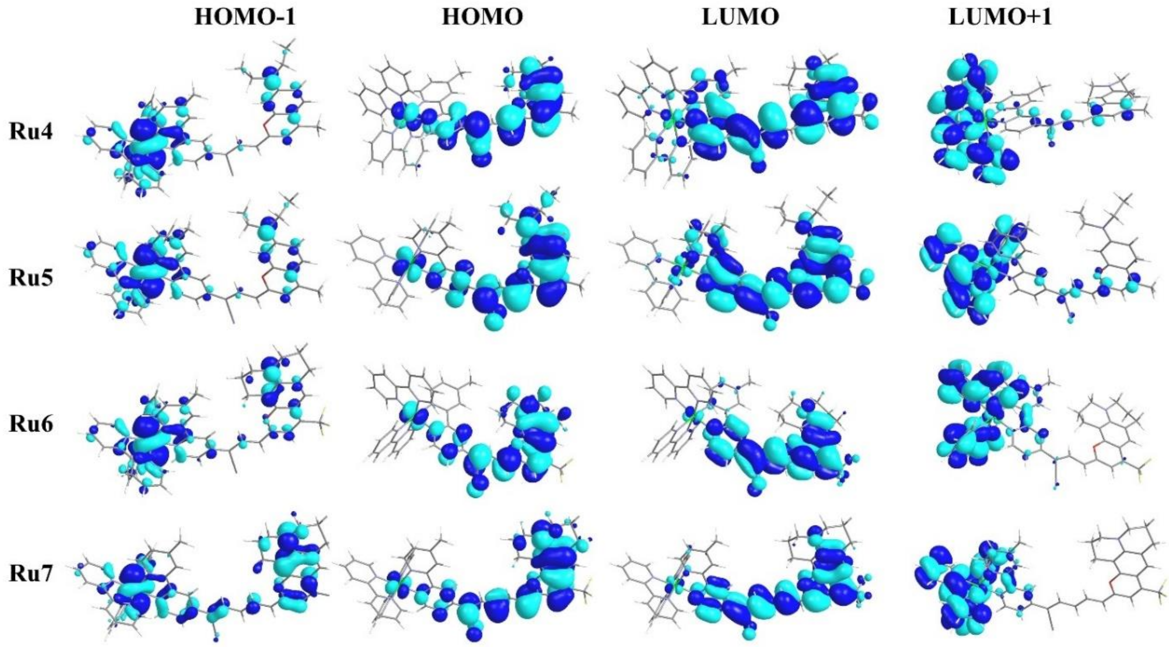

Figure S21. Metal-based HOMO-1, COUBPY-based HOMO and LUMO, and bpy-based LUMO+1 orbitals computed with the DFT/M06/6-31+G(d,p)/SDD method.

Ru4

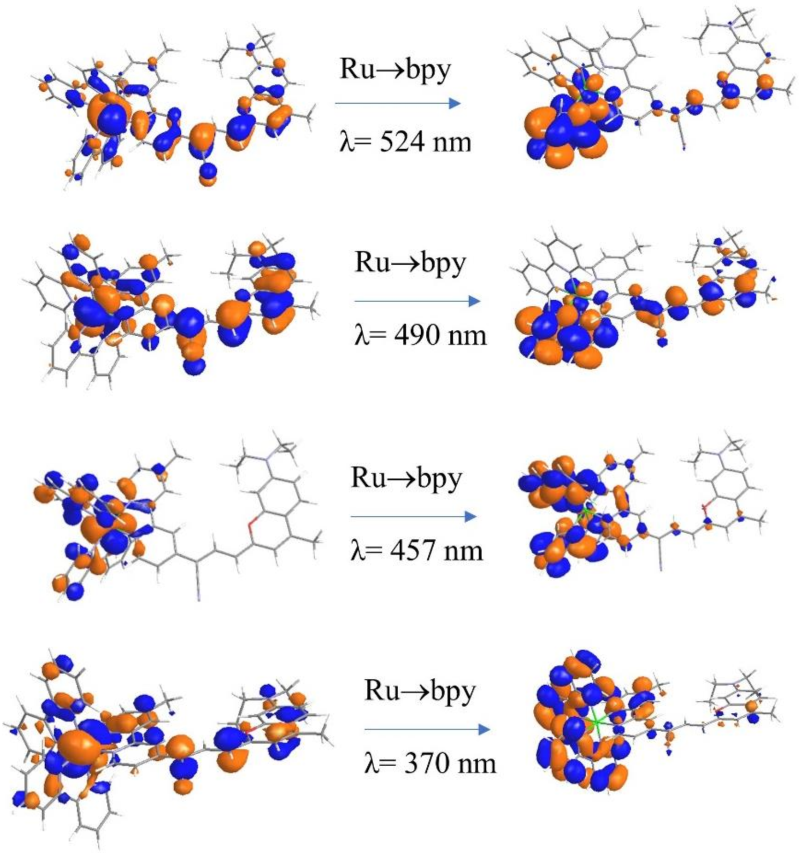

Figure S22. Natural Transition Orbitals of the main singlet-singlet transitions for **Ru4**.

Ru5

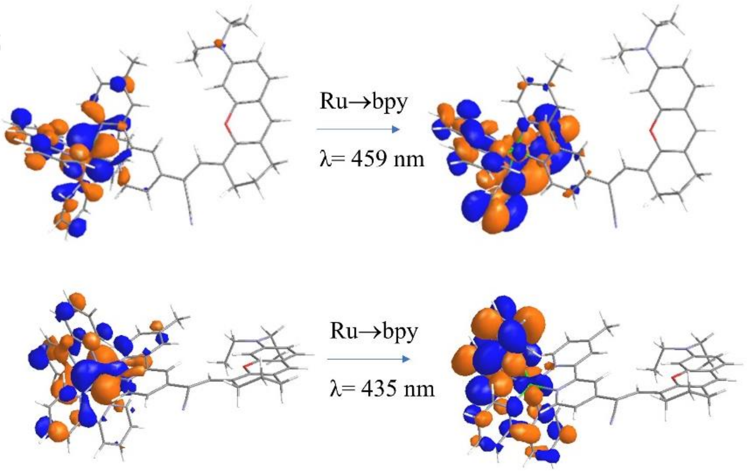

Figure S23. Natural Transition Orbitals of the main singlet-singlet transitions for **Ru5**.

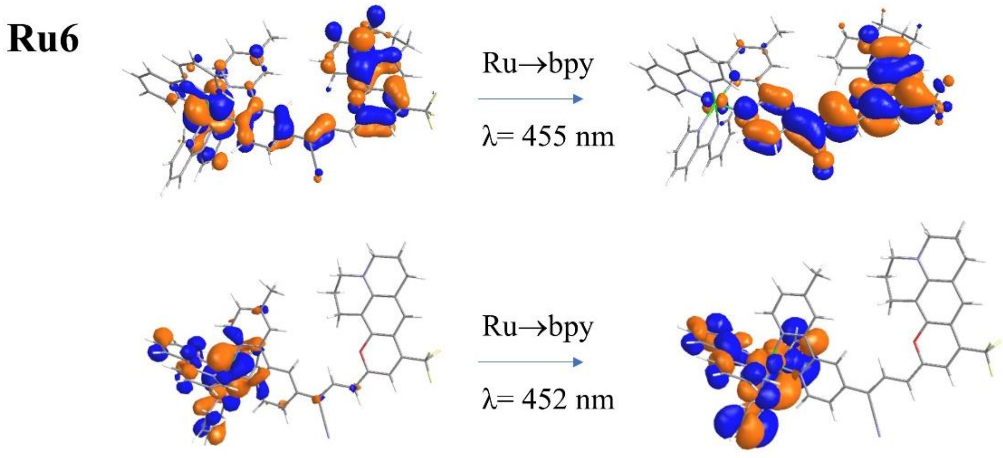

Figure S24. Natural Transition Orbitals of the main singlet-singlet transitions for **Ru6**.

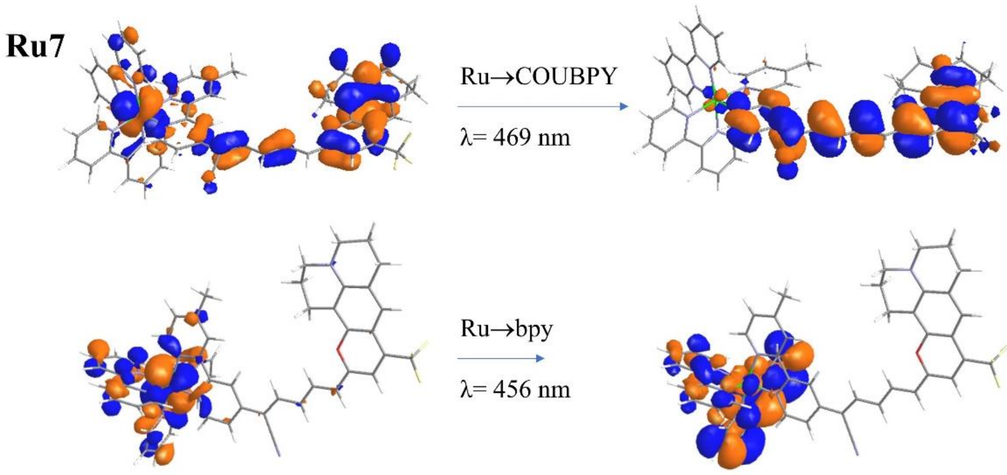

Figure S25. Natural Transition Orbitals of the main singlet-singlet transitions for **Ru7**.

3.- Dark stability and light stability studies in cell culture medium

3.1. Dark stability

For the dark stability studies, a solution of the compounds (30 µM) in DMEM cell culture medium supplemented with 10% FBS, 2 mM L-glutamine and 100 U·mL^{-1} of penicillin-streptomycin mixture, and containing 2,3,6,7-tetrahydro-1,1,7,7,9-pentamethyl-1*H*,5*H*,11*H*[1]benzopyrano-[6,7,8-*ij*]quinolizin-11-one (50 µM) as an internal standard, was incubated at 37 °C for 24 h. A 50 µL aliquot of the previous solutions was taken after the indicated time intervals (0, 2, 8 and 24 h) and analyzed by reversed-phase HPLC. The extent of degradation of the different compounds was evaluated by comparing the ratio between the peak area of investigated compound and that of the internal standard, before and after incubation for the indicated time interval.

The HPLC analysis was performed with a Waters alliance 2695 Separations Module, comprised of a quaternary pump solvent delivery module, online degasser, auto sampler and a Waters 2996 photodiode array detector. HPLC separation was carried out using a Jupiter Proteo C12 column (150 x 4.6 mm, 90 Å, 4 µm) from Phenomenex. The mobile phase was a linear gradient beginning with 70:30 (v/v) A/B and ending with 0:100 (v/v) A/B over 15 min at a flow rate of 1 mL/min (A: 0.05% TFA in H_2O; B: 0.05% TFA in ACN). The injection volume was 10 µL. Control of the HPLC instrument, as well as processing of the chromatogram output (annotation of retention times, integration of peaks, calculation of peak areas) was carried out with MassLynx V4.1 software.

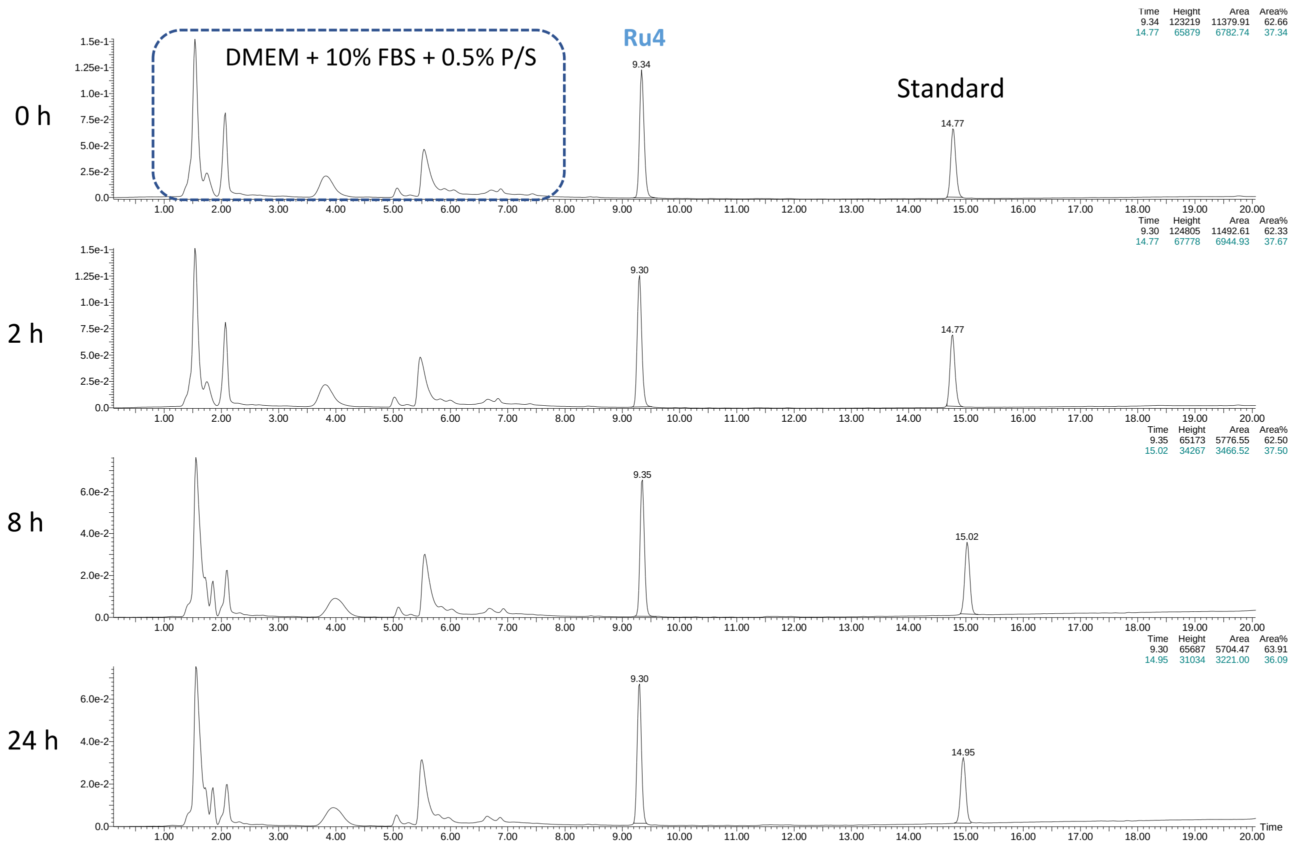

Figure S26. HPLC chromatograms showing the stability of **Ru4** in cell culture medium in the dark at 37 ºC after 0 h, 2 h, 8 h and 24 h.

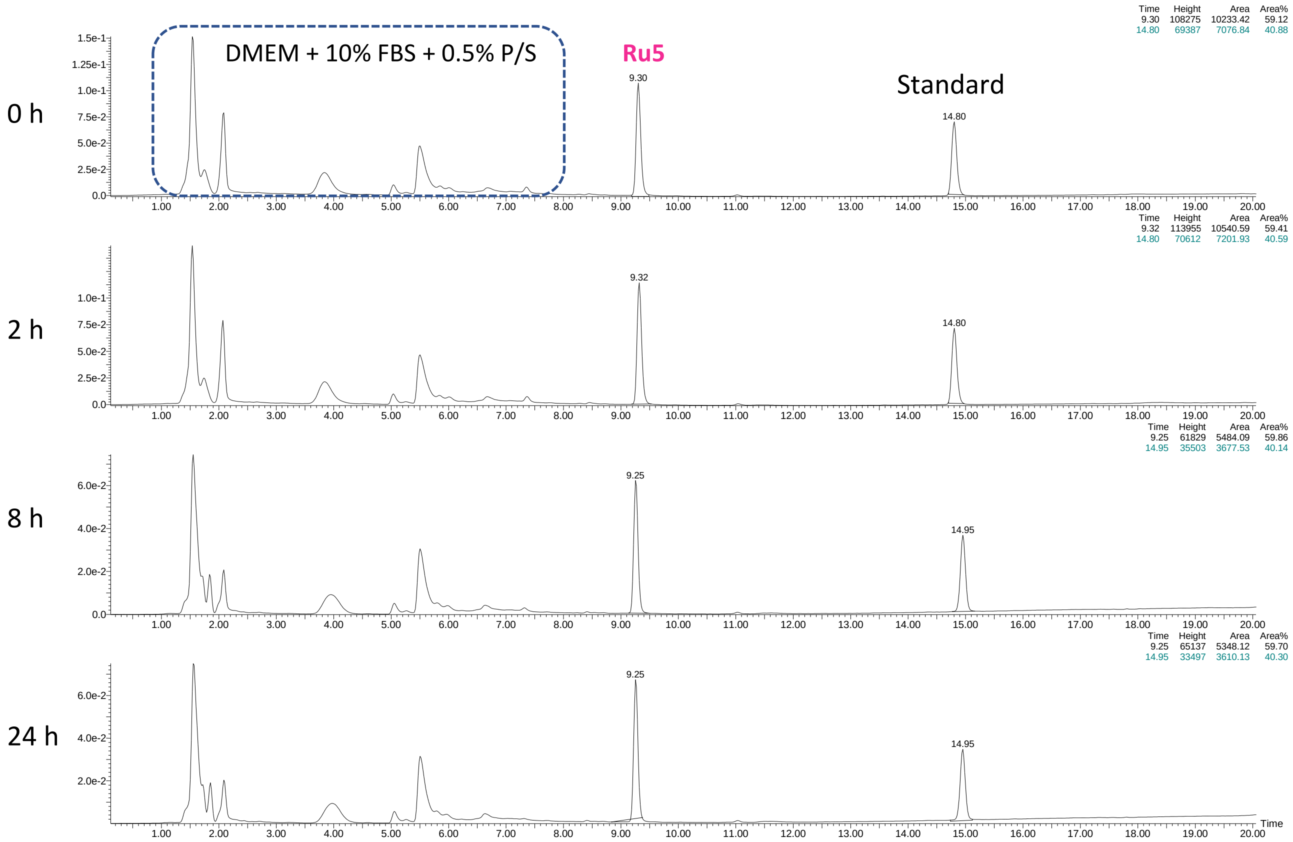

Figure S27 HPLC chromatograms showing the stability of **Ru5** in cell culture medium in the dark at 37 ºC after 0 h, 2 h, 8 h and 24 h.

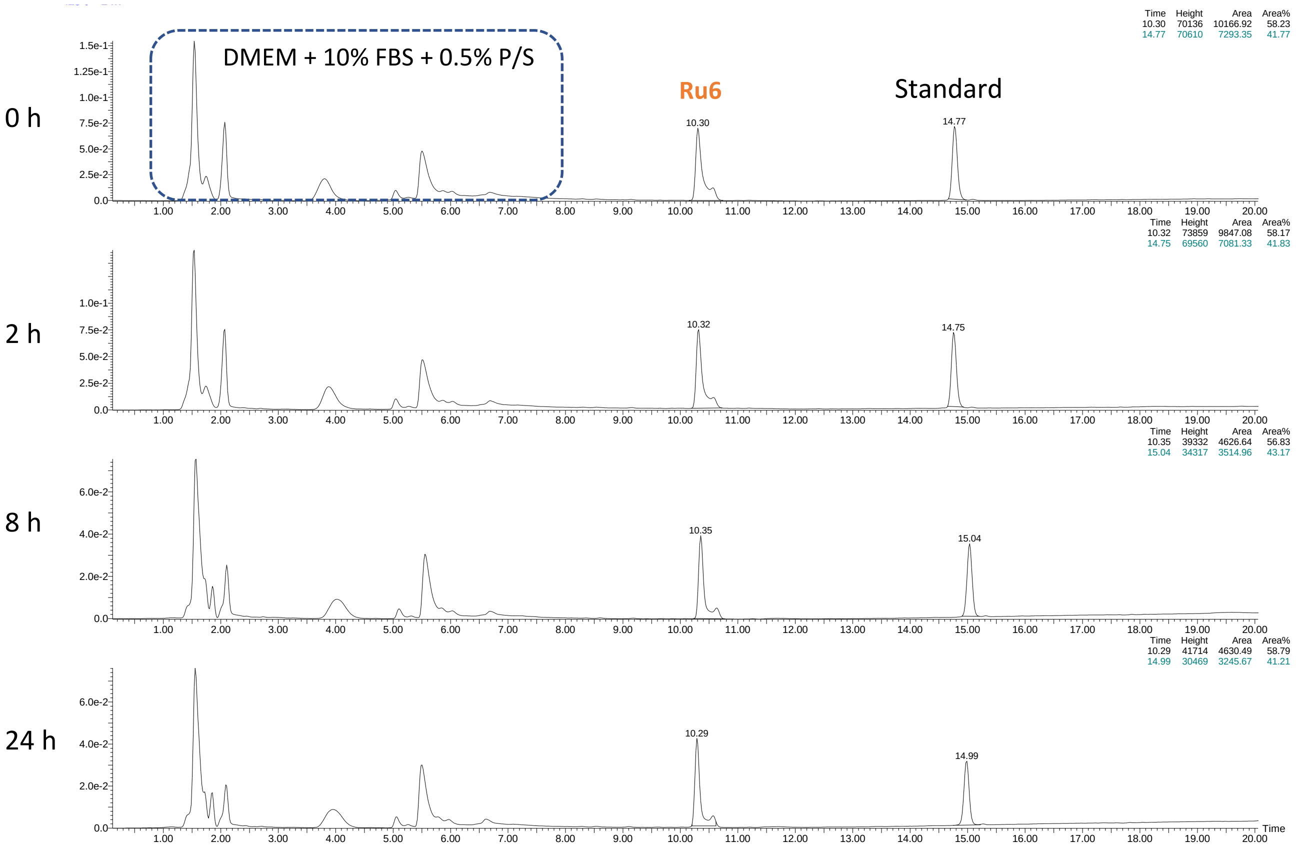

Figure S28 HPLC chromatograms showing the stability of **Ru6** in cell culture medium in the dark at 37 ºC after 0 h, 2 h, 8 h and 24 h.

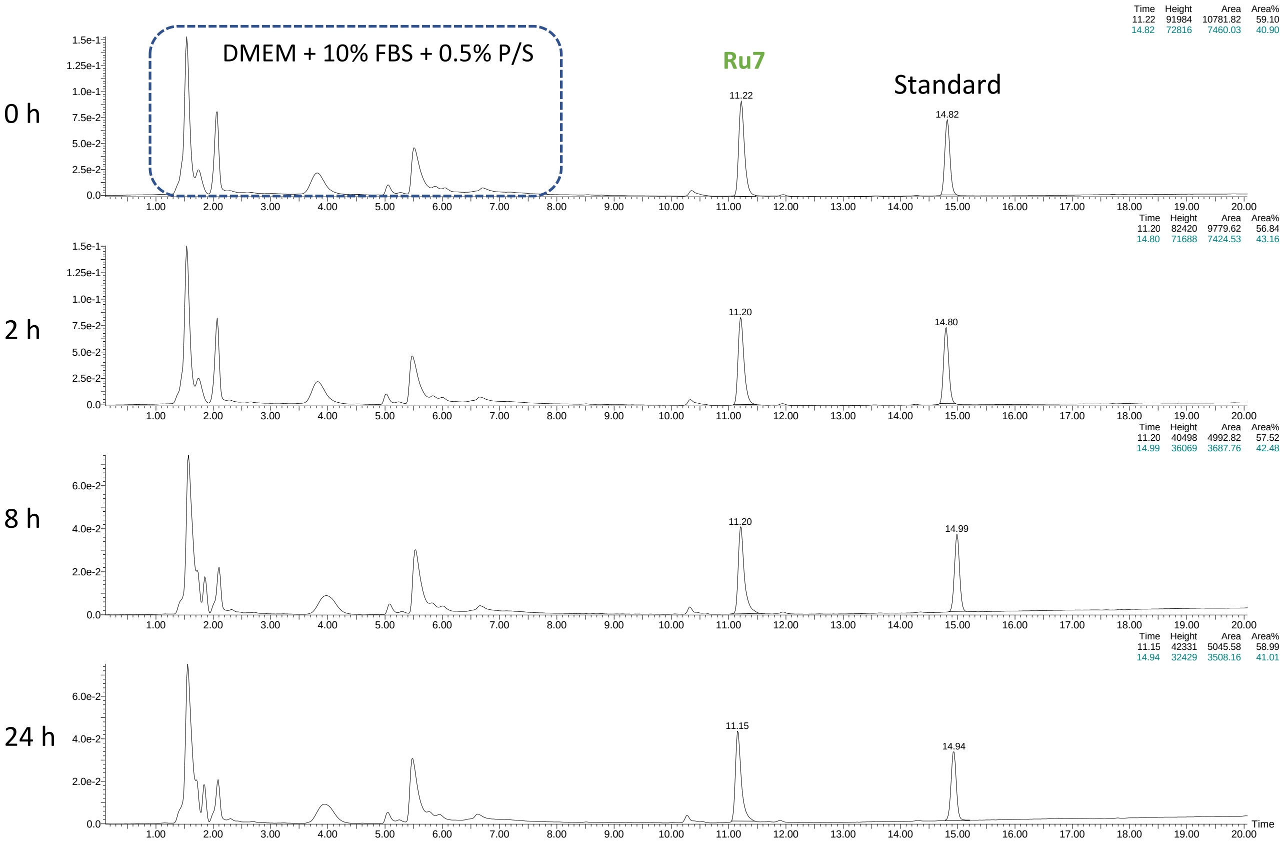

Figure S29. HPLC chromatograms showing the stability of **Ru7** in cell culture medium in the dark at 37 ºC after 0 h, 2 h, 8 h and 24 h.

3.2. Photostability

For the photostability studies, a solution of the compounds (30 µM) in DMEM culture medium supplemented with 10% FBS, 2 mM L-glutamine and 100 U·mL^{-1} of penicillin-streptomycin mixture, and containing 2,3,6,7-tetrahydro-1,1,7,7,9-pentamethyl-1*H*,5*H*,11*H*[1]benzopyrano-[6,7,8-*ij*]quinolizin-11-one (50 µM) as an internal standard, was irradiated for 2 h at 37 °C using a custom-built setup from Microbeam. This setup includes a thermostated cuvette holder and a mounted high-power red LED (620 ± 15 nm, 130 mW·cm^{-2}). The cuvette containing 1.5 mL of solution was placed directly in front of the light source and stirred continuously during irradiation. The irradiance at the sample position was measured using a calibrated optical power meter, Aliquots of 50 µL were taken at t = 0 and after irradiation for the indicated time intervals for each compound (15, 30, 45, 60, 90 and 120 min or 1, 2, 3, 4, 5, 10, 15 min) and analyzed by reversed-phase HPLC. The extent of photodegradation of the PS was evaluated by comparing the ratio between the peak area of the compounds and that of the internal standard, before and after irradiation at the specified time intervals. Photostability data were plotted as a function of the total fluence (in J·cm^{-2}), calculated from the measured irradiance and irradiation time.

The HPLC analysis was performed with a Waters alliance 2695 Separations Module, comprised of a quaternary pump solvent delivery module, online degasser, auto sampler and a Waters 2996 photodiode array detector. HPLC separation was carried out using a Jupiter Proteo C12 column (150 x 4.6 mm, 90 Å, 4 µm) from Phenomenex. The mobile phase was a linear gradient beginning with 70:30 (v/v) A/B and ending with 0:100 (v/v) A/B over 15 min at a flow rate of 1 mL/min (A: 0.05% TFA in H_2O; B: 0.05% TFA in ACN). The injection volume was 50 µL. Control of the HPLC instrument, as well as processing of the chromatogram output (annotation of retention times, integration of peaks, calculation of peak areas) was carried out with MassLynx V4.1 software. Elution traces were obtained at 260 nm.

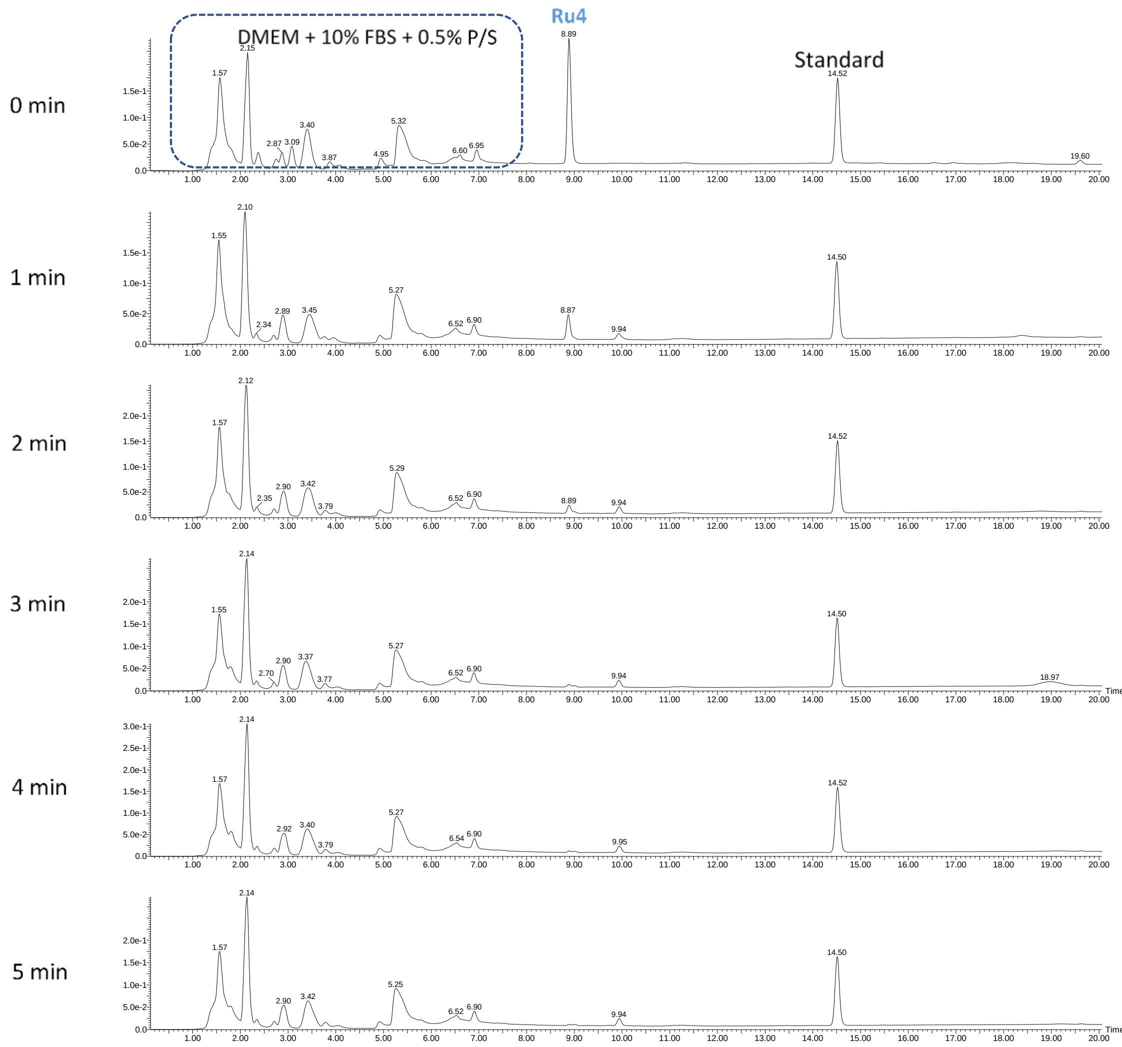

Figure S30. Photostability of **Ru4** in supplemented DMEM culture medium upon irradiation with red light. From top to bottom: HPLC chromatograms of **Ru4** + standard in culture medium before and after irradiation with red light for 1 min, 2 min, 3 min, 4 min and 5 min at 37 ºC.

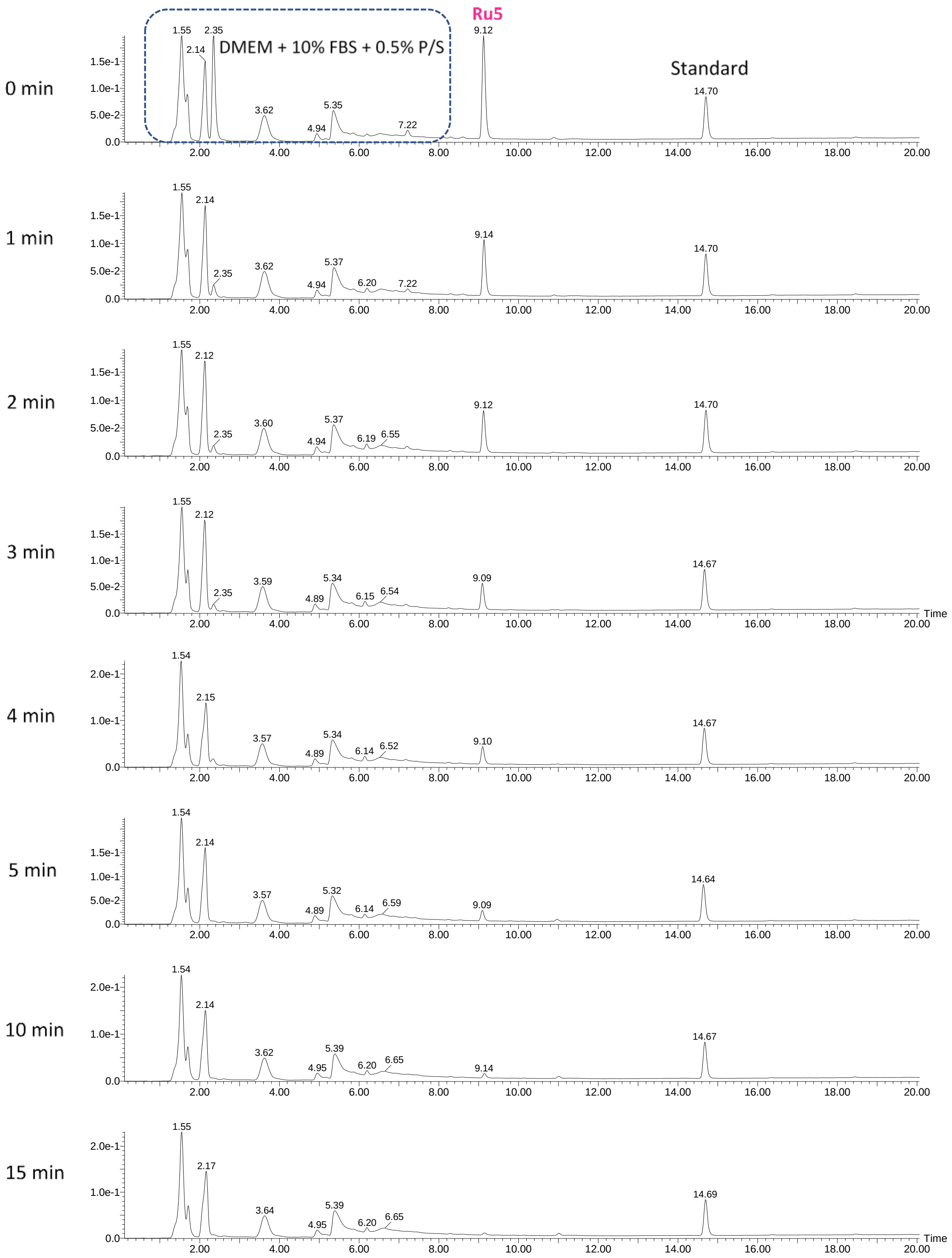

Figure S31. Photostability of **Ru5** in supplemented DMEM culture medium upon irradiation with red light. From top to bottom: HPLC chromatograms of **Ru5** + standard in culture medium before and after irradiation with red light for 1 min, 2 min, 3 min, 4, 5 min, 10 min and 15 min at 37 ºC.

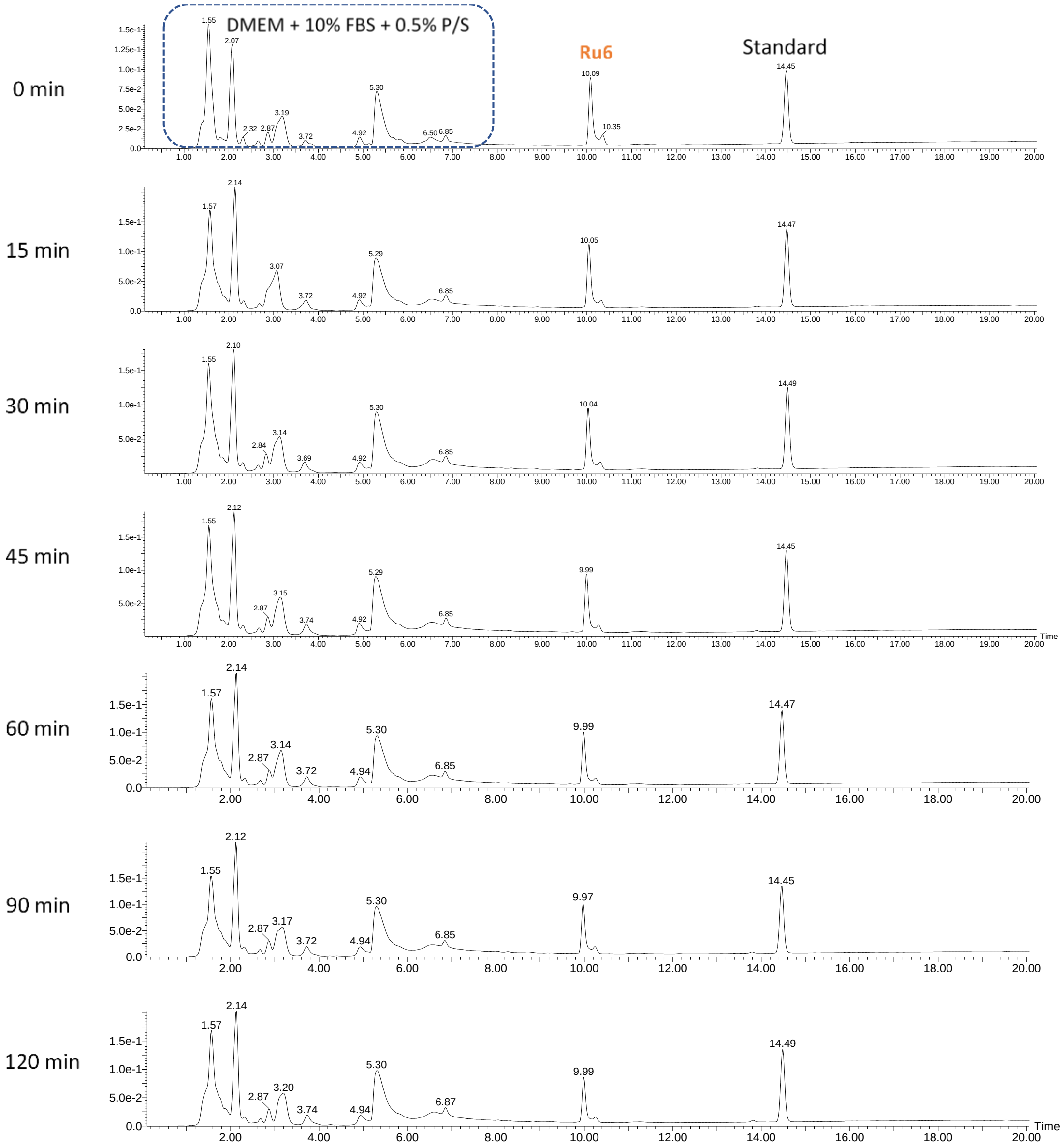

Figure S32. Photostability of **Ru6** in supplemented DMEM culture medium upon irradiation with red light. From top to bottom: HPLC chromatograms of **Ru6** + standard in culture medium before and after irradiation with red light for 15 min, 30 min, 45 min, 60 min, 90 min and 120 min at 37 ºC.

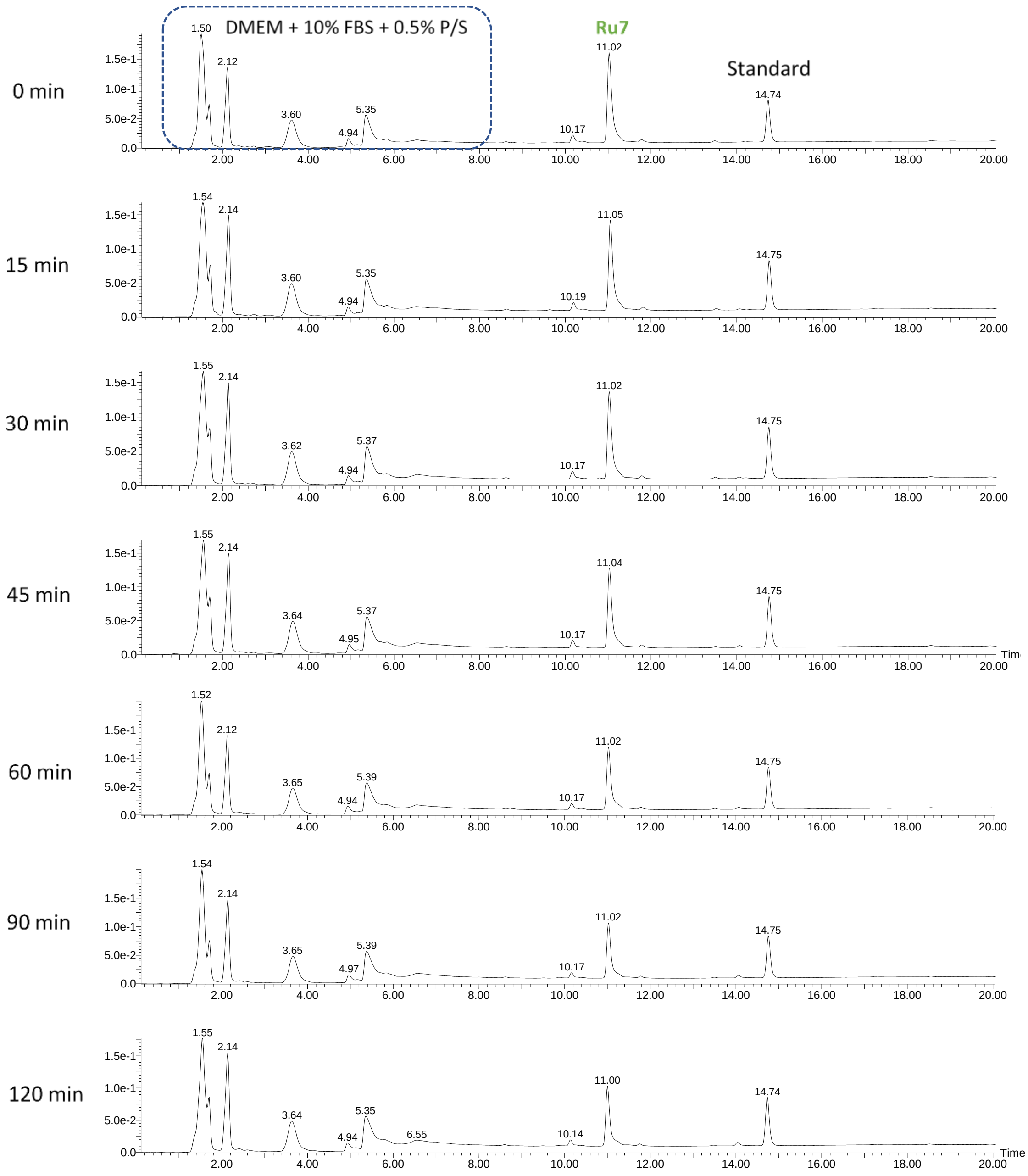

Figure S33. Photostability of **Ru7** in supplemented DMEM culture medium upon irradiation with red light. From top to bottom: HPLC chromatograms of **Ru7** + standard in culture medium before and after irradiation with red light for 15 min, 30 min, 45 min, 60 min, 90 min and 120 min at 37 ºC.

4.- Photochemical characterization: experimental and computational studies

4.1. Evaluation of singlet oxygen generation using SOSG

All fluorescence measurements were carried out using a Hellma® fluorescence quartz cuvette (Merck catalog number Z800546) with four clear Suprasil® quartz windows, a PTFE stopper, and a 1 cm path length along the excitation direction. The cuvette has a total capacity of 1.5 mL and is designed for use with magnetic stirrers. SOSG (5 µM) was added to a solution of the corresponding studied compound (10 µM) in PBS containing 2 % DMSO. The resulting solutions were irradiated with red LED light (620±15 nm, 130 mW·cm^{-2}) for the indicated time intervals (0, 1, 2, 3, 4 and 5 min). Immediately, the fluorescence spectra of the irradiated samples were collected by using a Photon Technology International (PTI) fluorimeter. The excitation wavelength was set to 500 nm (with excitation performed along the 1 cm path length), and emission spectra were recorded from 510-600 nm (SOSG: λ_{Ex}=504 nm, λ_{Em}=525 nm). The entrance and exit slits of the excitation and emission monochromators were set at 0.5 mm, providing a spectral bandwidth of 2 nm. The data interval was 1 nm, and the integration time was 0.7 sec. Positive control experiments were conducted using Methylene Blue (MB) as a reference, while negative control experiments were performed using sodium azide-saturated PBS as a singlet oxygen scavenger.

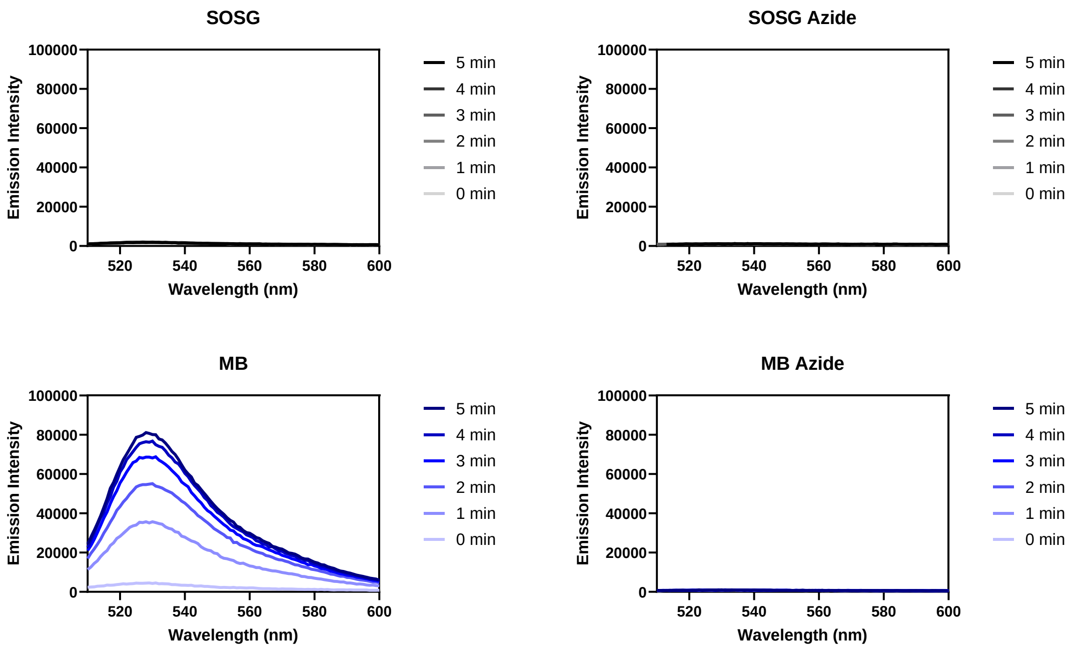

Figure S34. Top: Emission spectra of SOSG upon irradiation with red light in PBS (2 % DMSO) or in the presence of sodium azide-saturated PBS (2 % DMSO). Bottom: Photogeneration of singlet oxygen by MB. Increase of the fluorescence spectra emission of SOSG upon irradiation of MB with red light in PBS (2 % DMSO) alone or in the presence of sodium azide-saturated PBS (2 % DMSO).

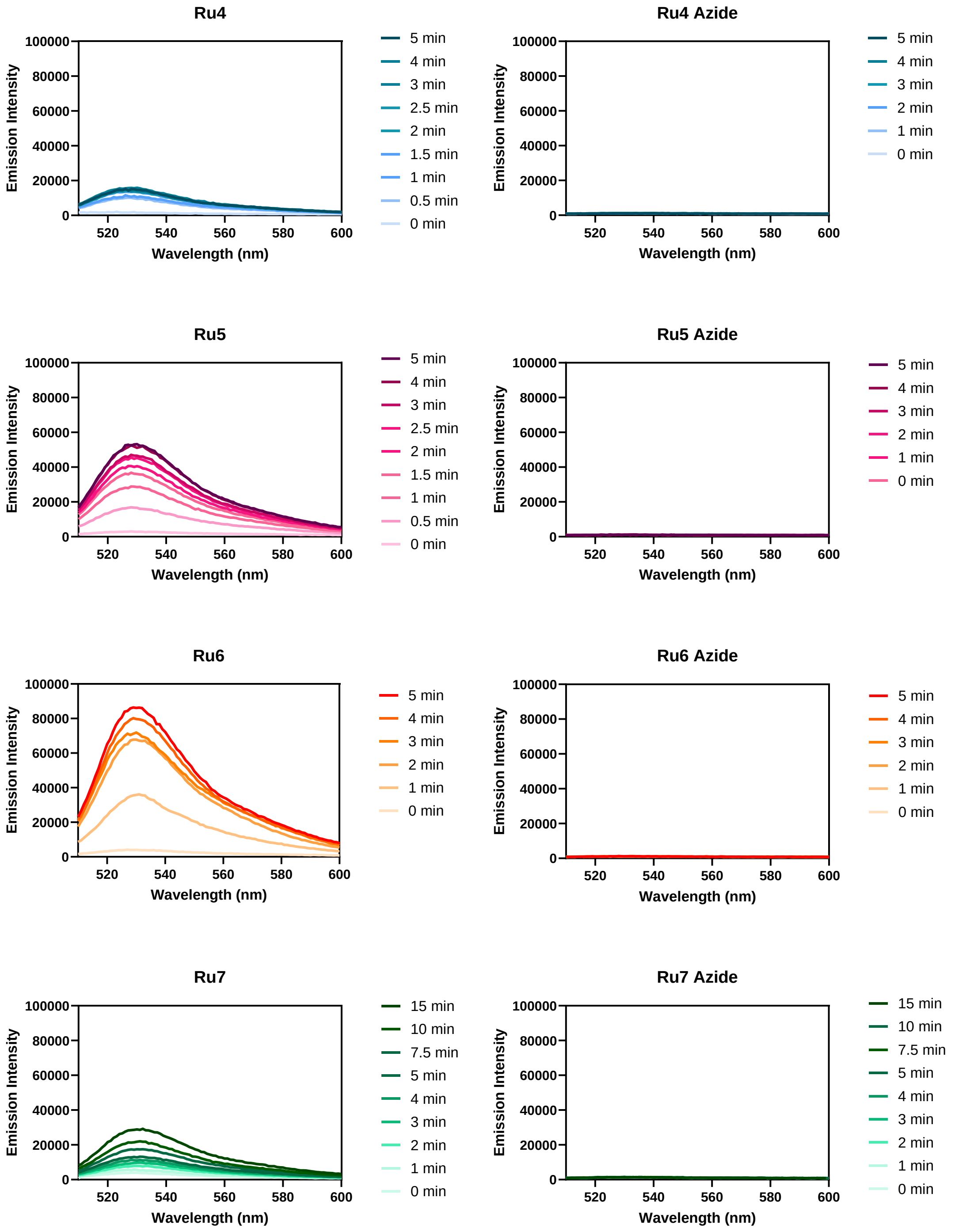

Figure S35. Photogeneration of singlet oxygen by Ru-COUBPY complexes **Ru4-7**. Increase of the fluorescence spectra emission of SOSG upon irradiation of the compounds alone with red light in PBS (2 % DMSO) or in the presence of sodium azide-saturated PBS (2 % DMSO).

4.2. Quantification of singlet oxygen generation

Following previously reported procedures,[15] the singlet oxygen quantum yields of Ru-COUBPY complexes were determined in an air-saturated DCM solution (bubbled for 15 min) using 1,3-diphenylisobenzofuran (DPBF) as a chemical trap under red light irradiation from a high-power LED source (620±15 nm; 130 mW cm^{-2}).[16] Upon reacting with singlet oxygen, the fluorescent scavenger DPBF decomposes into a colorless product.[17] The initial absorbance of DPBF in DCM was adjusted to approximately 1.0 (50 µM). Ru-COUBPY complexes were then added to the cuvette, and their absorbance was adjusted to 0.06 at the irradiation wavelength (620 nm). The decrease in the absorbance of DPBF at 411 nm was monitored after irradiation at 620 nm. The linear relationship between the change in absorbance (A_0-A_t) of DPBF at 411 nm and the irradiation time was plotted, as shown in Figure S36. Singlet oxygen quantum yields were calculated using the following equation:

$$\Phi_{\Delta,\mathrm{sample}} = \Phi_{\Delta,\mathrm{ref}} \frac{m_s}{m_r} \frac{(1-10^{\mathrm{A\lambda r}})}{(1-10^{\mathrm{A\lambda s}})}$$

where $\Phi_{\Delta,\mathrm{ref}}$ is the reference singlet oxygen quantum yield of methylene blue (MB) ($\Phi_{\Delta,\mathrm{ref}}$ = 0.57 in aerated DCM),[18] *m* are the slopes and Aλs and Aλr are the absorbance of the compounds and of the reference MB at the irradiation wavelength, respectively.

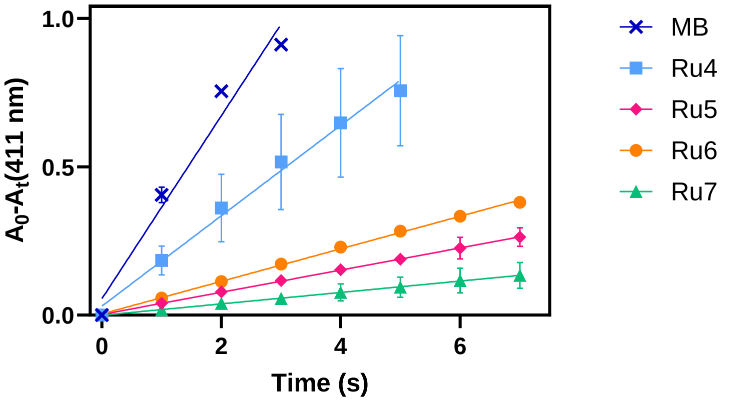

Figure S36. Plot of the changes in the absorbance (A_0-A_t) of DPBF at 411 nm against irradiation time with green light in the presence of the standard sensitizer MB and the Ru-COUBPY complexes in aerated DCM.

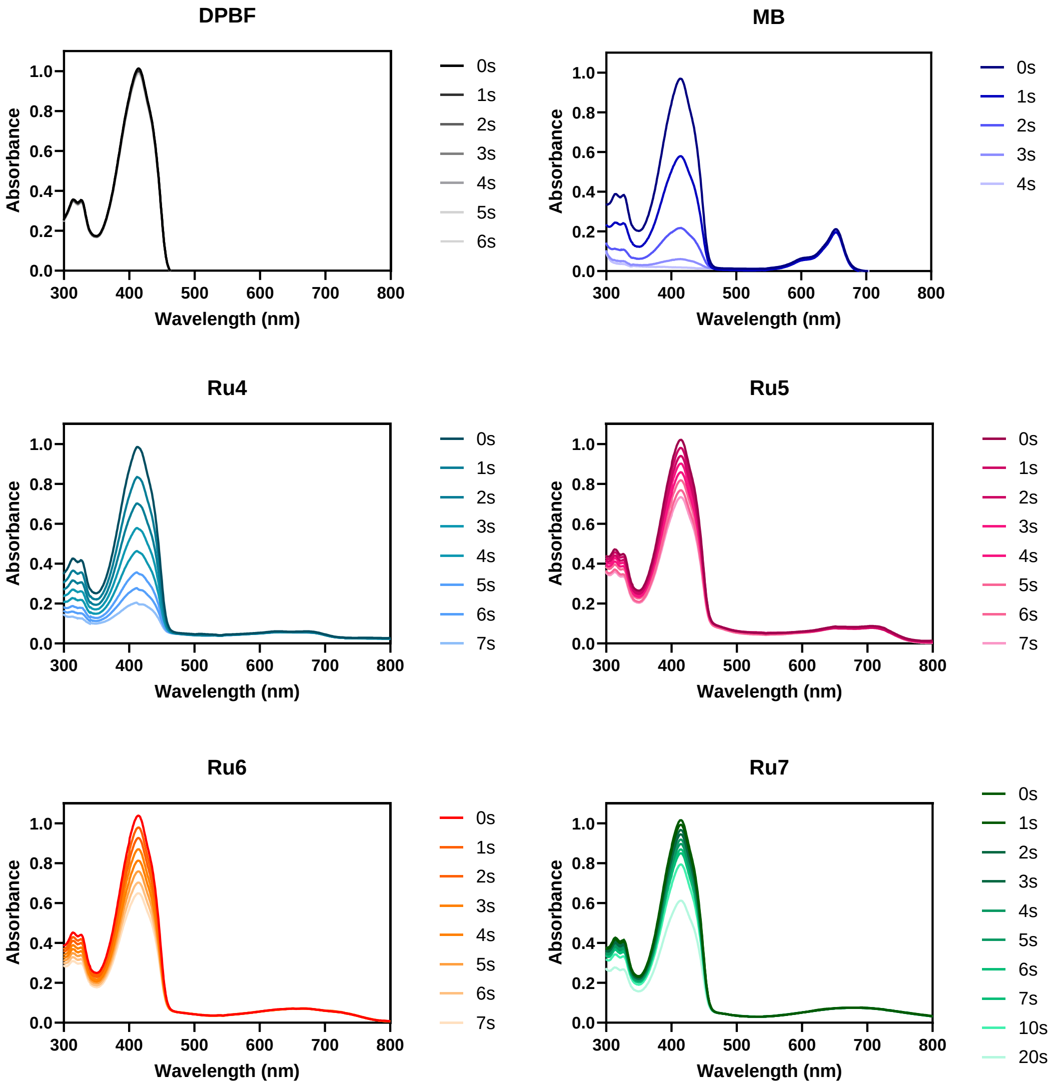

Figure S37. Changes in the absorption spectra of DPBF resulting from the irradiation with red LED light and in the presence of **MB** or Ru-COUBPY complexes.

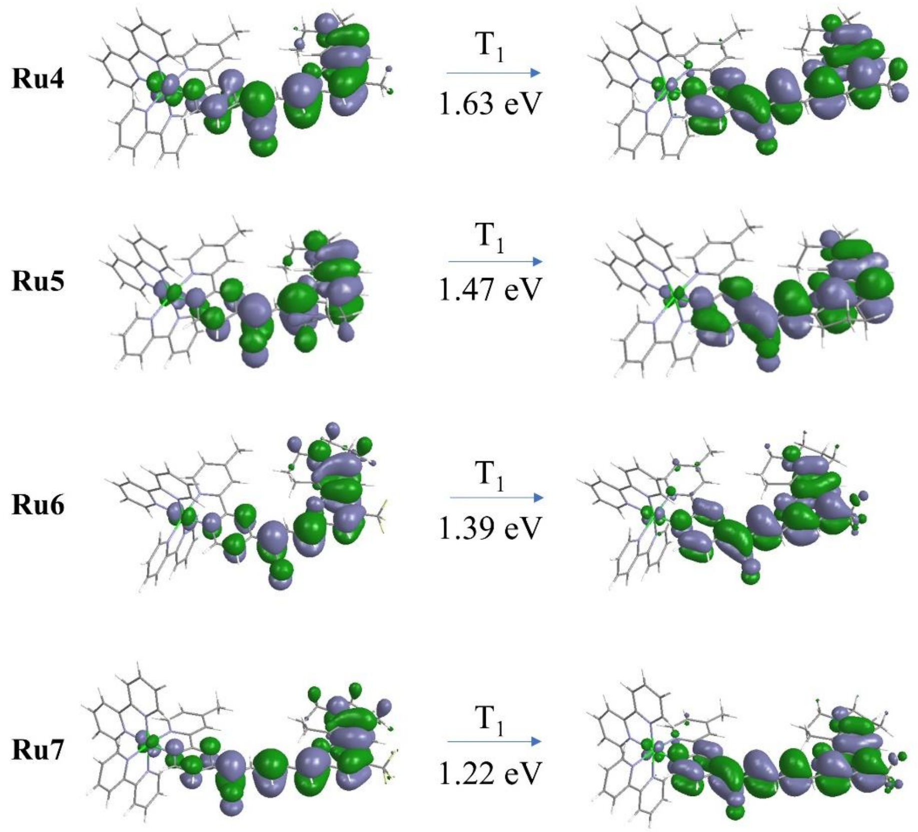

Figure S38. Natural Transition Orbitals of the lowest triplet state (T_1) for π-extended Ru-COUBPY complexes.

4.3. Evaluation of superoxide anion radical generation using DHR123

All fluorescence measurements were carried out using a Hellma® fluorescence quartz cuvette (Merck catalog number Z800546) with four clear Suprasil® quartz windows, a PTFE stopper, and a 1 cm path length along the excitation direction. The cuvette has a total capacity of 1.5 mL and is designed for use with magnetic stirrers. DHR123 (10 µM) was added to a solution of the studied compound (10 µM) in PBS containing 2% DMSO. The resulting solutions were irradiated with red LED light (620±15 nm, 130 mW·cm^{-2}) for the specified time intervals (0, 1, 2, 3, 4, 5, 7.5, 10 and 15 min). Immediately, the fluorescence spectra of the irradiated samples were collected by using a Photon Technology International (PTI) fluorimeter. The excitation wavelength was set to 500 nm (with excitation performed along the 1 cm path length), and emission spectra were recorded from 510 nm to 600 nm (DHR123: λ_{Ex}=507 nm, λ_{Em}=529 nm). The entrance and exit slits of the excitation and emission monochromators were set at 0.5 mm, giving a spectral bandwidth of 2 nm. The data interval was 1 nm, and the integration time was 0.5 sec. Positive control experiments were conducted using MB as a reference, while negative control experiments were performed using sodium 4,5-dihydroxybenzene-1,3-disulfonate (tiron)-saturated PBS as a superoxide anion radical scavenger.

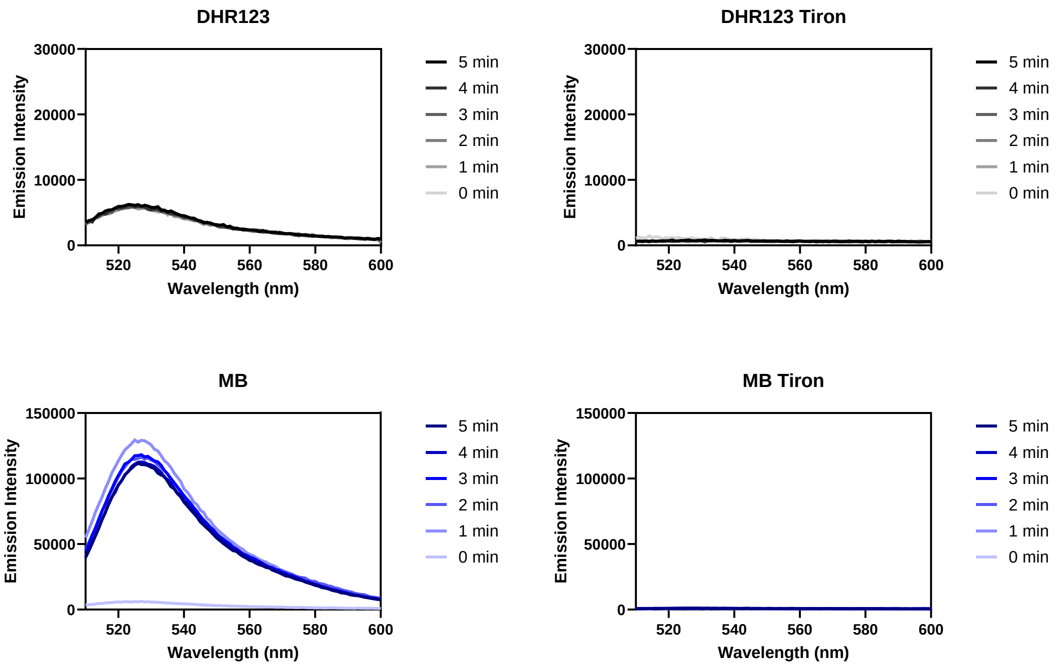

Figure S39. Top: Emission spectra of DHR123 alone upon irradiation with red light in PBS (2 % DMSO) or in the presence of tiron-saturated PBS (2 % DMSO). Bottom: Photogeneration of superoxide anion radical by **MB**. Increase of the fluorescence spectra emission of HPF upon irradiation of the compound and HPF with red light in PBS (2 % DMSO) or in the presence of tiron-saturated PBS (2 % DMSO).

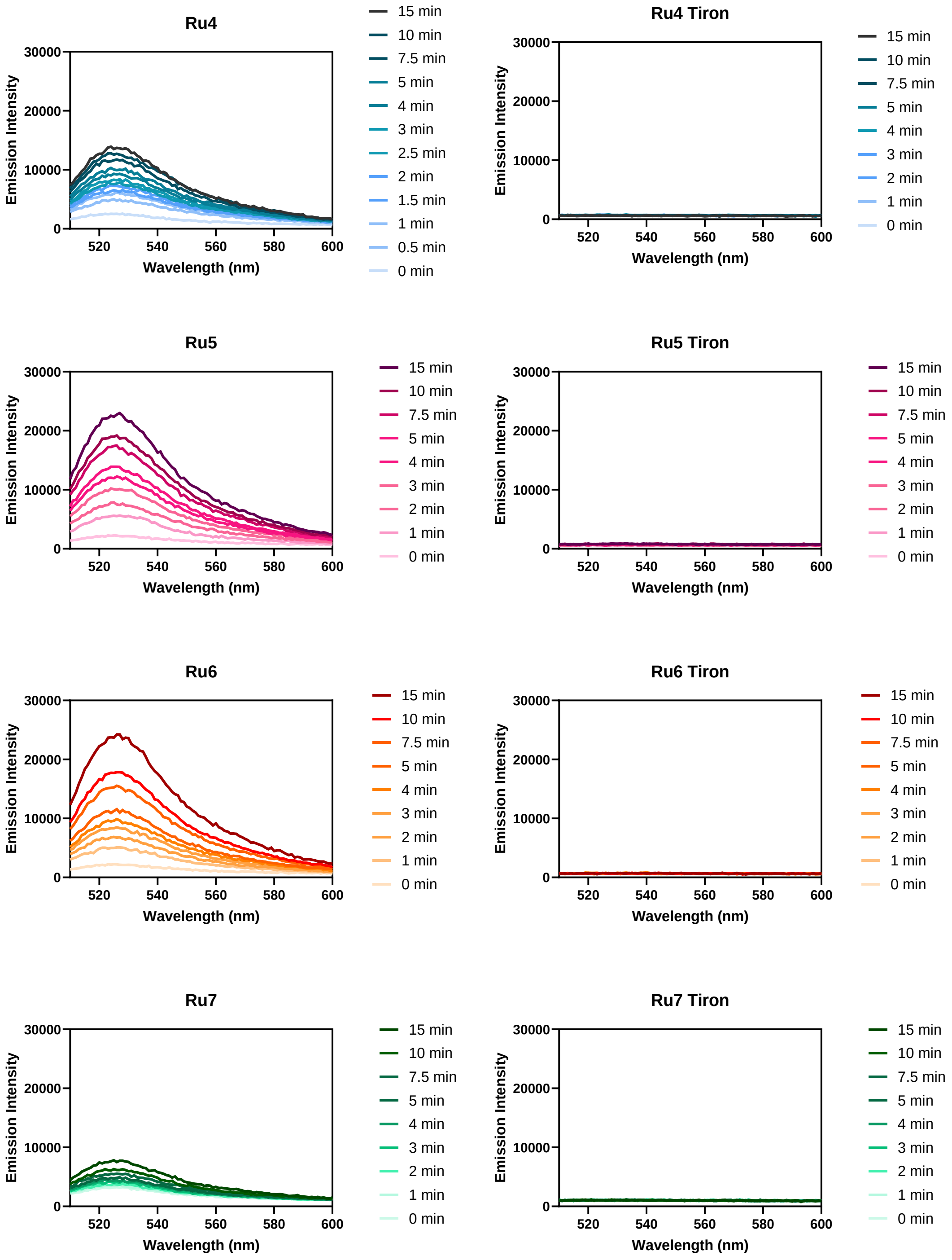

Figure S40. Photogeneration of superoxide anion radical by Ru-COUBPY complexes **Ru4-7**. Increase of the fluorescence spectra emission of DHR123 upon irradiation of the compounds alone with red light in PBS (2 % DMSO) or in the presence of tiron-saturated PBS (2 % DMSO).

4.4. Evaluation of hydroxyl radical generation using HPF

All fluorescence measurements were carried out using a Hellma® fluorescence quartz cuvette (Merck catalog number Z800546) with four clear Suprasil® quartz windows, a PTFE stopper, and a 1 cm path length along the excitation direction. The cuvette has a total capacity of 1.5 mL and is designed for use with magnetic stirrers. HPF (5 µM) was added to a solution of the studied compound (10 µM) in PBS containing 2% DMSO. The resulting solutions were irradiated with red (620±15 nm, 130 mW·cm^{-2}) LED light for the indicated time intervals (0, 1, 2, 3, 4 and 5 min). Immediately, the fluorescence spectra of the irradiated samples were collected by using a Photon Technology International (PTI) fluorimeter. The excitation wavelength was set to 490 nm (with excitation performed along the 1 cm path length), and emission spectra were recorded from 500 nm to 600 nm (HPF: λ_{Ex}=490 nm, λ_{Em}=515 nm). The entrance and exit slits of the excitation and emission monochromators were set at 0.5 mm, giving a spectral bandwidth of 2 nm. The data interval was 1 nm and the integration time was 0.5 sec. Positive control experiments were conducted using MB as a reference, while negative control experiments were performed using terephthalic acid (TA)-saturated PBS as a hydroxyl radical scavenger.

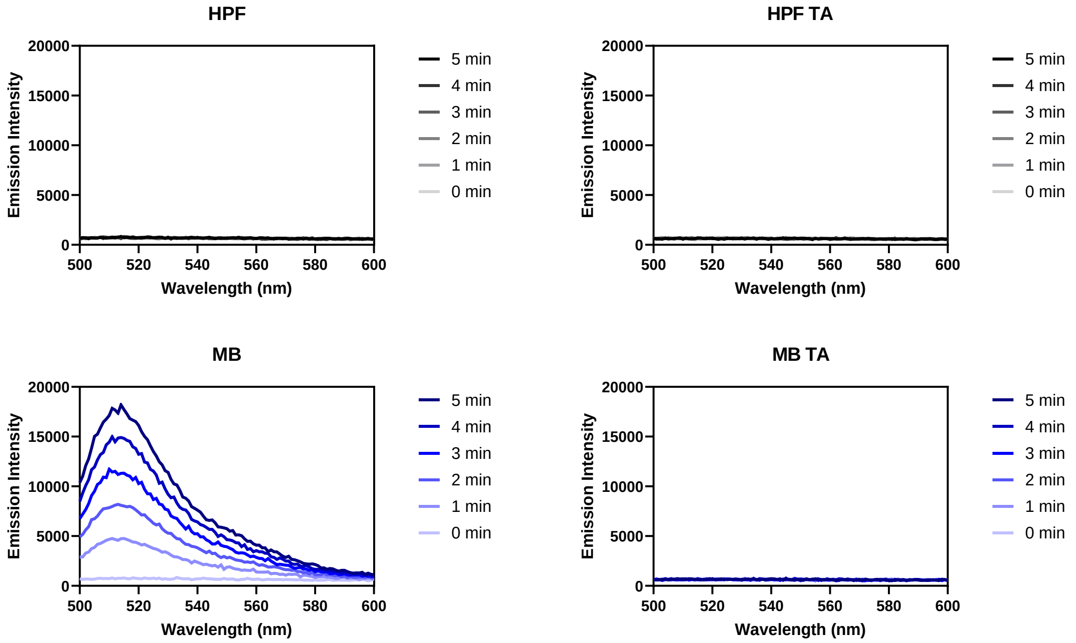

Figure S41. Top: Emission spectra of HPF alone upon irradiation with red light in PBS (2 % DMSO) or in the presence of TA-saturated PBS (2 % DMSO). Bottom: Photogeneration of hydroxyl radical by **MB**. Increase of the fluorescence spectra emission of HPF upon irradiation of the compound and HPF with red light in PBS (2 % DMSO) or in the presence of TA-saturated PBS (2 % DMSO).

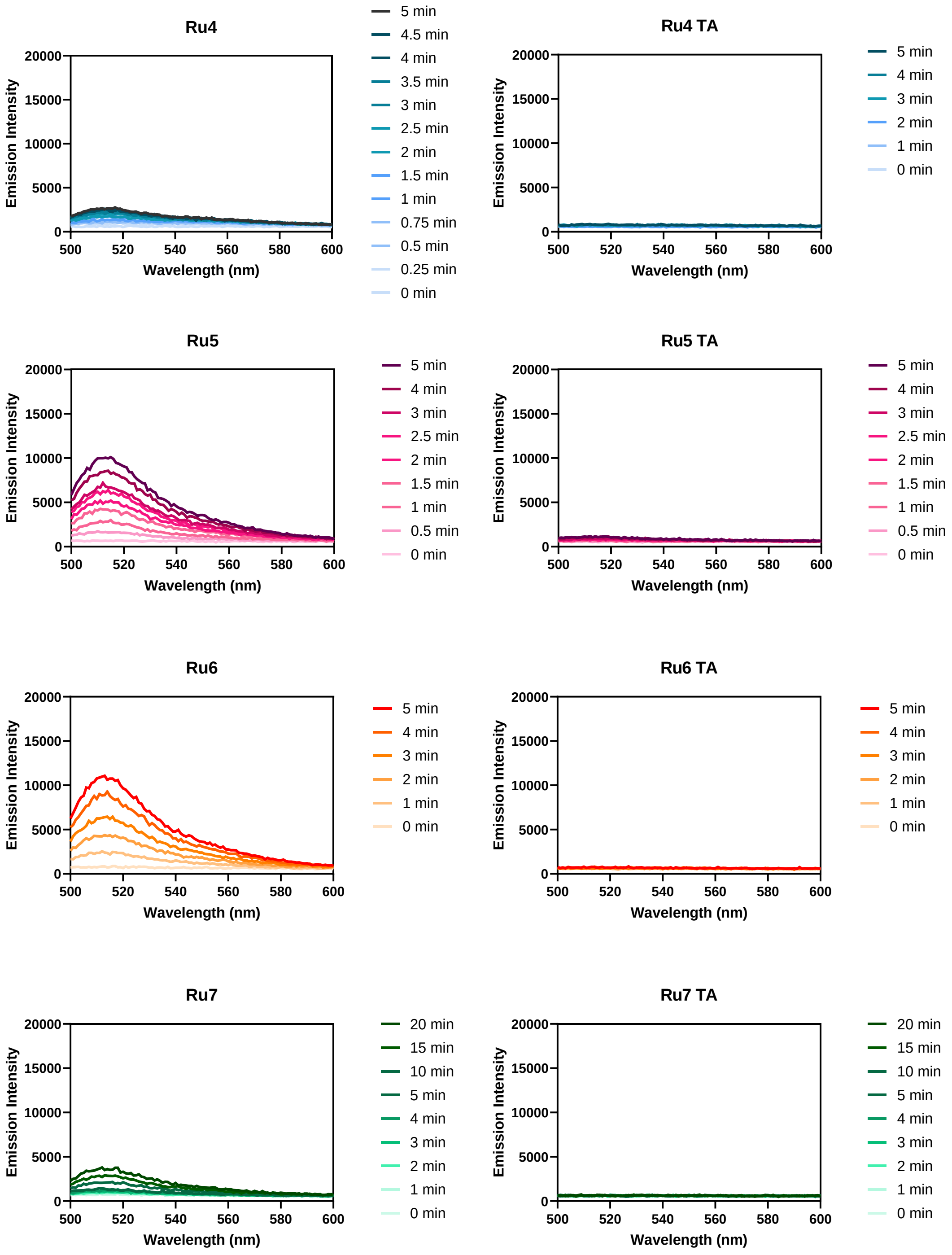

Figure S42. Photogeneration of hydroxyl radical by Ru-COUBPY complexes **Ru4-7**. Increase of the fluorescence spectra emission of HPF upon photoirradiation of the compounds alone with red light in PBS (2 % DMSO) or in the presence of TA-saturated PBS (2 % DMSO).

4.5. Electron paramagnetic resonance (EPR) studies

The corresponding Ru-COUBPY complex (500 µM) was dissolved in MeOH containing either 60 mM 4-amino-TEMP (4-amino-2,2,6,6-tetramethylpiperidine) as a spin trap for $^1O_2$, or 720 mM DMPO (5,5-dimethyl-1-pyrroline-N-oxide) as a spin trap for $O_2^{\bullet-}$. For $^{\bullet}OH$ radical trapping, DMPO was dissolved in PBS. In all cases, the resulting samples were loaded into Hirschmann 50 µL capillary tubes (both ends open) and sealed with Critoseal®. EPR measurements were conducted using a Bruker Elexsys 580 spectrometer operating in the X-band at room temperature, equipped with a Bruker ER4122 SHQE super-high-Q cylindrical resonator in continuous-wave (CW) mode. The EPR spectra shown in Figure 3 were recorded both in the dark and after white-light irradiation with an Asahi Spectra MAX-303 xenon light source (300 W) equipped with a 430–1000 nm bandpass filter. The light was delivered to the EPR cavity via a quartz light guide through the optical window of the ER4122 SHQE resonator. The microwave frequency was 9.858 GHz, with a modulation amplitude of 0.03 mT and a microwave power of 4.7 mW. These parameters were selected to avoid signal distortion or saturation. For the EPR spectra presented in Figure S43, irradiation was performed outside the EPR cavity for 2 min using a high-power red-light LED (620 nm, 130 mW cm^{-2}) to facilitate the detection of $^1O_2$.

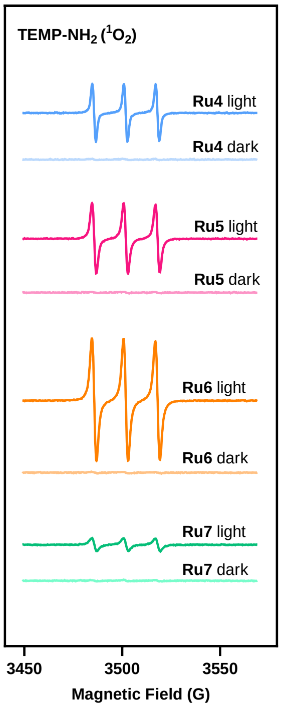

Figure S43. Photogeneration of singlet oxygen by *π*-extended Ru-COUBPY complexes Ru4-7, analyzed using EPR spectroscopy**.** EPR spectra of **Ru4-7** trapped by 4-amino-TEMP in MeOH, measured in the dark and after red-light irradiation (2 min, 620 ± 15 nm, 130 mW/cm²).

4.6. Computational studies

Vertical electron affinities (VEAs) and ionization potentials (VIPs) listed in Table S5 are computed as follows:

$$\mathrm{VEA} = E(\mathrm{A}_{n+1}) - E(\mathrm{A}_n)$$

$$\mathrm{VIP} = E(\mathrm{A}_{n-1}) - E(\mathrm{A}_n)$$

Where E stands for absolute energy, A is the molecule under study, and n refers to the total number of electrons. VEAs and VIPs have been computed employing restricted and unrestricted DFT ansatzes for closed and open shell systems, respectively. VEAs and VIPs have been computed with the PBE0/6-31+G(d,p)/SDD method except the T_1 state contribution, which has been computed by adding the vertical T_1-S_0 energy gap determined with the TD-M06 method for consistency with the TDFFT description of the excited states mentioned in section 2.2. Water has been described with the CPCM method, and all calculations have been performed with the Gaussian 16 quantum chemistry software without imposing any symmetry restraint.

The use of VEAs and VIPs to evaluate the feasibility of Type I mechanisms by metal- or metal-free photosensitizers for PDT is widely used in literature.[7,19,20,21,22]

Table S5. Vertical ionization potential (VIP) and electron affinity (VEA) in water. VEA for triplet oxygen ($^3O_2$) is also shown.[23] All energies in eV.

Complex	VEA(S_0)	VIP(S_0)	VEA(T_1)	VIP(T_1)
**Ru4**	-3.08	5.18	-4.71	3.55
**Ru5**	-3.09	5.06	-4.56	3.59
**Ru6**	-3.33	5.21	-4.72	3.82
**Ru7**	-3.41	5.06	-4.63	3.84

VEA($^3O_2$) = -3.43 eV in water

Table S6. Thermodynamics [$\Delta E = E$(products) $- E$(reactants)] of the Type I PDT reactions for **Ru6** based on the VEA and VIP values computed in acetonitrile. All energies in eV.

	**Autoionization Reactions**	ΔE(ev)
(1)	$^3Ps + ^1Ps \rightarrow {}^{\bullet}Ps^+ + {}^{\bullet}Ps^-$	0.53
(2)	$^3Ps + ^3Ps \rightarrow {}^{\bullet}Ps^+ + {}^{\bullet}Ps^-$	-0.86
	**Indirect Reaction**	
(3)	${}^{\bullet}Ps^- + {}^3O_2 \rightarrow {}^1Ps + {}^{\bullet}O_2^-$	-0.06
	**Direct Electron transfer**	
(4)	$^1Ps + ^3O_2 \rightarrow {}^{\bullet}Ps^+ + {}^{\bullet}O_2^-$	2.35
(5)	$^3Ps + ^3O_2 \rightarrow {}^{\bullet}Ps^+ + {}^{\bullet}O_2^-$	0.47

4.7. Cyclic voltammetry

Cyclic voltammetry (CV) experiments were performed using a portable potentiostat/galvanostat PalmSens3 (PalmSens) controlled by the software PSTrace4 (version 4.4.2). All experiments were carried out using a three-electrode cell with a glassy carbon disc (diameter = 3 mm) as the working electrode, a platinum-wire as the auxiliary electrode, and Ag/AgCl (MF-2052 BASi) as reference electrode. The solution of the metal complexes (1×10^{-4} M) in acetonitrile with the supporting electrolyte [nBu$_4$N][PF$_6$] (0.1 M) were deoxygenated by bubbling argon saturated with CH_3CN for 10 min and maintaining the argon-flow during the whole experiment. All the measurements were recorded at a scan rate of 0.1 $V{\cdot}s^{-1}$ in a clockwise direction. The experimental redox potentials are reported in reference to the the experimentally determined ferrocenium/ferrocene redox pair (Fc^+/Fc; $E°_{1/2}$ = +0.40 V *vs*. Ag/AgCl).

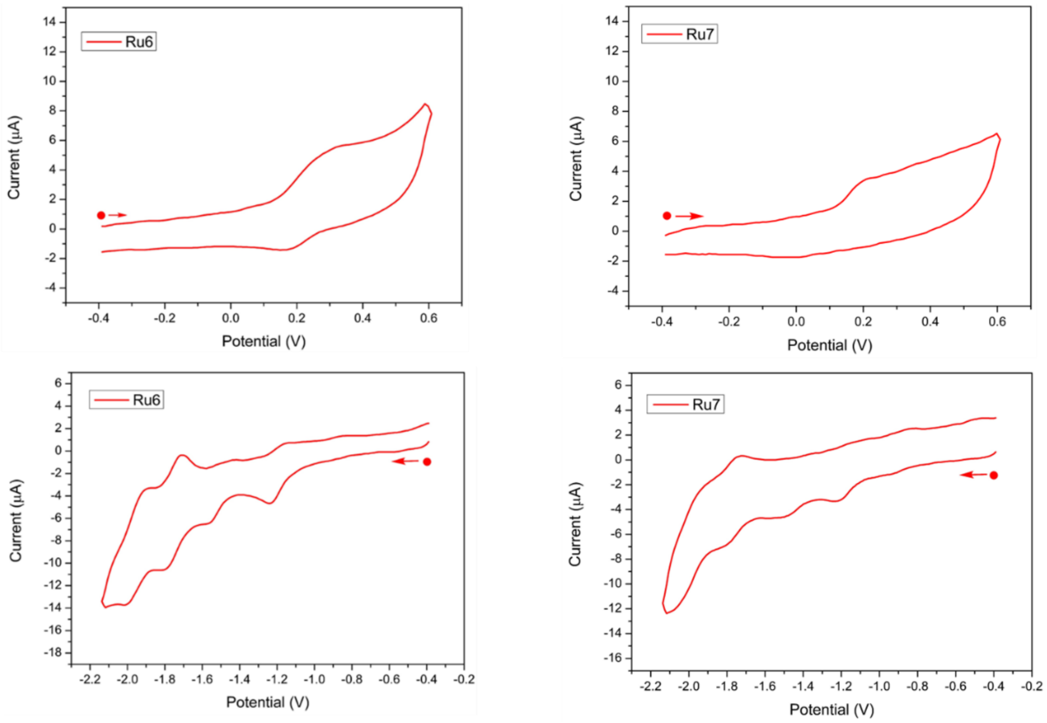

Figure S44. Cyclic voltammograms in the anodic and cathodic regions of **Ru6** and **Ru7** (1×10^{-4} M) in acetonitrile, using 0.1 M $[nBu_4N][PF_6]$ as supporting electrolyte and recorded with scan rate of 0.10 $V{\cdot}s^{-1}$. The cyclic voltammograms have been referenced to Fc^+/Fc.

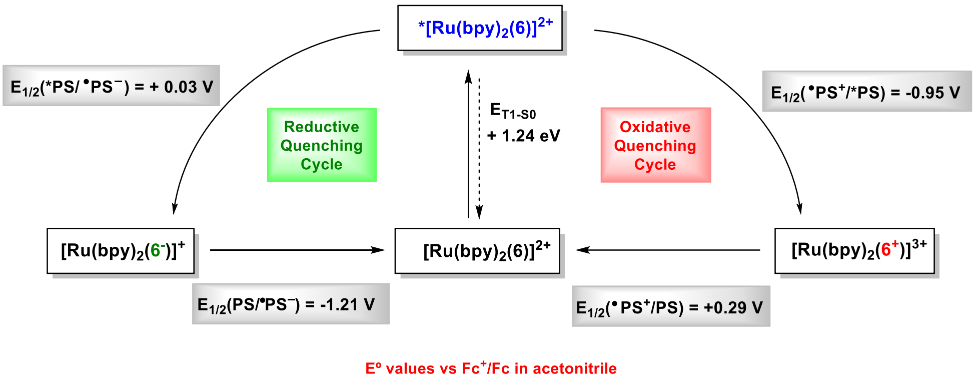

Figure S45. Latimer diagram for **Ru6**, with redox potentials determined by CV and the adiabatic energy difference ($\Delta E_{T1\text{-}S0}$).

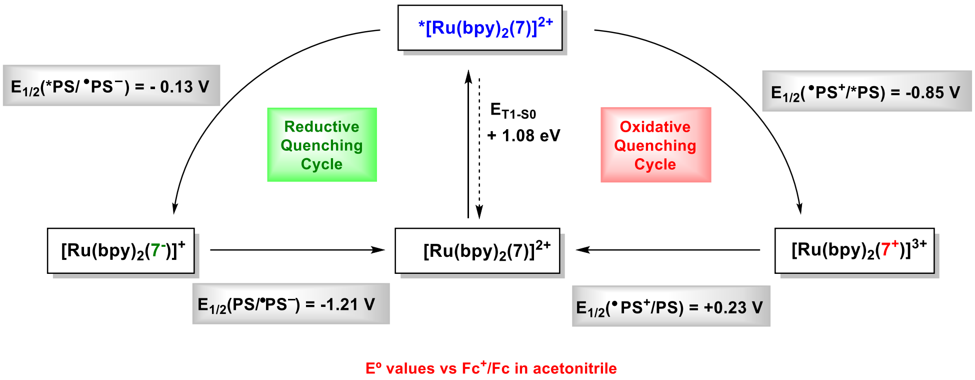

Figure S46. Latimer diagram for **Ru7**, with redox potentials determined by CV and the adiabatic energy difference ($\Delta E_{T1\text{-}S0}$).

Table S7. Redox potentials for complexes **Ru6** and **Ru7** referenced to Fc^+/Fc and recorded by cyclic voltammetry in acetonitrile solution in the anodic and cathodic regions. qr = quasi-reversible, ir = irreversible.[a]

Comp.	$E_{1/2}$ ($^{\bullet}PS^+/PS$) (*vs. Fc*$^+$*/Fc*) [V]	$E_{1/2}$ ($PS/^{\bullet}PS^-$) (*vs. Fc*$^+$*/Fc*) [V]	$\Delta E_{1/2}$ [V]	$\Delta E_{T1\text{-}S0}$ [eV][b]	$E_{1/2}$ ($^{\bullet}PS^+/PS^*$) (*vs. Fc*$^+$*/Fc*) [V]	$E_{1/2}$ ($PS^*/^{\bullet}PS^-$) (*vs. Fc*$^+$*/Fc*) [V]	$E_{1/2}$ ($^{\bullet}PS^+/PS^*$) (*vs. NHE*)[c]	$E_{1/2}$ ($PS^*/^{\bullet}PS^-$) (*vs. NHE*)[c]
**Ru6**	+0.29 (qr)	–1.21 (qr)	1.50	1.24	–0.95	+0.03	–0.32	+0.66
**Ru7**	+0.23 (ir)	-1.21 (ir)	1.44	1.08	–0.85	–0.13	–0.22	+0.50

[a] Voltammograms recorded in acetonitrile solution (1×10^{-4} M), using 0.1 M [*n*Bu_4N][PF_6] as supporting electrolyte with scan rate of 0.10 $V\cdot s^{-1}$ and referenced to Fc^+/Fc. [b] Adiabatic T_1-S_0 energy difference computed at the T_1 minimum in acetonitrile. [c] The conversion of potentials from Fc^+/Fc to NHE was calculated using the conversion constant reported by Pavlishchuk and Addison.[24]

5. Cellular uptake by confocal microscopy

Cell Culture and Treatments. HeLa cells were cultured in Dulbecco's Modified Eagle Medium (DMEM, 31966021 Gibco) containing GlutaMAX and high glucose (4.5 g/L), supplemented with 10% fetal bovine serum (FBS) and 50 U/mL penicillin-streptomycin. For cellular uptake experiments and subsequent microscopic observation, cells were seeded onto glass-bottom dishes (P35G-1.5-14-C, Mattek). Twenty-four hours after cell seeding, cells were incubated at 37 °C for 30 min with the compounds (10 μM) in supplemented DMEM. Then cells were washed two times with Dulbecco's Phosphate-Buffered Saline (DPBS, pH 7.0-7.3) to remove excess compounds and maintained in low glucose DMEM containing 10 mM HEPES (Corning) and lacking phenol red for fluorescence imaging.

For colocalization experiments with Mitotracker Green (MTG, ThermoFisher Scientific) or Lysotracker Green (LTG, ThermoFisher Scientific), HeLa cells were treated with the compounds (10 μM) in supplemented DMEM. Cells were then incubated with 0.5 μM MTG or LTG at 37 °C in unsupplemented DMEM for the appropriate duration (30 min or 5 min, respectively). After removing the medium and washing twice with DPBS, cells were maintained in low glucose DMEM with HEPES (10 mM) and without phenol red for fluorescence imaging.

Fluorescence Imaging. All microscopy observations were performed using a Zeiss LSM 880 confocal microscope equipped with an argon laser emitting at 488 nm and a 561 nm DPSS laser. The microscope was also equipped with a Heating Insert P S (Pecon). Cells were observed at 37ºC using a 63×1.4NA oil immersion objective. Π-extended Ru-COUBPY complexes were excited using the 561 nm laser line and detected from 570 to 760 nm. MTG and LTG were excited using the 488 nm laser line and detected from 495 to 560 nm. In all observations a stack of images was acquired with a 0.13 x 0.13 x 0.5 um of pixel size (xyz). Image processing and analysis were performed using Fiji.[25]

Colocalization analysis. Compound and tracker (MTG or LTG) images were processed by median filtering (radius = 1), Gaussian filtering (sigma = 1), and background subtraction (rolling ball radius = 30 for the *MTG* and radius = 10 for the *LTG* staining). In all experiments the Pearson correlation coefficient was measured in 3D using the JaCoP plugin. For each compound more than 45 cells on average were analysed.

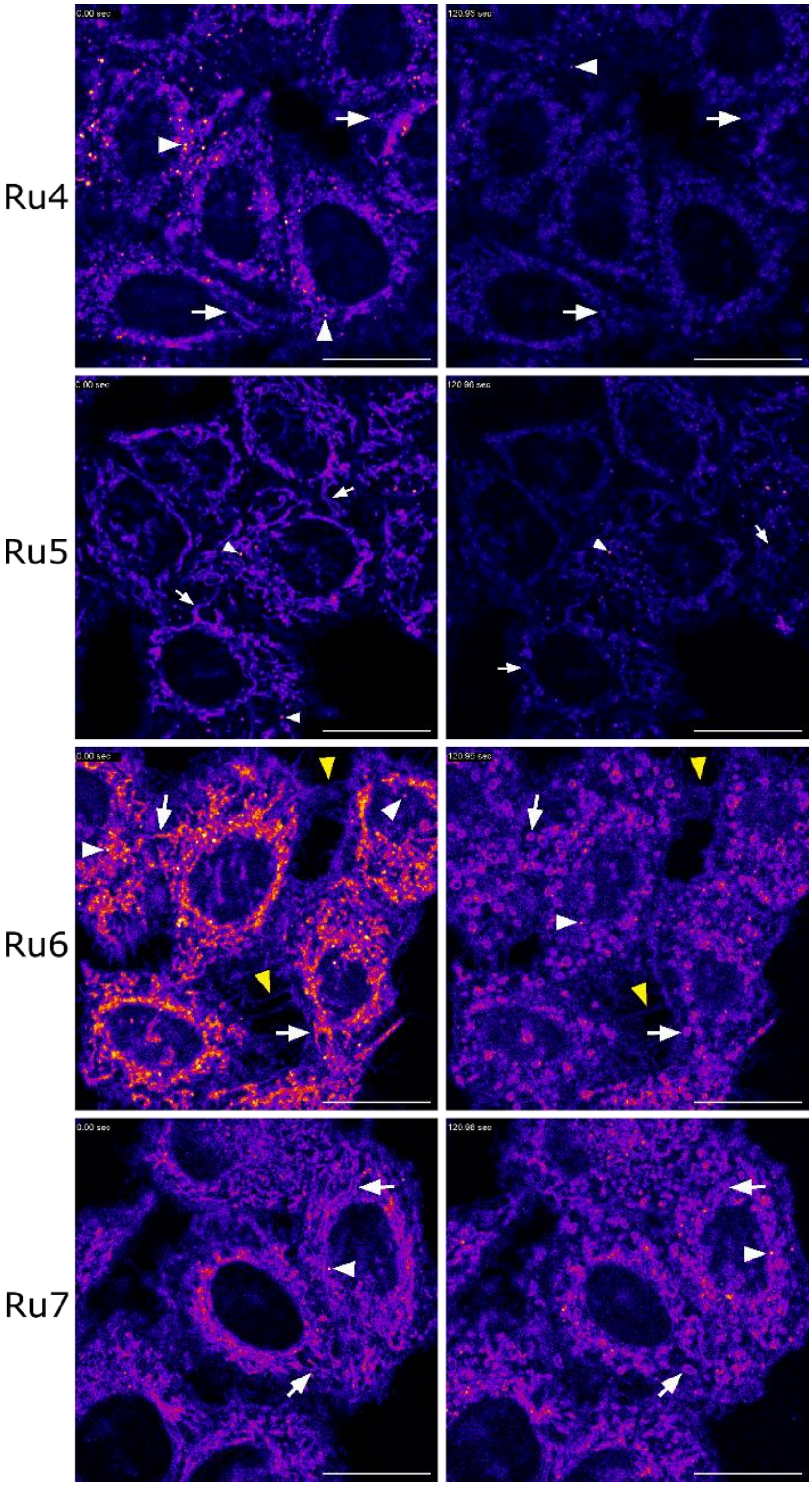

Figure S47. **Cellular uptake of Ru-COUBPY complexes Ru4-7**. Single confocal planes of HeLa cells incubated with the compounds (10 μM) for 30 min at 37 °C, imaged at t = 0 (left) and after 2 min (right) of first observation. Excitation was performed with the 561 nm laser line. White arrows point out mitochondria and white arrowheads vesicle staining. Yellow arrowheads for **Ru6** point out filopodia of the extracellular membrane of the cells. Scale bar: 20 μm. Fluorescence intensities are color coded using the “fire” look-up table.

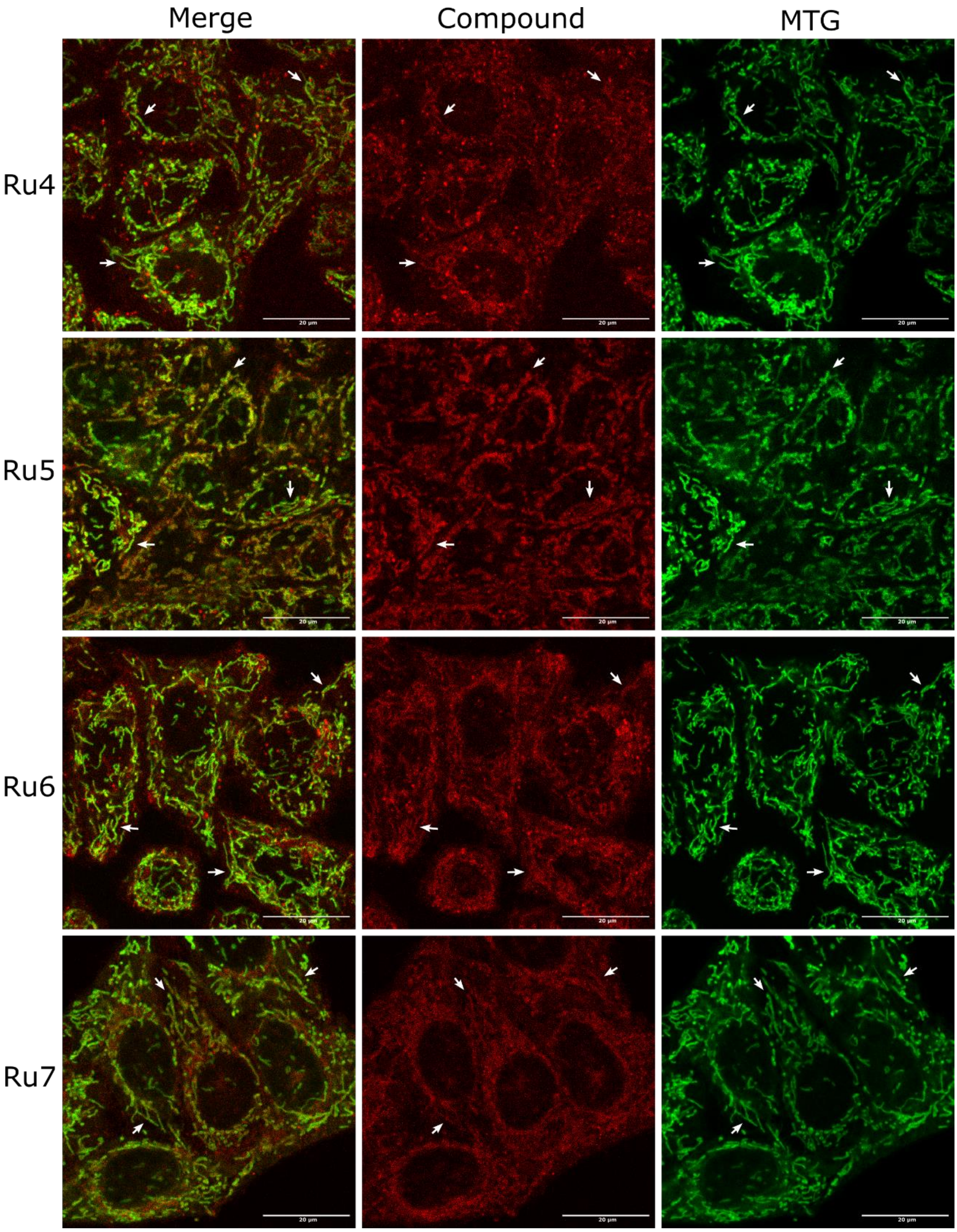

Figure S48. **Co-localization studies of Ru-COUBPY complexes Ru4-7 with Mitotracker Green (MTG).** Single confocal planes of HeLa cells incubated with the compounds (10 μM, green) and MTG (0.5 μM, red). Left: Overlay of the two staining. Center: Ru complexes' signal. Right: MTG signal. White arrows point out positive colocalization. Scale bar: 20 μm.

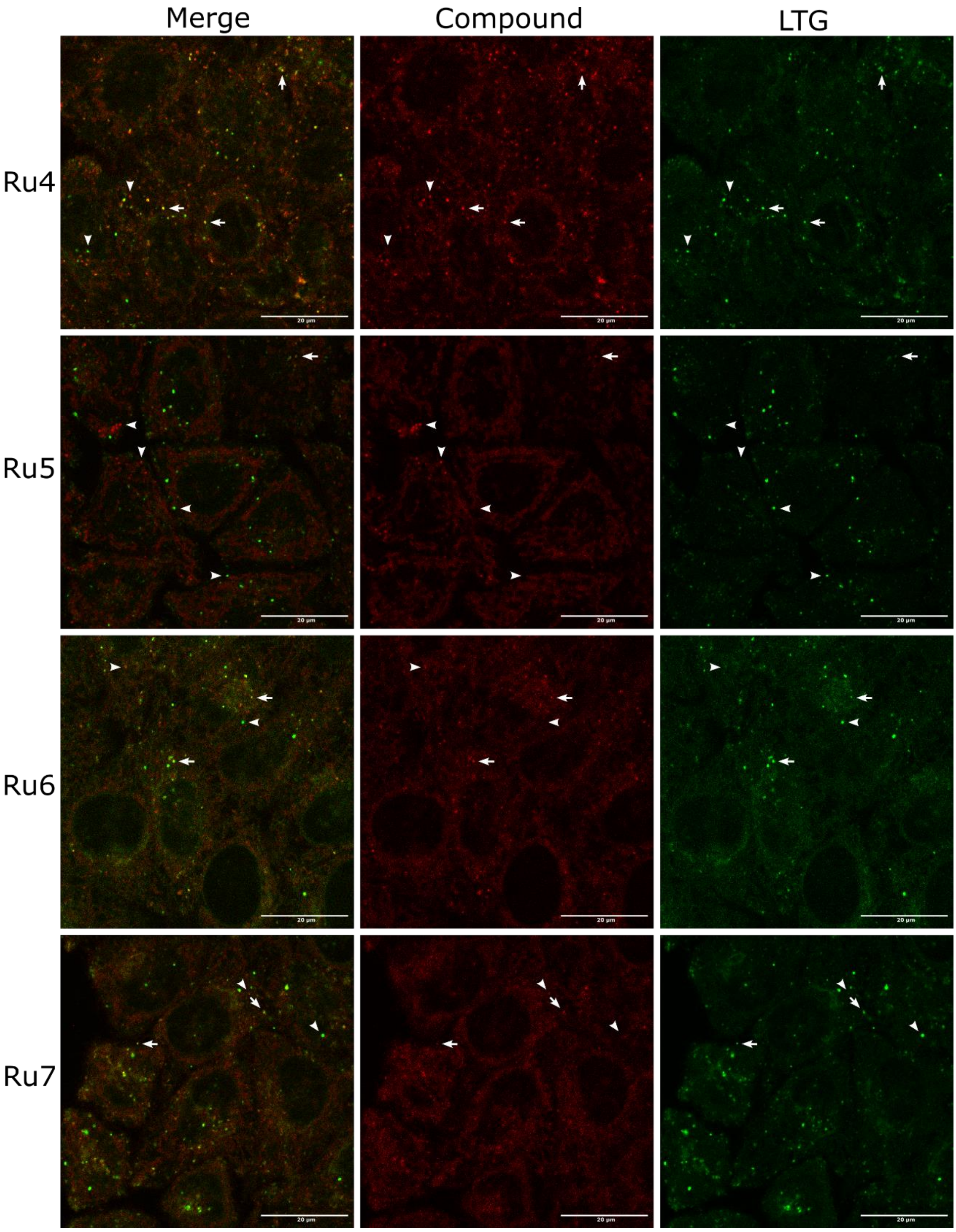

Figure S49. Co-localization studies of Ru-COUBPY complexes **Ru4-7** with Lysotracker Green (LTG). Single confocal planes of HeLa cells incubated with the compounds (10 µM, green) and LTG (0.5 µM, red). Left: Overlay of the two staining. Center: Ru complexes' signal. Right: LTG signal. White arrows and arrowheads point out positive and negative colocalization, respectively. Scale bar: 20 µm.

Table S8. Pearson's correlation coefficient with MTG and LTG.

Compound	MTG	LTG
**Ru4**	0.61	0.57
**Ru5**	0.78	0.28
**Ru6**	0.76	0.47
**Ru7**	0.74	0.44

6. *In vitro* (photo)cytotoxicity determination of Ru-COUBPY complexes

6.1. Cell culture

CT-26 cells were cultured in Dulbecco's Modified Eagle Medium (DMEM; Gibco) supplemented with 10% fetal calf serum, 2 mM L-glutamine, and 100 U/mL penicillin–streptomycin (Gibco). Cells were maintained at 37 °C in a humidified atmosphere with 5% CO_2. For hypoxia experiments, conditions were established by reducing O_2 to a minimum of 2% using nitrogen (N_2) in a Forma™ Steri-Cycle™ i160 incubator (Thermo Fisher Scientific). Cells were maintained under these hypoxic conditions for two weeks prior to experimentation

6.2. ((Photo)cytotoxicity evaluation in cancer cells

The (photo)cytotoxicity of the test compounds under normoxia (21% O_2) was assessed using a fluorometric resazurin-based cell viability assay (Acros Organics). CT-26 cells were seeded in triplicate in 96-well plates at 4000 cells/well in 100 µL of complete medium. After 24 h, cells were treated with serial dilutions of the test compounds (final DMSO ≤ 1%, 0–100 µM) and incubated for 4 h at 37 °C, 5% CO_2 in the dark. The medium was then replaced with fresh medium (100 µL/well), and plates were irradiated for 1 h at 37 °C, 0% CO_2 using 670 nm (3.75 mW/cm^2, 13.5 J/cm^2), 740 nm (3.50 mW/cm^2, 12.6 J/cm^2), or 770 nm (4.25 mW/cm^2, 15.3 J/cm^2) light from a multi-well LED photo-irradiation system (Atlas Photonics Lumos Bio). Treated cells maintained in the dark for 1 h at 37 °C, 0% CO_2 served as controls to evaluate the test compounds' cytotoxicity in the absence of light. After a 44-h recovery period at 37 °C and 5% CO_2, the medium was replaced with complete medium containing resazurin (0.2 mg/mL, 100 µL/well) and cells were incubated for further 4 h under the same conditions. Fluorescence of the resulting resorufin product was then measured (λ_{ex} = 540 nm, λ_{em} = 590 nm) using an Infinite 200 PRO microplate reader (TECAN). IC_{50} values were then calculated based on the inhibitory rate curves using the following equation:

$$I = \frac{I_{max}}{1 + \left(\frac{IC_{50}}{C}\right)^n}$$

where *I* represent the percentage inhibition of viability observed, I_{max} is the maximal inhibitory effect, IC_{50} is the concentration that inhibits 50% of maximal growth, *C* is the concentration of the treatment and *n* is the slope of the semi-logarithmic dose-response sigmoidal curves. The non-linear fitting was performed using GraphPad Prism software. All experiments were performed in three independent studies with triplicate points per concentration level.

The (photo)cytotoxicity of the test compounds under hypoxia (2% O_2) was assessed using a fluorometric resazurin-based cell viability assay (Acros Organics). CT-26 cells were seeded in triplicate in 96-well plates at a density of 2500 cells/well in a final volume of 100 μL/well and incubated at 2% O_2. A flask containing the corresponding cell culture medium was also pre-equilibrated in the incubator at 2% O_2. After 48 h in the hypoxia incubator, cells were treated with serial dilutions of the test compounds (final DMSO ≤ 1%, 0–100 μM) and incubated for 4 h at 37 °C, 5% CO_2 in the dark. The medium was then replaced with fresh medium (100 μL/well), and plates were irradiated for 1 h at 37 °C, 0% CO_2 using 670 nm (3.75 mW/cm^2, 13.5 J/cm^2) or 740 nm (3.50 mW/cm^2, 12.6 J/cm^2) light from a multi-well LED photo-irradiation system (Atlas Photonics Lumos Bio). Irradiation was performed inside a Plas-Labs 856-Series Glove Box equipped with an O_2 concentration sensor, with the atmosphere maintained at 2% O_2 using a controlled nitrogen flow. Treated cells maintained in the dark for 1 h at 37 °C in the hypoxia incubator served as controls to evaluate the test compounds' cytotoxicity in the absence of light. After a 44-h recovery period at 37 °C and 5% CO_2, the medium was replaced with complete medium containing resazurin (0.2 mg/mL, 100 μL/well) and cells were incubated for further 4 h under the same conditions. Fluorescence of the resulting resorufin product was then measured (λ_{ex} = 540 nm, λ_{em} = 590 nm) using an Infinite 200 PRO microplate reader (TECAN). IC_{50} values were then calculated as indicated above.

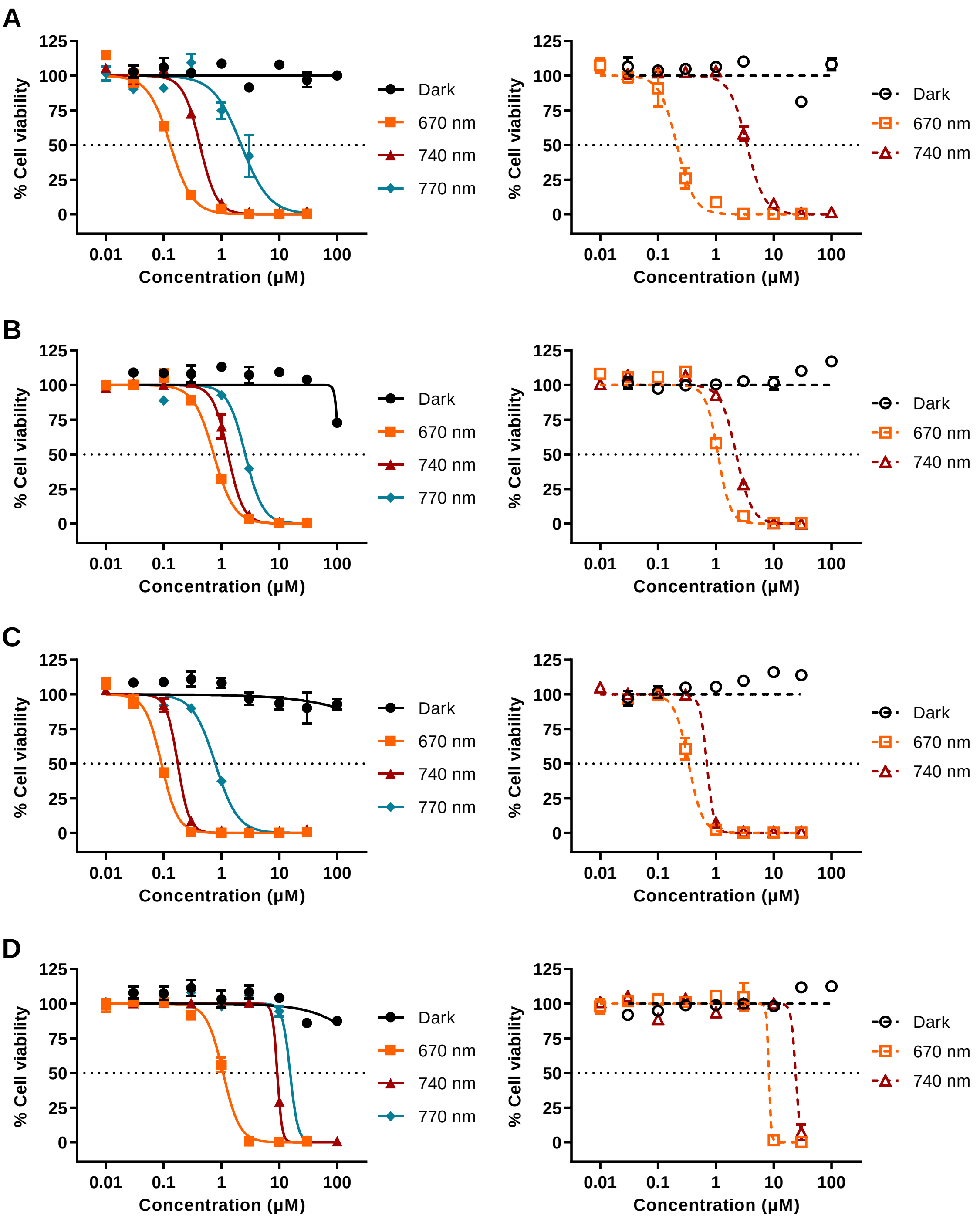

Figure S50. Dose-response curves from the chromatic (photo)cytotoxicity screening of **Ru4-7** complexes (A-D, respectively) in CT-26 cells under normoxic (21% O_2, left panels) and hypoxic (2% O_2, right panels) conditions. Irradiation was performed for 1 h using deep-red or NIR light at the following parameters: 670 nm (3.75 mW/cm², 13.5 J/cm²), 740 nm (3.50 mW/cm², 12.6 J/cm²), and 770 nm (6.75 mW/cm², 24.3 J/cm²).

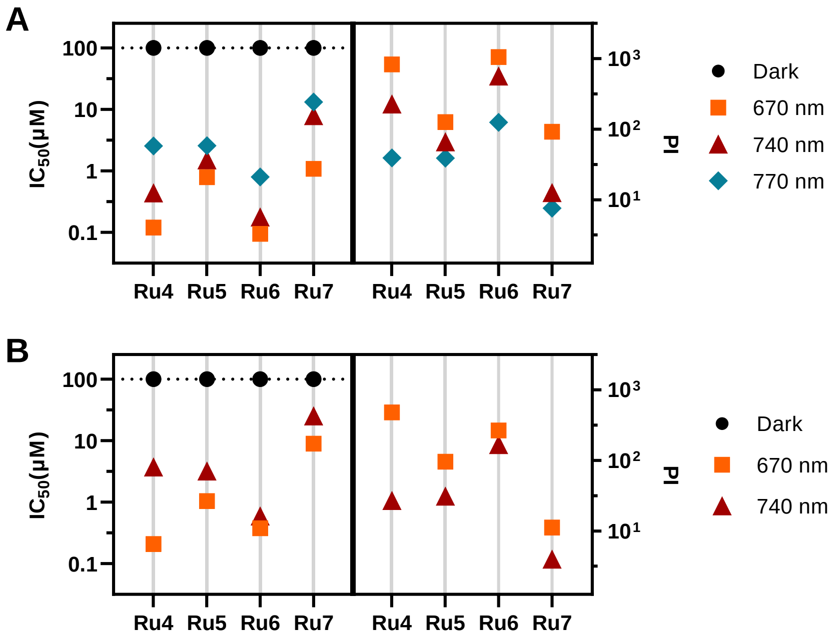

Figure S51. Activity plots showing the chromatic (photo)cytotoxicity screening of compounds **Ru4-7** in CT-26 cells in the dark and under deep-red (670 nm, 13.5 J cm^{-2}) or NIR (740 nm, 12.6 J cm^{-2} or 770 nm 24.3 J cm^{-2}) light irradiation, under (A) normoxic (21% O_2) and (B) hypoxic (2% O_2) conditions. The left panels display IC_{50} values, while the right panels depict the corresponding phototherapeutic indexes (PIs). For dark conditions, IC_{50} values exceeded 100 µM and could not be accurately determined within the tested concentration range; the plotted values correspond to the highest tested concentration (100 µM) and are shown for completeness and comparative purposes. Detailed IC_{50} values (mean ± SD) and PIs are reported in Tables 3 and 4.

6.3. Cellular accumulation by ICP-MS.

CT-26 cells were seeded in a 6-cm cell culture dish at a density of $2x10^6$ cells/dish and were incubated at 37 °C, 5% CO_2 for 24 h. The medium was replaced with 5 mL of a 5-μM dilution of the corresponding Ru-COUBPY complex diluted in fresh DMEM from a 10 mM stock solution in DMSO (DMSO <1 %). After 4 h incubation at 37 °C, cells were washed twice with PBS (1x), trypsinized, collected, counted and the resulting cell pellets were dried overnight in an oven at 37 ºC and stored in a freezer at –80 °C. ICP-MS samples were prepared as follows: cell pellets (~$1.5x10^6$ cells/sample) were digested using 70% nitric acid (500 μL, 65 °C, overnight) and then further diluted 1:100 using a 1% (w/v) solution of HCl in Milli-Q H_2O. All ICP-MS samples were measured using a NexIOn2000 (Perikin Elmer) quadrupole ICP-MS instrument from the CCITUB, Barcelona. The monitored isotopes were ^{100}Ru and ^{101}Ru. Throughout the course of the analytical sequence, an indium internal standard was injected after in-line mixing with the samples to correct for signal drift and matrix effects. A set of calibration standards were analyzed to confirm and model (through simple linear regression) the linear relationship between signal and concentration. The model was then used to convert measured sample counts to concentrations. Reported uncertainties were calculated using error propagation equations and considering the combination of standard deviation on replicated consecutive signal acquisitions (n=3), internal-standard ratio, and blank subtraction. The non-linear term (internal-standard ratio) was linearized using a first-order Taylor series expansion to simplify error propagation. The amount of metal detected in the cell samples was transformed from ppb to ng of Ru/mL. Data were normalized to the number of cells and expressed as ng of Ru/10^6 cells. Data are expressed as the mean ± SD of one independent experiment with n = 3 replicates.

6.4. Cell apoptosis assay.

Apoptosis was assessed using the Annexin V-FITC/Propidium iodide (AV/PI) dual staining method. Briefly, CT-26 cells (1×10^5 cells/well) were seeded in 12-well plates and incubated overnight at 37 °C. Compounds were tested at a concentration equivalent to twice their IC_{50} values. After 4 h of incubation, the medium was replaced with fresh medium, followed by 1 h of irradiation at either 660 nm (7.7 ± 0.8 mW/cm^2, 27.7 J/cm^2) or 760 nm (6.3 ± 0.4 mW/cm^2, 22.7 J/cm^2). Irradiated, non-treated cells served as the control group. Following a 24 h drug-free recovery period, cells were harvested by trypsinization, washed with PBS and centrifuged. The resulting the pellets were resuspended in 100 μL of the Annexin V binding buffer, following the manufacturer's instructions (Invitrogen). The resuspended cell solution was

incubated at room temperature in the dark for 15 min prior to flow cytometry analysis (Gallios, Beckman Coulter; 10^4 events acquired per sample). Cells were visualized using λ_{exc} = 488 channels, and emission was detected at 525 nm (Annexin V, FL1 channel) and 620 nm (propidium iodide, FL3 channel). Cell populations were classified as follows: AV^-/PI^- (live cells); AV^-/PI^+ (necrotic cells); AV^+/PI^- (early apoptotic cells) and AV^+/PI^+ (late apoptotic cells). Data were analyzed using FlowJo software. Three independent experiments were conducted for each treatment condition.

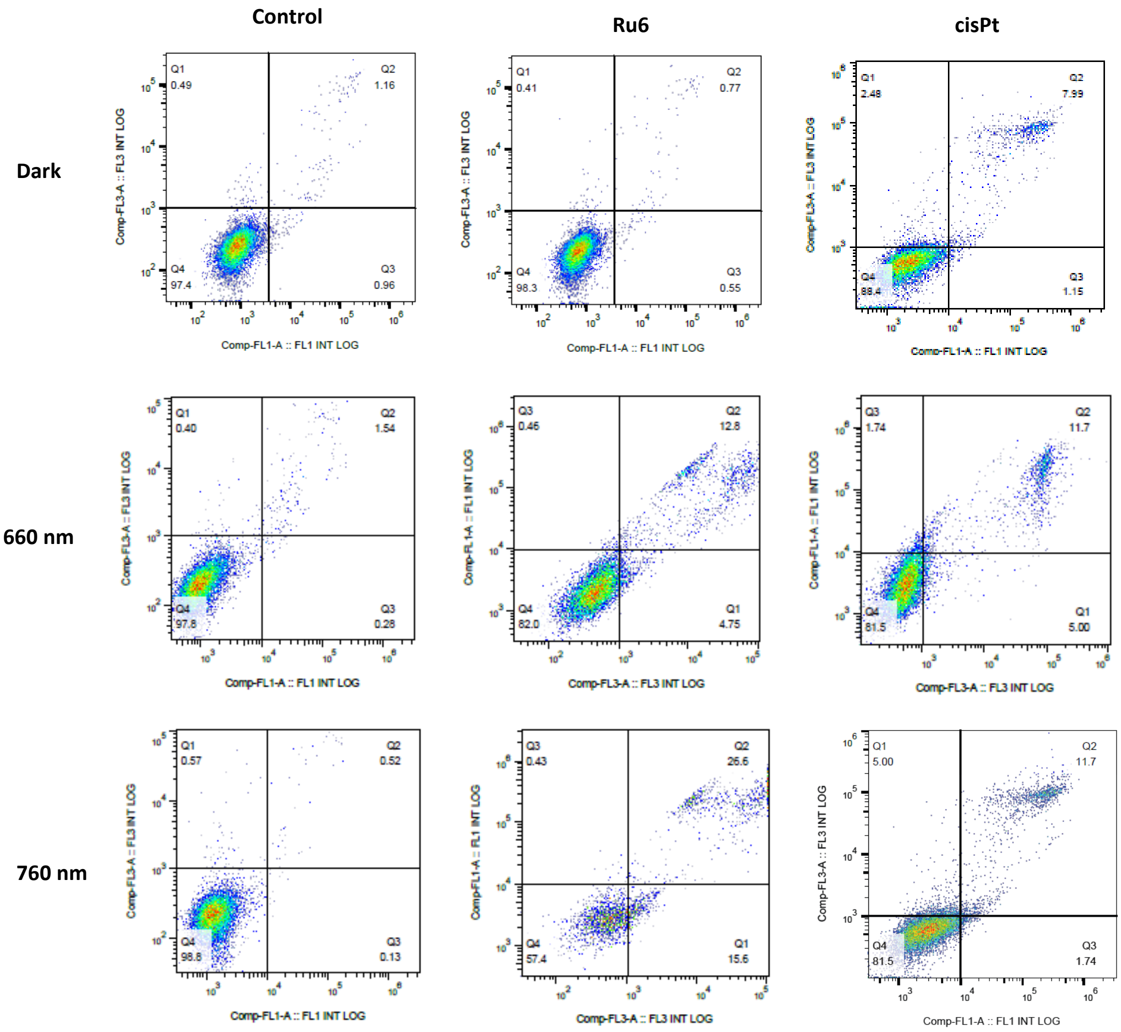

Figure S52. Representative replicate dot plots of CT-26 cells double-stained with annexin V-FITC (FL1) and propidium iodide (FL3), following treatment with **Ru6** (4 h incubation at 2×IC_{50} concentration), subsequent 1 h irradiation at 660 nm (7.7 ± 0.8 mW/cm^2, 27.7 J/cm^2) or 760 nm (6.3 ± 0.4 mW/cm^2, 22.7 J/cm^2), and a 24 h recovery period.

6.5. Ferroptosis assay.

CT-26 cells were seeded in 96-well plates at a density of 4.5x10^3 cells per well and incubated at 37 °C, 5% CO_2 for 24 h. Cells were incubated first with ferrostatin-1 (Fer-1) or deferoxamine (DFO) at 50 μM previous to co-incubation with **Ru6** (0.001-100 μM). After 4 h, the medium was replaced with 100 μL of fresh medium and cells were irradiated for 1 g at 660 nm (7.7 ± 0.8 mW/cm^2, 27.7 J/cm^2) or 760 nm (6.3 ± 0.4 mW/cm^2, 22.7 J/cm^2). Following irradiation, cells were incubated for an additional 44 h. To assess cell viability, 30 μL of freshly prepared resazurin solution (0.2 mg/mL) was added to each well and incubated for 4 h Resazurin fluorescence was measured at λ_{exc} = 520 nm and λ_{em} = 580-640 nm using a Glomax Plate Reader (Promega Biotech Ibérica, Spain).

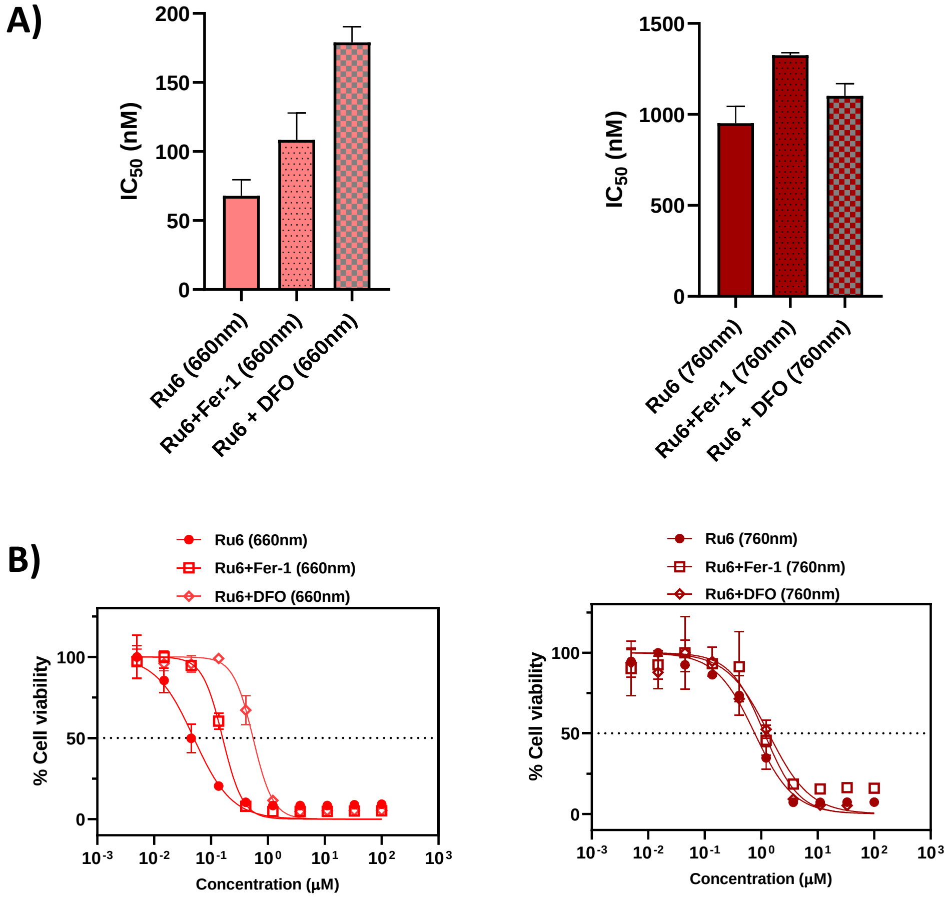

Figure S53. A) IC_{50} values (μM) of **Ru6** complex in CT-26 cells with or without preincubation with the ferroptosis inhibitors ferrostatin-1 (Fer-1, 50 μM) or deferoxamine (DFO, 50 μM). B) Dose-response curves of **Ru6** in the presence or absence of Fer-1 or DFO, following 1 h irradiation at either 660 nm (7.7 ± 0.8 mW/cm^2, 27.7 J/cm^2) or 760 nm (6.3 ± 0.4 mW/cm^2, 22.7 J/cm^2).

Table S9. IC_{50} values of **Ru6** in CT-26 cells after irradiation at 660 nm (7.7 ± 0.8 mW/cm^2, 27.7 J/cm^2) or 760 nm (6.3 ± 0.4 mW/cm^2, 22.7 J/cm^2), with (+) or without (-) preincubation with Fer-1 or DFO (50 µM). Data represent mean ± SD from three independent experiments.

	**660 nm**			**760 nm**		
	-	**+ Fer-1**	**+ DFO**	-	**+ Fer-1**	**+ DFO**
**IC_{50} (µM)**	0.068 ± 0.01	0.108 ± 0.019	0.179 ± 0.025	0.952 ± 0.092	1.325 ± 0.014	1.102 ± 0.116

7. *In vivo* PDT efficacy study of Ru6 in BALB/c mice bearing subcutaneous CT-26 syngeneic colon tumors

7.1. Ethical animal procedures and animal housing conditions

All animal procedures were conducted in compliance with local, national and European regulations on animal welfare. The study procotol was reviewed and approved by the Ethical Committee for Animal Experimentation of the Parc Científic de Barcelona (PCB, Barcelona, Spain), under authorization number 23-044-P1-(SC). All animals were housed in the PCB's accredited animal facility, which is registered with the regional competent authority for the care and use of animals in scientific procedures (registration number B-9900044), as well as with the national competent authority for the use of genetically modified organisms (authorization A/ES/16/1-03; notification number 65-7951/2016). Animals were maintained under sterile conditions at a constant temperature of 20-22°C and relative humidity (45-65%), with a 12-h light/dark cycle. All handling procedures were performed under a laminar flow hood. Sterilized water and food were provided *ad libitum*. For identification purposes, a chip was inserted subcutaneously in the dorsal region (upper back) of each mouse.

7.2. Tumor cell line

CT-26 (CT26.WT ATCC ® CRL-2638™), a colon carcinoma cell line, was grown in RPMI-1640 (Thermo Fisher, 42401042), supplemented with 10% FCS (Gibco, 10106-169) at 37°C and 5% CO_2. Cells were expanded when reached 90% confluence. Viability was monitored before and after injection, that should be >90%.

7.3. Formulation of the compound

Complex **Ru6** was dissolved in DMSO and diluted with DMEM to a final concentration of 3.8 mg/mL (1% DMSO). The solution was sonicated at 37 °C for 3 min and vortexed to ensure homogenization. The vial was kept at 37 °C until administration.

7.4. Irradiation systems for *in vivo* PDT studies

Two custom-built LED irradiation systems were used, designed and assembled by CD6 (Universitat Politècnica de Catalunya) in collaboration with the Universitat de Barcelona. The first system integrates a Thorlabs M660L4 LED (SN M00929533) driven by a LEDD1B current controller (SN M00936983), delivering a peak emission at 666 nm and a centroid at 660 nm. The second system uses a Thorlabs M780LP1 LED (SN M01210620) with a LEDD1B driver (SN M01221186), with peak emission at 780 nm and centroid at 774 nm. Spectral characterization was performed using CAS140D spectrometers (Instrument Systems GmbH), calibrated against standards traceable to NIST and PTB. The irradiance at the working distance (30 mm) was adjusted to ~100 mW/cm² in both systems.

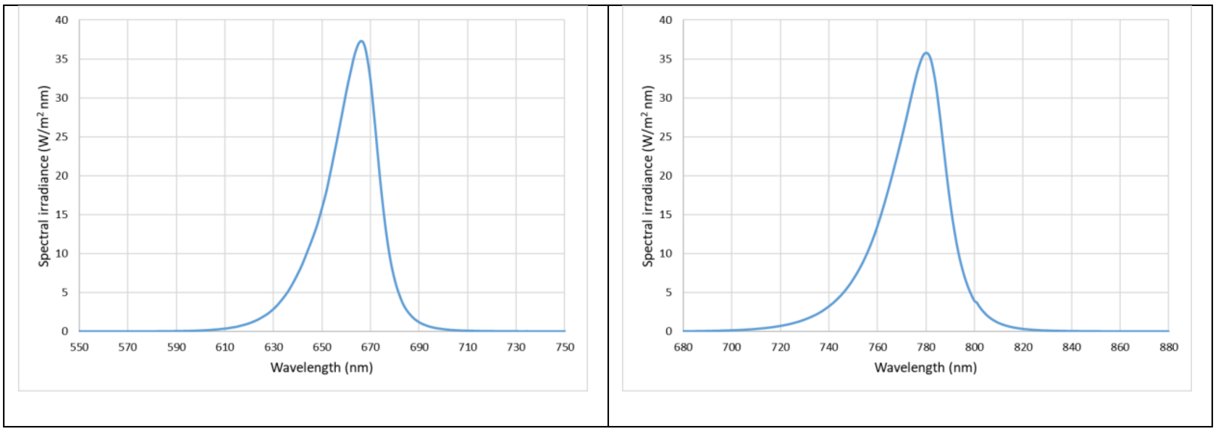

Figure S54. Emission spectra of the LED systems used for *in vivo* PDT at 660 nm (left) and 780 nm (right).

7.5. *In vivo* PDT efficacy study

Eight-week-old female BALB/c mice (Envigo) were subcutaneously inoculated with CT-26 cells (1.15×10^6 cells in 100 µL RPMI) into the right dorsolateral flank on day −10. By day 0, when tumors reached a volume of 50–100 mm³, mice were randomly assigned to experimental groups (n = 4 or 5/group, Table S8). Each mouse received an intratumoral (IT) injection of either vehicle (1% DMSO in DMEM) or **Ru6** (6 mg/kg), as detailed in Table S8. Treatments were administered using a 100 µL Hamilton syringe fitted with a 29G needle, delivering 40 µL over 2 min to ensure homogeneous distribution within the tumor. For light-treated groups, tumors were irradiated 2 min post-injection using either 660 nm or 780 nm light (20 min, 100 mW/cm², 120 J/cm²). Mice were anesthetized with isoflurane throughout the cell inoculation and treatment procedures.

Therapeutic efficacy was monitored by measuring tumor volume (TV) at regular intervals using a caliper. Due to the irregular shape of the tumors, volume was estimated using the formula: $TV = (D \times d^2)/2$, where D and d represent the maximum and minimum tumor diameters, respectively. Tumor progression was expressed as relative tumor volume (RTV), calculated as: $RTV = TV_{day\,x}/TV_{day\,0}$.

To evaluate treatment tolerability, body weight was monitored throughout the study, and animals were carefully observed for any treatment-related clinical signs, including morbidity and mortality. Special attention was given to identifying signs of toxicity, assessing their severity, time of onset, and duration of recovery. Clinical observations included, but were not limited to, the evaluation of skin, fur, eyes, and mucous membranes, as well as respiratory, circulatory, autonomic, and central nervous systems, somatomotor activity, and behavioral patterns. Routine monitoring also involved assessing deviations from normal behavior, such as changes in mobility, apparent food and water intake, eye or hair matting, and the presence of other abnormal signs.

The study was concluded when the average tumor volume in any group reached 1500 mm³. On day 12, mice were euthanized with CO_2, and blood samples were collected via cardiac puncture into pre-labeled microcentrifuge tubes containing EDTA as an anticoagulant. Samples were centrifuged at 5000 rpm for 10 min at 4 °C to obtain plasma, which was subsequently stored at −70 °C until biochemical analysis. Following blood collection, tumors and major organs (bladder, kidneys, and liver) were harvested, fixed, and embedded in paraffin block for histological evaluation. Prior to fixation, tumors were weighed as an additional parameter to

assess treatment efficacy. All experimental data were recorded and analyzed using Prism 9.0 for windows from GraphPad Software Inc.

Table S10. Experimental groups of the *in vivo* PDT efficacy studies.

Group	Item	Conditions	Light Source	Dose/day (mg/kg)	Administration schedule	Irradiation schedule
G1	Vehicle	Dark	-	-	2 times: day 1 and day 3	-
G2	Vehicle	Light	660 nm	-	2 times: day 1 and day 3	2 times: 20 min each after 5 min of administration
G3	**Ru6**	Dark	-	6	2 times: day 1 and day 3	-
G4	**Ru6**	Light	660 nm	6	2 times: day 1 and day 3	2 times: 20 min each after 5 min of administration
G5	Vehicle	Dark	-	-	2 times: day 1 and day 3	-
G6	Vehicle	Light	780 nm	-	2 times: day 1 and day 3	2 times: 20 min each after 5 min of administration
G7	**Ru6**	Dark	-	6	2 times: day 1 and day 3	-
G8	**Ru6**	Light	780 nm	6	2 times: day 1 and day 3	2 times: 20 min each after 5 min of administration
G9	**Ru6**	Dark	-	6	2 times: day 1 and day 3	-
G10	**Ru6**	Light	660 nm	6	2 times: day 1 and day 3	2 times: 20 min each after 5 min of administration
G11	**Ru6**	Light	780 nm	6	2 times: day 1 and day 3	2 times: 20 min each after 5 min of administration

8. ^{1}H and ^{13}C NMR spectra and HR ESI-MS of the compounds

COUBPY ligand 4

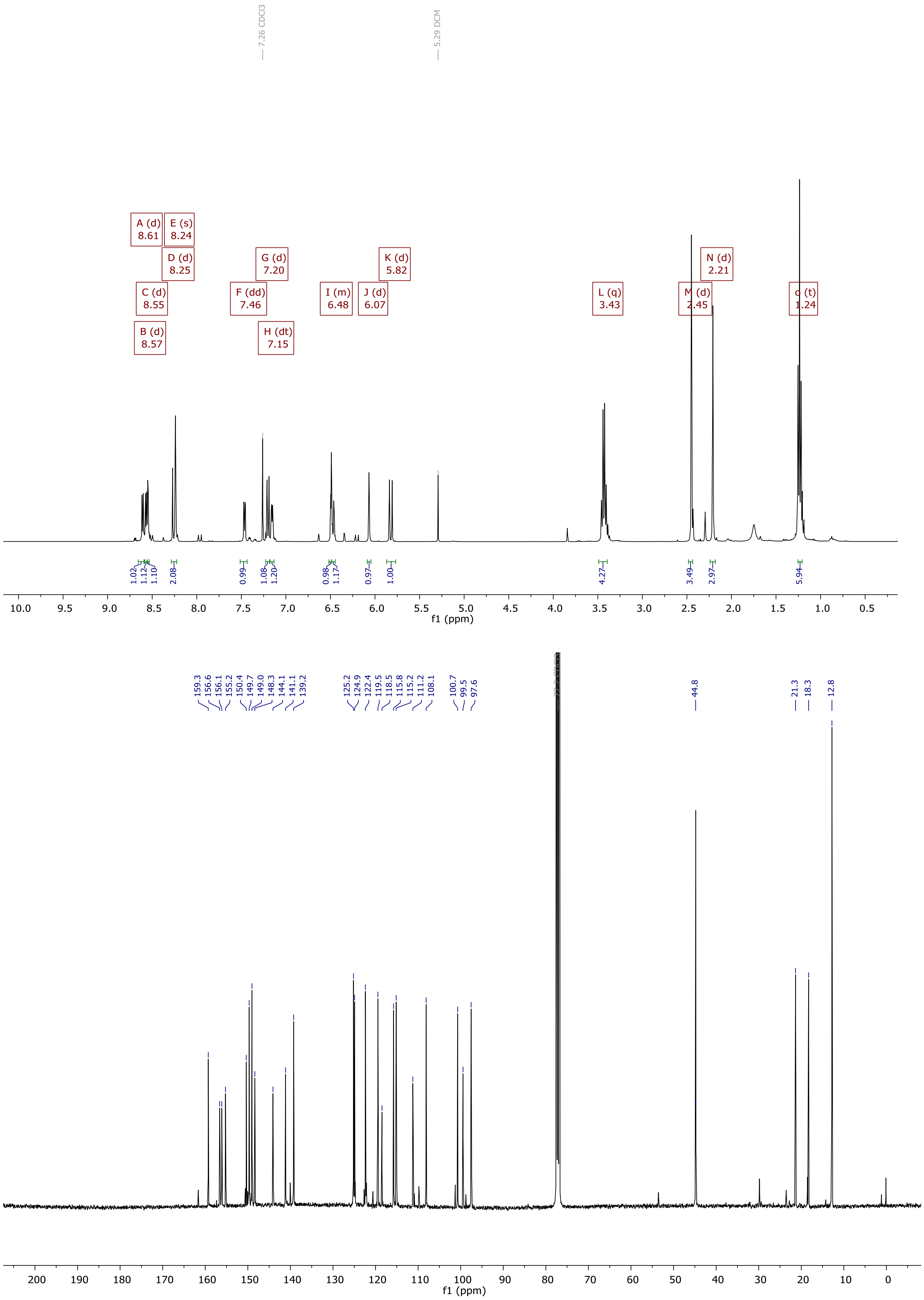

Figure S55. ^{1}H and ^{13}C NMR spectra of COUBPY ligand **4** in $CDCl_3$

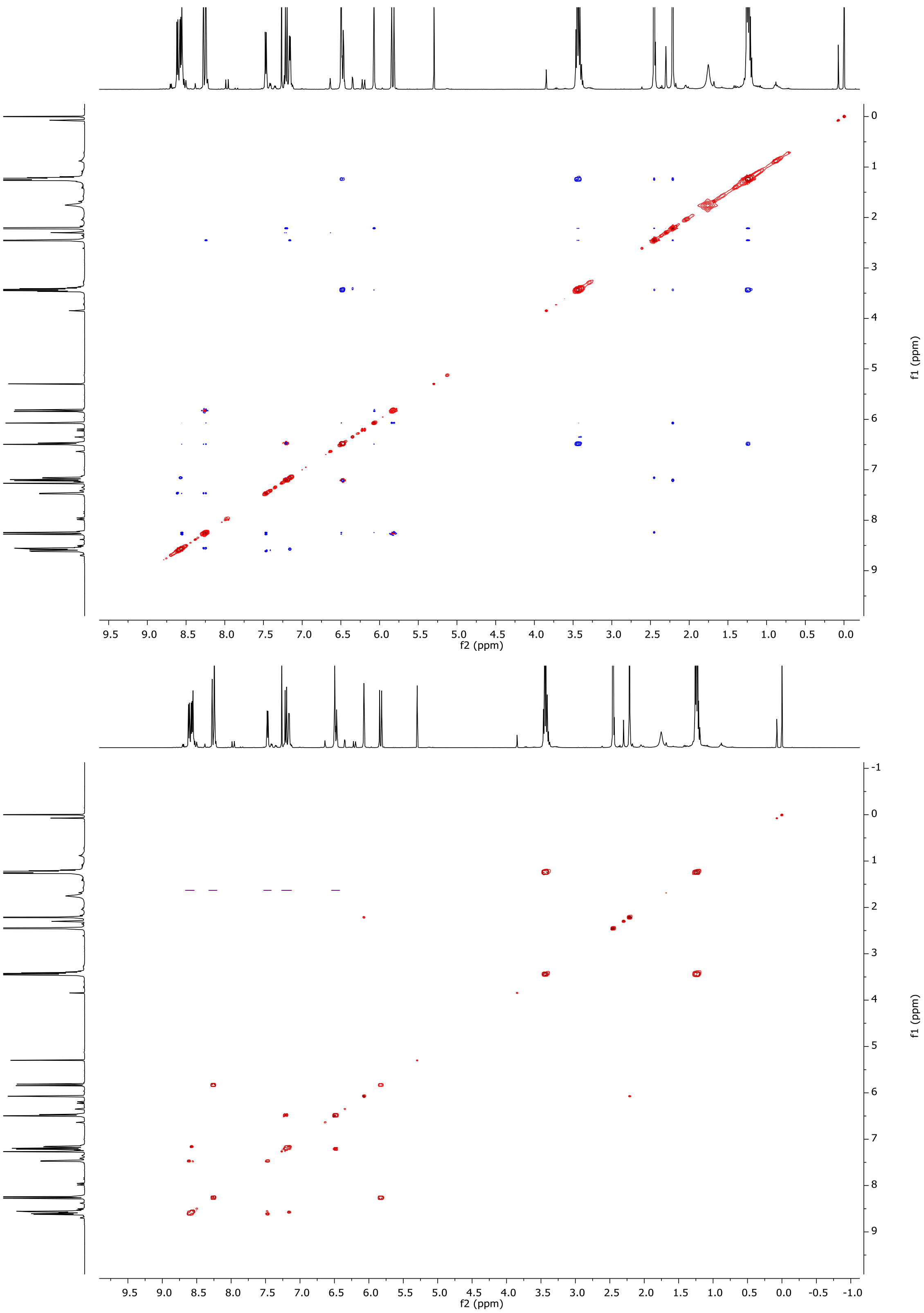

Figure S56. 2D NOESY and 2D COSY spectra of compound COUBPY ligand **4** in $CDCl_3$.

COUBPY ligand 5

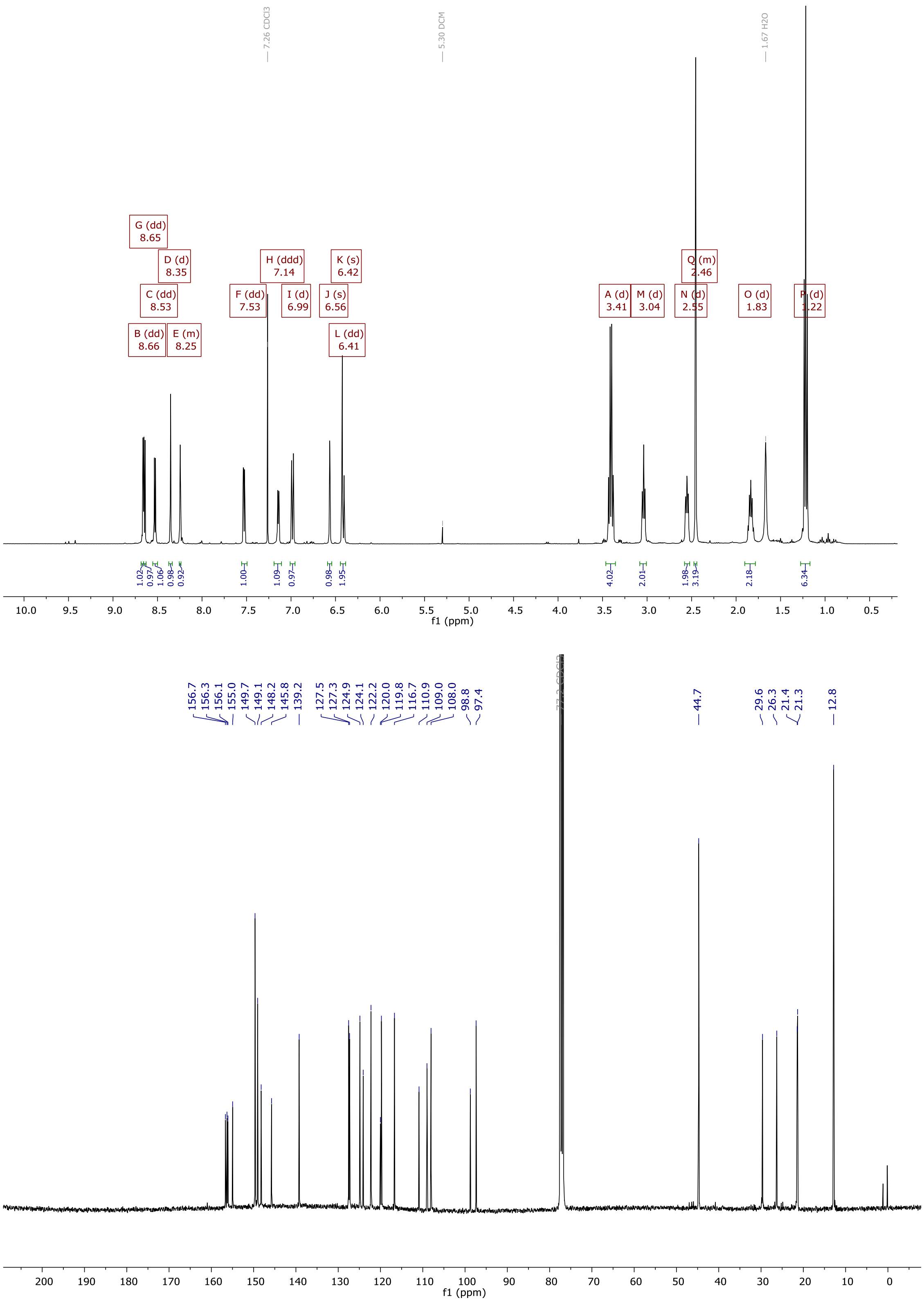

Figure S57. ^{1}H and ^{13}C NMR spectra of **COUBPY5** in $CDCl_3$

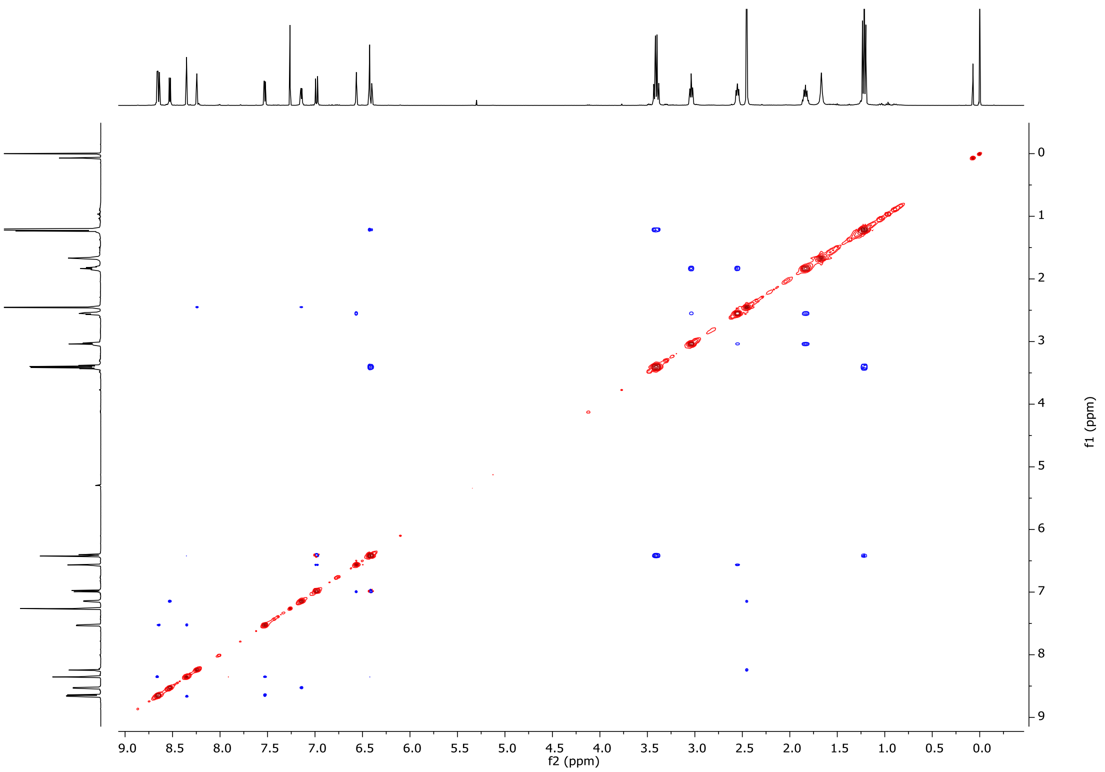

Figure S58. 2D NOESY spectrum of compound COUBPY ligand **5** in $CDCl_3$.

COUBPY ligand 6

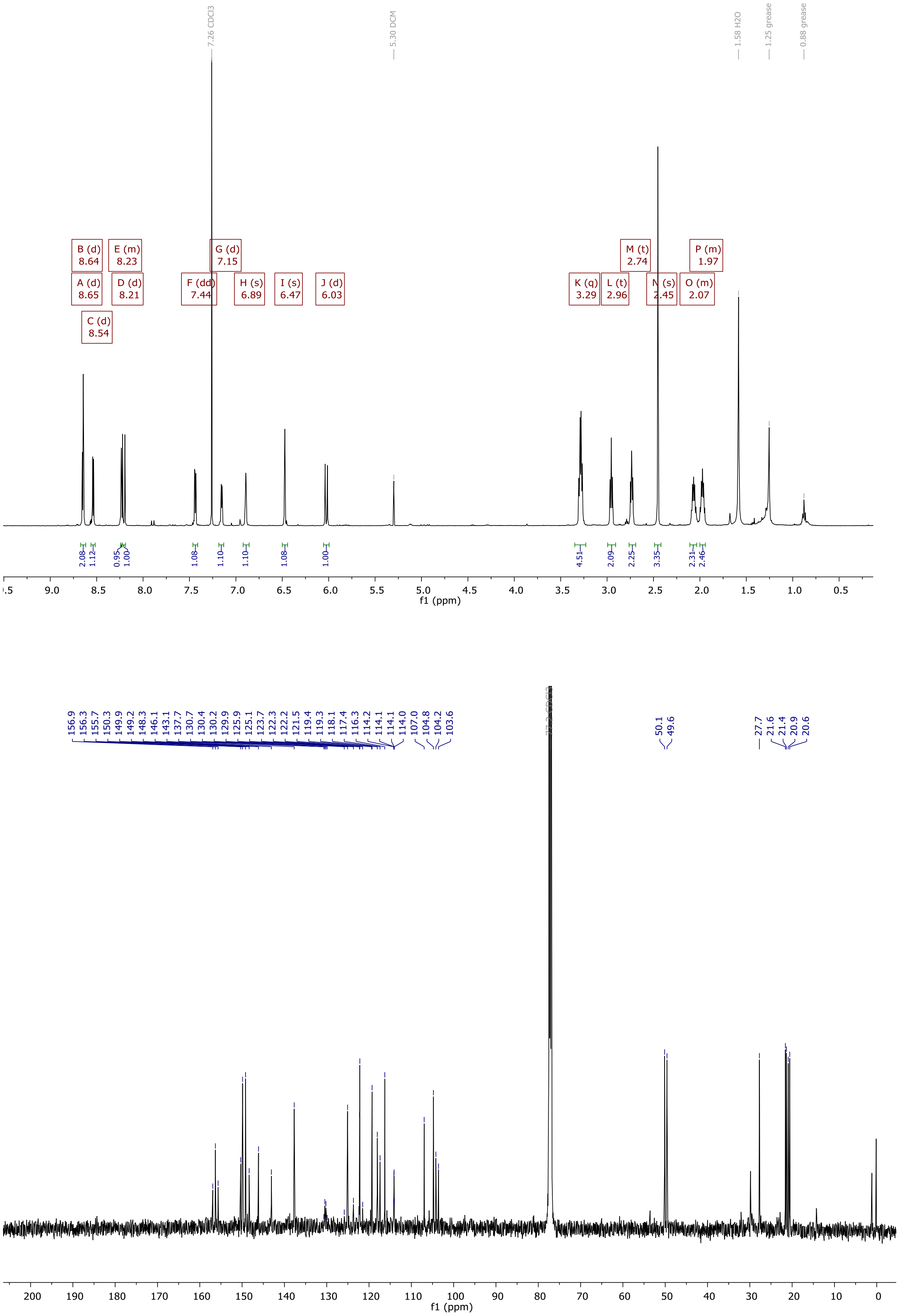

Figure S59. ^{1}H and ^{13}C NMR spectra of COUBPY ligand **6** in $CDCl_3$

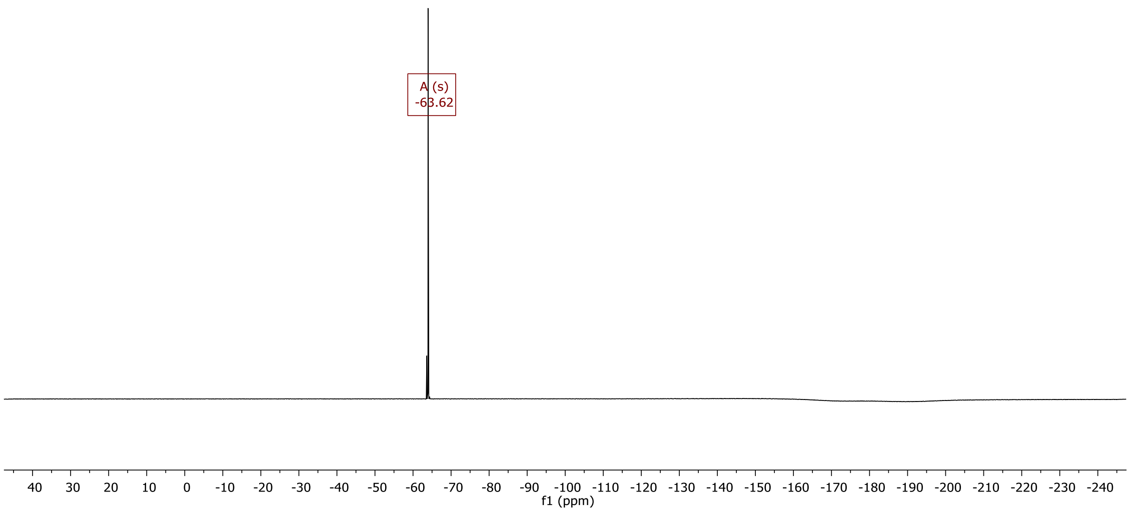

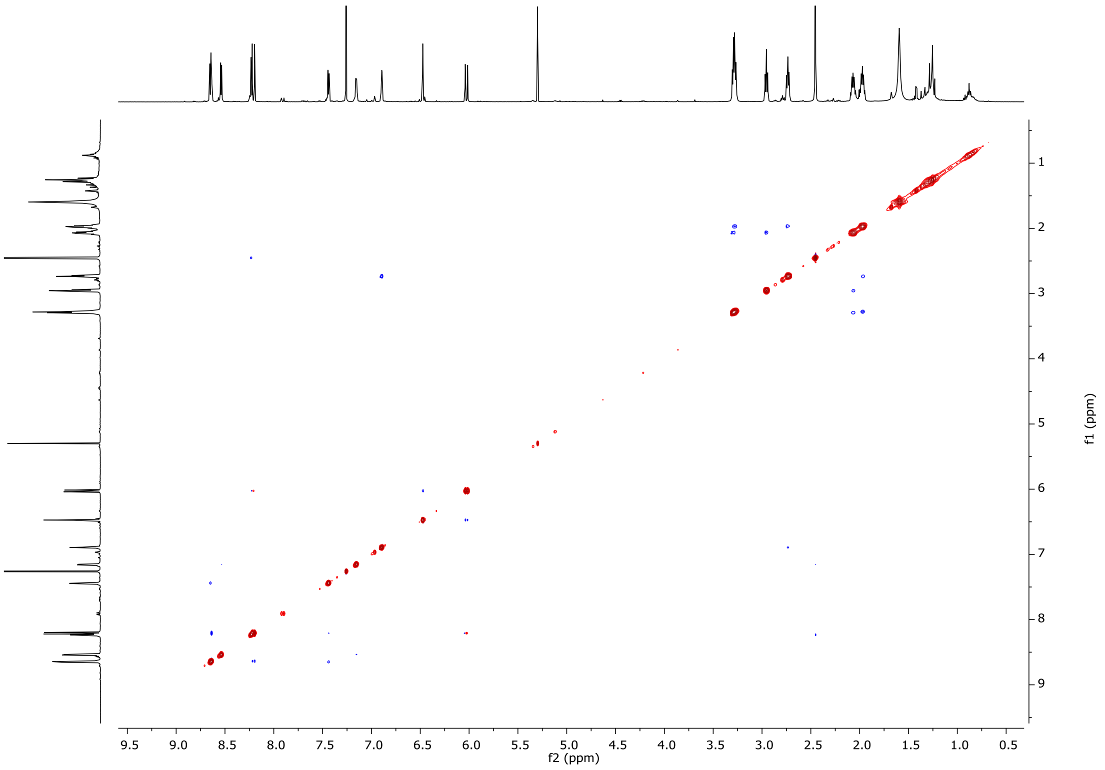

Figure S60. ^{19}F and 2D NOESY spectra of compound COUBPY ligand **6** in $CDCl_3$.

COUBPY ligand 7

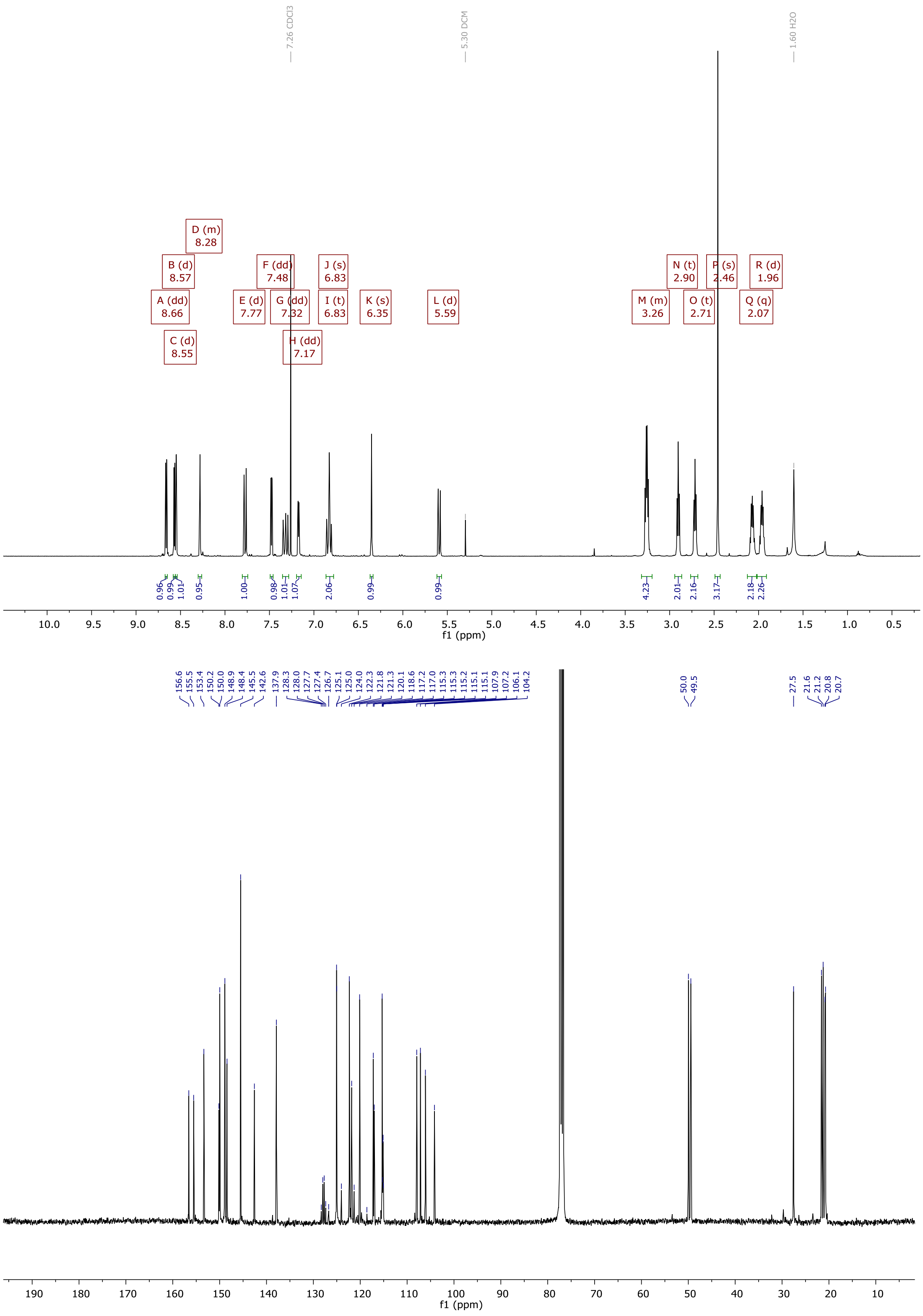

Figure S61. ^{1}H and ^{13}C NMR spectra of COUBPY ligand **7** in $CDCl_3$

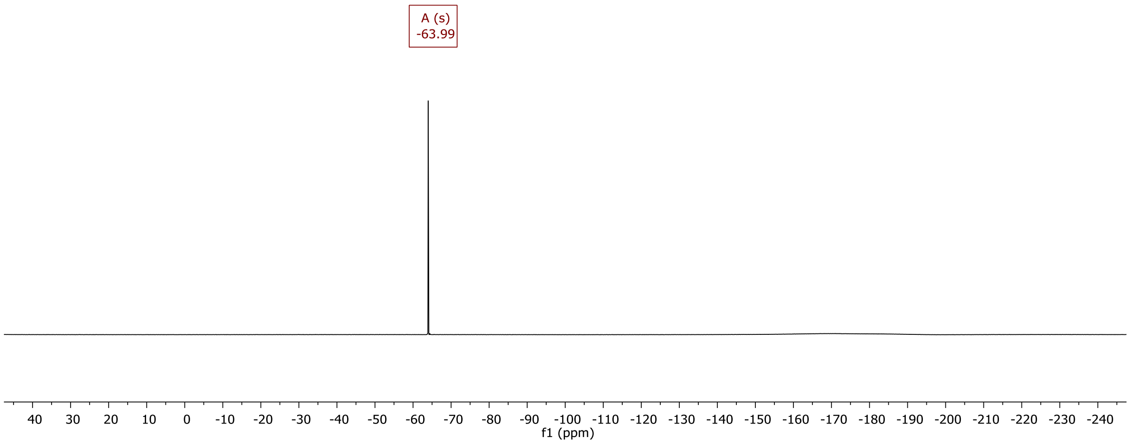

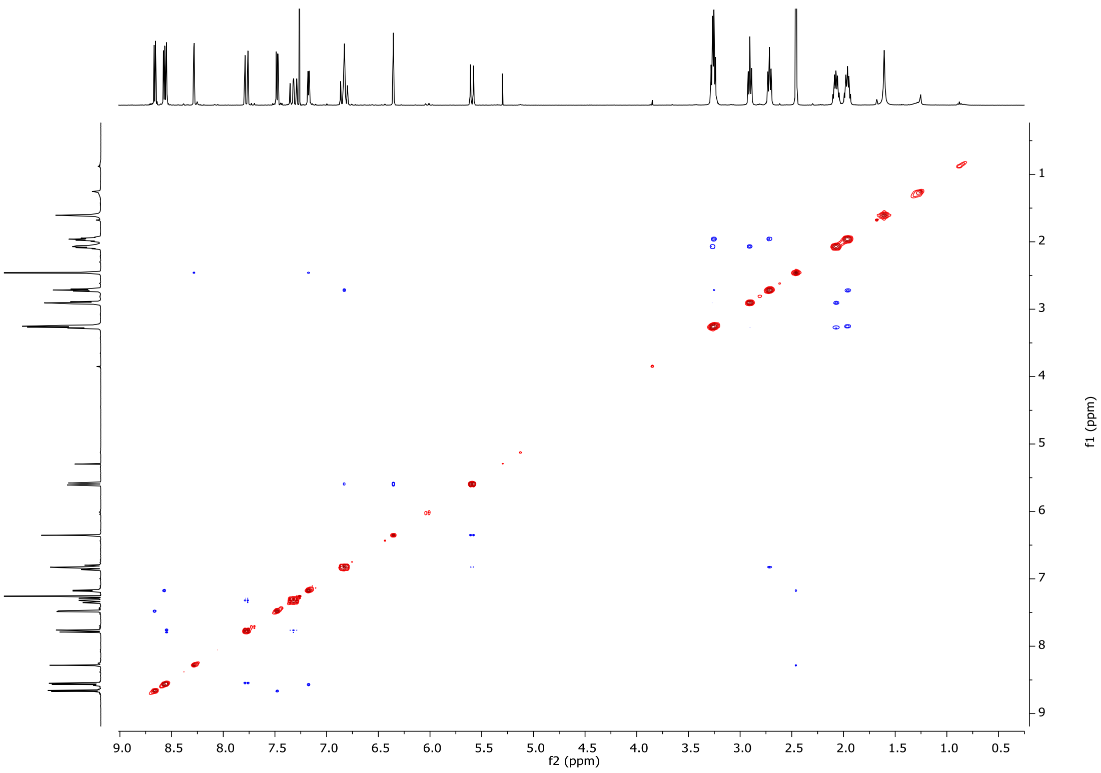

Figure S62. ^{19}F and 2D NOESY spectra of compound COUBPY ligand **7** in $CDCl_3$.

Ru4

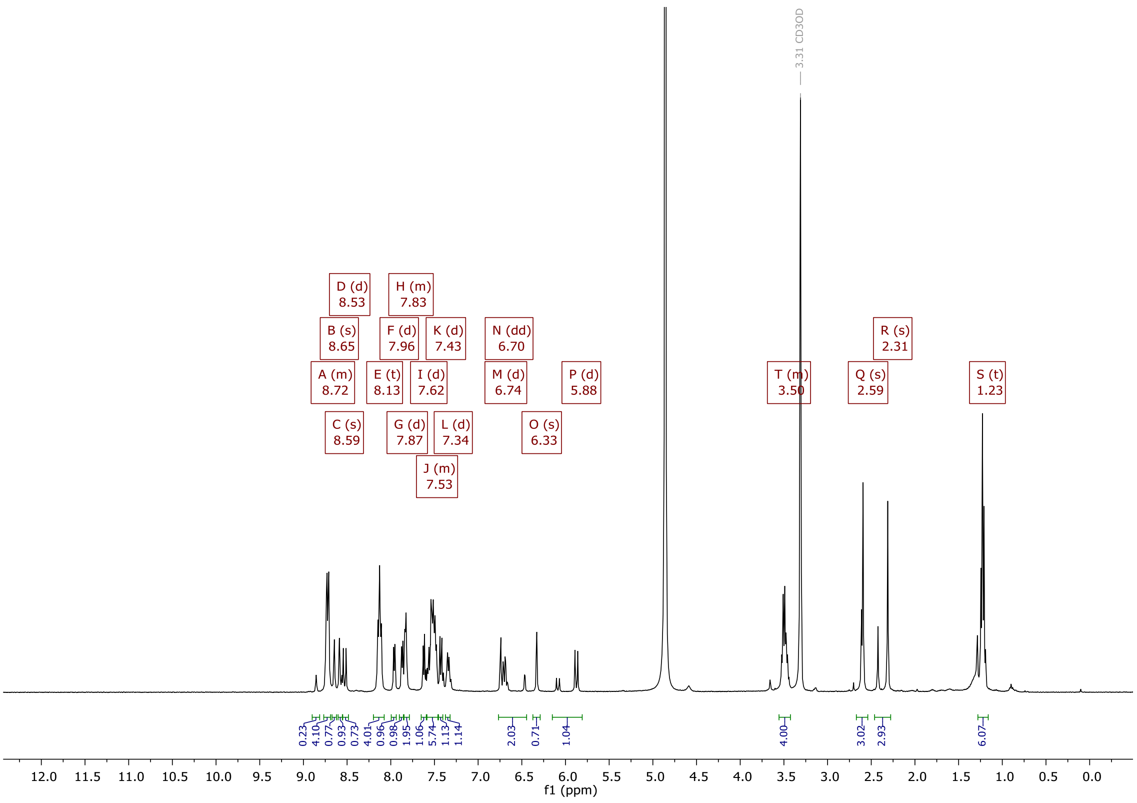

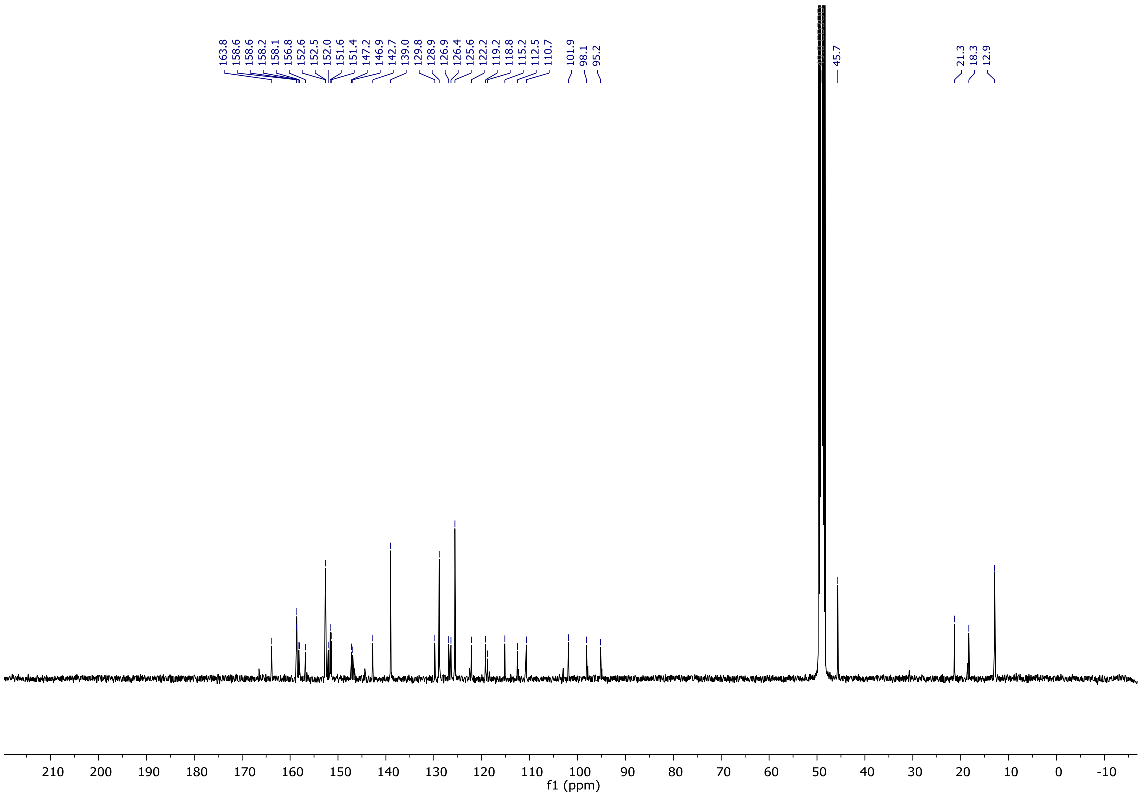

Figure S63. ^{1}H and ^{13}C NMR spectra of **Ru4** in Methanol-*d*$_4$

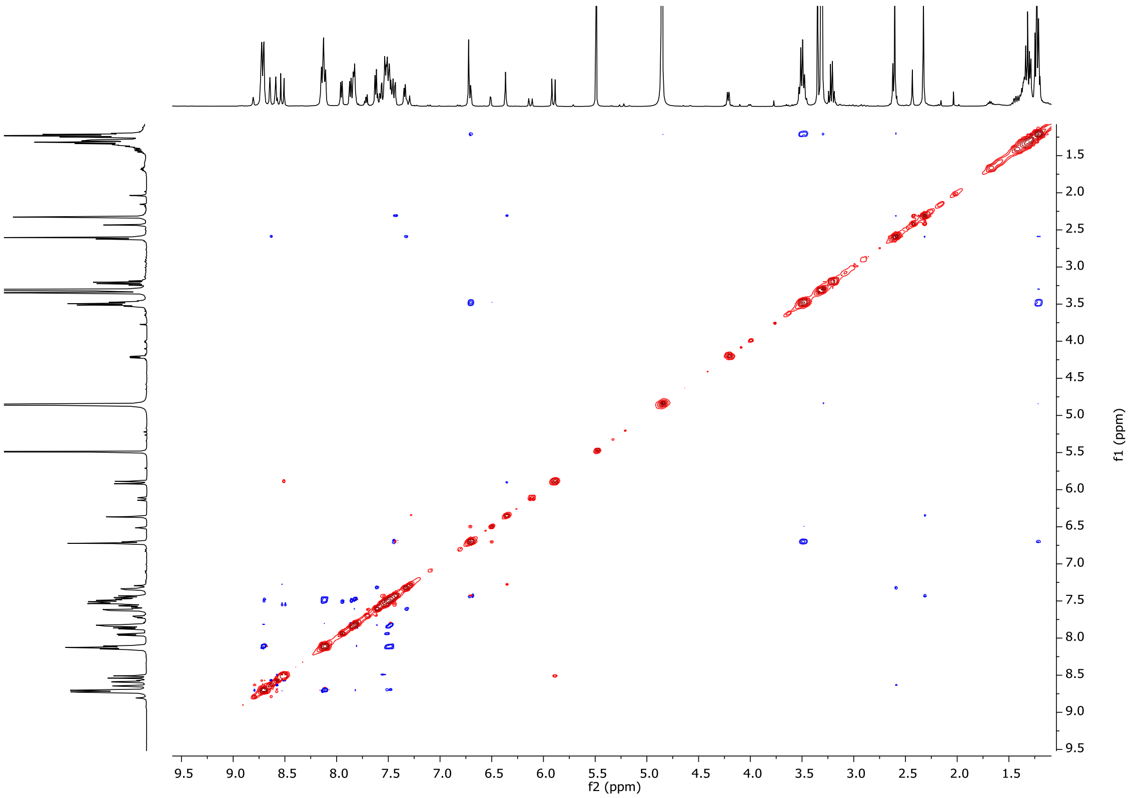

Figure S64. 2D NOESY spectrum of compound **Ru4** in Methanol-d_4.

Ru5

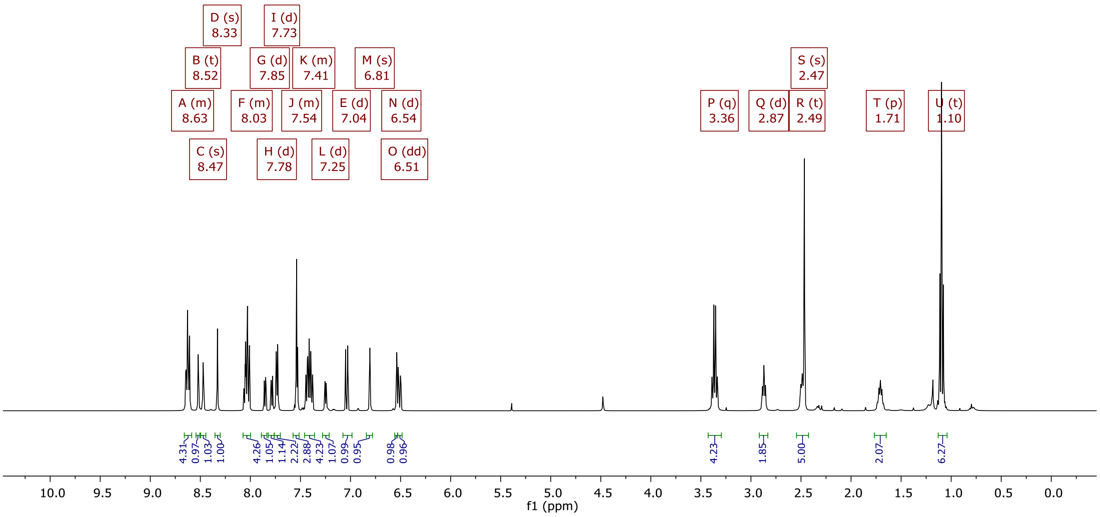

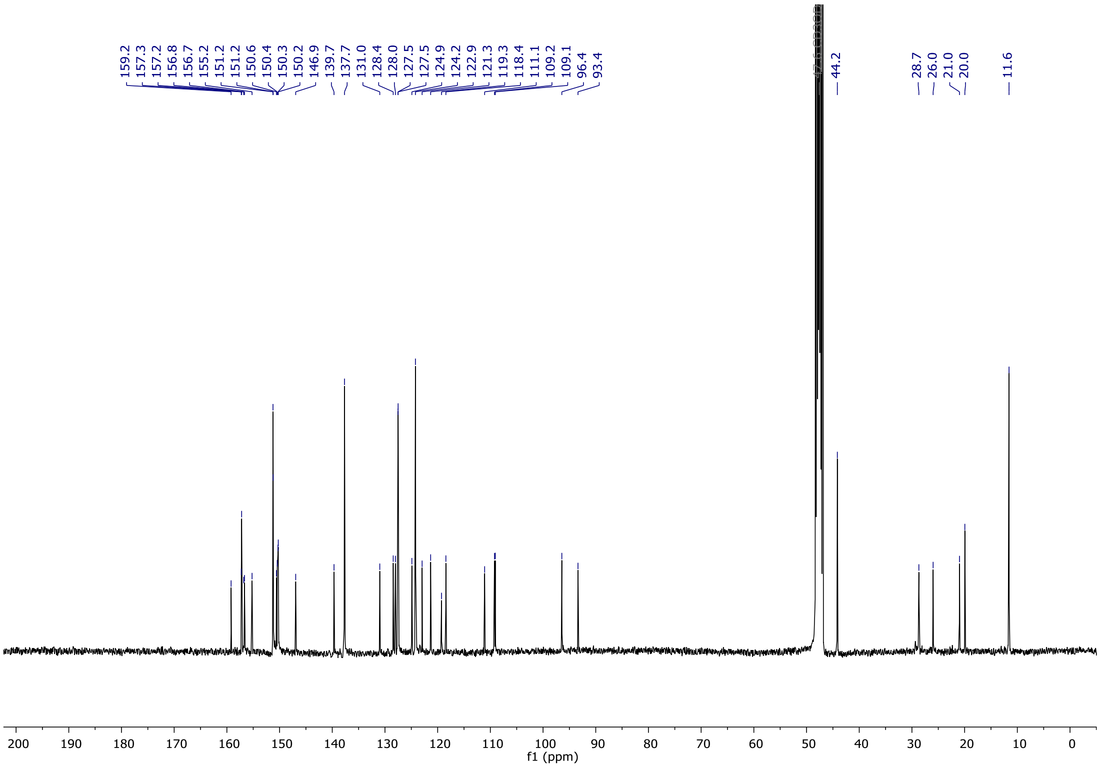

Figure S65. ^{1}H and ^{13}C NMR spectra of **Ru5** in Methanol-d_4

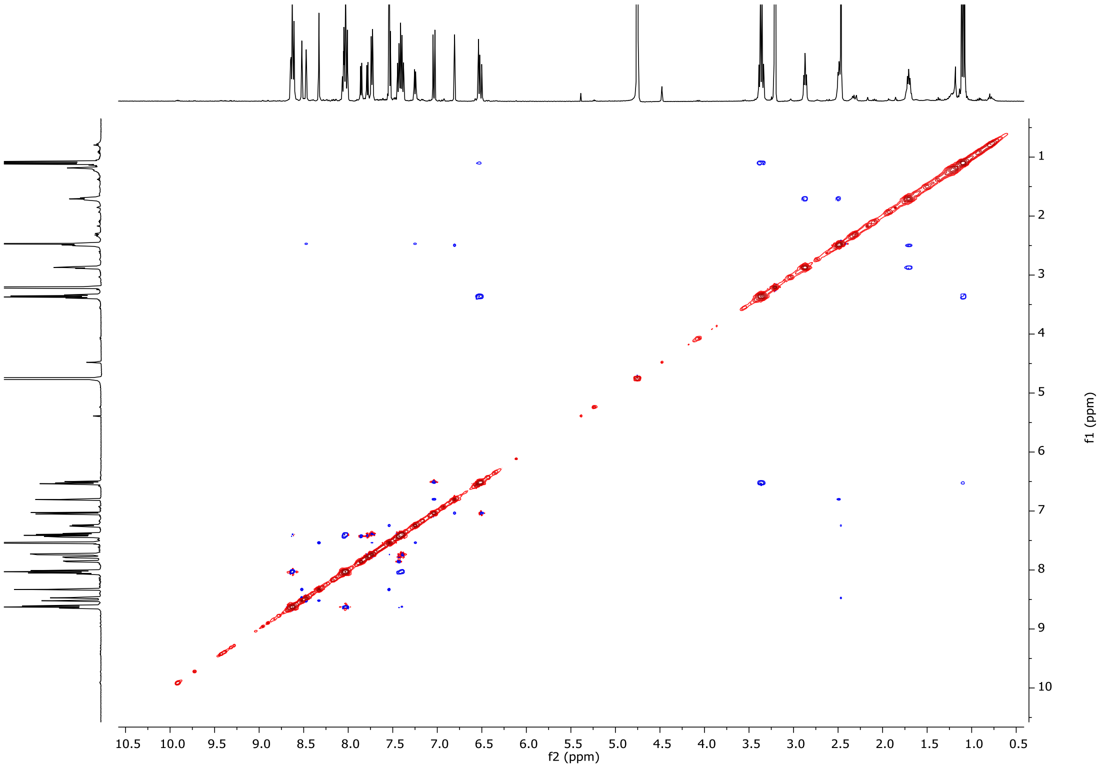

Figure S66. 2D NOESY spectrum of compound **Ru5** in Methanol-*d*$_4$.

Ru6

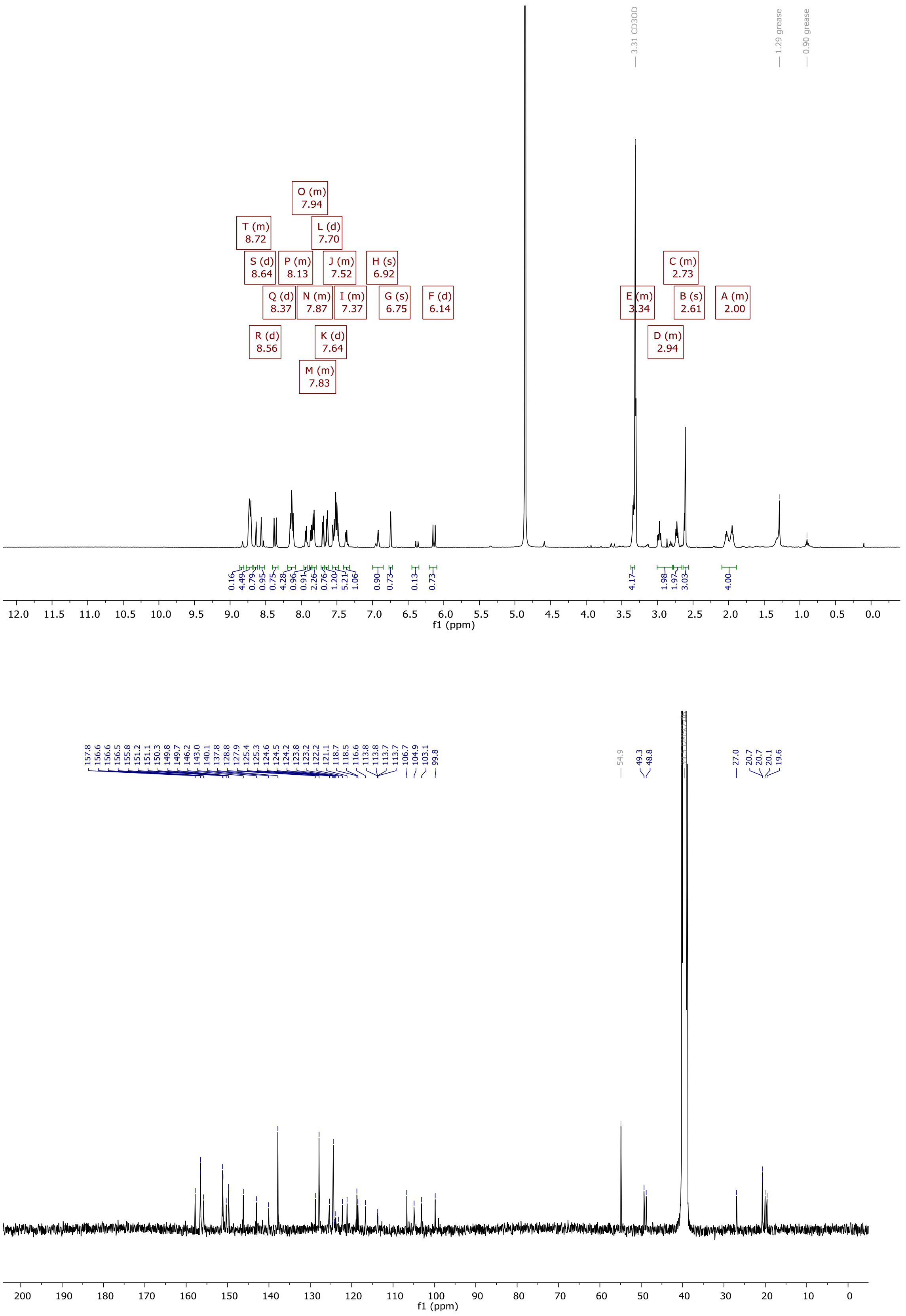

Figure S67. ^{1}H and ^{13}C NMR spectra of **Ru6** in Methanol-d_4

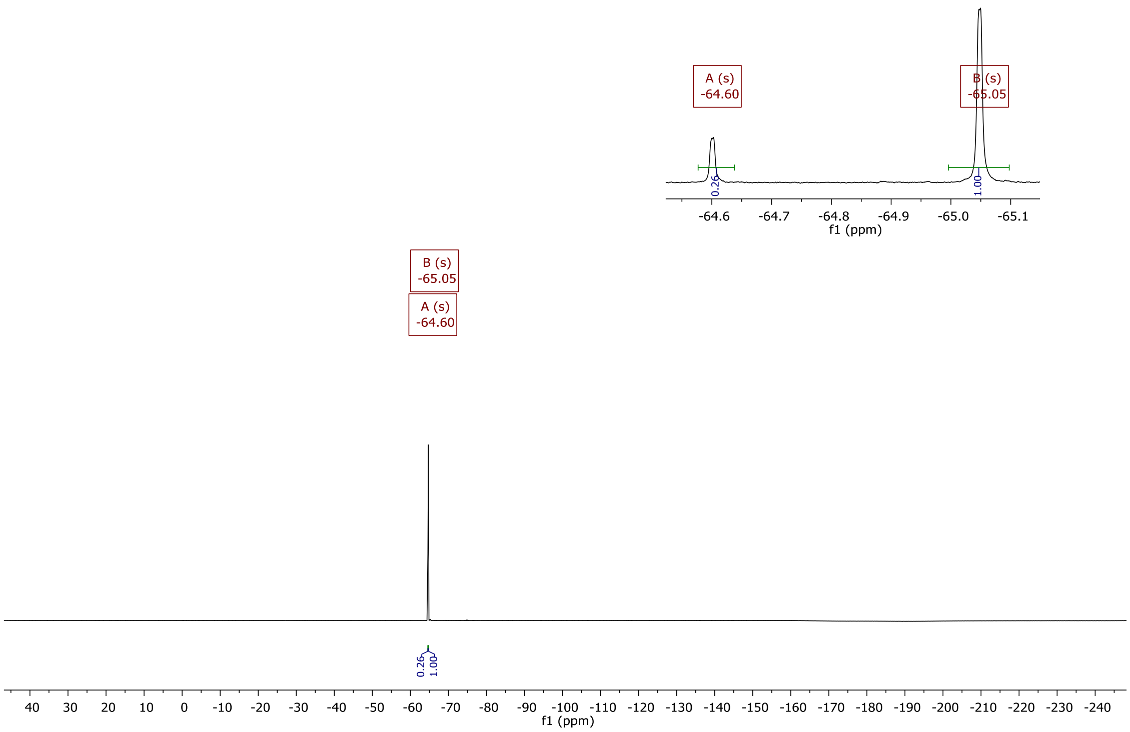

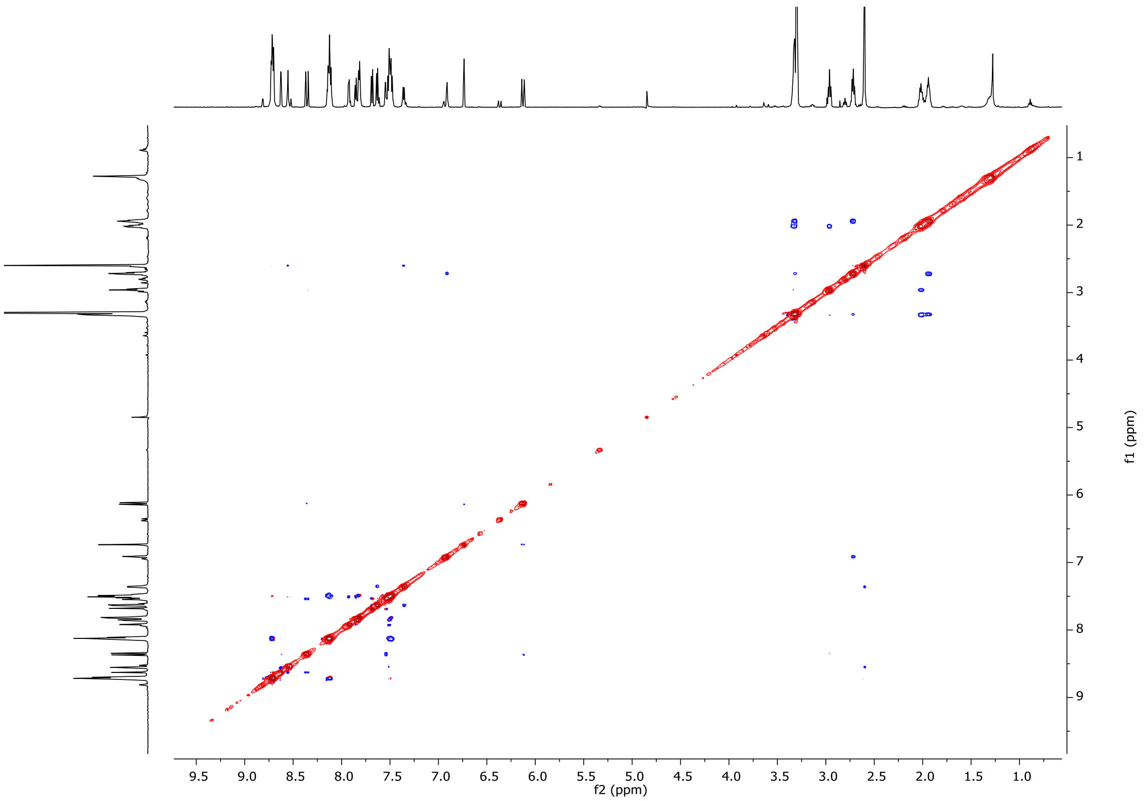

Figure S68. ^{19}F and 2D NOESY spectra of compound **Ru6** in Methanol-d_4.

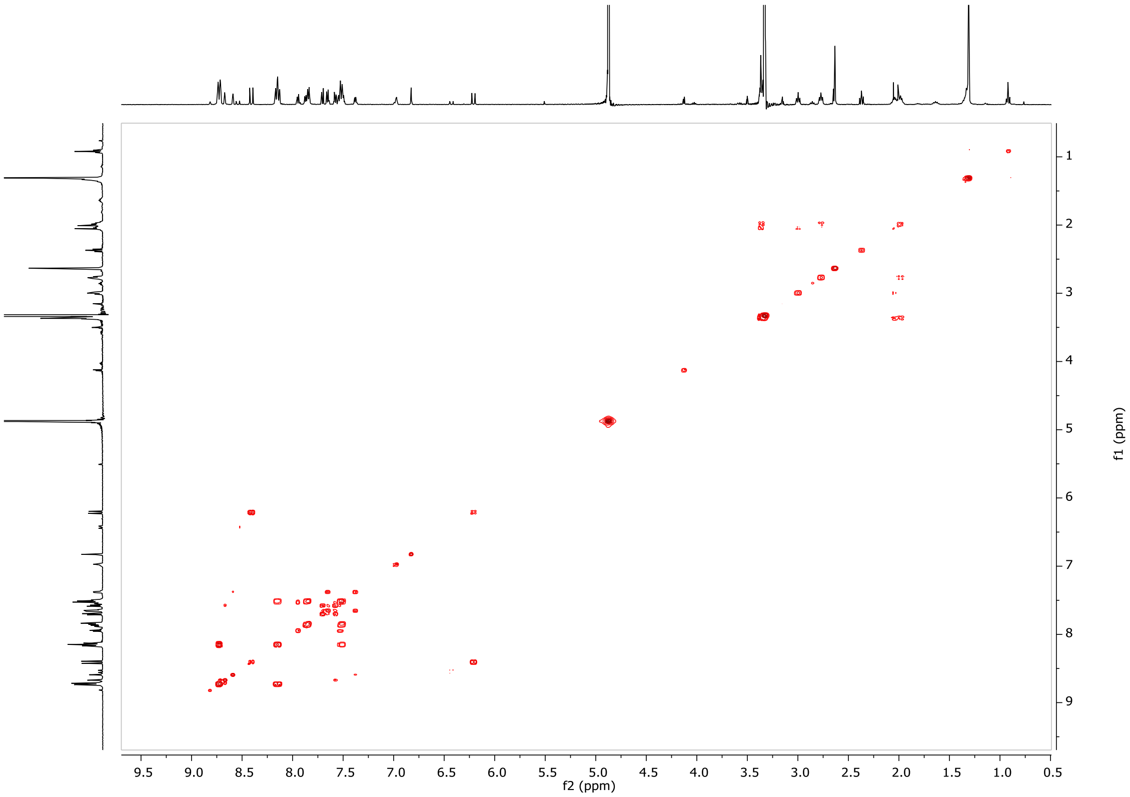

Figure S69. 2D COSY spectrum of compound **Ru6** in Methanol-d_4.

Ru7

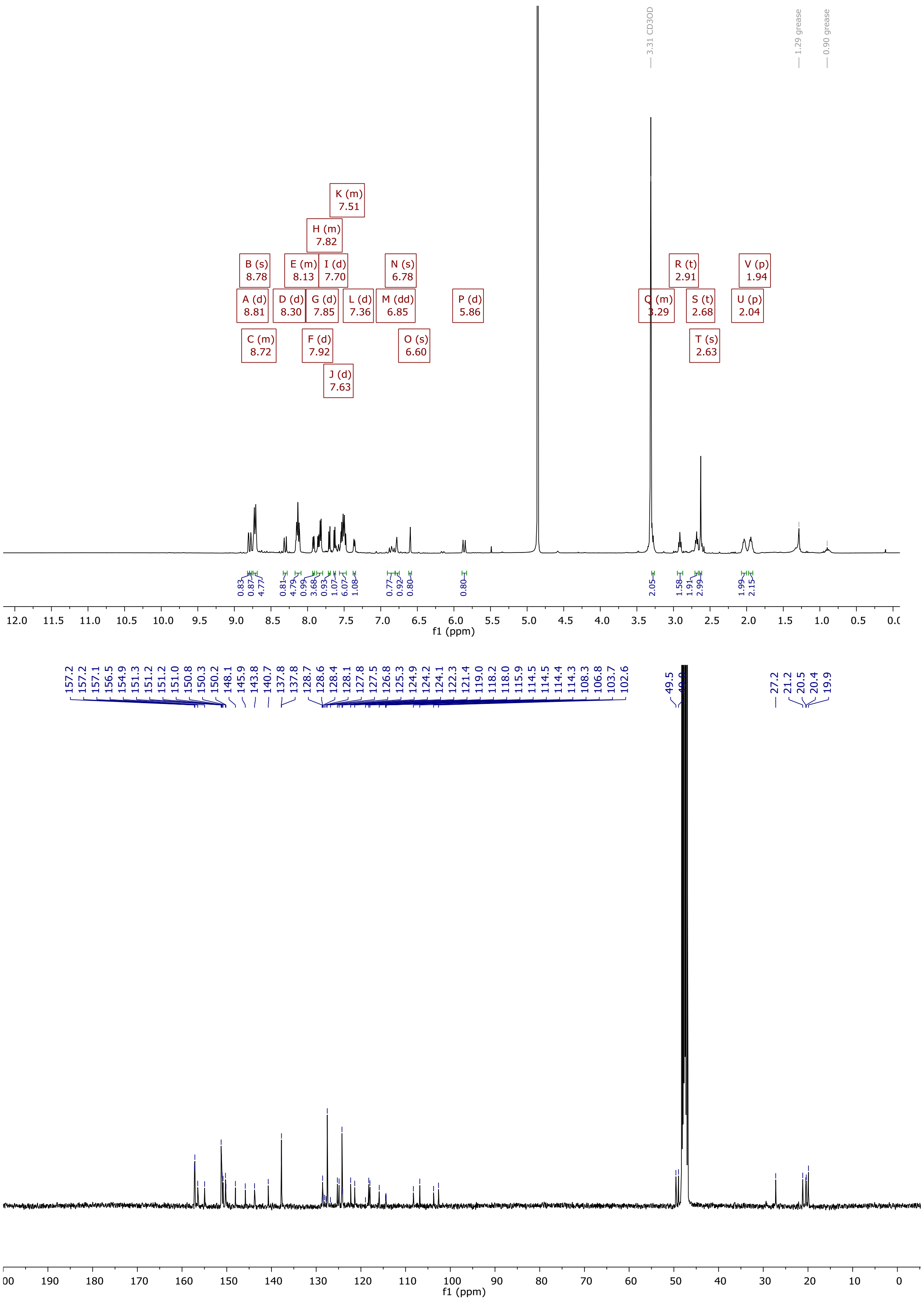

Figure S70. ^{1}H and ^{13}C NMR spectra of **Ru7** in Methanol-d_4

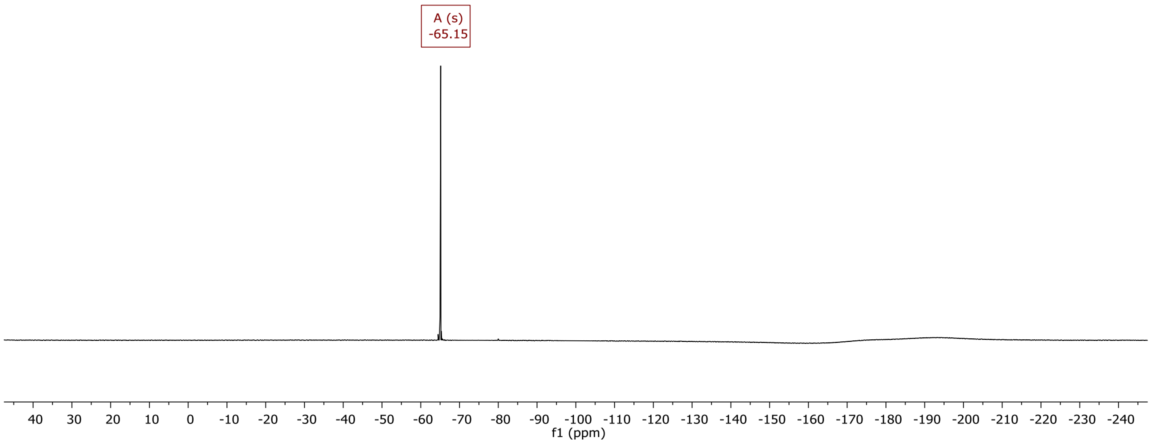

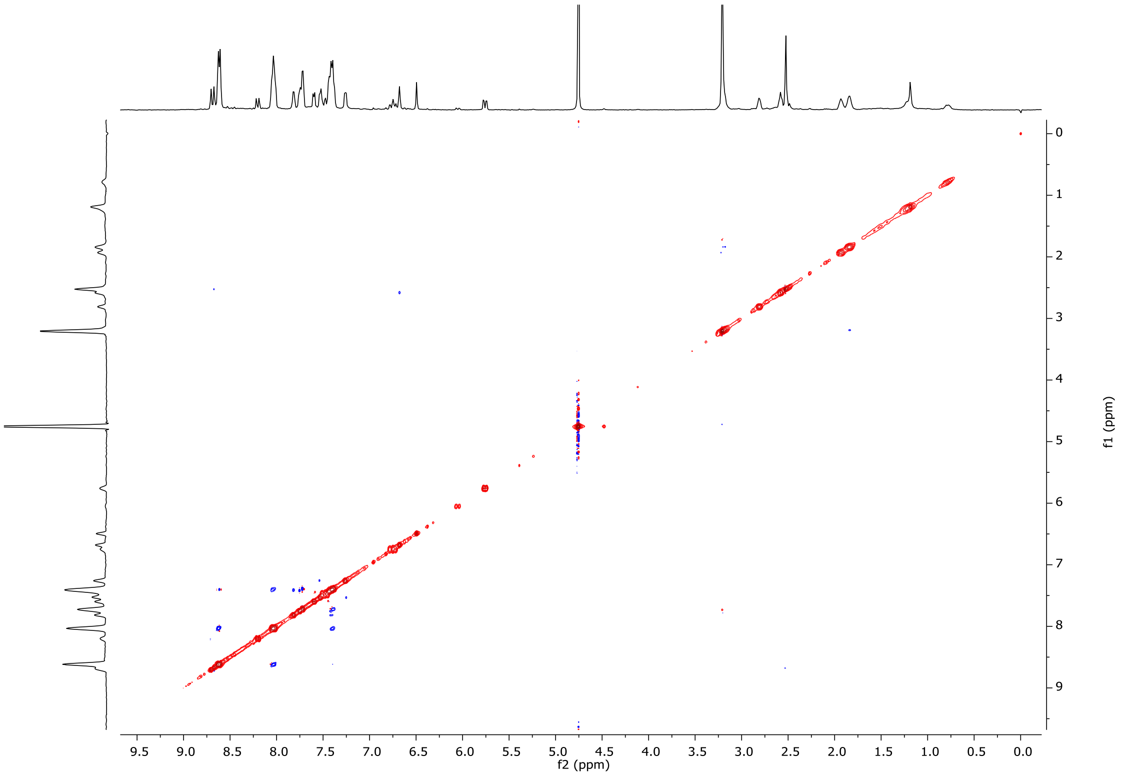

Figure S71. ^{19}F and 2D NOESY spectra of compound **Ru7** in Methanol-d_4.

Compound 13

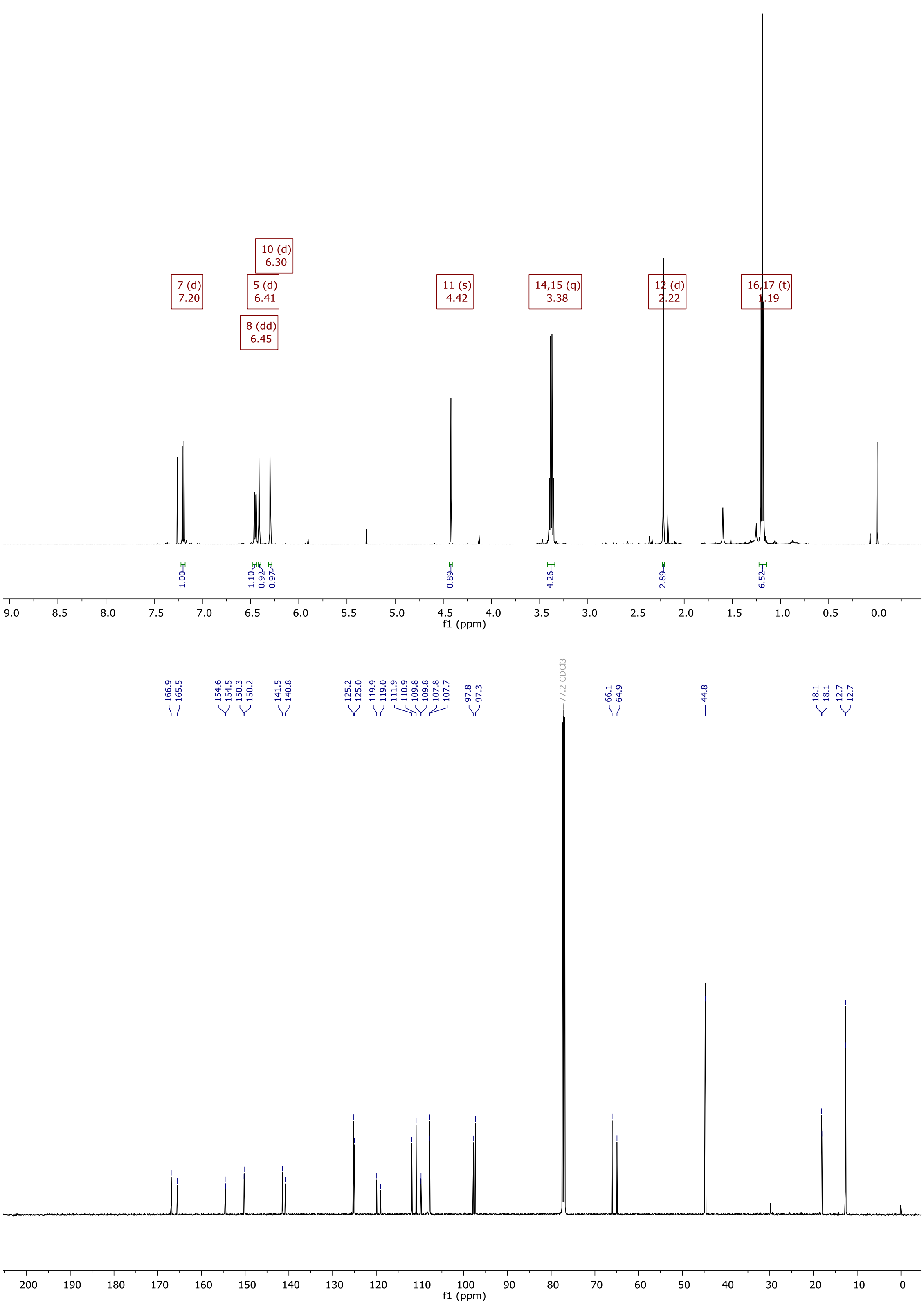

Figure S72. ^{1}H and ^{13}C NMR spectra of compound **13** in $CDCl_3$.

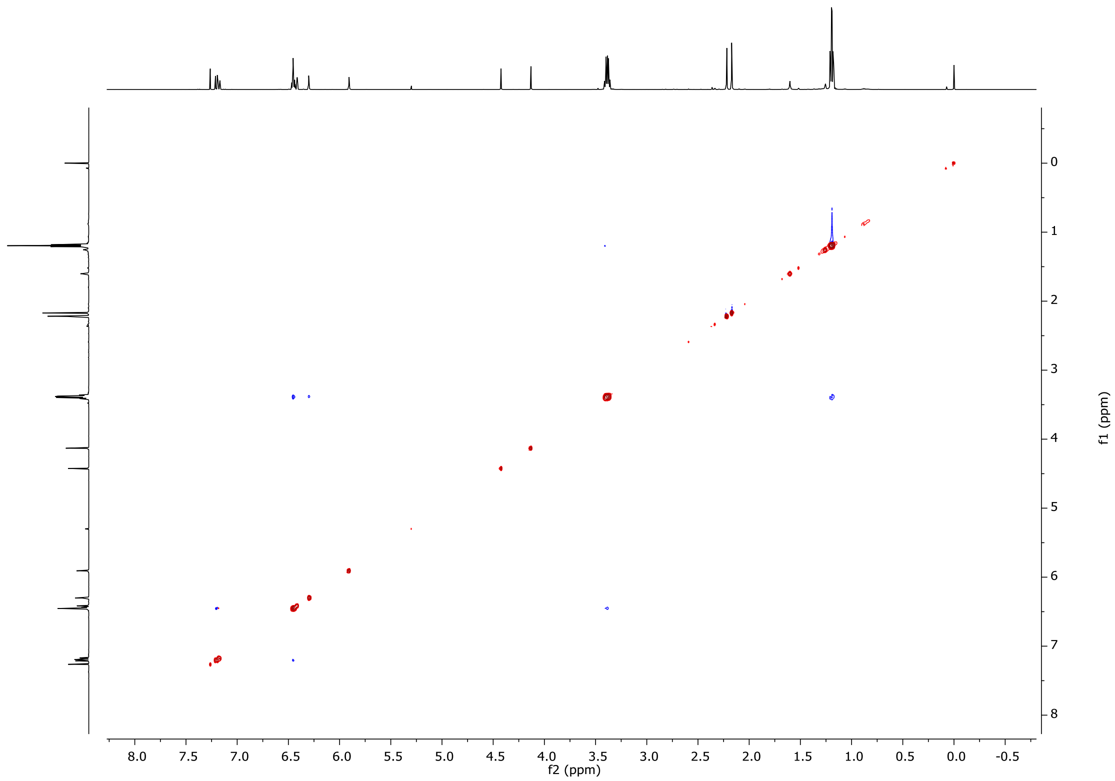

Figure S73 2D NOESY spectra of compound **13** in $CDCl_3$.

Compound 8

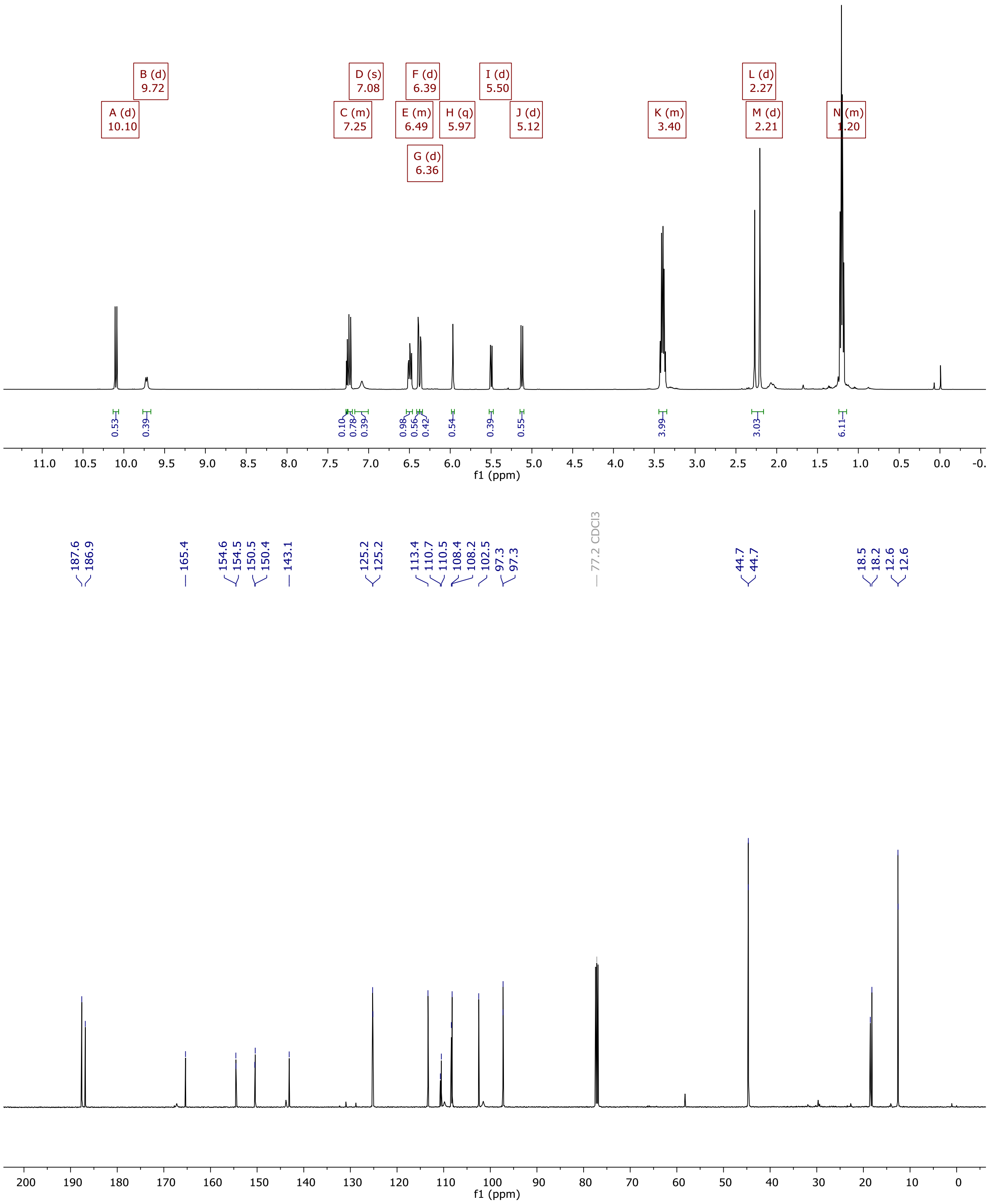

Figure S74. ^{1}H and ^{13}C NMR spectra of compound **8** in $CDCl_3$.

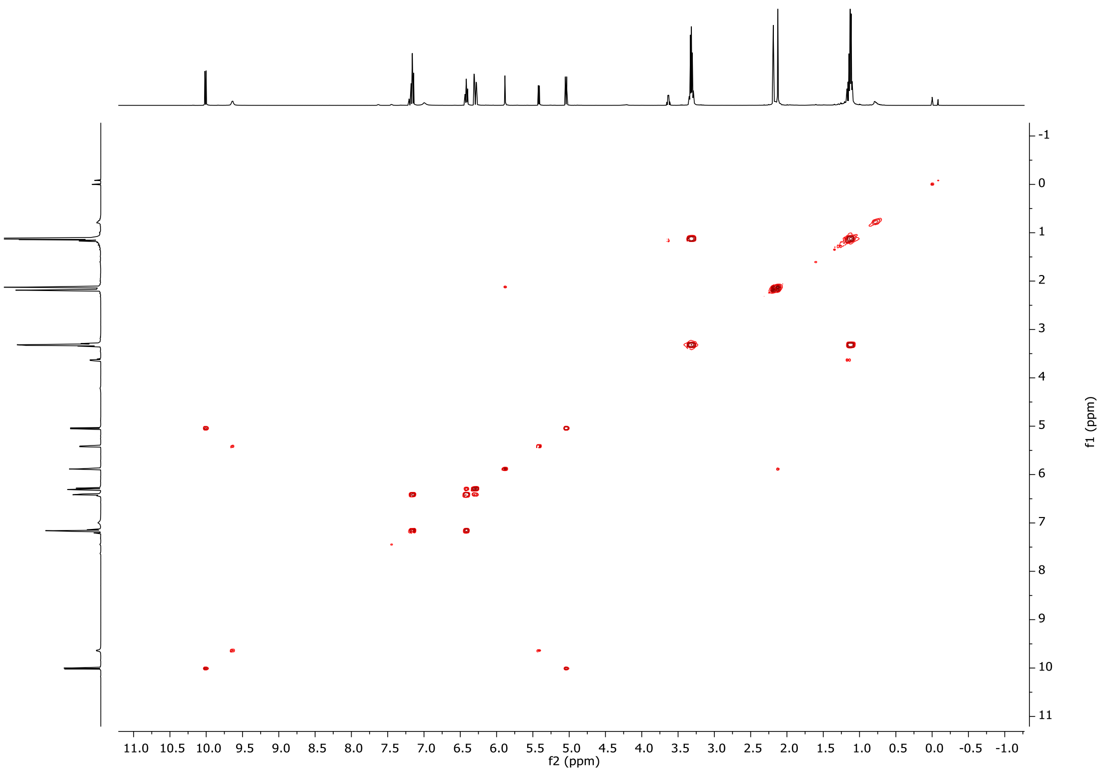

Figure S75. 2D COSY spectrum of compound **8** in $CDCl_3$.

Compound 14

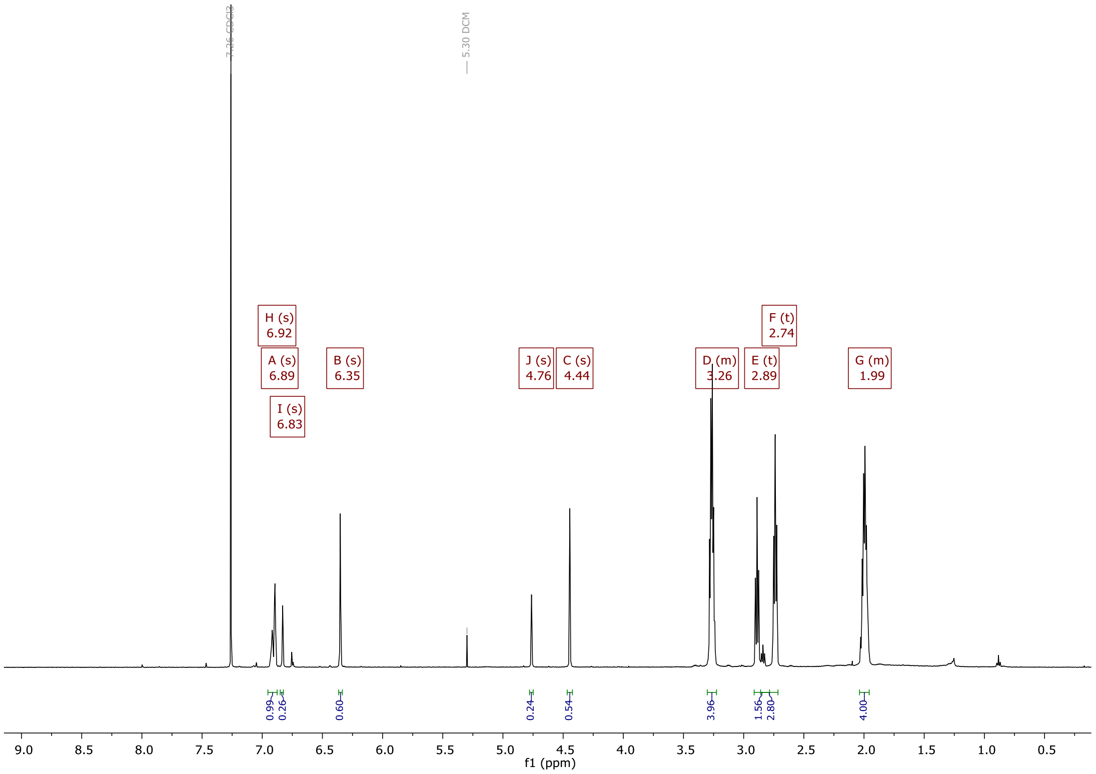

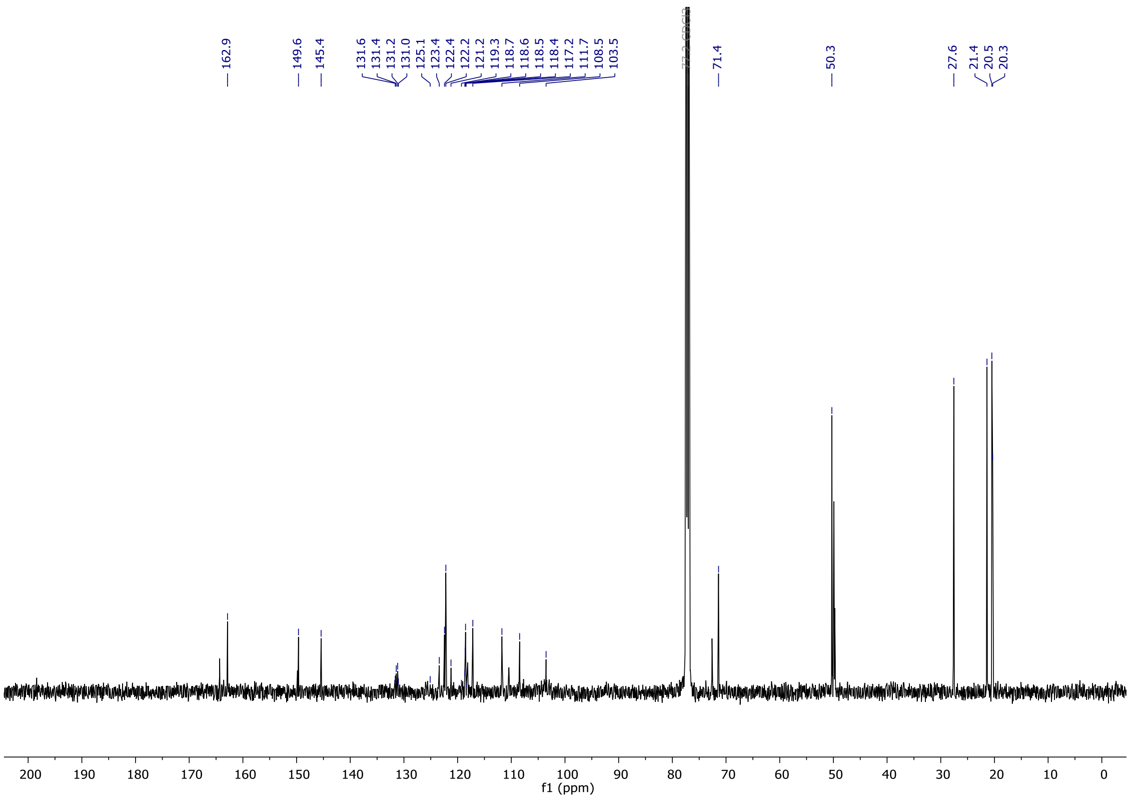

Figure S76. ^{1}H and ^{13}C NMR spectra of compound **14** in $CDCl_3$.

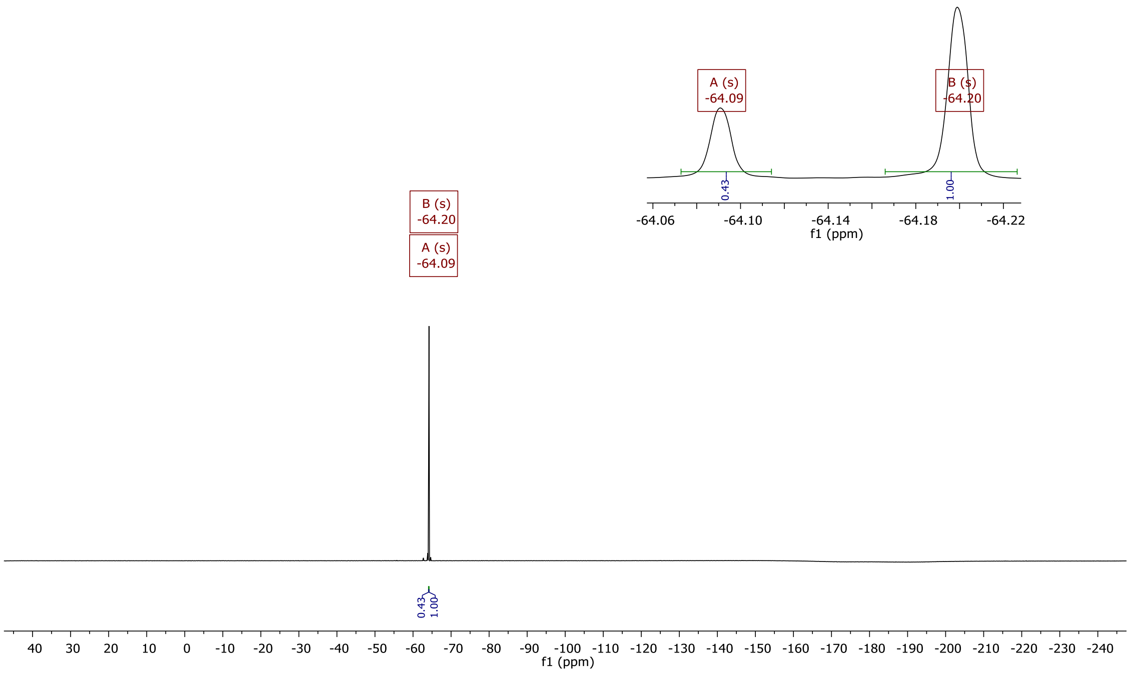

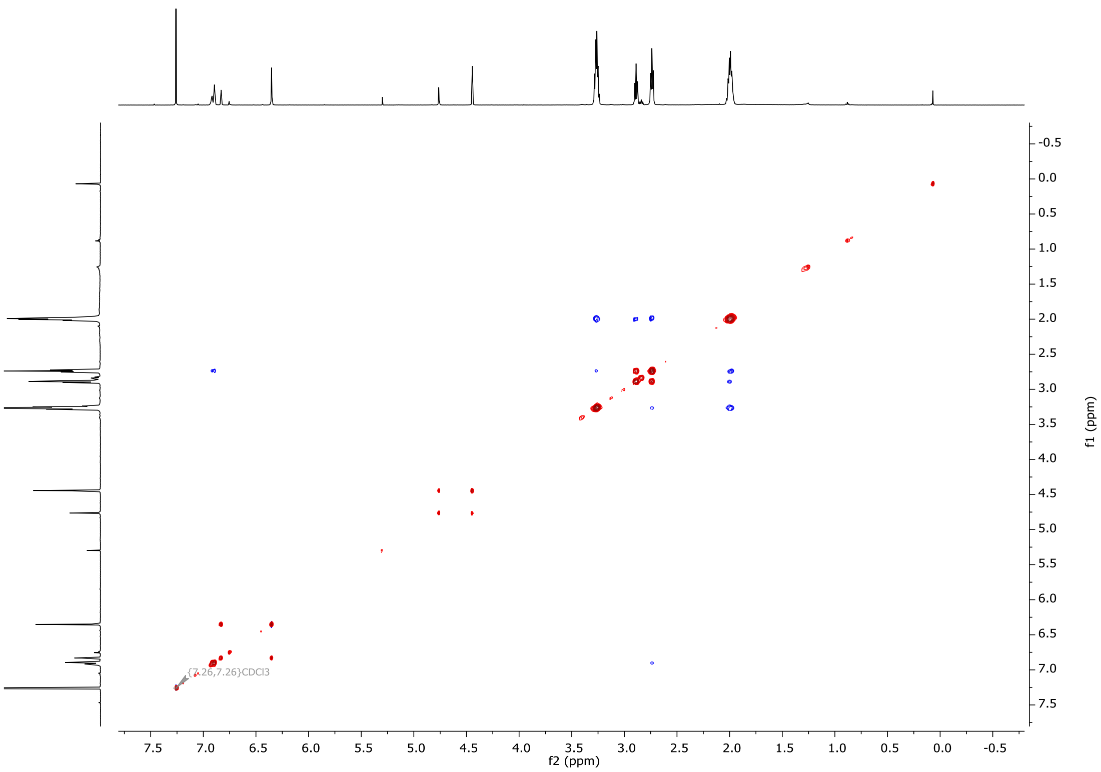

Figure S77. ^{19}F and 2D NOESY spectra of compound **14** in $CDCl_3$.

Compound 10

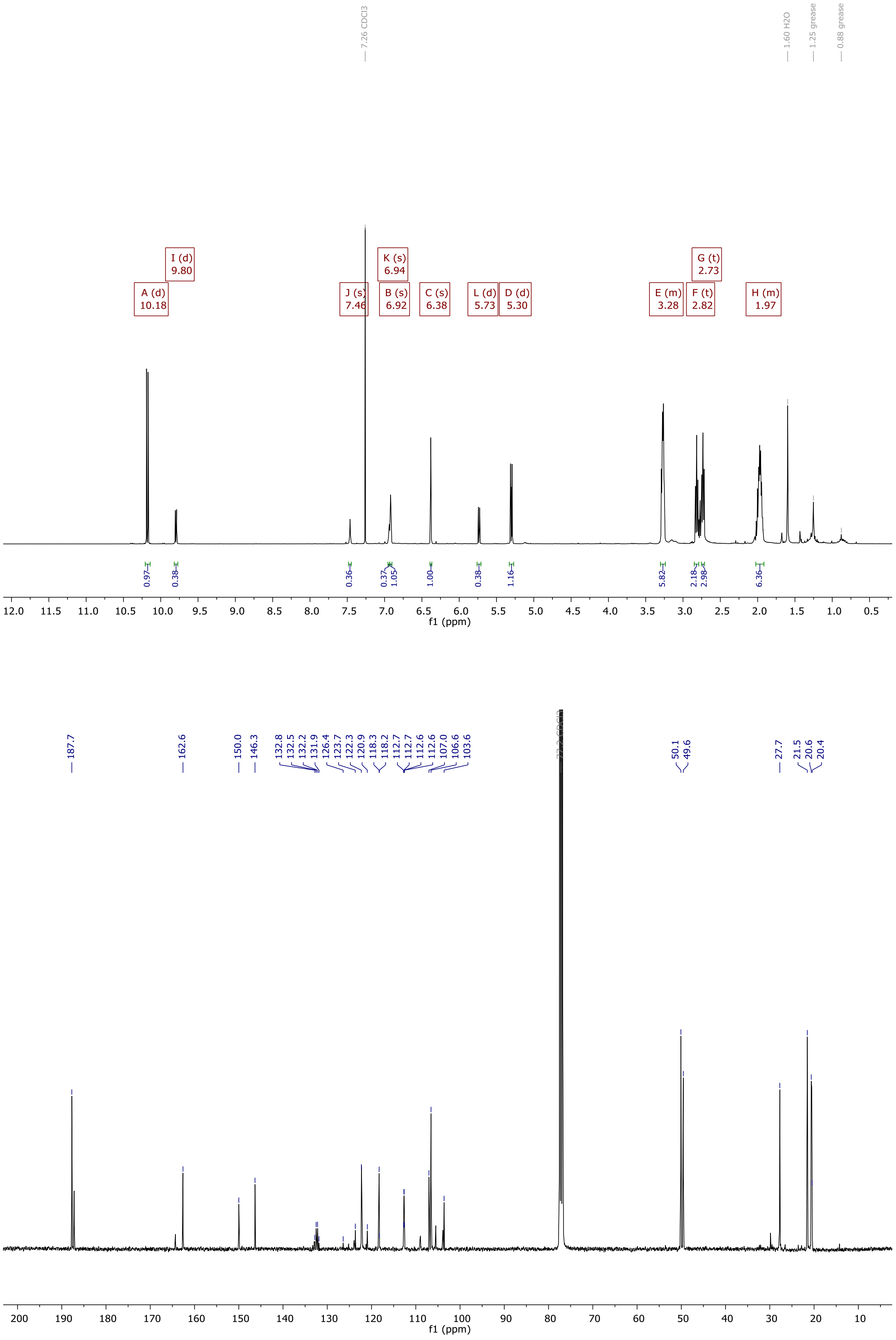

Figure S78. ^{1}H and ^{13}C NMR spectra of compound **10** in $CDCl_3$.

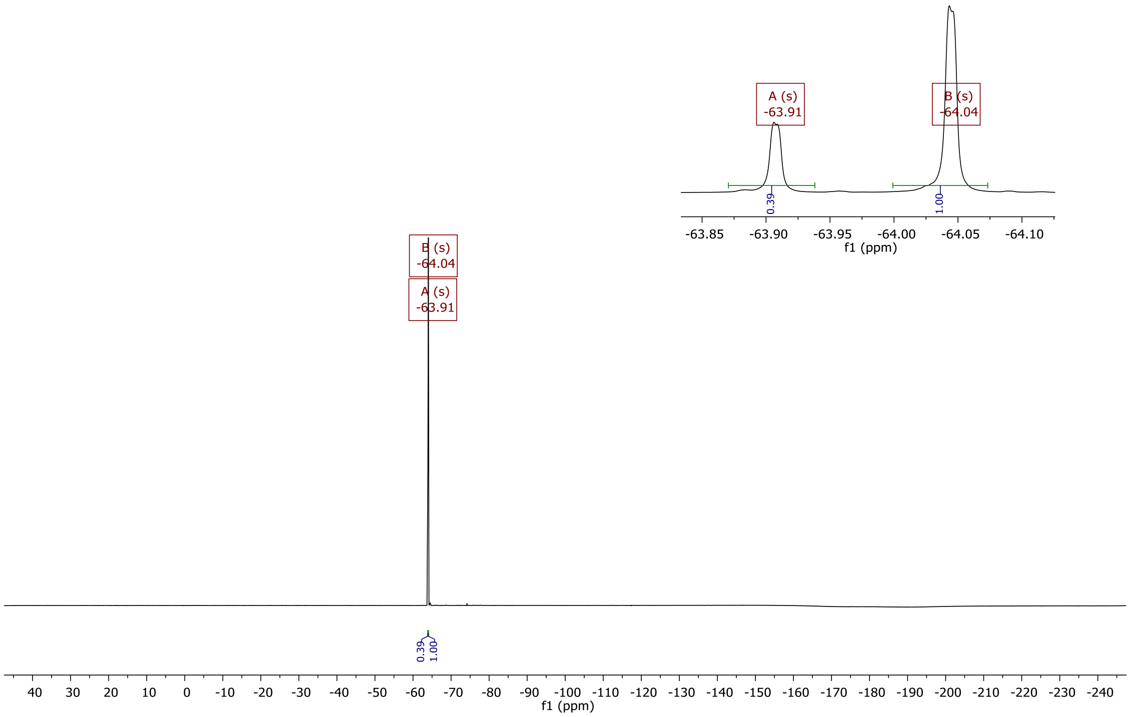

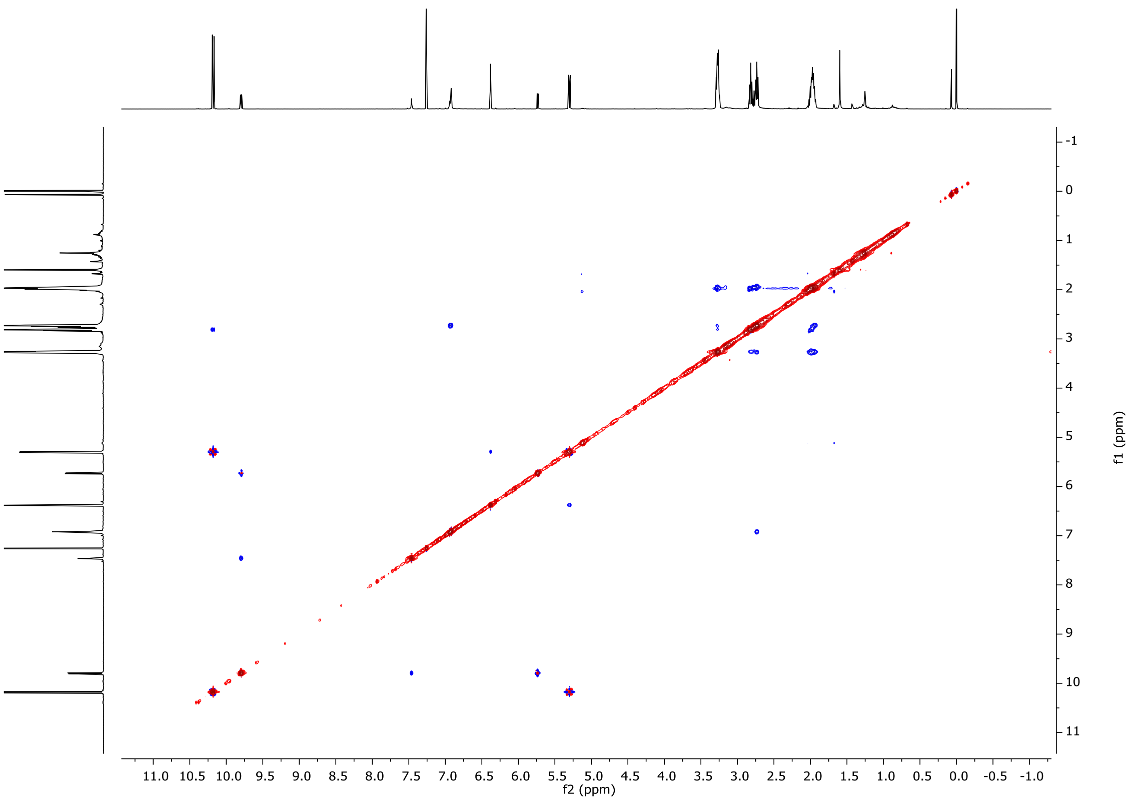

Figure S79. ^{19}F and 2D NOESY spectra of compound **10** in $CDCl_3$.

Compound 15

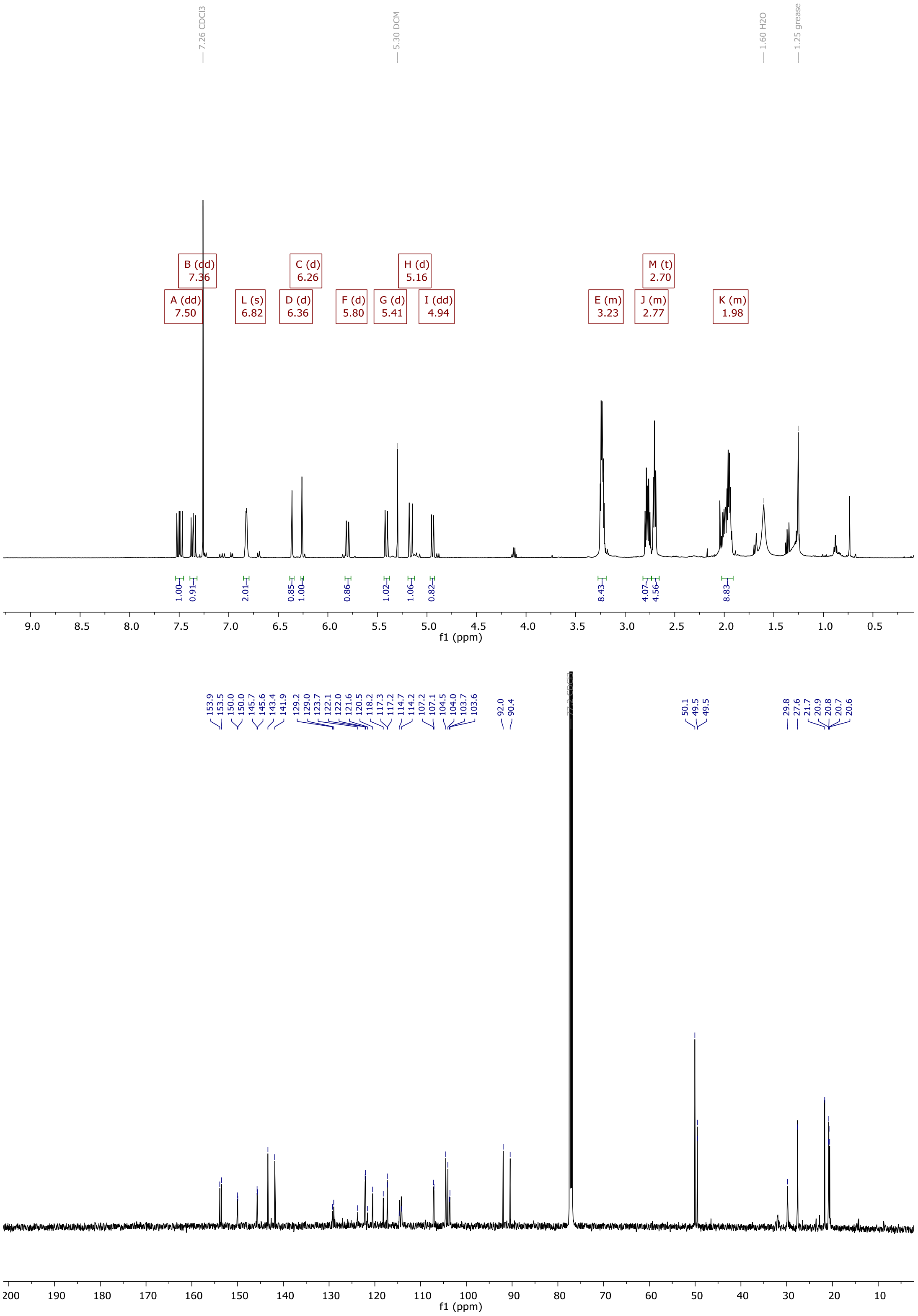

Figure S80. ^{1}H and ^{13}C NMR spectra of compound **15** in $CDCl_3$.

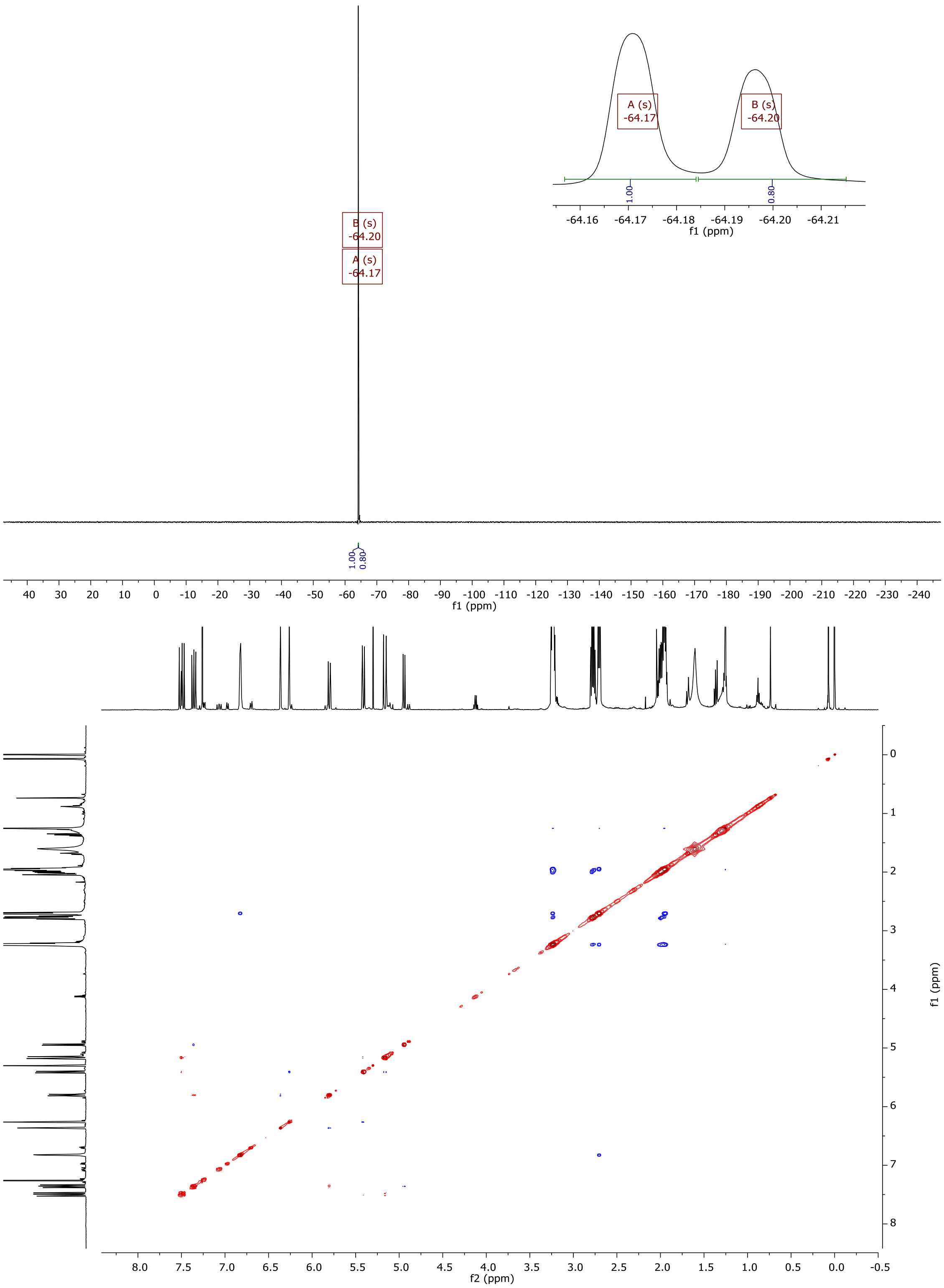

Figure S81. ^{19}F and 2D NOESY spectra of compound **15** in $CDCl_3$.

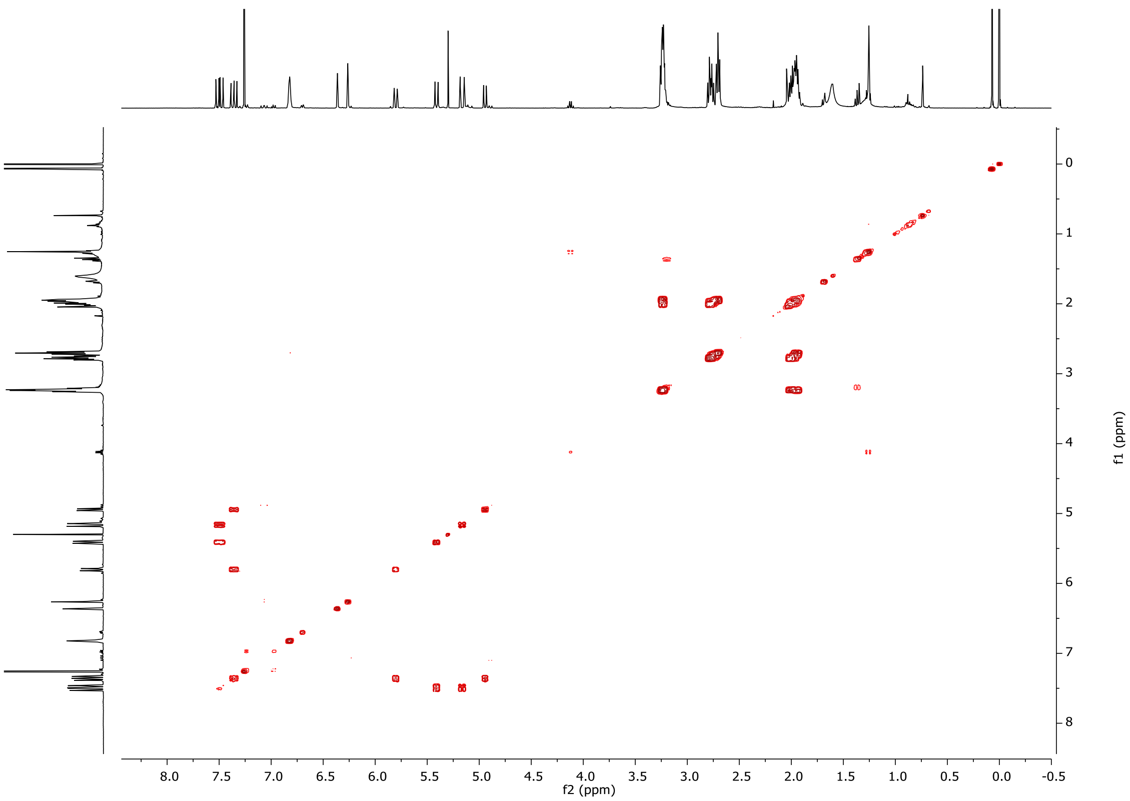

Figure S82. 2D COSY spectrum of compound **15** in $CDCl_3$.

Compound 11

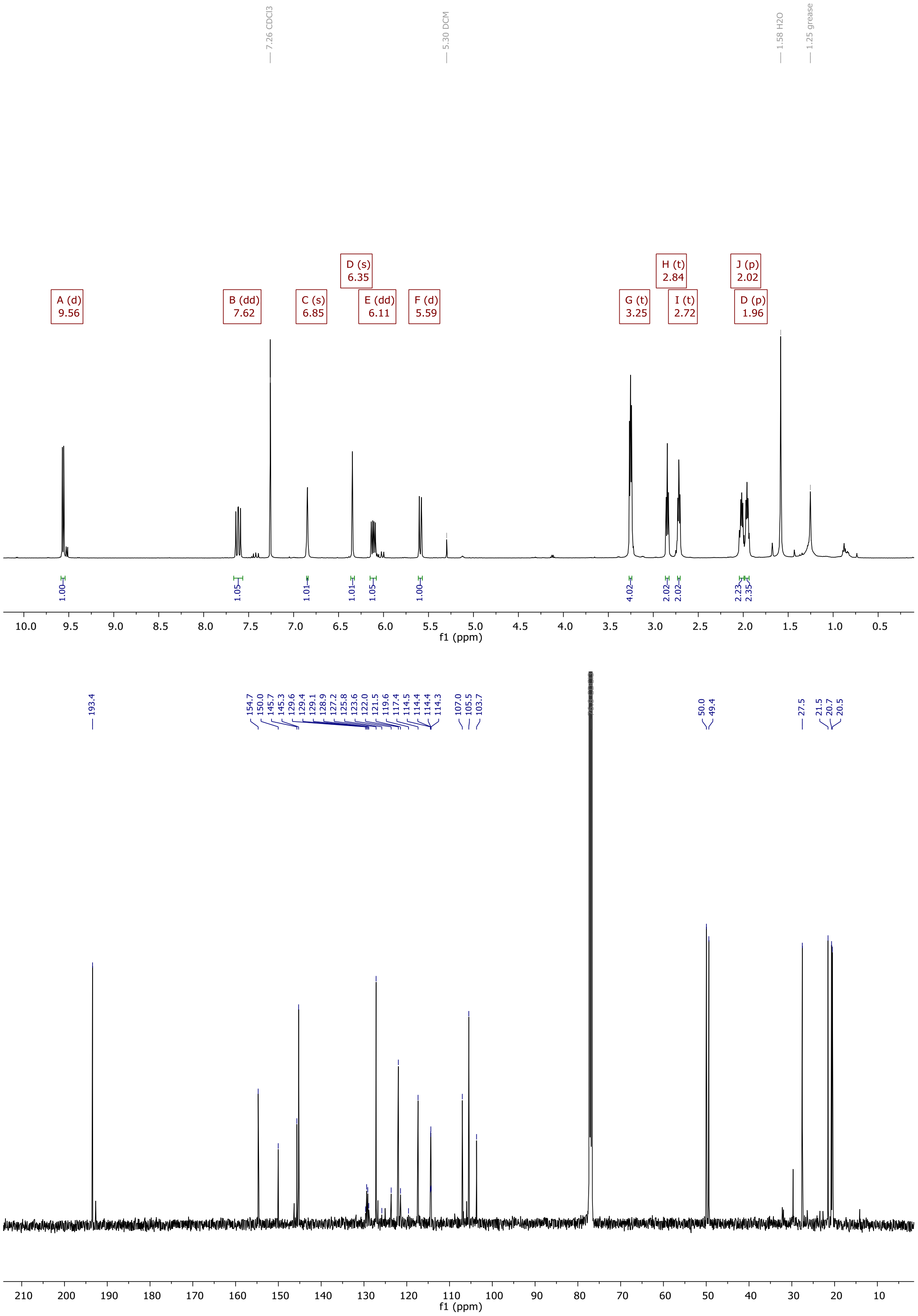

Figure S83. ^{1}H and ^{13}C NMR spectra of compound **11** in $CDCl_3$.

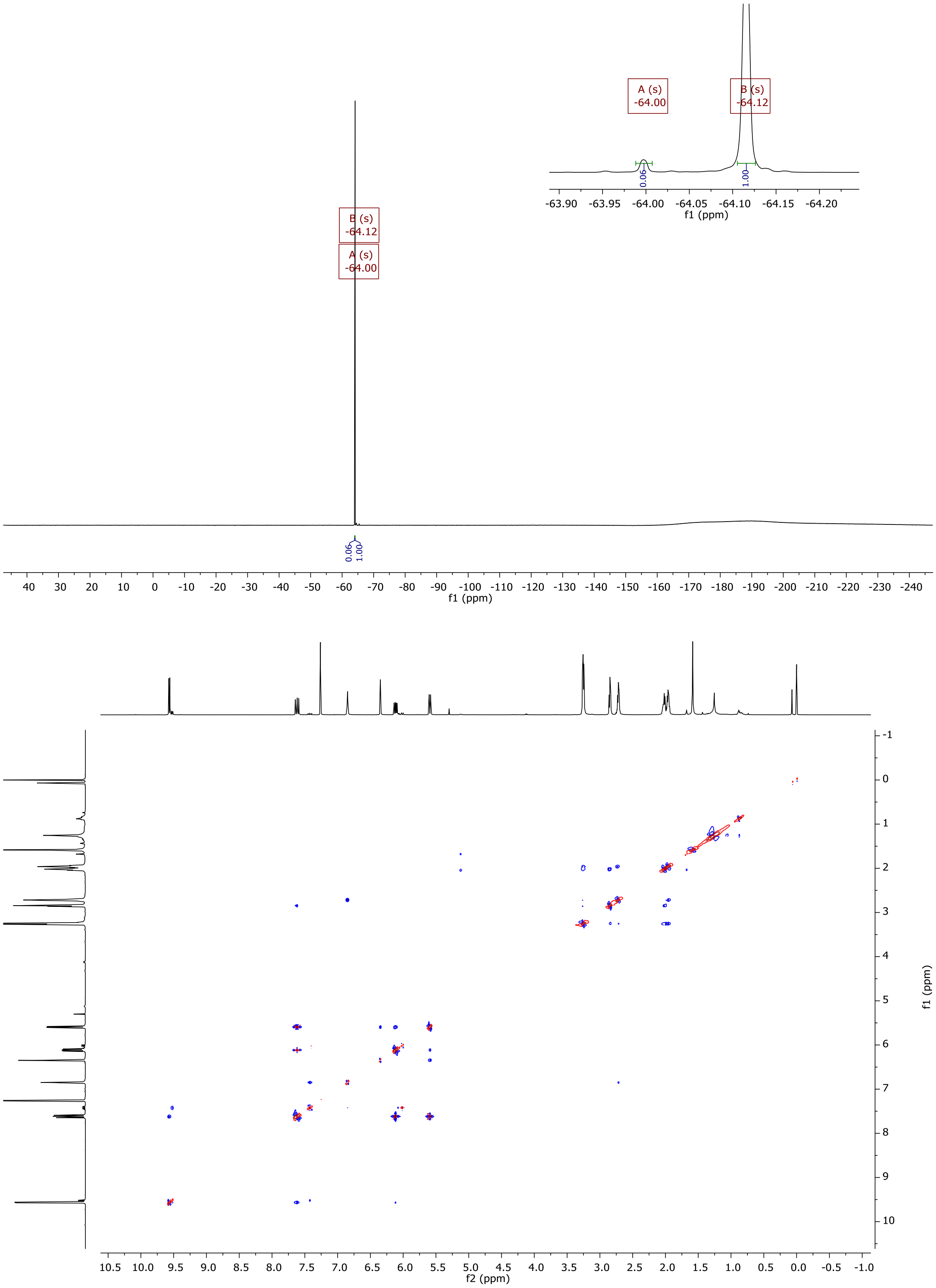

Figure S84. ^{19}F and 2D NOESY spectra of compound **11** in $CDCl_3$.

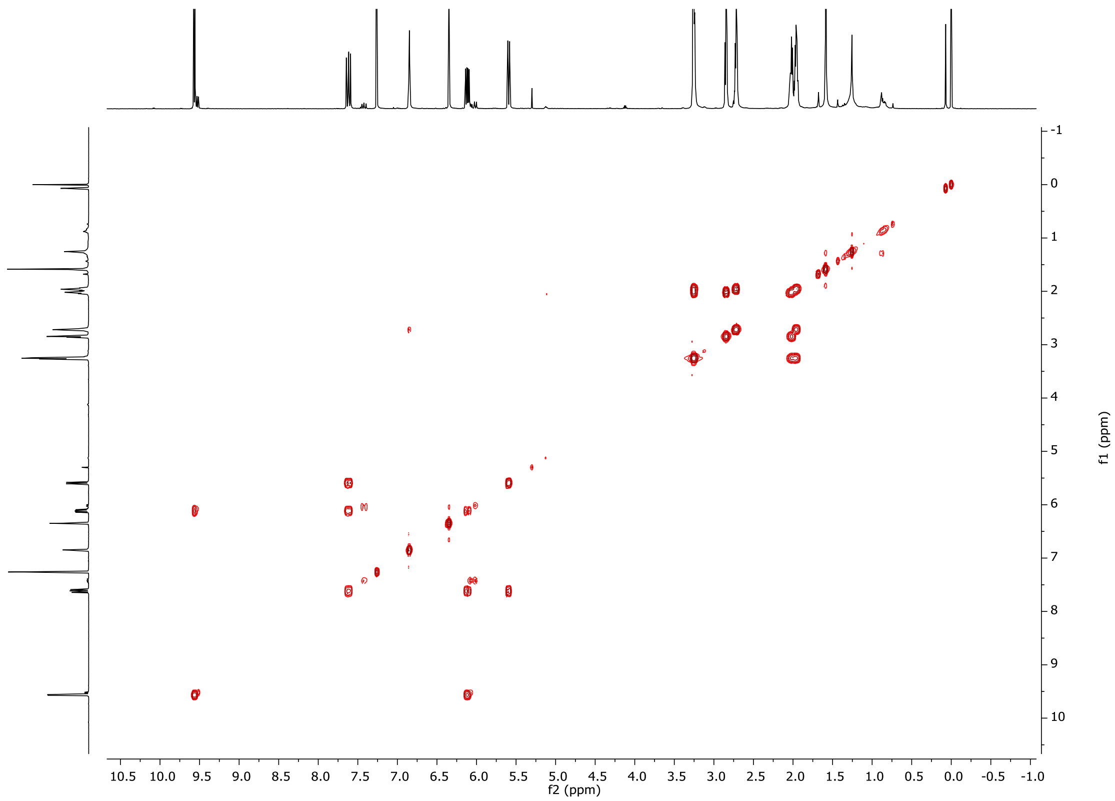

Figure S85. 2D COSY spectrum of compound **11** in $CDCl_3$.

Ru complex 12:

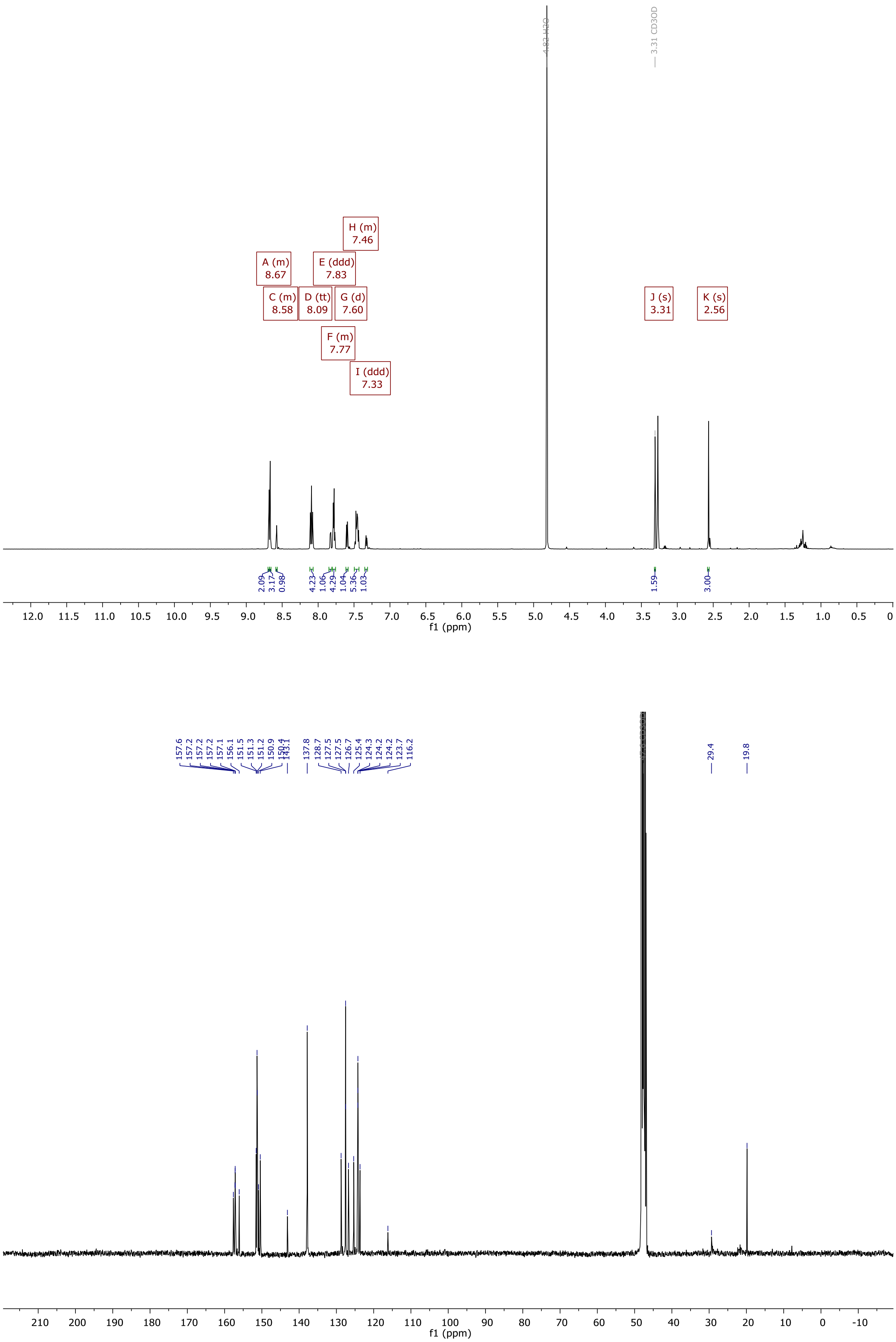

Figure S86. ^{1}H and ^{13}C NMR spectra of Ru complex **12** in CD_3OD.

9.- References

[1] A. Romieu, J.-A. Richard. An expedient synthesis of *N,N*-dialkylamino-dihydroxanthene-pyrylium conjugated near-infrared fluorescent dyes. *Tetrahedron Lett.* **2016**, *57*, 317–320.

[2] A. Gandioso, E. Izquierdo-García, P. Mesdom, P. Arnoux, N. Demeubayeva, P. Burckel, B. Saubaméa, M. Bosch, C. Frochot, V. Marchán, G. Gasser. Ru(II)-Cyanine Complexes as Promising Photodynamic Photosensitizers for the Treatment of Hypoxic Tumours with Highly Penetrating 770 nm Near-Infrared Light. *Chem. Eur. J.* **2023**, *29*, e202301742.

[3] Gaussian 16, Revision C.01, Frisch, M. J.; Trucks, G. W.; Schlegel, H. B.; Scuseria, G. E.; Robb, M. A.; Cheeseman, J. R.; Scalmani, G.; Barone, V.; Petersson, G. A.; Nakatsuji, H.; Li, X.; Caricato, M.; Marenich, A. V.; Bloino, J.; Janesko, B. G.; Gomperts, R.; Mennucci, B.; Hratchian, H. P.; Ortiz, J. V.; Izmaylov, A. F.; Sonnenberg, J. L.; Williams-Young, D.; Ding, F.; Lipparini, F.; Egidi, F.; Goings, J.; Peng, B.; Petrone, A.; Henderson, T.; Ranasinghe, D.; Zakrzewski, V. G.; Gao, J.; Rega, N.; Zheng, G.; Liang, W.; Hada, M.; Ehara, M.; Toyota, K.; Fukuda, R.; Hasegawa, J.; Ishida, M.; Nakajima, T.; Honda, Y.; Kitao, O.; Nakai, H.; Vreven, T.; Throssell, K.; Montgomery, J. A., Jr.; Peralta, J. E.; Ogliaro, F.; Bearpark, M. J.; Heyd, J. J.; Brothers, E. N.; Kudin, K. N.; Staroverov, V. N.; Keith, T. A.; Kobayashi, R.; Normand, J.; Raghavachari, K.; Rendell, A. P.; Burant, J. C.; Iyengar, S. S.; Tomasi, J.; Cossi, M.; Millam, J. M.; Klene, M.; Adamo, C.; Cammi, R.; Ochterski, J. W.; Martin, R. L.; Morokuma, K.; Farkas, O.; Foresman, J. B.; Fox, D. J. Gaussian, Inc., Wallingford CT, 2016.

[4] C. Adamo, V. Barone. Toward reliable density functional methods without adjustable parameters: The PBE0 model. *J. Chem. Phys.* **1999**, *110*, 6158-6170.

[5] S. Grimme, J. Antony; S. Ehrlich; H. Krieg. A consistent and accurate ab initio parametrization of density functional dispersion correction (DFT-D) for the 94 elements H-Pu. *J. Chem. Phys.* **2010**, *132*, 154104.

[6] M. Cossi, N. Rega, G. Scalmani, V. Barone. Energies, structures, and electronic properties of molecules in solution with the C-PCM solvation model. *J. Comput. Chem.* **2003**, *24*, 669–681.

[7] Y. Zhao, D. G. Truhlar. The M06 suite of density functionals for main group thermochemistry, thermochemical kinetics, noncovalent interactions, excited states, and transition elements: two new functionals and systematic testing of four M06-class functionals and 12 other functionals. *Theor. Chem. Acc.* **2008**, *120*, 215–241.

[8] D. Abad-Montero, A. Gandioso, E. Izquierdo-García, S. Chumillas, A. Rovira, M. Bosch, M. Jordà-Redondo, D. Castaño, J. Bonelli, V. V. Novikov, A. Deyà, J. L. Hernández, J. Galino, M. E. Alberto, A. Francés-Monerris, S. Nonell, G. Gasser, V. Marchán. *J. Am. Chem. Soc.* **2025**, *147*, 7360–7376.

[9] H. D. Cole, A. Vali, J. A. Roque III, G. Shi, A. Talgatov, G. Kaur, A. Francés-Monerris, M. E. Alberto, C. G. Cameron, S. A. McFarland. Ru(II) Oligothienyl Complexes with Fluorinated Ligands: Photophysical, Electrochemical, and Photobiological Properties. *Inorg. Chem.* **2024**, *63*, 21, 9735–9752.

[10] H. D. Cole, A. Vali, J. A. III Roque, G. Shi, G. Kaur, R. O. Hodges, A. Francés-Monerris, M. E. Alberto, C. G. Cameron, S. A. McFarland. Ru(II) Phenanthroline-Based Oligothienyl Complexes as Phototherapy Agents. *Inorg. Chem*. **2023**, *62*, 21181-21200.

[11] M. E. Alberto, A. Francés-Monerris. A multiscale free energy method reveals an unprecedented photoactivation of a bimetallic Os(II)–Pt(II) dual anticancer agent. *Phys. Chem. Chem. Phys*. **2022**, *24*, 19584-19594

[12] S. Hirata, M. Head-Gordon, Time-dependent density functional theory within the Tamm–Dancoff approximation. *Chem. Phys. Lett.* **1999**, *314*, 291–299.

[13] R. L. Martin. Natural transition orbitals. *J. Chem. Phys.* **2003**, *118*, 4775–4777.

[14] L. Skripnikov, 2020, Chemissian 4.67. www.chemissian.com.

[15] a) N. Adarsh, R. R. Avirah, D. Ramaiah. Tuning photosensitized singlet oxygen generation efficiency of novel aza-BODIPY dyes. *Org. Lett*. **2010**, *12*, 5720-5723; b) W. Li, L. Li, H. Xiao, R. Qi, Y. Huang, Z. Xie, X. Jing, H. Zhang. Iodo-BODIPY: a visible-light-driven, highly efficient and photostable metal-free organic photocatalyst. *RSC Adv*. **2013**, *3*, 13417-13421; c) Z. Lv, H. Wei, Q. Li, X. Su, S. Liu, K. Y. Zhang, W. Lv, Q. Zhao, X. Li, W. Huang. Achieving efficient photodynamic therapy underboth normoxia and hypoxia using cyclometalated Ru(II) photosensitizer through type I photochemicalprocess. *Chem. Sci*. **2018**, *9*, 502-512.

[16] M. López-Corrales, A. Rovira, A. Gandioso, M. Bosch, S. Nonell, V. Marchán. Transformation of COUPY Fluorophores into a Novel Class of Visible-Light-Cleavable Photolabile Protecting Groups. *Chem. Eur. J.* **2020**, *26*, 16222-16227.

[17] X. Zhang, G. Q. Zhang, J. Zhu. Methylated unsymmetric BODIPY compounds: synthesis, high fluorescence quantum yield and long fluorescence time. *J. Fluoresc*. **2019**, *29*, 407-416.

[18] U. Yoshiharu. Determination of quantum yield of singlet oxygen formation by photosensitization. *Chem. Lett*. **1973**, *2*, 743-744.

[19] J. Llano, J. Raber, L. A. Eriksson. Theoretical study of phototoxic reactions of psoralens. *J. Photochem. Photobiol., A.* **2003**, *154*, 235-243.

[20] A. Roque, P. C. Barrett, H. D. Cole, L. M. Lifshits, G. Shi, S. Monro, D. von Dohlen, S. Kim, N. Russo, G. Deep, C. G. Cameron, M. E. Alberto, S. A. McFarland. Breaking the barrier: an osmium photosensitizer with unprecedented hypoxic phototoxicity for real world photodynamic therapy. *Chem. Sci.* **2020**, *11*, 9784–9806.

[21] M. Ayoubi-Chianeh, F. Jafarpour. Theoretical study of new promising conjugated psoralens in psoralen ultraviolet A therapy. *J. Phys. Org. Chem*. **2022**, *35*, e4308.

[22] M. Spiegel, C. Adamo. Tuning the Photophysical Properties of Ru(II) Photosensitizers for PDT by Protonation and Metallation: A DFT Study. *J. Phys. Chem. A* **2023**, *127*, 3625–3635.

[23] J. A. Roque, P. C. Barrett, H. D. Cole, L. M. Lifshits, G. Shi, S. Monro, D. von Dohlen, S. Kim, N. Russo, G. Deep, C. G. Cameron, M. E. Alberto, S. A. McFarland. *Chem. Sci.* **2020**, *11*, 9784–9806.

[24] V. V Pavlishchuk, A. W. Addison. Conversion constants for redox potentials measured versus different reference electrodes in acetonitrile solutions at 25°C. *Inorg. Chim. Acta* **2000**, *298*, 97-102.

[25] J. Schindelin, I. Arganda-Carreras, E. Frise, V. Kaynig, M. Longair, T. Pietzsch, S. Preibisch, C. Rueden, S. Saalfeld, B. Schmid, J. Y. Tinevez, D. J. White, V. Hartenstein, K. Eliceiri, P. Tomancak, A. Cardona. Fiji: an open-source platform for biological-image analysis. *Nat. Methods* **2012**, *9*, 676−682.
